# A Novel Natural Siderophore Antibiotic Conjugate Reveals a Chemical Approach to Macromolecule Coupling

**DOI:** 10.1021/acscentsci.3c00965

**Published:** 2023-11-10

**Authors:** Thibault Caradec, Ernesto Anoz-Carbonell, Ravil Petrov, Muriel Billamboz, Kevin Antraygues, Francois-Xavier Cantrelle, Emmanuelle Boll, Delphine Beury, David Hot, Herve Drobecq, Xavier Trivelli, Ruben C. Hartkoorn

**Affiliations:** ∇ Université Lille, CNRS, Inserm, CHU Lille, Institut Pasteur Lille, U1019 - UMR 9017 - CIIL - Center for Infection and Immunity of Lille, F-59000 Lille, France; ‡ Université Lille, Inserm, CHU Lille, Institut Pasteur de Lille, U1167 - RID-AGE - Risk Factors and Molecular Determinants of Aging-Related Diseases, F-59000 Lille, France; § JUNIA, Health and Environment, Laboratory of Sustainable Chemistry and Health, F-59000 Lille, France; ∥ Université Lille, Inserm, Institut Pasteur de Lille, U1177 - Drugs and Molecules for Living Systems, F-59000 Lille, France; ⊥ CNRS, EMR9002 BSI Integrative Structural Biology, 59000 Lille, France; # Université Lille, CNRS, Inserm, CHU Lille, Institut Pasteur de Lille, UMR2014 - US41 - PLBS-Plateformes Lilloises de Biologie & Santé, F-59000 Lille, France; 7 Université Lille, CNRS, INRAE, Centrale Lille, Université d’Artois, FR 2638 - IMEC - Institut Michel-Eugène Chevreul, 59000 Lille, France

## Abstract

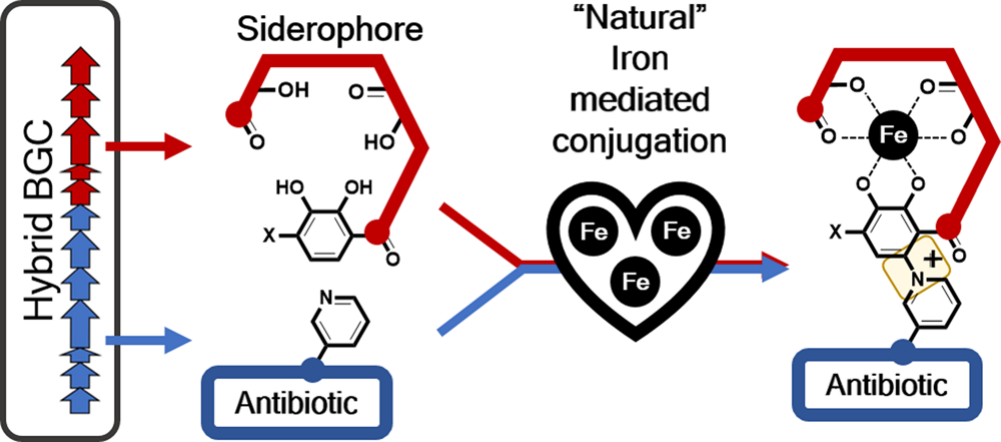

Inspired by natural sideromycins, the conjugation of antibiotics to siderophores is an attractive strategy to facilitate “Trojan horse” delivery of antibiotics into bacteria. Genome analysis of a soil bacterium, *Dactylosporangium fulvum,* found a “hybrid” biosynthetic gene cluster responsible for the production of both an antibiotic, pyridomycin, and a novel chlorocatechol-containing siderophore named chlorodactyloferrin. While both of these natural products were synthesized independently, analysis of the culture supernatant also identified a conjugate of both molecules. We then found that the addition of ferric iron to purified chlorodactyloferrin and pyridomycin instigated their conjugation, leading to the formation of a covalent bond between the siderophore-catechol and the pyridomycin-pyridine groups. Using model reactants, this iron-based reaction was found to proceed through a Michael-type addition reaction, where ferric iron oxidizes the siderophore-catechol group into its quinone form, which is then attacked by the antibiotic pyridyl-nitrogen to form the catechol–pyridinium linkage. These findings prompted us to explore if other “cargo” molecules could be attached to chlorodactyloferrin in a similar manner, and this was indeed confirmed with a pyridine-substituted TAMRA fluorophore as well as with pyridine-substituted penicillin, rifampicin, and norfloxacin antibiotic analogues. The resultant biomimetic conjugates were demonstrated to effectively enter a number of bacteria, with TAMRA–chlorodactyloferrin conjugates causing fluorescent labeling of the bacteria, and with penicillin and rifampicin conjugates eliciting antibiotic activity. These findings open up new opportunities for the design and facile synthesis of a novel class of biomimetic siderophore conjugates with antibiotic activity.

## Abstract 2

Iron can mediate coupling of catechol-containing siderophores with pyridine-containing antibiotics in a process that can be exploited to design custom siderophore conjugates.

## Introduction

Antibiotic drug resistant bacterial infections pose an ever-growing burden to global healthcare systems worldwide, with research and development into novel antibiotic innovations classed as a critical priority by the World Health Organization to help fight such drug resistant bacterial infections.^1−3^ An important hurdle in the development of novel antibiotics is to design antibiotics capable of effectively traversing the complex bacterial cell envelope that acts as a first line defense mechanism to antibiotics. This innate penetration barrier is often reinforced by efficient antibiotic efflux pumps that further prevent intracellular accumulation. The development of novel technologies to facilitate the delivery of antibiotics to their targets would therefore open up new doors to the development of future antibiotics.

The majority of currently used antibiotics are natural products (or their synthetic derivatives) that were thus naturally selected for activity and hence capable of penetrating bacteria to reach their targets. These natural antibiotics often have optimal physicochemical properties to enter bacteria by passive diffusion or though alternative routes such as through porins.^4^ Inspired by the properties of these natural products, major developments in medicinal chemistry allow for rational approaches to designing and improving antibiotic entry across the bacterial envelope of Gram-negative *E. coli* through the implementation of eNTRy rules.^5^ As an alternative approach for improved antibiotic penetration, a select number of natural products enter bacteria by hijacking bacterially active uptake systems for siderophore-mediated iron acquisition. Such “Trojan horse” antibiotics, also known as sideromycins, are often conjugates between an antibiotic and a siderophore (elegantly reviewed in refs (6−11)), but they may also constitute a free antibiotic (such as rifabutin uptake in *A. baumannii*(12)) or antibiotics able to bind iron such as cefiderocol.^13^

To date, three major natural sideromycin families have been described, namely albomycins,^14,15^ salmycins,^16^ and ferrimycins,^17,18^ as well as natural monoglucosylated enterobactin linked class IIb microcins^19^ (such as, microcin E492m^20,21^). When considered alone in their nonconjugated form, the antibacterial warheads of such conjugates often display low antibiotic activities because they are unable to effectively enter bacteria. The corresponding antibiotic conjugates show greatly enhanced penetration capacity as a result of hijacking bacterial siderophore-iron uptake systems.^22^ In the case of albomycin and salmycin, it was demonstrated that in addition to bacterial penetration, these antibiotic moieties are liberated (decoupled) from their respective siderophores once inside the bacteria, either by an intracellular peptidase for albomycin,^23^ and through intramolecular cyclization following iron reduction for salmycin.^24^ These entry and decoupling properties of natural sideromycins provide important blueprints for the rational design and synthesis of alternative synthetic siderophore–antibiotic conjugates.

For several years, great efforts have been devoted to the synthesis and study of non-natural siderophore–antibiotic conjugates.^6−11^ Despite these tremendous efforts, few examples resulted in favorable antibiotic activity. In particular, synthetic conjugates of siderophores with β-lactam antibiotics have been frequently found to lead to active molecules.^8,25−27^ It should, however, be noted that β-lactam antibiotics target penicillin-binding proteins of the bacterial periplasmic space and are likely active without the need for decoupling, meaning that these molecules possess partial attributes of the natural sideromycins. While it remains challenging, a number of intracellularly active siderophore–antibiotic conjugates have been described based on siderophores such as desferrioxamine,^28,29^ enterobactin,^30−32^ pyochelin,^33^ and pyoverdin^34,35^ (also see refs (6−10)). Research has also investigated the designed introduction of cleavable linkers to allow for intracellular release, including the introduction of cleavable linkers targeted by peptidases or esterases^31^ as well as redox labile linkers, such as disulfide bonds^36^ and the “trimethyl lock”,^29,37^ to mention some. To further facilitate the design of synthetic siderophores, it would be ideal if additional natural siderophore conjugates were found that could provide additional insight into their design.

In this paper, we report the discovery and characterization of a novel natural siderophore–antibiotic conjugate with its biosynthesis encoded by a hybrid biosynthetic gene cluster in *Dactylosporangium fulvum*. Atypically, this siderophore–antibiotic conjugate was found to be generated through a unique iron-mediated conjugation of the individual siderophore and antibiotic units. We then investigate the likely mechanism of this siderophore–antibiotic conjugation and demonstrate that this strategy can be exploited for the construction of other siderophore–antibiotic and siderophore–fluorophore conjugates. Finally, we present data supporting the bacterial uptake of these conjugates, clearly demonstrating that they reach their antibiotic targets in *D. fulvum*. Together, these findings not only enrich our knowledge on siderophore–antibiotic conjugates but also provide an important blueprint to facilitate their future design.

## Results and Discussion

### Discovery of Chlorodactyloferrin

**Figure 1:**
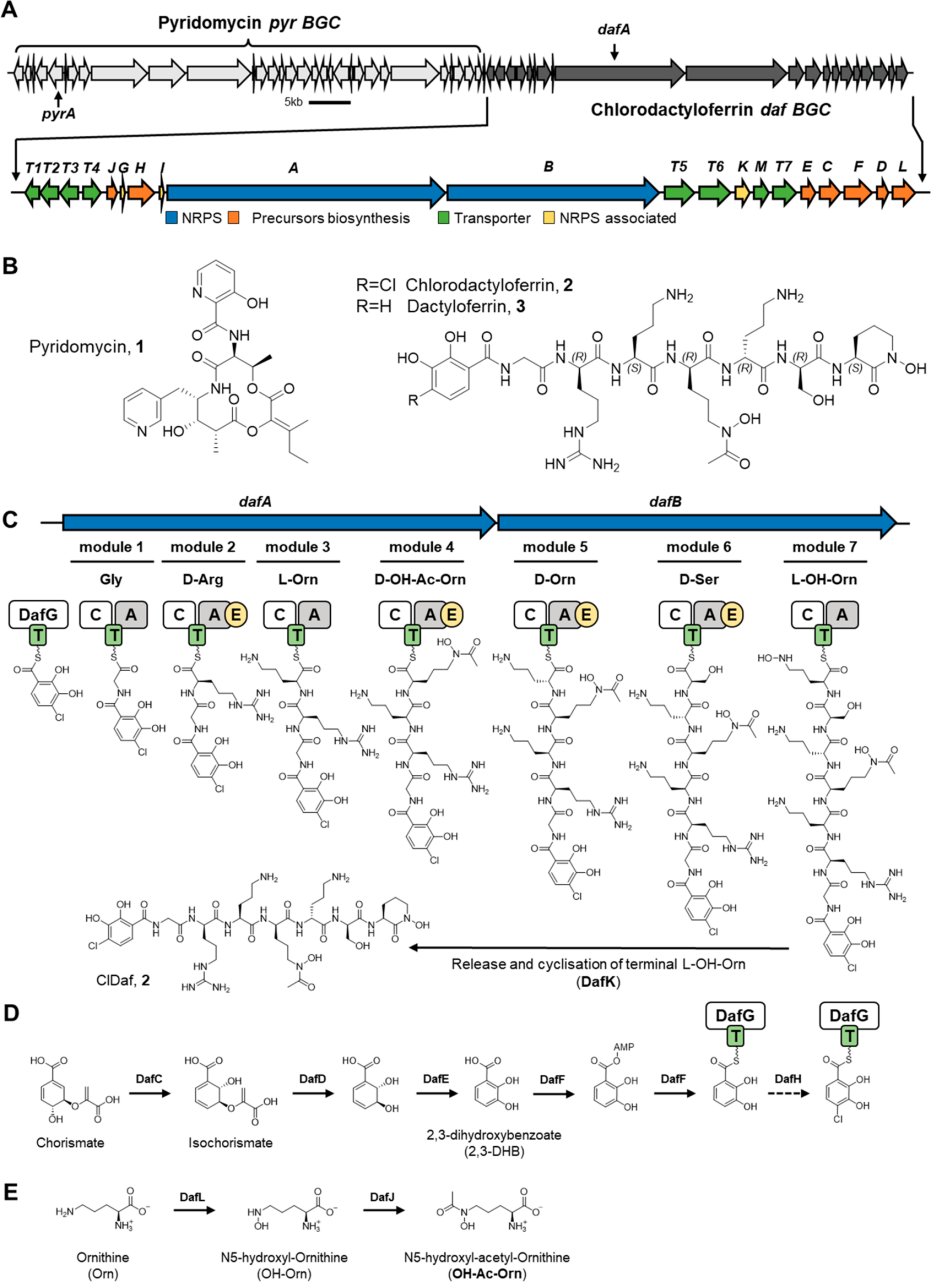
Proposed biosynthesis pathway of ClDaf **2** by*D. fulvum*. (A) Genetic organization of the hybrid Pyr (light gray) and ClDaf (dark gray) BGCs, with a detailed view of the ClDaf BGC shown below with the associated gene function prediction (color) and attributed gene names (with *daf* prefix). (B) Chemical structures of Pyr **1** and ClDaf **2**. The stereochemical assignment of ClDaf **2** is based on the NRPS module organization (presence of epimerase domains) and results from the Marfrey’s analysis shown in Figure S9. (C) Schematic of the predicted sequential biosynthesis of ClDaf **2** by the NRPSs, DafA, and DafB based on the NRPS modules and domain organization (C: Condensation, A: Adenylation, T: Thiolation; E: Epimerization domains), (D) Proposed precursor biosynthesis of 4-chloro-2,3-DHB and (E) modified ornithine precursors by in-cluster gene products.

Whole genome sequencing of *Dactylosporangium fulvum* (accession number CP073720.11) followed by the annotation and classification of biosynthetic gene clusters (BGCs) using AntiSMASH^38^ identified 20 BGCs. One of these BGCs appeared to be structured as a hybrid cluster (Figure 1, Table S1), with half encoding for machinery needed for the production of the antibiotic pyridomycin (Pyr **1**), similar to that in *S. pyridomyceticus,*(39) and the neighboring half for the production of a NRPS-derived halogenated siderophore (hereafter named chlorodactyloferrin, ClDaf, **2**). The presence of a Pyr BGC was unsurprising, as *D. fulvum* is a known producer of this antibiotic that targets bacterial ACP-enoyl reductase of the fatty acid synthesis type II (FASII) system^40−42^ (further characterization of the Pyr BGC is to be presented in a future manuscript). Transcriptome analysis of *D. fulvum* by RNA-Seq found that both halves of this “hybrid” BGC were actively coexpressed (Figure S1). Genetic disruption of the ClDaf BGC through inactivation of the first NRPS gene, *dafA* (*D. fulvum ΔdafA*), did not impact Pyr **1** production but led to the loss of a secondary metabolite, with the mass around [M + H]^+1^ = 1001 and an isotopic profile of a chlorinated molecule (Figure S2). In addition to ClDaf **2**, a minor metabolite with the mass of [M + H]^+1^ = 967 was also no longer produced in *D. fulvum ΔdafA*, a compound later identified as a non-halogenated derivative of ClDaf **2**, named dactyloferrin (Daf **3**). In reciprocal experiments, genetic inactivation of a core pyridomycin biosynthetic gene *pyrA* (*D. fulvum ΔpyrA*) prevented Pyr **1** production, as previously demonstrated in *S. pyridomyceticus,*(39) but did not impact the production of ClDaf **2** or Daf **3** (Figure S2). To confirm that ClDaf **2** had iron binding capacity as a siderophore, UHPLC-MS analysis confirmed that addition of ferric iron to the bacterial supernatant led to the appearance of the expected iron bond complex **2:iron** (Figure S3) **(**HRMS [M + H]^+^: 1054.3707, C_40_H_63_N_14_O_14_ClFe). In addition, purified ClDaf **2** and Daf **3** were both shown to bind iron using the Chrome Azurol S assay (Figure S4). Unlike typical siderophores, the ClDaf **2** BGC was found to be transcribed under iron normal conditions (Figure S1**)**, and this transcription appeared not to be coupled to iron availability, as addition of the iron-chelator 2,2′-dipyridyl (DIP) did not induce upregulation of the ClDaf **2** BGC (Figure S5C), while it did induce another predicted *D. fulvum* siderophore BGC (predicted to produce deferoxamine). In addition, ClDaf **2** production by *D. fulvum* appeared unaffected by iron DIP mediated iron depletion (Figure S5D).

HRMS analysis of purified ClDaf **2** and Daf **3** indicated a mass of [M + H]^+^ = 1001.4590 and 967.4956, corresponding to the predicted formulas of C_40_H_66_N_14_O_14_Cl (1001.4571, 0.9 ppm) and C_40_H_67_N_14_O_14_ (967.4961, −0.5 ppm) respectively. NMR characterization of ClDaf **2** in water confirmed it to be a linear NRPS derived molecule composed of eight amide linked monomers, specifically: 4-chloro-2,3-dihydroxybenzoic acid, glycine, arginine, ornithine, *N-*hydroxyl-*N*-acetyl-ornithine, ornithine, serine, and cyclo-*N*-hydroxy-ornithine (Figure S6, Table S2). Consistent with previous work,^43,44^ NMR analysis of the purified diamagnetic gallium(III) complex of ClDaf, **2:gallium**, in water confirmed the expected metal coordination by the catecholate and by the hydroxamate moieties of the *N*-hydroxyl-*N-*acetyl-ornithine and cyclo-*N*-hydroxy-ornithine units (Figure S7, Table S2). The structural elucidation of Daf **3** by NMR confirmed it to be the non-chlorinated analogue of ClDaf **2** (Figure S8**).** The assigned ^1^H, ^13^C, and ^15^N chemical shifts of **2**, **2:gallium**, and **3** are reported in Table S2.

In line with the eight identified monomers of ClDaf **2**, analysis of the ClDaf BGC showed two large multimodular NRPS proteins (DafA and B), with DafA comprising a starting condensation domain (for the addition of the first monomer) and four modules with adenylation domains, and where DafB included three additional modules with adenylation domains (Figure 1). Four of the DafA/B NRPS modules additionally contain an epimerase domain, indicating that the added monomers are converted to their d-isomer (Figure 1C). The incorporation of d-serine and d-arginine into ClDaf **2** was unequivocally confirmed using the Marfey’s protocol^45^ on the acid hydrolysate of ClDaf **2** against amino acid standards (Figure S9). This analysis also confirmed the incorporation of both d- and l-ornithine-based amino acids into ClDaf **2**, but as acid hydrolysis removes ornithine modifications^46,47^ and because the molecule is predicted to contain both two d- and two l-ornithines, an absolute assignment could not be established by this approach. Nonetheless, in line with this supporting data, it appears reasonable to assume that the structure of ClDaf **2** is consistent with the arrangement of epimerase domains in the ClDaf NRPS modules (Figure 1B). Taken together, the structural and BGC data allow for the proposition of a biosynthesis scheme for ClDaf **2** (Figure 1). Here it was suggested that the 4-chloro-2,3-dihydroxybenzoic acid (Cl-DHB) starting module is synthesized by the halogenation of 2,3-dihydroxybenzoic acid (DHB) by the halogenase DafH (Table S1). The DHB unit itself is expected to be biosynthesized from chorismate by DafC, DafD, DafE, and DafF (Figure 1D, Table S1), that bear high homology to the proteins required for DHB biosynthesis for enterobactin in *E. coli.*(48) Regarding the biosynthesis of non-proteinogenic amino acid precursors of ClDaf **2**, the biosynthesis of modified ornithine precursors is likely mediated by the monooxygenase *dafL* (expected to mediate ornithine *N*-hydroxylation) and the acetyl transferase *dafJ* (expected to mediate ornithine N-acetylation) (Figure 1E). Finally, as the last biosynthetic module lacks a thioesterase, cyclization and release of the C-terminal hydroxyl-ornithine is likely carried out by DafK (a α/β-hydrolase) that is homologous to AmcB of *Amycolatopsis* sp. AA4 described to mediate cyclization of hydroxyl-ornithine for the biosynthesis of amychelin.^49^ In addition to the biosynthetic genes, the ClDaf BGC also encodes seven genes that encode transport systems (Figure 1A, Table S1), where DafT1–4 share high homology with enterobactin uptake transporters FepC, G, D, and B respectively,^50^ likely involved in **2:iron** uptake, and where DafT5/6 (predicted ABC efflux transporters) are likely involved in ClDaf **2** efflux.

### Discovery and Synthesis of a Chlorodactyloferrin–Pyridomycin Conjugate

While ClDaf **2** and Pyr **1** were demonstrated to be biosynthesized independently in *D. fulvum* using the genetic inactivation of their respective BGCs, their proximity in this hybrid BGC invited the investigation of a possible hybrid siderophore-antibiotic molecule, especially in the context of previously described natural sideromycins such as albomycin, salmycin, and ferrimycin.^14−18^ To this end, it was found that when *D. fulvum* was grown in a modified medium containing mannitol as a carbon source, a secondary metabolite was identified with the isotopic profile of a chlorinated molecule and with a mass matching a potential conjugate of ClDaf **2** and Pyr **1** (see MALDI-TOF analysis, Figure S10A), later named ClDaf-Pyr **4.** It was then found that ClDaf-Pyr **4** was not detected in cultures of *D. fulvum* mutants not producing Pyr **1** (*D. fulvum:ΔpyrA*) or ClDaf **2** (*D. fulvum:ΔdafA*) (Figure S10). Rather unexpectedly it was then found that supplementation of *D. fulvum:ΔpyrA* with exogenous pyridomycin (100 μM) complemented the detection of ClDaf-Pyr **4** (MALDI-TOF shown in Figure S11). Together these results supported that the detected molecule consists of both ClDaf **2** and Pyr **1** and that its formation occurs following the synthesis of the individual natural products.

**Figure 2:**
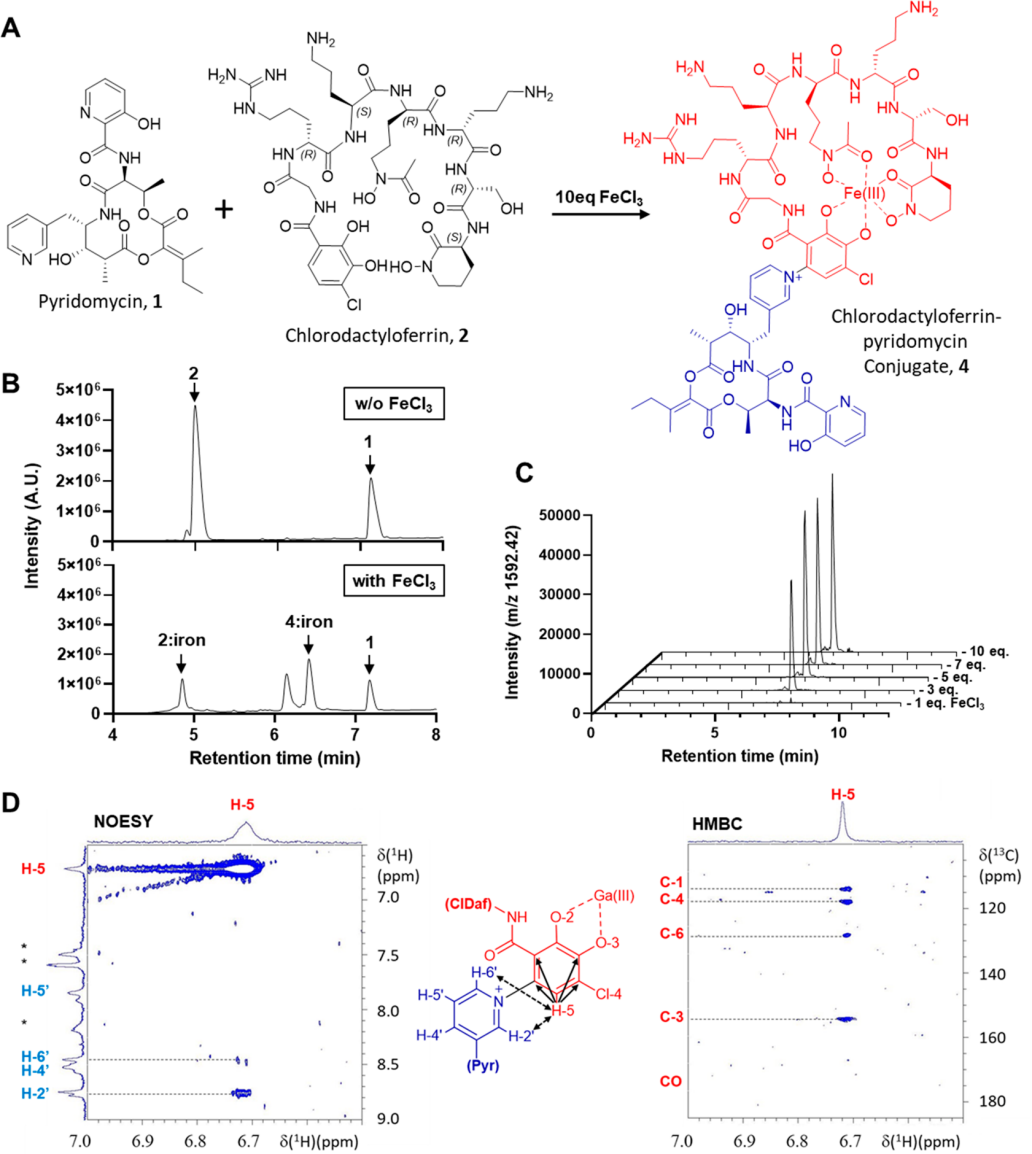
(A) Reaction scheme illustrating the chemical conjugation between ClDaf **2** and Pyr **1** by ferric iron, to generate the ClDaf-Pyr **4** pyridinium product complexed with iron. (B) UHPLC results (UV absorption = 254 nm) of ClDaf **2** and Pyr **1** conjugation in the absence and presence of excess FeCl_3_. (C) UHPLC-MS chromatograms of ClDaf-Pyr **4** iron complex formation (ion call, [M + H]^+^ = 1592.42) in the presence of increasing equivalents of FeCl_3_. (D) NOESY (left) and HMBC (right) NMR data zoomed to show ClDaf **2** – Pyr **1** (Cl-DHB:3-pyridine) attachment (middle structure). 14T NMR spectra were recorded on ClDaf:Ga(III)-Pyr in acetonitrile-*d*_3_/D_2_O (1/1, v/v). 2D ^1^H-NOESY at 293 K shows the spatial proximity between H-5 from the Cl-DHB moiety of ClDaf **2** and H-2′ and H-6′ from the pyridine group of Pyr **1**. The scalar correlations of the single Cl-DHB proton of the conjugate in the 2D ^1^H-^13^C-HMBC at 318 K involve C-4′/C-6′ and C-1′/C-3′ but not CO; only H-5 can give this correlation pattern through 2 and 3 bonds, respectively, after the N-1′ and C-6 bond formation.

To determine if ClDaf-Pyr **4** could be formed from ClDaf **2** and Pyr **1** in the absence of biological material (bacteria/enzymes), we first attempted to mix purified Pyr **1** and ClDaf **2**, but no product was observed by either MALDI-TOF or LC-MS. Taking into account the role of siderophores in chelating ferric iron, and that the latter is a known redox-active metal in a number of biological systems,^51−54^ we then evaluated whether the addition of iron to the mixture of Pyr **1** and ClDaf **2** had any impact. Significantly, this experiment showed that the addition of noncatalytic quantities of ferric iron (FeCl_3_, typically 3–10 equiv, Figure 2B,C) leads to the formation of a number of products, including the corresponding iron-bound **4:iron** ([M + H]^+^ = 1592.7). The formation of noncomplexed ClDaf-Pyr **4** was also observed with CuSO_4_ (10 equiv) but not with ferrous iron (FeSO_4_), CoCl_3_, or GaCl_3_ (all at 10 equiv amounts) (Figure S12**)**. Together these data indicate the notion that only redox-active transition metals acting as oxidation reagents support the formation of ClDaf-Pyr **4.**

### Structural Elucidation of the ClDaf-Pyr **4** Conjugate

The iron complexed form of ClDaf-Pyr, **4:iron**, was shown to have a HRMS of [M + H]^+^ = 1592.5743 (C_67_H_93_N_18_O_22_ClFe, predicted mass of 1592.5763, −0.7 ppm). Mass spectrometry fragmentation of ClDaf-Pyr **4** suggested that the molecules were covalently linked at the chloro-DHB moiety of ClDaf **2** but could not provide further resolution (data not shown). Solution NMR analysis of the purified **4:gallium** complex was carried out to verify the conjugate structure. ^1^H and ^13^C NMR analysis in acetonitrile-*d*_3_/D_2_O (50% v/v) confirmed the detection of the majority of the signals from both Pyr **1** and ClDaf **2** (Table S3). Important differences were, however, observed for the signals corresponding to the chloro-DHB moiety of ClDaf **2**, where only a single aromatic proton in the 6.5–7.0 ppm range was detected, indicating that the other proton is lost to form the covalent bond. ^13^C-HMBC data of this remaining proton had scalar correlations through two and three bonds with C-1, 3, 4, and 6 of the chloro-DHB, but not with the carbonyl (CO), confirming that it corresponds to position 5 (H-5), and provided additional evidence that the covalent bond was formed with C-6 (Figure 2D). NOESY experiments confirmed dipolar correlations of the chloro-DHB H-5 with the H-2’ and H-6’ protons of the Pyr **1** pyridine moiety (Figure 2D). Together these data pointed to ClDaf-Pyr **4** being a conjugate bound through a C–N bond of the ClDaf **2** chloro-DHB C-6 and the 3-pyridine nitrogen of Pyr **1**, leaving a positively charged pyridinium product. Further support for this C6–N linkage was obtained using structural analysis of model conjugates (**18a** and **18b**) as described further below.

### Identification of the Catechol–Pyridine Conjugation Mechanism

The catechol–pyridinium linkage found in the structure of ClDaf-Pyr **4** represents a rare molecular feature, with a similar bond previously only identified in the natural siderophore chryseomonin. The retrosynthesis of chryseomonin from chrysobactin by Adolphs and colleagues^55^ was achieved using chemistry described by Saxena et al.,^56^ where pyridine was linked to the catechol moiety of chrysobactin using molecular iodine as an oxidant. Neiland and co-workers^57^ then showed that the mechanism of this conjugation involved a formal oxidation of the catechol to its quinone form, followed by the Michael-type addition of the nucleophilic pyridine nitrogen on the C-6 electrophilic carbon of the quinone. Several publications have appeared in recent years documenting the same mechanism with various other nucleophiles.^58^ In parallel, ferric iron is a known oxidation agent of catechols, which can mediate the concomitant formation of semiquinone radicals, and ultimately quinones when excess ferric iron is provided.^58,59^ Therefore, it seemed plausible that the newly identified conjugation strategy based on the use of iron(III) also proceeded through a Michael-type addition reaction. In our attempt to validate this prediction, a number of experiments were conducted and cross-referenced for the final results.

**Figure 3:**
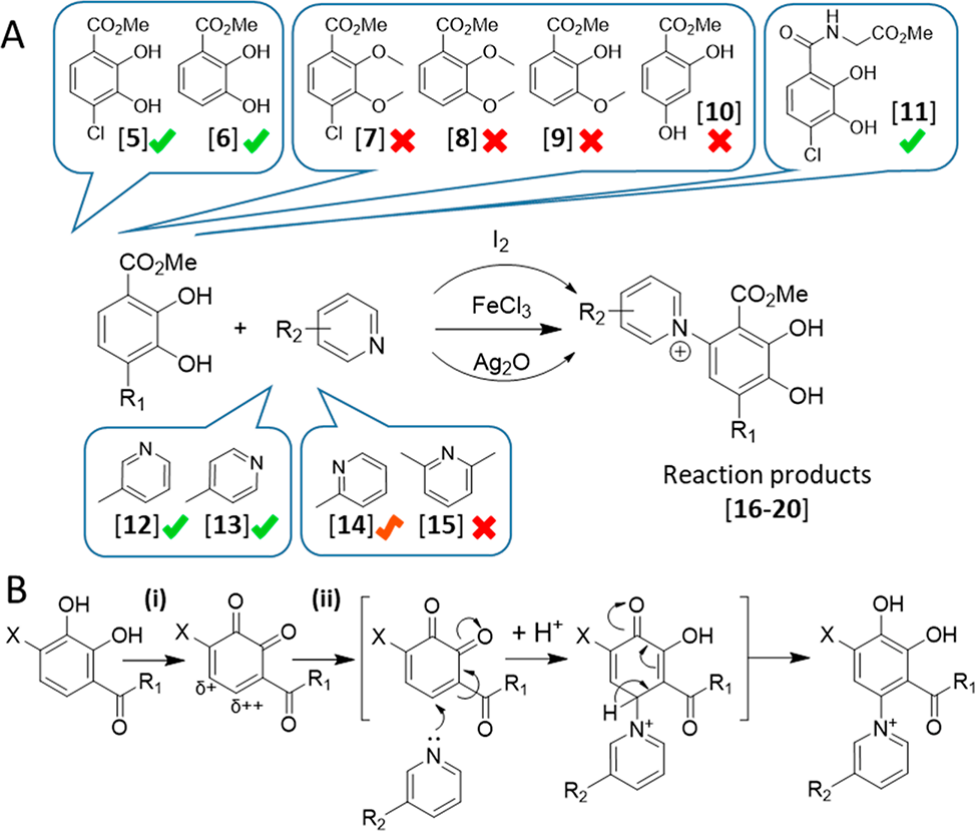
(A) Summary of the results of model reactions with iron(III), molecular iodine, and silver oxide. All three oxidants allowed for the conjugation of catechols **5** and **6** with pyridines **12**–**14** (green tick). No reactivity was observed with derivatives **7**–**10** (indicated with red-cross). Similar conjugation efficiency was observed with all three oxidants. The sterically hindered **14** and **15** gave poor and no resulting conjugate, respectively. (B) Proposed mechanism: (i) oxidation of the catechol moiety to its electrophilic quinone form, and (ii) Michael-type addition reaction by the pyridine nitrogen on the most electrophilic C-6 carbon.

To test the reaction mechanism, several experiments were performed using model reactants that would mimic the iron-based formation of ClDaf-Pyr **4.** For this goal, 4-chloro-2,3-dihydroxybenzoate methyl ester **5** and 3-methylpyridine **12** (Figure 3A) were selected as structurally related surrogates of ClDaf **2** and Pyr **1,** respectively. For the model substrates, addition of excess amounts of iron trichloride (10 equiv), molecular iodine (1 equiv), and silver oxide resulted in the formation of product **16**, with identical regiospecific pyridinium coupling of ClDaf-Pyr **4,** as confirmed by NMR NOESY/HMBC correlations (Figure S13). In a similar manner, these three oxidants also allowed conjugation of the non-chlorinated catechol methyl esters **6** with pyridine derivatives (Table S4). When the selected catechol methyl ester mimics were redox inactive, such as compounds **7**–**10**, neither FeCl_3_, I_2_, nor Ag_2_O were able to mediate their coupling with the pyridines (Table S4). In addition, the oxidation of model compound **11** was also tested by electrochemistry (in a nondivided cell) and found to also allow for the formation of the identical region-specific conjugate **20** (Figure S14) as produced using FeCl_3_, I_2_, and Ag_2_O. With respect to the reaction efficiency, FeCl_3_, I_2_, and Ag_2_O all allowed for bonding the catechol moiety with 3- and 4-methylpyridines **12** and **13**, while lower yields were observed with 2-methylpyridines **14**, and no product observed with 2,6-dimethylpyridine **15**. Lower yields experienced with compound **14** and lack of reactivity with **15** can be rationally explained based on obstructed access to the nitrogen atom in these substrates. The fact that identical products formed with a variety of oxidants points out the fact that the Fe(III)-mediated conjugation follows the same mechanistic pathway. In a way, the Fe-mediated coupling most closely resembles the one described for I_2_, even though in both cases the formation of the respective quinone could not be directly confirmed in either case. It therefore appears that in the presence of an excess amount of FeCl_3_ the reaction equilibrium is pushed to catechol oxidation with the transient formation of the highly reactive quinone, after which the pyridine nitrogen acts as a nucleophile by attacking the most electrophilic C-6 of the putative quinone in a Michael-type addition reaction (Figure 3B).

### Additional Evidence for Specific C6-N Chlorocatechol-Pyridinium Conjugation Product

To gain additional evidence on the C6-N linkage between the chlorocatechol and pyridine moieties of the generated pyridinium conjugates, we decided to conduct further investigation on the model conjugate **18**. As described above, the synthesis of compound **18** was achieved either using the iron(III) trichloride method to produce **18a** (in which the complexed iron was replaced with gallium) or through the iodine-mediated method to give compound **18b**. It was postulated that dechlorination of the **18** conjugate would introduce an additional aromatic hydrogen into the catechol moiety of the conjugate to allow for the facile distinction between two adjacent aromatic doublets with a large ^3^*J*-coupling constant (in the case of a dechlorinated C6-N conjugate) or with a small ^4^*J*-coupling constant (in the case of an alternative dechlorinated C5-N conjugate) (Figure S15a). To achieve this dechlorination, reductive hydrogenation of **18** over a palladium catalyst was used,^7,60^ which also mediated the reduction of the pyridinium salts to the corresponding piperidine derivatives (in line with refs (61−63)) (Figure S15a). Using this method, **18b** (generated using iodine) was dechlorinated to the catechol-methylpiperidine product (**18f**) with the proton NMR of **18f** revealing two aromatic doublets with large ^3^*J*-coupling constants (^3^*J* = 8.5 Hz) at 6.71 and 6.87 ppm, which, together with other NMR data (including 2D ^1^H-^13^C and ^1^H-^15^N HMBC, Figure S16) further consolidated that the C-N conjugation occurs specifically at C6 of the catechol. When this procedure was applied to the iron generated **18a**, the respective dechlorinated product **18e** was obtained, with the corresponding proton NMR analysis mirroring those of **18f** (Figure S15b/c) and confirming that the conjugation products were identical. Collectively, these data validate that the chlorocatechol–pyridine linkage occurs between C6 of the catechol and the pyridine nitrogen atom. Unfortunately we were not able to extend this approach to ClDaf-Pyr:Ga **4:Gallium**, as its hydrogenation led to uncontrolled degradation of the conjugate.

### Synthesis and Evaluation of Biomimetic Chlorodactyloferrin Fluorophore Conjugates

**Figure 4:**
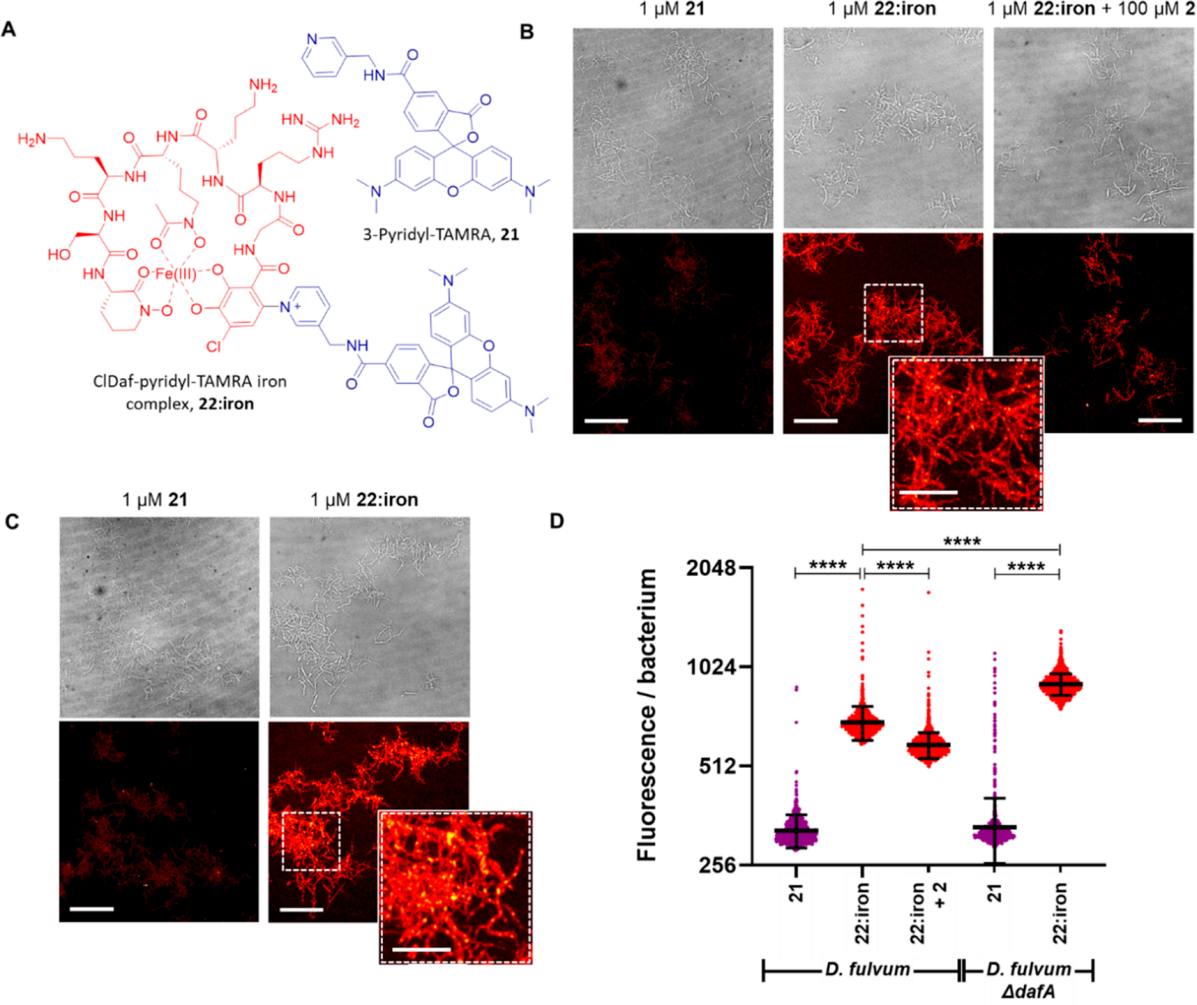
(A) Chemical structures of 3-pyridyl-TAMRA **21** and the ClDaf-3-pyridyl-TAMRA **22:iron** conjugate. (B) Representative phase contrast (top) and fluorescence (bottom) confocal images of wild-type *D. fulvum* incubated for 4 h in the presence of 3-pyridyl-TAMRA **21** (left images), ClDaf-3-pyridyl-TAMRA **22:iron** (middle images), and ClDaf-3-pyridyl-TAMRA **22:iron** with supplemented nonlabeled ClDaf **2** (right images). (C) Representative phase contrast (top) and fluorescence (bottom) confocal images of *D. fulvum ΔdafA* grown for 4 h in the presence of 3-pyridyl-TAMRA **21** (left images) and ClDaf-3-pyridyl-TAMRA **22:iron** (right images). Scale bar represents 20 μm for the full view and 10 μm for the local view. (D) Quantification of the fluorescence intensity per bacterium in conditions from panels B and C, showing significantly improved uptake of ClDaf-3-pyridyl-TAMRA **22:iron** as compared to nonconjugated 3-pyridyl TAMRA **21**, which was partially suppressed by addition of nonfluorescent ClDaf **2**. Image also shows an increased uptake of ClDaf-3-pyridyl-TAMRA **22:iron** in *D. fulvum* unable to synthesize ClDaf **2** by itself. Black lines and errors indicate mean ± SD. Asterisks denote significance by Kruskal–Wallis and Dunn’s multiple comparison test: ****P < 0.0001. The data shown are from at least three independent images (*n* > 200 cells per condition).

To evaluate the biological “Trojan horse” potential of siderophore conjugates generated using the here uncovered catechol–pyridine conjugation, it was decided to commence with the attachment of a fluorescent cargo molecule to ClDaf **2**. To install the pyridine functionality, 3-pyridyl-tetramethylrhodamine (3-pyridyl-TAMRA **21**) was synthesized from 5-carboxytetramethylrhodamine and 3-picolylamine. 3-Pyridyl-TAMRA **21** was then conjugated to ClDaf **2** in the presence of 10 equiv of FeCl_3_, allowing for the successful formation of the iron bound ClDaf-3-pyridyl-TAMRA conjugate 22 (Figure 4A). Following metal exchange with gallium(III), NMR analysis of the purified product confirmed identical regioselective conjugation between the ClDaf catechol and the pyridine of 3-pyridyl-TAMRA **21** as seen with ClDaf-Pyr **4** (Figures S17, S18).

To identify whether the ClDaf-3-pyridyl-TAMRA **22** conjugate could be taken up by bacteria, confocal microscopy on *D. fulvum* was used to follow the compound uptake. The results showed that in the presence of ClDaf-3-pyridyl-TAMRA **22** bacteria readily became fluorescent (excitation and emission wavelengths of 561 and 595 nm, respectively), while this effect was less pronounced in the presence of equimolar concentrations of the nonconjugated 3-pyridyl-TAMRA **21** (Figure 4B). To support that the fluorescent conjugate was actively taken up by *D. fulvum,* uptake experiments were repeated in the presence of supplemented nonlabeled ClDaf **2** (to act as a competitor), and the corresponding fluorescence uptake was found to be significantly decreased (Figure 4B). As an additional approach, the non-ClDaf producing *D. fulvum ΔdafA* mutant was found to allow for greater fluorescence accumulation of ClDaf-3-pyridyl-TAMRA **22** (Figure 4C). DIP mediated iron limitation was found not to greatly impact ClDaf-3-pyridyl-TAMRA **22 (**Figure S19**)**, in line with the before described constitutive and non-iron regulated expression of the ClDaf BGC (Figure S5). Finally, uptake of ClDaf-3-pyridyl-TAMRA **22** and 3-pyridyl-TAMRA **21** was also evaluated in other bacterial species, with data revealing ClDaf-3-pyridyl-TAMRA **22** uptake by all evaluated *Dactylosporangium* sp. and *Streptomyces coelicolor,* while no significant uptake of either fluorophore was observed for *S. aureus* and *P. aeruginosa* (Figure S20). Together these data support the hypothesis that ClDaf-3-pyridyl-TAMRA **22** can be actively taken up as a “Trojan horse fluorophore” by a number of *D. fulvum* and other closely related bacteria.

### Biomimetic Conjugation of Chlorodactyloferrin with Pyridine-Based Antibiotics

**Figure 5:**
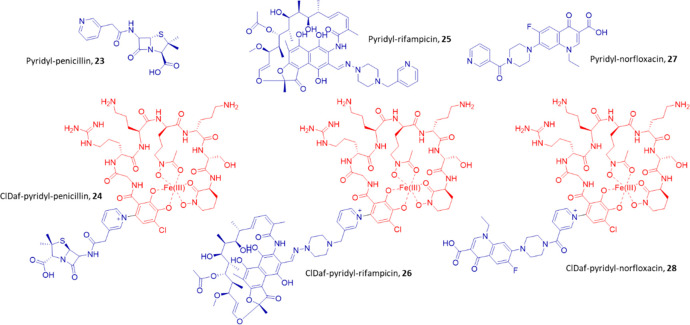
Chemical structures of the pyridyl-modified antibiotics **23**, **25**, and **27**, and their respective ClDaf-conjugates **24**, **26**, and **28**.

Following evidence for the uptake of fluorescent ClDaf-3-pyridyl-TAMRA **22** conjugates, it was questioned whether biomimetic conjugates could allow for antibiotic uptake and subsequent antibiotic activity. Initial studies with ClDaf-Pyr **4** (up to 50 μM) found it to be inactive against other *Dactylosporangium* strains, though neither of these bacteria were susceptible to Pyr **1**. To elucidate if antibacterial activity could be attained, it was thus decided to evaluate the potential of coupling other antibiotics to ClDaf **2**. With *D. fulvum* being a Gram-positive bacterium (without an outer cell membrane), a β-lactam antibiotic was first selected (**23**) to probe siderophore–antibiotic conjugate penetration across the well wall to the peptidoglycan layer (where the target penicillin-binding proteins are located) without having to cross a membrane. Second, a rifamycin (**25**) was selected, as it targets the cytosol-located DNA dependent RNA polymerase (RNAP) and thus requires translocation across the bacterial membrane. To allow for biomimetic conjugation to ClDaf **2**, 3-pyridyl moieties were introduced into these antibiotics to give 3-pyridyl-penicillin **23**, a penicillin G analogue where the 3-pyridine group replaced the phenyl group, and 3-pyridyl-rifampicin **25**, a rifampicin analogue with a 3-pyridine extension on the rifampicin piperazine fragment (Figure 5). Both 3-pyridyl-penicillin **23** and 3-pyridyl-rifampicin **25** maintained antibiotic activity on *D. fulvum* (Table 1). In addition, a 3-pyridyl-containing fluoroquinolone (3-pyridyl-norfloxacin, **27**) was commercially obtained, but it failed to show appreciable antibiotic activity against *D. fulvum*.

**Table 1:** Bacterial Antibiotic Susceptibility in Liquid Culture to 3-Pyridyl-Penicillin **23**, 3-Pyridyl Rifampicin **25**, and Their Respective ClDaf **2** Conjugates **24** and **26**^a^

	**Minimal Inhibitory Concentration, μM(mg/L)**			
**Bacterial strain**	3-pyridyl-penicillin **23**	ClDaf-3-pyridyl-penicillin conjugate **24:iron**	3-pyridyl-rifampicin **25**	ClDaf-3-pyridyl-rifampicin conjugate **26:iron**
***D. fulvum*WT**	0.62 (0.26)	2.5 (3.5)	2.5 (2.25)	10 (19.5)
***D. fulvum ΔdafA***	1.25 (0.53)	2.5 (3.5)	5 (4.5)	10 (19.5)
***D. fulvum rpoB*H418R**	0.62 (0.26)	ND	>100 (>90)	>100 (>195)
***D. aurantiacum***	1.25 (0.53)	5 (6.9)	5 (4.5)	10 (19.5)
***D. matsuzakiense***	5 (2.13)	10 (13.9)	5 (4.5)	10 (19.5)
***D. roseum***	5 (2.13)	10 (13.9)	5 (4.5)	20 (39)
***D. vinaceum***	1.25 (0.53)	5 (6.9)	2.5 (2.25)	5 (9.75)
***S. coelicolor***	>50 (>21.3)	>20 (>27.7)	>50 (>45)	>20 (>39)
***S. aureus***	2.5 (1.1)	>20 (>27.7)	0.002 (0.0018)	0.78 (1.52)
***E. coli***	>50 (>21.3)	>20 (>27.7)	2.5 (2.25)	>20 (>39)
***P. aeruginosa***	>50 (>21.3)	>20 (>27.7)	>20 (>18)	>20 (>39)

a MICs were determined in GYM supplemented with 100 μM DIP for *Dactylosporangium* strains and *S. coelicolor*, while in CAMHB supplemented with 300 μM DIP for *E. coli*, *P. aeruginosa*, and *S. aureus*. Bacterial viability was determined using resazurin reduction, and data are an average of three biological replicates in μM (mg/L).

Once synthesized, 3-pyridyl-penicillin **23** and 3-pyridyl-rifampicin **25** were conjugated with ClDaf **2** using the ferric iron approach, to give the expected ClDaf-3-pyridyl-penicillin **24** and ClDaf-3-pyridyl-rifampicin **26** conjugates, respectively (Figure 5). The structures of both conjugates were confirmed by HRMS and NMR analysis to be as previously seen for ClDaf-Pyr **4 (**Figures S17, S18). As a note, HRMS confirmed the mass and expected formula of ClDaf-3-pyridyl penicillin **24**; its low stability in D_2_O resulted in temporal hydrolysis of the **24,** which was confirmed by the corresponding NMR analysis. Even if 3-pyridyl norfloxacin **27** was inactive against *D. fulvum*, ClDaf-3-pyridyl-norfloxacin **28** was prepared to demonstrate the translatability of the iron-based protocol (Figure 5).

The antibiotic activities of the 3-pyridyl antibiotics **23** and **25** and their ClDaf conjugates **24** and **26** were next evaluated in the dose response against *D. fulvum* using liquid culture-based assays. These studies showed *D. fulvum* to be susceptible to both 3-pyridyl-penicillin **23** (MIC = 0.6 μM) and 3-pyridyl-rifampicin **25** (MIC = 2.5 μM). Interestingly, ClDaf-3-pyridyl-penicillin **24** was found to retain antibiotic activity against *D. fulvum* (MIC = 2.5 μM) (Table 1), suggesting that this conjugate was able to cross the bacterial cell wall to the peptidoglycan layer and inhibit the target penicillin-binding proteins. As mentioned above, access to the peptidoglycan layer in Gram-positive bacteria as *D. fulvum* is not restricted by an outer cell membrane, and the activity of ClDaf-3-pyridyl-penicillin **24** therefore does not necessitate active uptake. Additional work demonstrated that other *Dactylosporangium* species that were shown to take up ClDaf-3-pyridyl-TAMRA **22** (Figure S20) were also susceptible to ClDaf-3-pyridyl-penicillin **24** (Table 1). Finally, iron depletion of the medium with DIP was found not to increase *D. fulvum* susceptibility to ClDaf-3-pyridyl-penicillin **24** (Table S5).

ClDaf-3-pyridyl-rifampicin **26** was encouragingly found to elicit antibiotic activity against *D. fulvum* (MIC = 10 μM, also observed in other *Dactylosporangium* species, Table 1), though it was slightly less active compared to its parent compound, 3-pyridyl-rifampicin **26** (Table 1). To confirm that this conjugate was acting through the inhibition of the DNA dependent RNA polymerase, a rifampicin resistant *D. fulvum* mutant was isolated and characterized to carry a known rifampicin conferring mutation in the RpoB subunit of RNAP, namely H418R.^64^ This rifampicin resistant *D. fulvum* strain (rpoB-H418R) was confirmed to be cross-resistant to 3-pyridyl-rifampicin **25** but also to ClDaf-3-pyridyl-rifampicin **26** (Table 1), confirming the activity of the siderophore conjugate on the RNAP in *D. fulvum*. Iron depletion of the media by DIP was found not to increase bacterial susceptibility to ClDaf-3-pyridyl-rifampicin **26** (Table S5), in accordance with the constitutive expression of the ClDaf BGC (Figure S5) and minimal impact of DIP on ClDaf-3-pyridyl-TAMRA **22:iron** uptake (Figure S19). Using analytical UHPLC-MS, ClDaf-3-pyridyl-rifampicin **26:iron** was found to be stable in *D. fulvum* culture filtrate (spent media), with more than 90% recovery measured following 2 days of incubation (Figure S21) and without detectable release of 3-pyridyl-rifampicin (similar results were observed with incubation in fresh GYM media), suggesting that no conjugate decoupling occurred in the bacterial culture filtrate. These data were in line with the highly rifamycin-susceptible bacterium, *S. aureus,* being 400 fold less susceptible to ClDaf-3-pyridyl-rifampicin **26** compared to 3-pyridyl-rifampicin **25**. Together these data suggest entry of ClDaf-3-pyridyl-rifampicin **26** into the *D. fulvum* cytosol.

RNAP inhibition by ClDaf-3-pyridyl-rifampicin **26** may occur directly by the antibiotic conjugate itself or alternatively may require an intracellular decoupling process for rifamycin release and subsequent RNAP inhibition. To this end, *in vitro* biochemical inhibition studies were performed to determine direct inhibition of these compounds on commercially available *E. coli* and *S. aureus* RNAP assays. As expected, both rifampicin and 3-pyridyl-rifampicin **25** showed potent inhibition of both RNA-polymerases (Table 2). However, despite the attachment of a voluminous iron-bound ClDaf **2** moiety, ClDaf-3-pyridyl-rifampicin **26** was found to retain substantial RNAP inhibition activity for both bacterial RNAPs (Table 2). While this was unexpected, other rifampicin conjugates with large additions to the rifampicin piperazine moiety, such as GE23077^65^ and TNP-2092,^66^ have also been reported to inhibit the enzyme. This data therefore does not allow us to clearly establish whether ClDaf conjugates are processed intracellularly by bacteria to induce cargo decoupling, and this will be the subject of a separate investigation.

**Table 2:** Activity of Rifampicin, 3-Pyridyl-rifampicin **25**, and Its ClDaf-Conjugate **26** on the RNA Polymerases of *E. coli* and *S. aureus*^a^

	**IC**_**50**_**(nM)**		
**RNA polymerase**	Rifampicin	3-pyridyl-rifampicin **25**	ClDaf-3-pyridyl-rifampicin, **26:iron**
***E. coli***	45.5 ± 5.0	136.6 ± 29.8	1122 ± 370
***S. aureus***	15.3 ± 3.8	151.0 ± 23.6	101.5 ± 19.7

a IC_50_ values (i.e. concentration of compound at which the enzyme activity is inhibited by 50%) are presented as a mean ± SD (in nM) of four independent biological replicates.

## Discussion

Few natural sideromycins have been discovered thus far, but they have served as invaluable blueprints for the rational design of custom siderophores–antibiotic conjugates. These natural systems have provided crucial insight into how nature has selected conjugates that hijack siderophore uptake systems for bacterial entry and antibiotic activity. Unfortunately, the restricted number of natural siderophore–antibiotic conjugates has hindered the capacity to copy and translate their naturally selected chemical properties for the synthesis of custom siderophore–antibiotic conjugates. Moreover, these natural molecules are constituted by antibiotics coupled to hydroxamate-based siderophores, such as ferrichrome, ferrioxamine, and danoxamine, and therefore, they provide limited insight into how antibiotics can be coupled on to catecholate-containing siderophores. This is particularly important because antibiotic penetration into Gram-negative bacteria is a major hurdle, and many of these bacteria use catechol-containing siderophores (such as enterobactin,^67^ acinetobactin,^68^ and fimsbactin^69^) for iron assimilation. To this extent, the discovery and characterization of the ClDaf-pyr **4** conjugate presents a novel insight into the design of siderophore–antibiotic conjugates using catechol-containing siderophores.

Among the known natural conjugates of siderophores and antibiotics, ClDaf-Pyr **4** is atypical in that it can be prepared by chemically combining the individual antibiotic and siderophore entities. As an example, both albomycin^70,71^ and microcin E492m^21^ are biosynthesized through enzymatic conjugation of their siderophore and bioactive antibiotic/peptide parts. In biological systems, ubiquitous enzymes such as horseradish peroxidases and polyphenol oxidases readily mediate catechol oxidation,^58^ and they could potentially enable the activation step needed for the conjugation of ClDaf and Pyr. To this extent, we cannot exclude that enzymes in *D. fulvum* are able to facilitate the formation of the ClDaf-Pyr conjugate, though no such in-cluster candidates have been identified in *D. fulvum*. Nevertheless the finding that ferric iron is not only chelated by ClDaf **2** but also has the potential to oxidize the siderophore catechol group when added in excess (also previously described^72−75^) is intriguing and suggests that such conjugates can potentially be formed in environments rich in ferric iron. Interestingly, the critical role of environmental iron was previously demonstrated for catechol-based reactions such as for mussel adhesion.^76,77^

The ability to conjugate a catechol-containing siderophore with a pyridine-containing antibiotic in a biomimetic manner offers a number of potential advantages. First, catecholate-containing siderophores are commonly used for iron assimilation by bacteria, but particularly by Gram-negative bacteria for which antibiotic drug discovery is the most critical, such as *Enterobacterales*, *Acinetobacter baumannii,*(2) known to synthesize and use catechol-containing enterobactin,^67^ acinetobactin,^68^ and fimsbactin^69^. Second, conjugates can be generated without modifications of the natural catechol-containing siderophores. Third, the nonclassical coupling approach has the benefit of forming a conjugate where the resultant siderophore catechol group will be in its oxidation resistant form and thus able to continue binding iron as a siderophore. This attractive property is achieved due to the electron withdrawing nature of the positively charged pyridinium nitrogen, and such a molecular feature is difficult to attain with classical nucleophiles such as amines and thiols, where the resultant conjugates would form in their oxidized quinone form unable to bind iron.

The biological studies presented here go a long way toward characterizing the uptake of catechol-pyridine-based siderophore antibiotic conjugates. Uptake studies with conjugated fluorophores (ClDaf-3-pyridyl-TAMRA **22**) provide evidence that this conjugate is taken up preferentially over the unconjugated 3-pyridyl-TAMRA **21** and that this uptake can be decreased through competition with unconjugated ClDaf **2** or increased by genetically preventing the bacteria from producing competing ClDaf **2**. Similarly, the conjugated antibiotics were found to have antibiotic activity suggesting that they reached their respective targets. Siderophore transporters are often encoded by in cluster genes (*fepC,G,D,B* for the ClDaf BGC), and multiple attempts were made to link these transporters with the uptake of the ClDaf conjugates. Unfortunately, using routine genetic approaches that commonly work for *D. fulvum* (also used to inactivate *dafA* and *pyrA* in this work), to disrupt *dafT3* or *dafT4* was not successful, neither by single cross over inactivation (only the clones obtained had genomic duplication of the gene locus, with a maintained WT copy) nor by double cross over gene removal (only single cross over achieved). The reason for this inability to knockout the transporters suggests that they play a critical role in bacterial viability or may be related to the high local GC content (75–80%). Further work is therefore required to pinpoint the exact mechanism of ClDaf-cargo uptake.

In addition to the uptake of ClDaf conjugates, it was of interest to find out whether an intracellular decoupling mechanism existed to allow for antibiotic release. The design of ClDaf-3-pyridyl-rifampicin **27** was intended to help answer this question, as it was considered unlikely that such a conjugate would be able to effectively enter and inhibit the bacterial RNAP. While susceptibility data confirmed ClDaf-3-pyridyl-rifampicin **27** to elicit its antibiotic activity through inhibition of the RNAP (cross resistance), biochemical data showed that this inhibition could occur directly, and that decoupling was not required. At this point, it is difficult to unambiguously establish the existence of an intracellular decoupling mechanism, and further work with other conjugates is needed to address this question.

Together, this work has brought to light a novel “natural” mechanism by which catechol-containing siderophores can be coupled to antibiotics through a previously unexploited manner. This mechanism of this siderophore-antibiotic coupling was shown not to be restricted to ClDaf-Pyr, and could be translate to other pyridine-based bioactive or fluorescent molecules. Experiments with the model substrates suggest that such conjugation can also be achieved with other catechol-containing siderophores, as the main requirement is the need for a catechol group. Further work is now in progress with the focus on expanding this research to target pathogenic bacteria for which antibiotic R&D is an ongoing priority.

## Supporting Information Available

Experimental procedures (materials and methods) as well as supplemental tables (Tables S1–S2) and figures (Figures S1–S21) (PDF)
Supplemental synthetic and analytical chemistry procedures (PDF)
Supplemental HRMS and NMR spectra (PDF)
