# Supplementary material for: A Novel Natural Siderophore Antibiotic Conjugate Reveals a Chemical Approach to Macromolecule Coupling
Source: ACS Cent Sci. 2023 Nov 10;9(11):2138–49. doi: 10.1021/acscentsci.3c00965 (PMC10683483; doi:10.1021/acscentsci.3c00965)
Supplement: Supplementary file 1 — oc3c00965_si_001.pdf [file oc3c00965_si_001.pdf]

Supplementary data

Biological Materials and Methods, Supplemental Tables and Figures

A novel natural siderophore antibiotic conjugate reveals a chemical approach to macromolecule coupling

Thibault CARADEC[1+], Ernesto ANOZ-CARBONELL[1+], Ravil PETROV[1+], Muriel BILLAMBOZ[2,3], Kevin ANTRAYGUES[4], Francois-Xavier CANTRELLE[2,5], Emmanuelle BOLL[2,5], Delphine BEURY[6], David HOT[6], Herve DROBECQ[1], Xavier TRIVELLI[7], Ruben C. HARTKOORN[1]*

[+] Joint first authorship (contributed equally)
** Corresponding Author, Ruben C. Hartkoorn: Email: ruben.hartkoorn@inserm.fr*

[1] Univ. Lille, CNRS, Inserm, CHU Lille, Institut Pasteur Lille, U1019 - UMR 9017 - CIIL - Center for Infection and Immunity of Lille, F-59000 Lille, France.
[2] Univ. Lille, Inserm, CHU Lille, Institut Pasteur de Lille, U1167 - RID-AGE - Risk Factors and Molecular Determinants of Aging-Related Diseases, F-59000 Lille, France.
[3] JUNIA, Health and Environment, Laboratory of Sustainable Chemistry and Health, Lille, F-59000, France
[4] Univ. Lille, Inserm, Institut Pasteur de Lille, U1177 - Drugs and Molecules for Living Systems, F-59000, Lille, France.
[5] CNRS, EMR9002 BSI Integrative Structural Biology, 59000 Lille,
[6] Univ. Lille, CNRS, Inserm, CHU Lille, Institut Pasteur de Lille, UMR2014 - US41 - PLBS-Plateformes Lilloises de Biologie & Santé, F-59000, Lille, France.
[7] Univ. Lille, CNRS, INRAE, Centrale Lille, Univ. Artois, FR 2638 - IMEC - Institut Michel-Eugène Chevreul, 59000, Lille, France.

Table of Contents

Experimental Procedures S4

Bacterial strains and growth conditions S4

D. fulvum culture for secondary metabolite formation S4

General molecular biology techniques used S4

Construction of *D.fulvum ΔpyrA* S4

Construction of *D.fulvum ΔdafA* S4

RNA extraction and RT-qPCR S4

Confocal fluorescence microscopy S5

Antibacterial activity S5

Rifamycin resistant mutant selection S5

RNA polymerase assay S6

MALDI-TOF analysis of secondary metabolites S6

Analytical mass spectrometry (UHPLC-MS) S6

High-resolution Mass spectrometry (HRMS) S6

Preparative HPLC purification S6

Production and purification of pyridomycin, 1, from *D. fulvum* S6

Production and purification of chlorodactyloferrin, 2, and dactyloferrin, 3, from *D. fulvum* S6

NMR S7

Supplementary Tables S8

Table S1: S8

Table S2: S9

Table S3: S10

Table S4: S11

Table S5. .... S12

Table S6: S12

Supplementary Figures S13

Figure S1: S13

Figure S2. .... S14

Figure S3. .... S15

Figure S4. .... S15

Figure S5. .... S16

Figure S6: S17

Figure S7: S18

Figure S8: S19

Figure S9: S20

Figure S10. .... S21

Figure S11. .... S22

Figure S12. .... S23

Figure S13. .... S24

Figure S14. .... S25

Figure S15 .... S26

Figure S16 .... S27

Figure S17: S28

Figure S18: S29

Figure S19: S30

Figure S20. .... S31
Figure S21. .... S32
References: S33

Experimental Procedures

Bacterial strains and growth conditions

Dactylosporangium fulvum NRRL B-16292, *Dactylosporangium vinaceum* NRRL B-16297, *Dactylosporangium matsuzakiense* NRRL B-16293, *Dactylosporangium aurantiacum* NRRL B-8018 and *Dactylosporangium roseum* NRRL B-16295 were obtained from the ARS Culture Collection (NRRL). *Streptomyces coelicolor* strain A3(2) was kindly provided by Juan-Pablo Gomez-Escribano of the John Innes Centre (StrepStrain) (UK). *Staphylococcus aureus* strain SH100 was kindly provided by Simon J Foster from the University of Sheffield (UK). *Pseudomonas aeruginosa* strain POA1 was obtained from the Belgian Coordinated Collection of Microorganism (BCCM: LMG 1025).

Dactylosporangium strains and *S. coelicolor* were routinely grown in GYM/ISP2 medium (4 g/L glucose, 4 g/L yeast extract, 10 g/L malt extract, pH 7.2) or MYM medium (4 g/L mannitol 4 g/l, 4 g/L yeast extract, 10 g/L malt extract, pH 7.2) at 28 °C at 250 rpm. *B. subtilis, S. aureus and P. aeruginosa* were grown in cation-adjusted Mueller-Hinton broth (CAMHB; BD Difco, Franklin Lakes, New Jersey, USA) at 37°C at 180 rpm. When indicated, the iron chelator 2,2'-dipyridyl (DIP) (Sigma-Aldrich) was added to the culture media to generate iron-restrictive conditions. For bacterial culture on solid media, same media were used with 1.5% agar (BD Difco).

D. fulvum culture for secondary metabolite formation

For the production of pyridomycin [1], chlorodactyloferrin [2] or dactyloferrin [3], a well grown seed culture of *D. fulvum* was used to inoculate (2%) larger volumes of either GYM/ISP2 medium (4 g/L glucose, 4 g/L yeast extract, 10 g/L malt extract, pH 7.2) or MYM medium (4 g/L mannitol 4 g/l, 4 g/L yeast extract, 10 g/L malt extract, pH 7.2) at 28 °C at 250 rpm. For bacterial culture on solid media, the same media were used with 1.5% agar. Compound production was typically followed by analytical UHPLC-MS, with the secondary metabolites of interest abundantly produced following 1 week culture.

General molecular biology techniques used

Genomic DNA was extracted from 1 mL *D. fulvum* cultures by bacterial disruption (bead beating), and standard phenol/chloroform/isoamyl alcohol DNA extraction procedure. DNA amplification for cloning or Sanger sequencing was achieved by PCR using Q5 High-Fidelity DNA polymerase.

Construction of *D.fulvum ΔpyrA*

An in-frame genetic inactivation of *Dfulv_14540 (pyrA)*, encoding an AMP-dependent synthetize and ligase previously demonstrated to be essential for pyridomycin biosynthesis [1], was achieved by standard double cross over gene removal of the majority of the gene (while keeping operon in-frame) as described previously to generate; *D. fulvum ΔpyrA* [2].

Construction of *D.fulvum ΔdafA*

Genetic inactivation of the primary NRPS gene *dafA* (*Dfulv_14720*) in *D. fulvum* was achieved through single cross over disruption of dafA (and likely the rest of its downstream operon) using a pDF3.1 based vector similar to that described previously [2] (non-replicating vector with AprR (apramycin resistance) cassette, and expressing XylE/T and oriT). Firstly, a 1321 bp region of *dafA* was amplified by PCR using primers sets (5'-ATAAGCTTAGCTGTCCTATGTGGAGCTCG-3' and 5'-ATGAATTCTGCCGGTGACATAGGCGA-3'), and cloned into pDF3.1 by restriction digest (*Eco*RI and *Hind*III), ligation and transformation into chemically competent *Escherichia coli* TOP10 cells (Thermofisher). The resultant vector, pDF3.1:dafA$_{Frag}$ was verified by Sanger sequencing. pDF3.1:dafA$_{Frag}$ was then transformed into chemically competent conjugation competent *E. coli* ET12567/pUZ8002, which was then used to transform *D. fulvum* by conjugation. Apramycin resistant *D. fulvum:ΔdafA* colonies were confirmed to have catechol-2,3-dioxygenase activity (from xylE/T on pDF3.1 backbone), and *dafA* disruption was confirmed by PCR confirmation.

RNA extraction and RT-qPCR

Gene expression analysis was performed on *D. fulvum* grown under different growth conditions: GYM media and GYM supplemented with 100 and 350 μM 2,2'-dipyridyl (iron-restrictive conditions). Briefly, 3 mL of an exponential culture of *D. fulvum* (OD_{600nm} of 0.7-0.8, indicated with an arrow in Table S6) was mixed directly with 2 volumes of RNAprotect® bacteria reagent (Qiagen), to instantly stabilize bacterial RNA. The bacteria were centrifuged (3200×g, 15 min, 4 °C) and RNA extraction was performed with the RNeasy® mini kit (Qiagen) following manufacturer's protocol for bacteria RNA extraction. This protocol includes a cell disruption step by bead beating: 3 cycles of 30 s (with 5 min on ice in-between) at maximum power, in tubes with lysing matrix B (MP Biomedicals™)

in a FastPrep system (MP Biomedicals™). Remaining genomic DNA depleted with the TURBO DNA-free™ kit (ThermoFisher Scientific). cDNA was synthesized with the LunaScript® RT SuperMix Kit (New England Biolabs) as per manufacturer's instructions. 20 µL real time PCR reactions were setup using 10 µL of 2X KAPA SYBR FAST qPCR Universal Master Mix (Sigma Aldrich), 1 µL of cDNA product and 0.4 µL of each 10 µM of each primer pair. Primer pairs are shown for the amplification of the genes of interest and housekeeping *sigA* are shown in Table SX. The real time PCR was followed on a LightCycler® 480 Instrument II (Roche) over 40 cycles (3 s at 98 °C and 40 s at 60 °C) followed by a melting curve analysis. Relative expression of the genes of interest were analyzed using the $\Delta\Delta C_t$ quantification method using *sigA* as housekeeping gene and the iron-rich condition (non-treated GYM) as reference condition. RT-qPCR experiments were performed on 4 independent biological replicates samples with 3 technical replicates.

Confocal fluorescence microscopy

All images were acquired using an inverted Eclipse Ti inverted confocal microscope (Nikon France Instruments, Champigny sur Marne, France) equipped with a CSU-W1 spinning-disk (Yokogawa, Roper Scientific, France). A live-SR module (Gataca Systems, France) was added to the system to improve the obtained resolutions. Observations were done with a 60× oil immersion objective (Nikon Plan Apo 60× NA 1.4), and bright field and fluorescence images (laser excitation at 561nm, and emission filter 595/50 nm) were acquired with sCMOS Prime 95B camera (Photometrics, UK). All the devices are piloted by MetaMorph (Molecular Devices, USA). Images were processed with the Huygens Professional Software (SVI, Cambridge, UK). After the deconvolution, images were analyzed using MicrobeJ plugin [3] in Fiji/ImageJ software [4].

To evaluate the uptake of TAMRA-derivatives, exponential phase cultures of *Dactylosporangium* strains were washed twice with PBS, resuspended in GYM media supplemented with 350 µM DIP (iron-restrictive media) and grown at 28 °C for 12 h with shaking at 250 rpm. Cells were washed twice with PBS and resuspended in 350 µM GYM with DIP to an OD_{600} of 0.1, and incubated with 1 µM **21** or **22** for 4 h. For competition experiment, 100 µM **2** was added together with 1 µM **22**. The cells were fixed with 4% paraformaldehyde in PBS for 1 hour, washed three times with PBS and placed on microscope slides with Mowiol® 4-88 (Sigma-Aldrich) mounting medium. Experiments with *S. aureus* and *P. aeruginosa* were performed in a similar fashion but incubating the cells with **21** or **22** for 1 h at 37 °C in CAMHB supplemented with 300 µM DIP.

3-pyridyl-TAMRA **21** and ClDaf-3-pyridyl-TAMRA **22** conjugate were excited with 561 nm laser and fluorescence was collected after a 595/50 nm bandpass filter. Images were analyzed with MicrobeJ. Briefly, bacterial cells were detected using the filament detection method (with default parameters and allowing for cell segmentation) and average fluorescence intensity per cell was measured. At least 3 independent images were analyzed per condition (> 200 individual bacteria). Kruskal–Wallis followed by Dunn's multiple comparison test was computed to compare the more than two datasets: n.s., not significant; **P = 0.002; ****P < 0.0001.

Antibacterial activity

The minimum inhibitory concentration (MIC) was determined using the resazurin microtitre assay (REMA). For *Dactylosporangium* strains, experiments were performed in 96-deep well plate format with a 400 µL final volume. Briefly, suspensions of the strains were prepared in GYM media supplemented with 100 µM DIP at an OD_{600} of 0.01. Compounds were tested in a concentration gradient through serial dilutions. After 2 days of incubation at 28°C with heavy shaking (to ensure the growth of strictly aerobic bacteria), 40 µL of resazurin 12.5% were added to each well, and an additional incubation was carried out for 2 h. 100 µL of each well were transferred to a 96-well plate and fluorescence was measured using a Fluostar Optima BMG Labtech (Ex: 530nm; Em: 590nm). Values were converted to resazurin turnover based on minimal (no bacteria added to medium) and maximal (no antibiotic added) fluorescence values. MIC_{95} values were determined when resazurin turnover was <5% of control. Results are determined from three independent experiments. Experiments with *E. coli*, *P. aeruginosa* and *S. aureus* were performed similarly, but in CAMHB media supplemented with 300 µM DIP in 96-well plate format with a final volume of 100 µL and 5 hours of incubation before the addition of 10 µL of resazurin.

Rifamycin resistant mutant selection

To select spontaneous Rif-resistant *D. fulvum* isolates, an exponential culture was concentrated to an OD_{600} of 10 and 100 µL spread onto GYM agar plates containing 10 mg/L of rifampicin (approximately 12.1 µM) (Sigma-Aldrich) and incubated until resistant colonies apparition (28°C, 2-3 weeks). Given that most of mutations conferring high-level resistance to rifampicin occur at the *rpoB* gene (encoding the RNA polymerase β subunit), this part of the gene was amplified with primers 5'-CGACATCGACCACTTCGGCAAC-3' and 5'-CGTCGACCACCTTGCGGTAC -3', and Sanger-sequencing was performed at GenoScreen (Lille, Fr). The

resistant isolates were then grown in antibiotic free liquid media to mid-log phase for the determination of antibiotic susceptibility by REMA.

RNA polymerase assay

Biochemical inhibition of transcription studies were performed using *E. coli* and *S. aureus* RNA polymerase kits (Profoldin products, bought from Mobitek, Germany). RNA synthesis assays were performed as per manufacturer's instructions in 30 µL reactions. Briefly, RNA polymerase (*S. aureus* or *E. coli*) was pre-incubated for 10 min (RT) with the single-stranded polyribonucleotide and serial dilutions of the compounds (0.0001-10 µM). Reaction was initiated by the addition of NTP mix, incubated at 37°C for 1 h and then stopped by adding the fluorescence dye in 30 µL. The fluorescence intensity was measured at an excitation wavelength of 485 nm and emission wavelength of 528 nm using an EnVision® 2105 (Perkin Elmer) or Infinite M200 Pro (Tecan) plate readers.

MALDI-TOF analysis of secondary metabolites

Dactylosporangium fulvum liquid cultures were grown in GYM/ISP2 medium or MYM medium at 28 °C at 250 rpm. Secondary metabolite detection on bacterial culture supernatants or pellet extracts were performed on a MALDI-TOF-TOF Autoflex speed (Bruker). Sample spots were prepared by the "dried droplet" method. Briefly, 1µl of supernatant was mixed with 1µL of 2,5-dihydroxybenzoic acid as matrix (HCCA) matrix and deposited on MALDI-TOF plate. Spectra were acquired in reflector, positive-mode.

Analytical mass spectrometry (UHPLC-MS)

Analytical (non-high resolution) spectrometry analysis (UHPLC-MS) were performed on an Ultimate 3000 UHPLC system, coupled with a LCQ Fleet Ion Trap Mass Spectrometer (Thermo Scientific). Chromatographic separation was achieved using an Acquity UPLC Peptide BEH C18 Column (300Å, 1.7 µm, 2.1 mm X 100 mm). Mobile phase system was composed of solvent A (H_2O, 0.1% formic acid) and B (acetonitrile, 0.1% formic acid), typically run through a linear gradient from 0% to 100% of solvent B in 10 min. Elution of compounds was monitored by UV absorbance at 215 nm and 254 nm, and by mass spectrometry by electrospray ionization.

High-resolution Mass spectrometry (HRMS)

High resolution mass spectrometry of purified compounds was performed by the ARIADNE-ADME platform (Institut Pasteur de Lille, France), using a quadrupole time-of-flight (TOF) LCT Premier XE mass spectrometry machine (Waters)

Preparative HPLC purification

Compounds were purified with a preparative RP-HPLC systems PLC 2020 (Gilson) using either a semi-preparative XBridge™ Peptide BEH C18 column (10 × 250 mm, 130 Å, 5 micron) or a preparative XBridge™ Peptide BEH C18 OBDTM column (19 × 150 mm, 130 Å, 5 micron) (Waters). Compounds were eluted using a gradient of solvent A (H_2O, 0.1% formic acid) to B (acetonitrile, 0.1% formic acid). The collected fractions were assayed by analytical UHPLC-MS to determine the molecular weights and purities, and fractions containing the target molecules were pooled together. The acetonitrile was evaporated from the fractions and the aqueous layer was lyophilized to obtain the compounds of interest.

Production and purification of pyridomycin, 1, from *D. fulvum*

Pyridomycin was purified from *D. fulvum* liquid cultures in a similar manner to that described previously [5]. Briefly, pyridomycin was first extracted from the culture supernatant using Amberchrom™ CG300 resin (Sigma-Aldrich), and eluted using methanol. Fractions containing pyridomycin were then dried and further enriched by liquid/liquid extraction using water/ethyl acetate. The organic layer containing pyridomycin was then dried and purified by preparative HPLC.

Production and purification of chlorodactyloferrin, 2, and dactyloferrin, 3, from *D. fulvum*

D. fulvum (8L) was grown in 500 mL volumes in GYM medium for 10 days at 28°C (250 rpm). Culture were centrifuged (6,900 x g for 30 minutes) and supernatant was mixed (30 min, RT) with 4 grams of Amberlite (weakly acidic cation exchanger, hydrogen form resin, Sigma-Aldrich). Following removal of the supernatant, the Amberlite resin was washed with 200 mL of ultrapure water, and then with 200 mL of 0.1 M NaCl, followed by elution of Amberlite resin bound compounds using 200 mL of 1M NaCl. The resulting impure extract was then purified with a preparative low-pressure C18 column (PF-30C18HP-F0040, Interchim, France) using a puriFlash XS 420+ system (Interchim). Dactyloferrin **3** and chlorodactyloferrin **2** were eluted using a linear gradient of solvent A (H_2O, 0.1% formic acid) to B (acetonitrile, 0.1% formic acid) over 30 min (flow rate of 20 mL/min). Automated fractions

collection was performed and fractions were analyzed for content by UHPLC-MS and/or MALDI-TOF analysis. Fractions containing purified (chloro)dactyloferrin were dried by lyophilization. Chlorodactyloferrin **2** (31 mg) and dactyloferrin **3** (5 mg) appeared as light brown powder. To generate the chlorodactyloferrin-Ga(III) complex, 2 equivalents of $GaCl_3$ were added to chlorodactyloferrin **2** and re-purified using a C18 column.

Determination of amino acid stereochemistry in ClDaf 2.

Stereochemistry of the amino acids constituting ClDaf was determined by the Marfey protocol [6–8]. Briefly, 0.5 mg of ClDaf **1** were hydrolyzed in 6 M HI at 110°C for 5 h (which hydrolyses ClDaf to individual amino acids, but also removes ornithines modifications to produce the base ornithines), and derivatized with N^{α}-(2,4-dinitro-5-fluorophenyl)-L-alaninamide (FDAA, 1% solution in acetone, 40 µL) (Sigma-Aldrich) at 37°C for 1 h. Amino acid standards: glycine, L-serine, D-serine, L-arginine, D-arginine, L-ornithine and D-ornithine (Sigma-Aldrich) were derivatized following the same procedure. FDAA-derivatized samples were then analyzed by analytical LCMS-UV using an Acquity UPLC Peptide BEH C18 Column (300Å, 1.7 µm, 2.1 mm X 100 mm). Solvent A was H_2O with 0.1% formic acid and solvent B, acetonitrile with 0.1% formic acid. Elution of compounds was achieved with a linear gradient from 0 to 60% of solvent B, and monitored by UV absorbance at 340 nm, and by mass spectrometry by electrospray ionization.

Determination ClDaf-pyridyl-rifampicin 26 stability in "spent culture" filtrate.

"Spent" media was prepared from the culture filtrate of a well developed *D. fulvum* culture growing in GYM media (similar conditions used for MIC analysis, by centrifuging the culture and filtering the culture supernatant (0.22 µm filter). 100 µM **26** was spiked into this spent media and incubated at 30°C. To measure the conjugate stability, samples (100 µL) were taken at different time points (0 1 and 2 days), mixed with an equal volume of acetonitrile, stored at -20°C, centrifuged, and the supernatant analyzed by analytical UHPLC-MS as described above. The amount of **26** conjugate in the samples was measured from the area under the peak of the extracted single ion chromatogram of the double charge **26** (mass call: 770.0), and expressed as a percentage of that measured at time 0.

NMR

NMR experiments were run at 293K on a Bruker AVANCEIII 300 spectrometer equipped with a broad-band direct probe and on a Bruker AVANCEIIIHD 600 spectrometer equipped with a $^1H/^2H/^{13}C/^{15}N$-TXI and a cryo-$^1H/^2H/^{13}C/^{15}N/^{19}F$-QCI probes. The (chloro)dactyloferrin alone and the Ga(III)-complex were solubilized into 90%-H_2O/10%-D_2O or 100%-D_2O with DSS as a reference. The ClDaf:Ga(III)-Pyr conjugate **4** was dissolved in acetonitrile-d3/water (H_2O or D_2O), 1/1, v/v and transferred into a hermetic Young-valve NMR tube. Mono-dimensional ^1H, $^{19}F\{^1H\}$, $^{13}C\{^1H\}$, ^{13}C-DEPT45 and ^{13}C-DEPT135, bi-dimensional ^1H-COSY, ^1H-TOCSY, ^1H-NOESY, ^1H-ROESY, ^1H-^{13}C-HSQC, ^1H-^{13}C-HSQC-DEPT, ^1H-^{13}C-HSQC-TOCSY, ^1H-^{13}C-HMBC, ^1H-^{15}N-HSQC, ^1H-^{15}N-HSQC-DEPT, ^1H-^{15}N-HMBC were recorded .

Supplementary Tables

Table S1: Description of the BlastP results and proposed function of genes in the ClDaf BGC

Locus tag	Gene name	Best BlastP result [organism]	% ident	% cover	% similarity	Proposed function
Dfulv_14680	*dafT1*	ABC transporter ATP-binding protein [*Actinoplanes ovalisporus*]	91.94	89	96	Ferric ClDaf import – FepC
Dfulv_14685	*dafT2*	iron chelate uptake ABC transporter family permease subunit [*Dactylosporangium sucinum*]	88.6	100	93	Ferric ClDaf import - FepG
Dfulv_14690	*dafT3*	iron ABC transporter permease [*Dactylosporangium sucinum*]	91.45	88	95	Ferric ClDaf import - FepD
Dfulv_14695	*dafT4*	ABC transporter substrate-binding protein [*Dactylosporangium sucinum*]	92.83	90	96	Ferric ClDaf import - FepB
Dfulv_14700	*dafJ*	acetyltransferase [*Dactylosporangium sucinum*]	86.8	99	91	Acetylation of ornithine moieties
Dfulv_14705	*dafG*	phosphopantetheine-binding protein [*Actinoplanes sp. L3-i22*]	94.81	100	98	Covalent binding of DHB moiety
Dfulv_14710	*dafH*	tryptophan 7-halogenase [*Dactylosporangium sucinum*]	93.76	100	96	Chlorination of DHB
Dfulv_14715	*dafI*	MbtH family protein [*Dactylosporangium sucinum*]	86.11	100	88	Accessory NRPS protein
Dfulv_14720	*dafA*	non-ribosomal peptide synthetase [*Dactylosporangium sucinum*]	80.57	98	84	Core peptide biosynthesis
Dfulv_14725	*dafB*	non-ribosomal peptide synthetase [*Dactylosporangium sucinum*]	80.39	99	85	Core peptide biosynthesis
Dfulv_14730	*dafT5*	ABC transporter ATP-binding protein/permease [*Dactylosporangium sucinum*]	87.25	100	90	ClDaf export
Dfulv_14735	*dafT6*	ABC transporter ATP-binding protein/permease [*Dactylosporangium sucinum*]	83.99	100	88	ClDaf export
Dfulv_14740	*dafK*	alpha/beta hydrolase [*Actinoplanes durhamensis*]	73.51	99	85	Potential Thioesterase
Dfulv_14745	*dafM*	siderophore-interacting protein [*Dactylosporangium sucinum*]	86.52	100	92	Potential Reductase
Dfulv_14750	*dafT7*	cation:proton antiporter [*Dactylosporangium sucinum*]	85.88	96	94	Unknown
Dfulv_14755	*dafE*	2,3-dihydro-2,3-dihydroxybenzoate dehydrogenase [*Dactylosporangium sucinum*]	91.8	97	91	DHB biosynthesis
Dfulv_14760	*dafC*	isochorismate synthase [*Dactylosporangium sucinum*]	89.68	95	91	DHB biosynthesis
Dfulv_14765	*dafF*	(2,3-dihydroxybenzoyl)adenylate synthase [*Dactylosporangium sucinum*]	86.42	100	92	DHB biosynthesis
Dfulv_14770	*dafD*	isochorismatase family protein [*Dactylosporangium sucinum*]	88.07	100	90	DHB biosynthesis
Dfulv_14775	*dafL*	lysine N(6)-hydroxylase/L-ornithine N(5)-oxygenase family protein [*Dactylosporangium sucinum*]	85.61	95	76	Hydroxylation of ornithine

Table S2: $^{1}H/^{13}C/^{15}N$ chemical shifts assignments of ClDaf [2](left), ClDaf:Ga(III)(middle), and Daf [3](right) in water at 293K and 14 T. In bold, the significant differences between ClDaf:Ga(III) and ClDaf (middle), and between Daf and ClDaf (right).

		ClDaf alone				ClDaf:Ga(III)				Daf alone			
		δ_H / ppm	Mult.	δ_C / ppm	δ_N / ppm	δ_H / ppm	Mult.	δ_C / ppm	δ_N / ppm	δ_H / ppm	Mult.	δ_C / ppm	δ_N / ppm
		(600 MHz)	Hz	(150 MHz)	(61 MHz)	(600 MHz)	Hz	(150 MHz)	(61 MHz)	(600 MHz)	Hz	(150 MHz)	(61 MHz)
**ClDHB1**	1			117.4				**120.3**				**118.9**	
	2			151.5				**155.5**				**149.8**	
	3			144.0				**151.6**				**147.4**	
	4			128.2				**122.9**		**7.12**	d 8.0	**122.5**	
	5	7.01	d 8.8	122.9		**6.70**	d 8.6	**119.5**		6.90	t 8.0	122.6	
	6	7.29	d 8.8	122.2		**6.63**	d 8.6	**117.2**		7.31	d 8.0	122.0	
	7			173.0				**175.0**				173.5	
**Gly2**	1	4.11&4.16		45.6		3.86&4.50		45.6		4.13&4.18		45.6	
	2			174.7				**176.4**				174.7	
	**1-HN**	9.07			109.3	8.74			115.5	9.03	t 5.1		109.4
**D-Arg3**	1	4.31		56.7		4.28		57.4		4.34		56.6	
	2	1.78&1.89		30.5		1.62&1.98		29.3		1.81&1.90		30.53	
	3	1.63&1.68		27.3		1.52&1.62		28.1		1.65&1.70		27.2	
	4	3.20		43.2		**2.93&2.99**		43.5		3.22		43.2	
	5			159.3				159.1				159.4	
	6			176.9				178.2				176.9	
	**1-HN**	8.57	d 6.2		120.9	**9.30**	d 7.7		120.9	8.58	d 6.3		121.0
	**4-HN**	7.19	t 5.3		84.7	**6.74**			84.1	7.21	t 5.4		84.7
	**5-H_2N**	6.7	br			**6.47**	br			6.7	br		
**L-Orn4**	1	4.36		56.2		4.23		57.6		4.37		56.1	
	2	1.67&1.75		30.7		**1.89&1.97**		30.3		1.81&1.94		30.55	
	3	1.69		26.1		1.60&1.70		26.5		1.72		26.1	
	4	2.97	t 7.4	41.8		2.96		41.2		2.99		41.5	
	5			176.1				176.6				176.2	
	**1-HN**	8.49	d 7.0		120.2	**8.85**	d 6.6		**118.2**	8.50	d 7.2		121.2
	**4-H_2N**	7.64	br			7.64	t 4.0		32.5	7.62	br		31.8
**D-AcOHOrn5**	1	4.29		56.3		4.38		**58.9**		4.31		56.2	
	2	1.67&1.75		30.6		**1.76&2.04**		29.5		1.71&1.78		30.58	
	3	1.58		25.1		**1.95**		**27.6**		1.62		25.1	
	4	3.51		49.8		**3.39&4.08**		**52.3**		3.55		49.8	
	5			172.1				**165.8**				172.1	
	6	2.03	s	22.3		**2.18**		**18.3**		2.05		22.3	
	7			176.5				**179.5**				172.1	
	5'			176.4						2.12		22.0	
	6'	2.10	s	22.0								176.5	
	**1-HN**	8.25	d 6.5		121.2	9.24			122.1	8.32	d 6.6		121.5
	**4-NOH**				178				**197**				178
	**4-NOH'**				180								180
**D-Orn6**	1	4.34		56.1		**4.05**		**58.7**		4.36		56.0	
	2	1.78&1.87		30.5		1.88&1.93		29.3		1.81&1.90		30.48	
	3	1.73		25.9		1.79&1.87		26.3		1.75		25.9	
	4	3.01	t 7.5	41.9		3.04		41.4		3.01	t 7.3	41.6	
	5			175.8				**177.6**				175.8	
	**1-HN**	8.40	d 6.6		122.0	**9.25**			**126.7**	8.41	d 6.8		121.9
	**4-H_2N**	7.64	br			7.64	br		32.5	7.62	br		31.5
**D-Ser7**	1	4.43		58.1		**4.21**		58.8		4.44		58.2	
	2	3.82		63.9		3.81&4.11	d 12.0	63.0		3.84		63.9	
	3			173.7				173.8				173.7	
	**1-HN**	8.34	d 7.1		117.7	**8.00**	d 5.3		**109.5**	8.35	d 7.2		117.6
**L-cOHOrn8**	1	4.49		53.1		**4.79**		**50.1**		4.51		53.1	
	2	1.79&2.04		29.4		1.81&2.01		28.8		1.82&2.07		29.3	
	3	1.96&2.01		22.7		1.92&2.10		**20.1**		1.96&2.06		22.7	
	4	3.63&3.68		54.4		3.60&3.66	d 12.8	53.2		3.64&3.70		54.4	
	5			169.3				**161.4**				169.3	
	**1-HN**	8.48	d 8.1		124.1	**7.47**	d 8.6		**121.6**	8.48	d 8.5		124.0
	**4-NOH**								**203**				176

Table S3: $^{1}H/^{13}C/^{15}N$ chemical shifts assignments of ClDaf Gallium complex (**2:gallium**) in water with DSS for CS referencing, and ClDaf-Pyr Gallium complex (**4:gallium**) in acetonitrile/water 1/1 v/v referenced against acetonitrile residual peaks, at 293K and 14 T.

		ClDaf:Ga(III) in water				ClDaf:Ga-Pyr in water/ACN			
		δ_H / ppm (600 MHz)	Mult. Hz	δ_C / ppm (150 MHz)	δ_N / ppm (61 MHz)	δ_H / ppm (600 MHz)	Mult. Hz	δ_C / ppm (150 MHz)	δ_N / ppm (61 MHz)
**ClDHB1**	**1**			120.3					
	**2**			155.5					
	**3**			151.6					
	**4**			122.9					
	**5**	6.70	d 8.6	119.5		6.61		122.6	
	**6**	6.63	d 8.6	117.2					
	**7**			175.0					
**Gly2**	**1**	3.86&4.50		45.6		3.63&4.12		43.6	
	**2**			176.4					
	**1-HN**	8.74			115.5	8.72			119.3
**D-Arg3**	**1**	4.28		57.4		4.17		54.9	
	**2**	1.62&1.98		29.3		1.45&1.86		27.5	
	**3**	1.52&1.62		28.1		1.36		26.3	
	**4**	2.93&2.99		43.5		2.80		41.5	
	**5**			159.1				158.0@318K	
	**6**			178.2					
	**1-HN**	9.30	d 7.7		120.9	8.83			119.0
	**4-HN**	6.74			84.1	6.90			84.7
	**5-H_2N**	6.47	br						
**L-Orn4**	**1**	4.23		57.6		3.98		56.0	
	**2**	1.89&1.97		30.3		1.73&1.79		28.7	
	**3**	1.60&1.70		26.5		1.59&1.71		24.7	
	**4**	2.96		41.2		2.84		39.4	
	**5**			176.6					
	**1-HN**	8.85	d 6.5		118.2	8.25			116.9
	**4-H_2N**	7.64	br		32.5	7.45			
**D-AcOHOrn5**	**1**	4.38		58.9		4.19		57.0	
	**2**	1.76&2.04		29.5		1.64&1.81		25.9	
	**3**	1.95		27.6		ca. 1.90		27.9	
	**4**	3.39&4.08		52.3		3.31&3.91		50.3	
	**5**			165.8				164.2	
	**6**	2.18		18.3		2.06		16.0	
	**5'**			179.5					
	**6'**								
	**7**								
	**1-HN**	9.24			122.1	9.17			121.2
	**4-NOH**				197				197@318K
	**4-NOH'**								
**D-Orn6**	**1**	4.05		58.7		3.91		56.8	
	**2**	1.88&1.93		29.3		ca. 1.94		27.6	
	**3**	1.79&1.87		26.3		1.68&1.73		24.4	
	**4**	3.04		41.4		2.90		39.6	
	**5**			177.6					
	**1-HN**	9.25			126.7	8.87			125.1
	**4-H_2N**	7.64	br		32.5	7.47			
**D-Ser7**	**1**	4.21		58.8		4.10		56.7	
	**2**	3.81&4.11	d 12.0	63.0		3.67&4.03		61.3	
	**3**			173.8					
	**1-HN**	8.00	d 5.4		109.5	7.55			107.7
**L-cOHOrn8**	**1**	4.79		50.1		4.71		48.0	
	**2**	1.81&2.01		28.8		1.68&2.07		27.2	
	**3**	1.92&2.10		20.1		1.81&1.87		18.1	
	**4**	3.60&3.66	d 12.8	53.2		3.51		51.5	
	**5**			161.4				160.1@318K	
	**1-HN**	7.47	d 8.7		121.6	7.34			120.6
	**4-NOH**				203				

Table S4: Summary of the conjugation reactions performed between various model catechol and pyridine derivatives, using different oxidants, and their outcome.

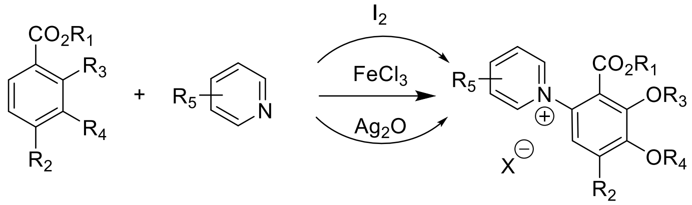

Model Catechol					Model Pyridine		Oxidant	Predicted Product Detection			
**Comp No.**	**R_1**	**R_2**	**R_3**	**R_4**	**Comp No.**	**R_5**		**Comp. Mass**	**Det.**	**AUC (mass call)**	**Comp No.**
[5]	Me	Cl	OH	OH	[12]	3-Me	Fe(III)	294.1	YES	222898	[16a]
[5]	Me	Cl	OH	OH	[12]	3-Me	I_2	294.1	YES	367233	[16b]
[5]	Me	Cl	OH	OH	[12]	3-Me	Ag_2O	294.1	YES		[16c]
[6]	Me	H	OH	OH	[12]	3-Me	Fe(III)	260.1	YES	12646	[17a]
[6]	Me	H	OH	OH	[12]	3-Me	I_2	260.1	YES	178706	[17b]
[7]	Me	Cl	OMe	OMe	[12]	3-Me	Fe(III)	322.1	NO		
[7]	Me	Cl	OMe	OMe	[12]	3-Me	I_2	322.1	NO		
[7]	Me	Cl	OMe	OMe	[12]	3-Me	Ag_2O	322.1	NO		
[8]	Me	H	OMe	OMe	[12]	3-Me	Fe(III)	288.1	NO		
[8]	Me	H	OMe	OMe	[12]	3-Me	I_2	288.1	NO		
[8]	Me	H	OMe	OMe	[12]	3-Me	Ag_2O	288.1	NO		
[9]	Me	H	OH	OMe	[12]	3-Me	Fe(III)	274.1	NO		
[9]	Me	H	OH	OMe	[12]	3-Me	I_2	274.1	NO		
[9]	Me	H	OH	OMe	[12]	3-Me	Ag_2O	274.1	NO		
[10]	Me	OH	OH	H	[12]	3-Me	Fe(III)	260.1	NO		
[10]	Me	OH	OH	H	[12]	3-Me	I_2	260.1	NO		
[10]	Me	OH	OH	H	[12]	3-Me	Ag_2O	260.1	NO		
[11]	Gly-OMe	Cl	OH	OH	[12]	3-Me	Fe(III)	351.1	YES		[18a]
[11]	Gly-OMe	Cl	OH	OH	[12]	3-Me	I_2	351.1	YES		[18b]
[11]	Gly-OMe	Cl	OH	OH	[12]	3-Me	Electro.	351.1	YES		[18d]
[5]	Me	Cl	OH	OH	[13]	4-Me	Fe(III)	294.1	YES	338909	[19a]
[5]	Me	Cl	OH	OH	[13]	4-Me	I_2	294.1	YES	355743	[19b]
[5]	Me	Cl	OH	OH	[13]	4-Me	Ag_2O	294.1	YES		[19c]
[5]	Me	Cl	OH	OH	[14]	2-Me	Fe(III)	294.1	YES	13574	[20a]
[5]	Me	Cl	OH	OH	[14]	2-Me	I_2	294.1	YES	62958	[20b]
[5]	Me	Cl	OH	OH	[14]	2-Me	Ag_2O	294.1	YES		[20c]
[5]	Me	Cl	OH	OH	[15]	2,6-Me	Fe(III)	308.1	NO		
[5]	Me	Cl	OH	OH	[15]	2,6-Me	I_2	308.1	NO		
[5]	Me	Cl	OH	OH	[15]	2,6-Me	Ag_2O	308.1	NO		

Table S5. Antibiotic susceptibility of *D. fulvum* in different media to 3-pyridyl-penicillin **23**, 3-pyridyl rifampicin **25**, and their respective ClDaf **2** conjugates **24** and **26**. Bacterial viability was determined using resazurin reduction and data are an average of 3 biological replicates.

**Media condition**	**Minimal Inhibitory Concentration (µM)**			
	3-pyridyl-penicillin **23**	ClDaf-3-pyridyl-penicillin conjugate **24 :iron**	3-pyridyl-rifampicin **25**	ClDaf-3-pyridyl-rifampicin conjugate **26 :iron**
**GYM**	0.62-1.2	2.5	2.5	10
**GYM + 100 µM DIP**	0.62	2.5	2.5	10
**GYM + 350 µM DIP**	0.62	2.5	2.5	10

Table S6: Primers used for RT-qPCR analysis.

**Gene**	**Forward primer (5' to 3')**	**Reverse primer (5' to 3')**
dafT3	AGCATCGACTGTGTGAACG	GTCGTGATCGGCATCCAG
dafT4	GCCAAACTCAGCAAACTCG	TCTTGACCTTGGAGATCTTGC
dafA	TCAAACAGGTCAAGGAGCAG	CCAGGTAGTTGAACAGGATCTC
dafB	GGAGTTGTATCTGTCGGGTG	CACGGAGCTTCACCTGATC
pyrA	ACTACGCCTACCAGATCCG	AGGTGAAGTTGAACTCGATCG
sigA	TTGATCACCTCGACCATGTG	GCTACAAGTTCTCCACCTACG
Dfulv_43390	TACATCCGCGAGAGCTTCTA	GTAGCGCGAGTTGTGAATGA
Dfulv_43395	CCAACCAGGTCGACAAGAG	GGATTGAGCAGGGTGAACTT

Supplementary Figures

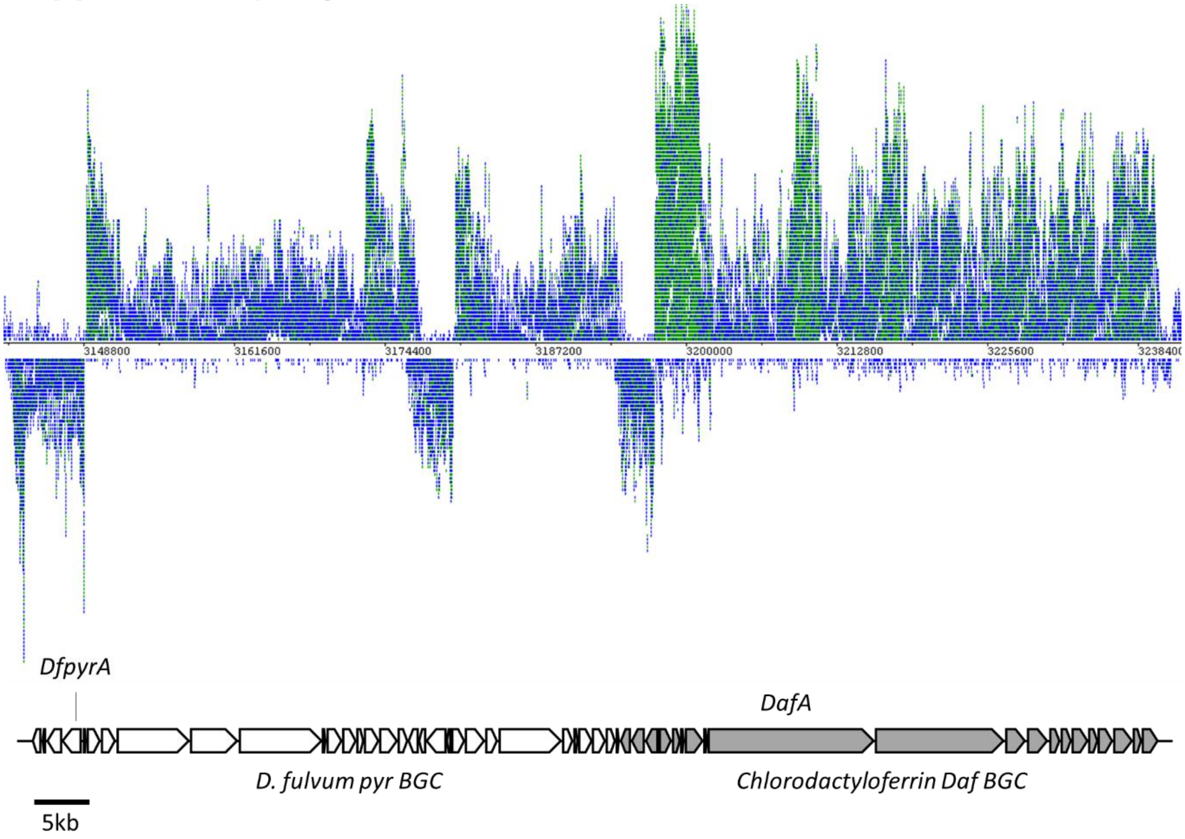

Figure S1: Transcription profile of the Pyr and ClDaf hybrid cluster in *D. fulvum* (grown to late log phase) as determined by RNAseq. The Image generated in Artemis[9] shows the mapped directional RNAseq reads to the *D. fulvum* hydrid Pyr-ClDaf BGC (shown below).

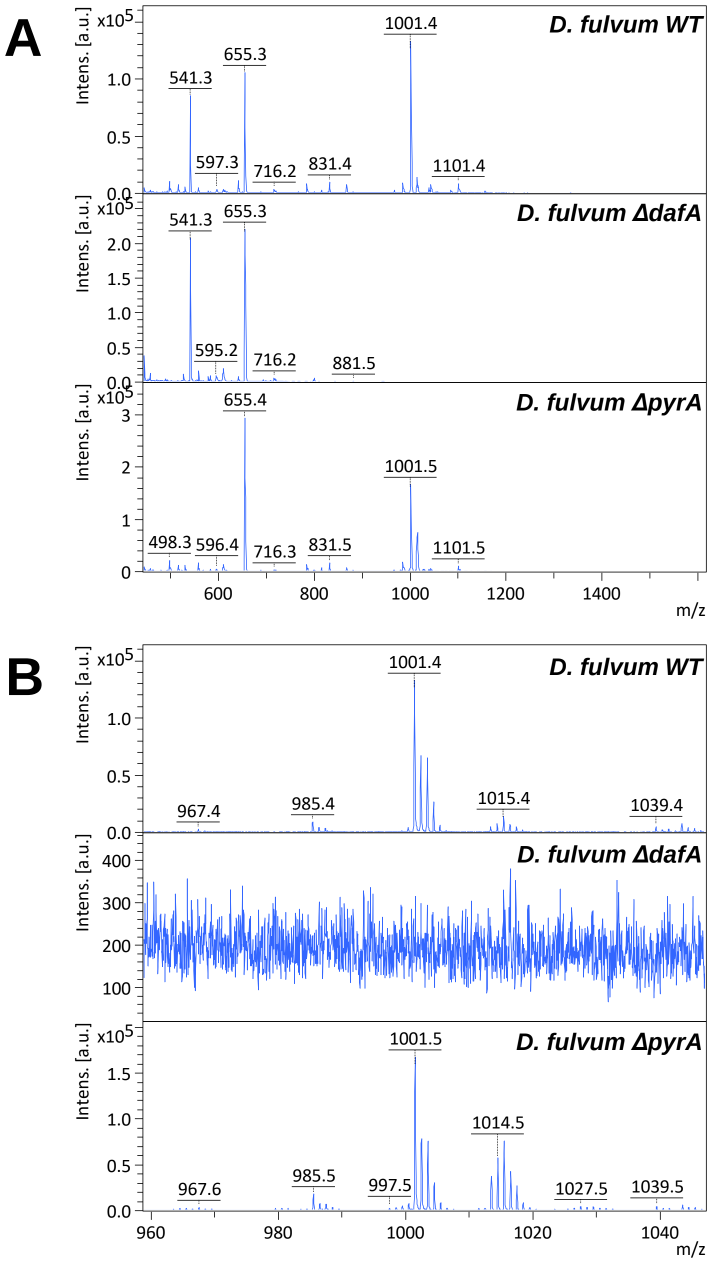

Figure S2. MALDI-TOF mass spectrometry spectra with a m/z range of **A**) 450-1600 and **B**) 960-1045, for the culture supernatant wild-type *D. fulvum (top) and mutant D. fulvum ΔdafA (middle) and D. fulvum ΔpyrA (bottom)*. In the supernatant of wild-type *D. fulvum*, mass signals (low resolution) can be seen for numerous metabolites, including Pyr **1** ($[M+H]^+$ = 541.2), ClDaf **2** ($[M+H]^+$=1001.5) and Daf **3** (967.6 Da). The isotope profile of ClDaf **2** agrees to that of a mono-chlorinated compound. Data shows that *D. fulvum ΔpyrA* no longer produces Pyr **1**, while *D. fulvum ΔdafA* no longer produces ClDaf **2** and Daf **3**, as well as a number of other uncharacterized secondary metabolites likely related to ClDaf **2**.

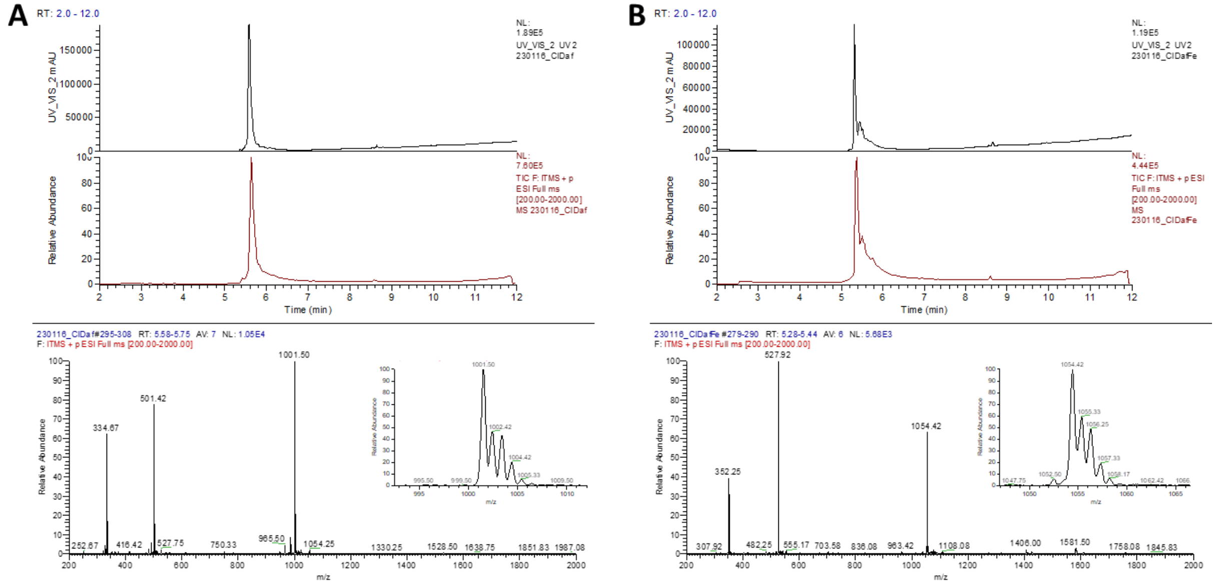

Figure S3. **Evidence of ClDaf iron binding by UHPLC-MS.** UHPLC-MS Chromatographic analysis (non-HRMS) of **A**) ClDaf **2** alone and **B**) ClDaf **2** with 1 eq of ferric iron ($FeCl_3$). Top chromatogram (UV 215 nm absorption), second chromatogram (Total ion current), Bottom: mass spectrum of peak, with insert showing zoomed view of parent peak. Data shows clear iron binding of ClDaf **2** in the presence of Iron.

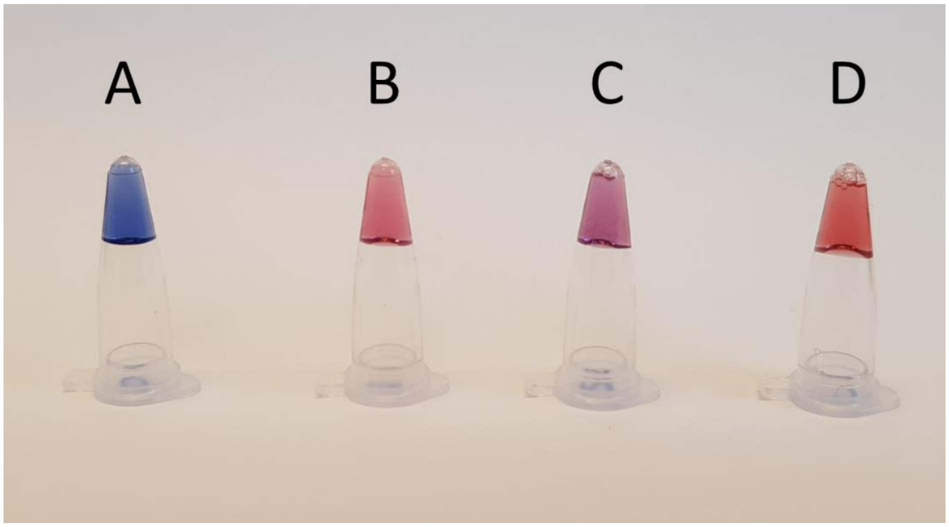

Figure S4. ClDaf, **2,** iron binding by CAS assay. Image of the CAS (Chrome Azurol S) assay. Images show compound mediated color changes in the assay induced by **A**) DMSO, (no colour change control), **B**) 10 mM desferroxamine B, **C**) 10 mM Daf, **3** **D**) 10 mM ClDaf, **2**

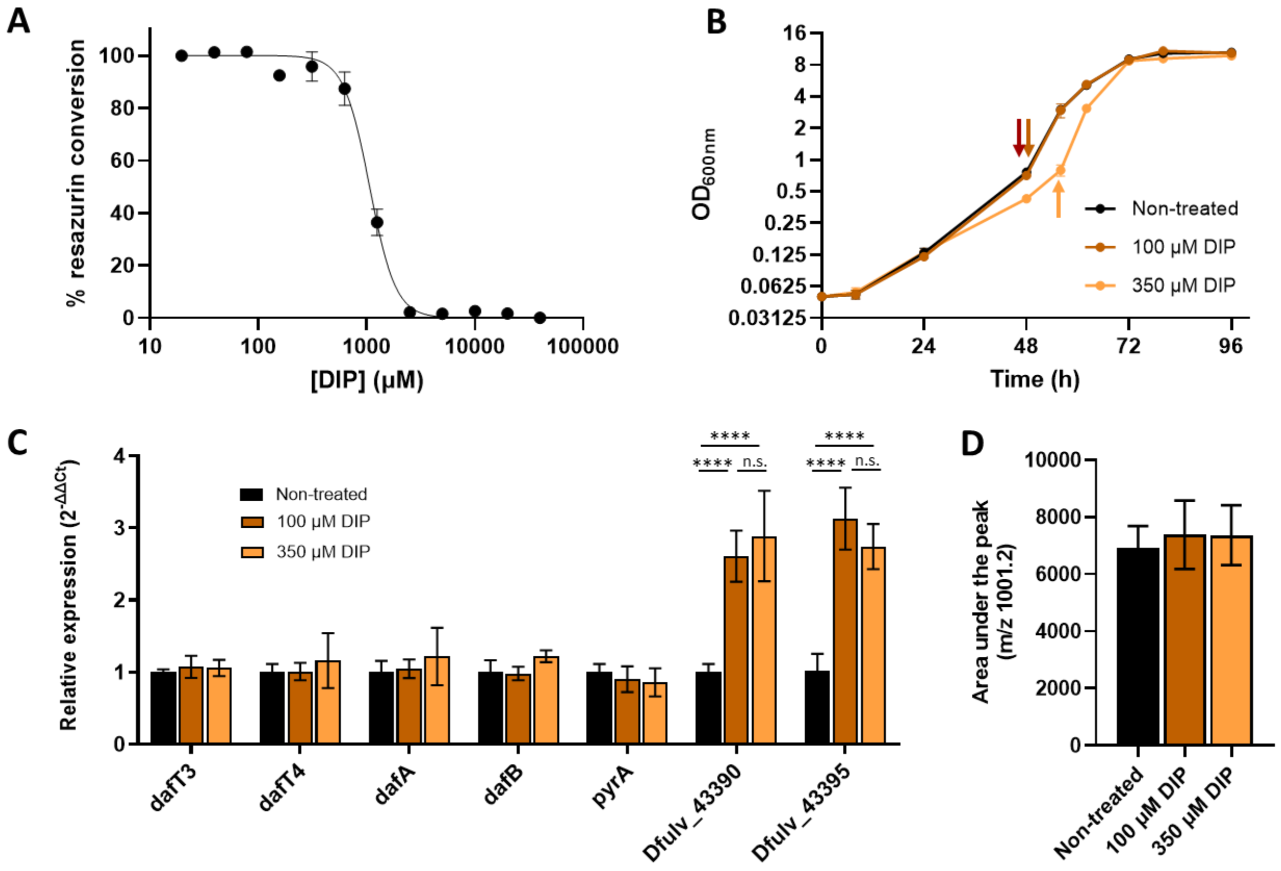

Figure S5. **A**) Measurement of the minimum inhibitory concentration (MIC) of DIP against *D. fulvum* WT in GYM media by the resazurin reduction method. Data are the mean ± SD of 4 independent experiments. The MIC for DIP was 2.5 mM. with 100 and 350 µM DIP not showing appreciable effect on cellular viability. **B)** Growth curve of *D. fulvum* WT GYM media and GYM media supplemented with 100 and 350 µM DIP. 350 µM DIP showed a small growth retardation, while 100 µM DIP had no effect on bacterial growth compared to the untreated control. Data shown are mean ± SD of 4 independent experiments. Arrow indicate sample points for gene expression studies in C) and ClDaf **2**, production in D) where the OD_{600nm} was between 0.7-0.8. **C**) RT-qPCR analysis of selected genes in response to iron depletion. Selected genes are involved in ClDaf uptake (*dafT3* and *dafT4*) and biosynthesis (*dafA* and *dafB*), in Pyr **1** biosynthesis (*pyrA*)¸and in the biosynthesis of another predicted *D. fulvum* siderophore: deferoxamine (*Dfulv_43390* and *Dfulv_43395)* (AntiSMASH prediction). Relative gene expression was determined by the $\Delta\Delta C_t$ quantification method using *sigA* (*rpoD)* as housekeeping gene and the iron-normal condition (GYM media) as reference condition. Data show gene expression of the ClDaf-Pyr hybrid cluster to not be regulated by DIP while this did mediate the induction of genes associated with the biosynthesis of deferoxamine. RT-qPCR experiments were performed on 4 independent biological samples with 3 technical replicates. **D**) ClDaf **2**, production by *D. fulvum* in the absence and presence of 100 of 350 µM DIP as measured in crude extracts by UHPLC-MS (ion call, $[M+H]^+$ = 1001.2). ClDaf production does not vary significantly regarding different iron-availability conditions, in line with the RT-qPCR data for the expression of the genes in its BGC.

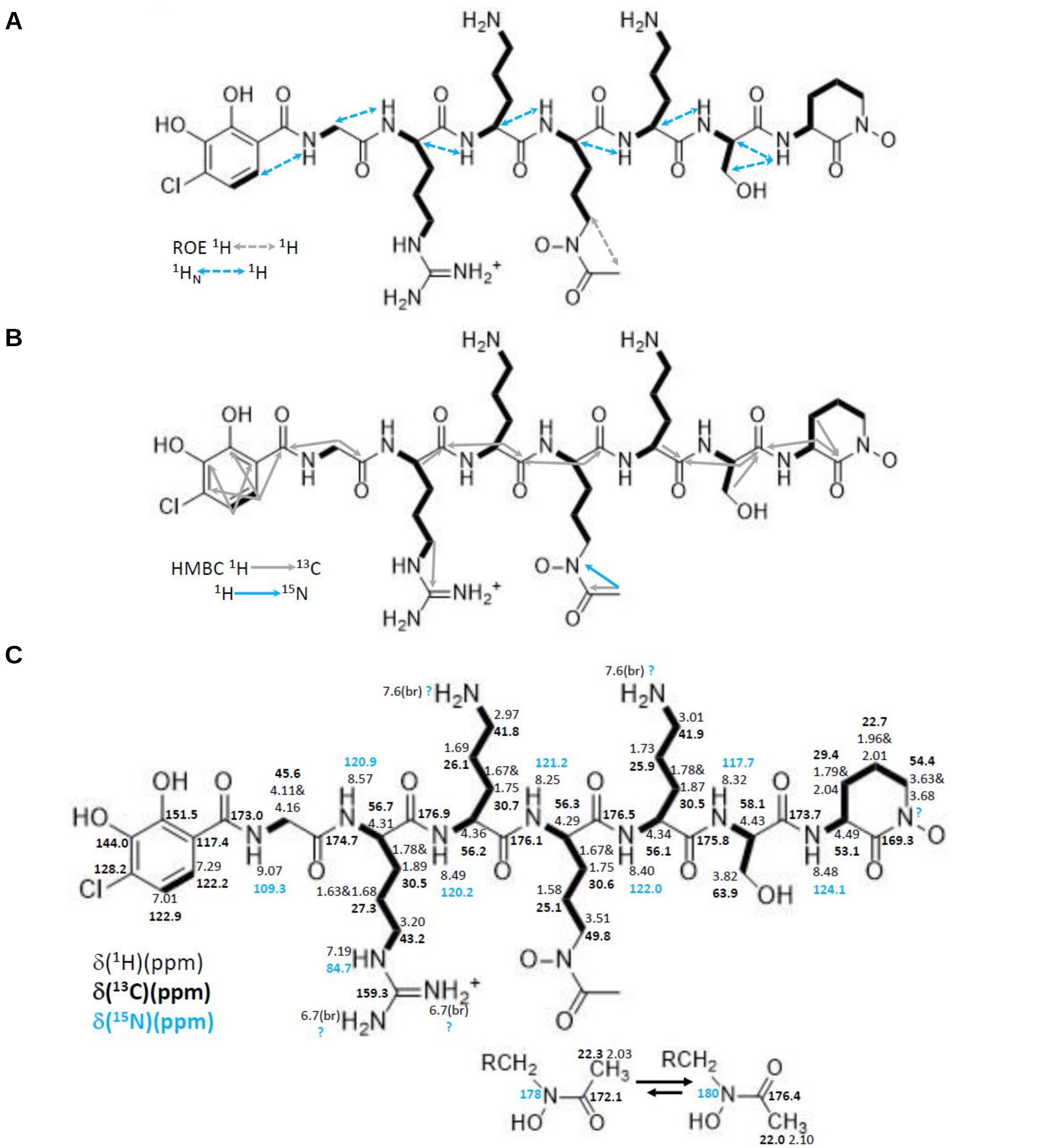

Figure S6: NMR characterization of ClDaf **2** in water at 293K and 14 T. Confirmation of the structure of ClDaf **2** through **A,** pertinent homonuclear ROE correlations (dotted arrows) between isolated spins system (bold bonds), and **B,** heteronuclear long-range scalar correlations (plain arrows) from HMBC to identify quaternary ^{13}C and ^{15}N. **C**) A summary of ^{1}H, ^{13}C and ^{15}N chemical shifts assignment of the ClDaf **2**.

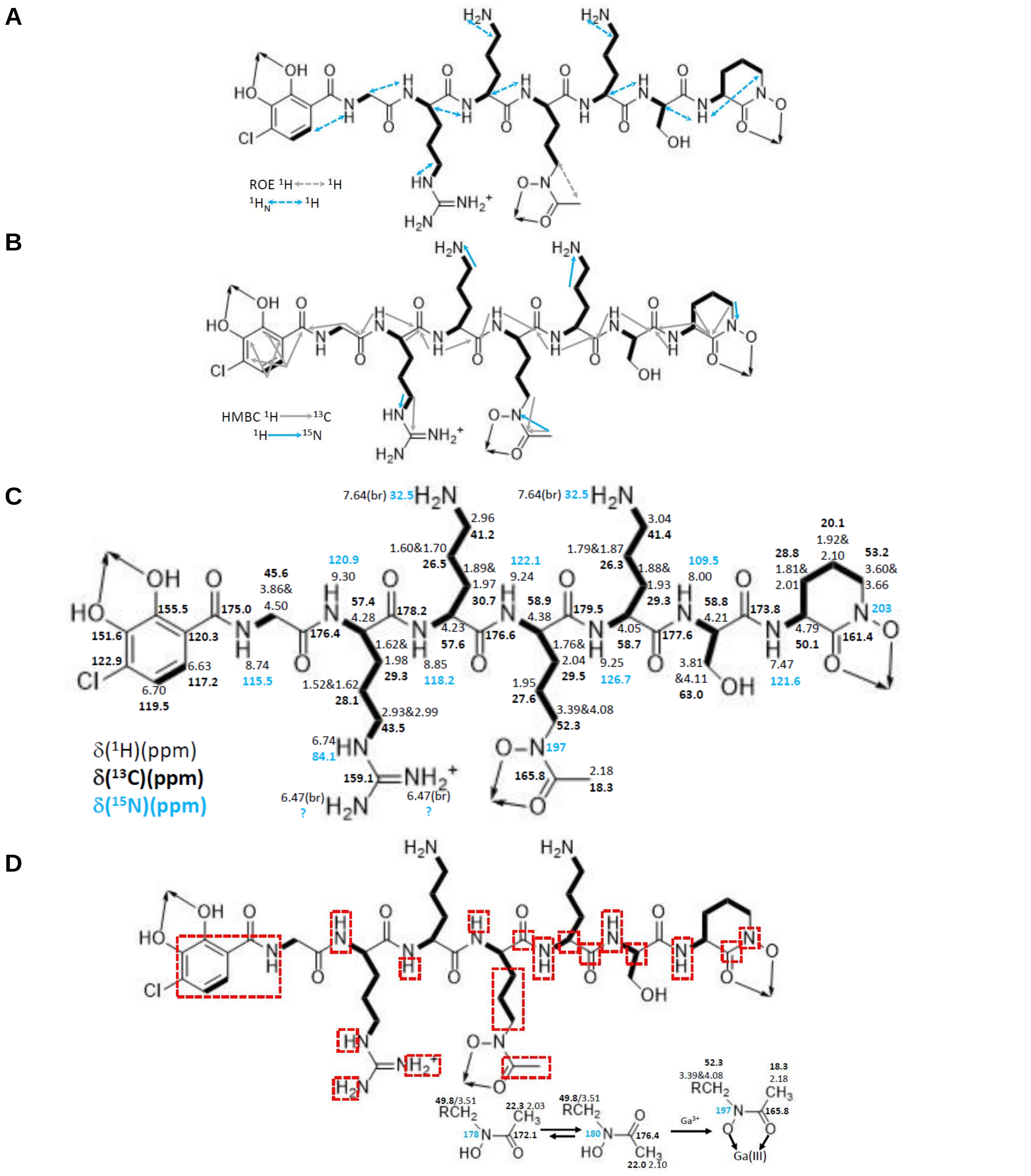

Figure S7: NMR characterization of ClDaf gallium(III) complex, **2:gallium** in water at 293K and 14 T. **A,** Confirmation of the structure of **2:gallium** through pertinent homonuclear ROE correlations (dotted arrows) between isolated spins system (bold bonds) and **B**, heteronuclear long-range scalar correlations (plain arrows) from HMBC to identify quaternary ^{13}C and ^{15}N . **C**) A Summary of ^{1}H, ^{13}C and ^{15}N chemical shifts assignment of **2:gallium**. **D**) Indication of the positions of ClDaf **2,** affected by the gallium complexation.

A

B

C

D

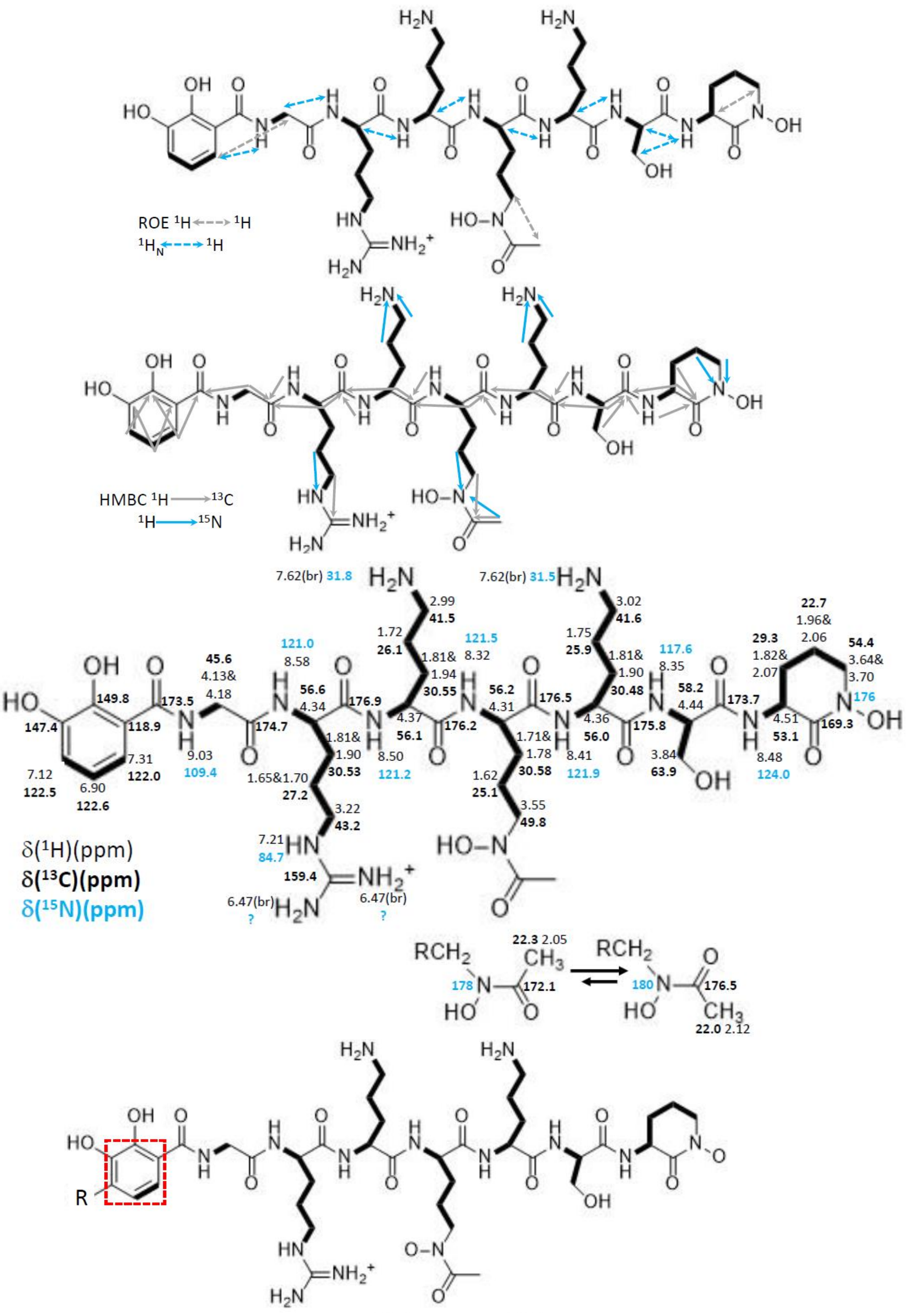

Figure S8: NMR characterization of dactyloferrin, **3** in water at 293K and 14 T. **A,** Confirmation of the structure of Daf, **3** through pertinent homonuclear ROE correlations (dotted arrows) between isolated spins system (bold bonds), and **B,** heteronuclear long-range scalar correlations (plain arrows) from HMBC to identify quaternary ^{13}C and ^{15}N. **C,** A summary of ^{1}H, ^{13}C and ^{15}N chemical shifts assignment of the molecule. **D,** Chemical shifts positions affected by the Hydrogen/Chlorine (R) replacement into the 2,3-dihydroxy-benzoate (DHB) moiety for Daf, **3** (R=H) and ClDaf, **2** (R=Cl).

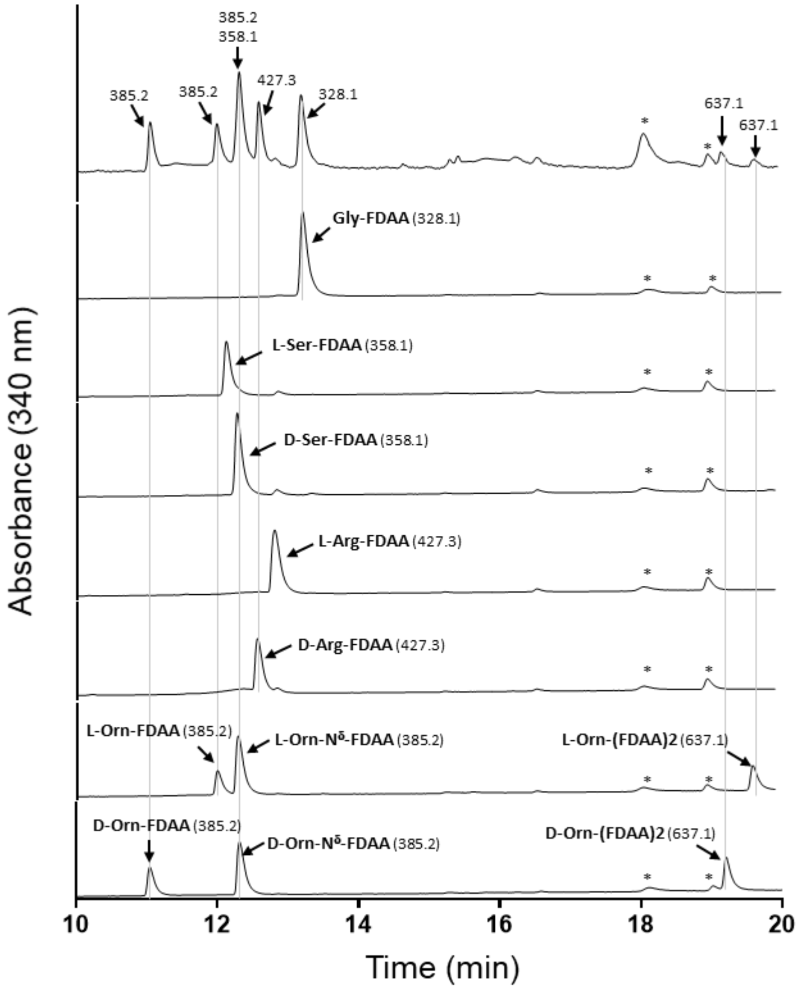

Figure S9: Analysis of the stereochemistry of the ClDaf, **2** amino acids using the Marfey protocol [6–8]. Data shows the UHPLC chromatogram (340 nm) of the HI acid hydrolysate of ClDaf **2** derivatized with FDAA, together with the chromatograms of the FDAA-derivatized L- and D-amino acid standards. The masses ($[M+H]^+$) associated with each peak are indicated in brackets, and peaks associated to residual FDAA indicated by asterisks. Data confirm the presence of D-serine and D-arginine in ClDaf, **2**. In addition, data shows that both D- and L- ornithines are present in ClDaf **2**, but because it contains a total of four ornithine derived amino acids, and because the acid hydrolysis removes ornithine modifications [6,7], the stereochemistry of the individual ornithine-derived amino acids cannot be unequivocally defined.

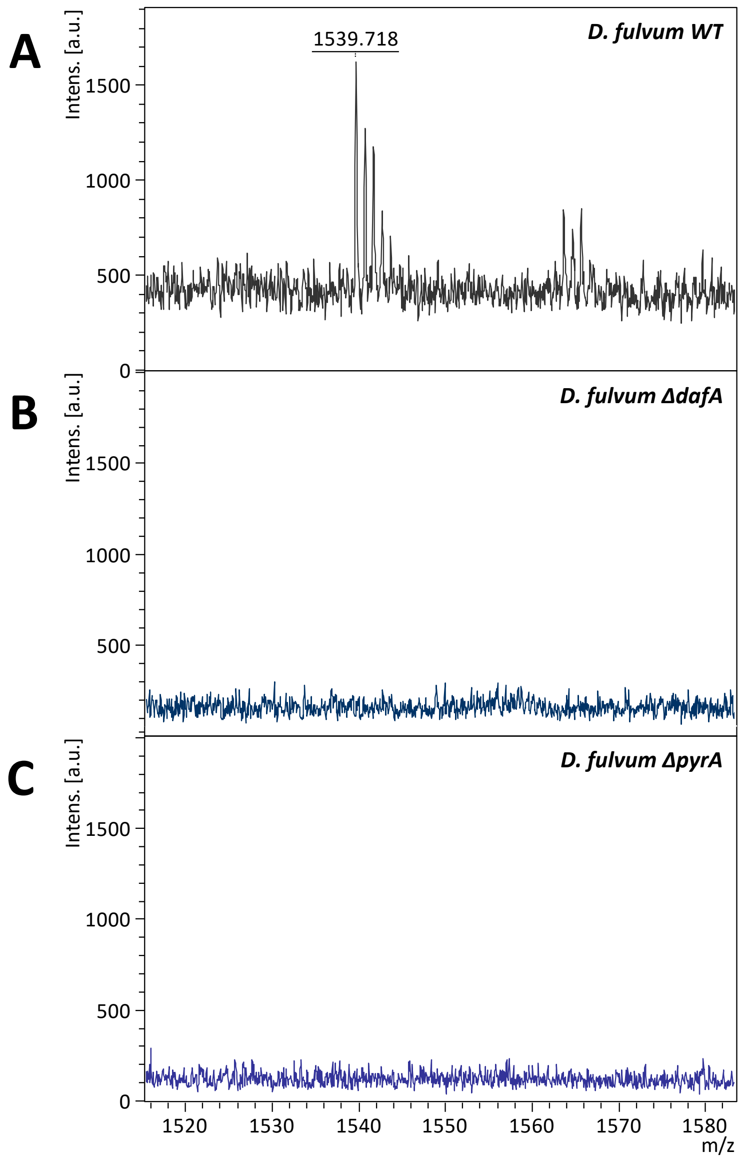

Figure S10. MALDI TOF mass spectrometry spectra (non-HRMS), focused on 1515-1590 region. Data shows the production of low amounts of the ClDaf-Pyr **4** conjugate ($[M+H]^+$= 1539.17) in **A**) *D. fulvum* WT, but not in the knockout mutants **B**) *D. fulvum* ΔpyrA and **C**) *D. fulvum* ΔdafA. The isotopic profile of ClDaf-Pyr **4** agrees to that of a mono-chlorinated compound.

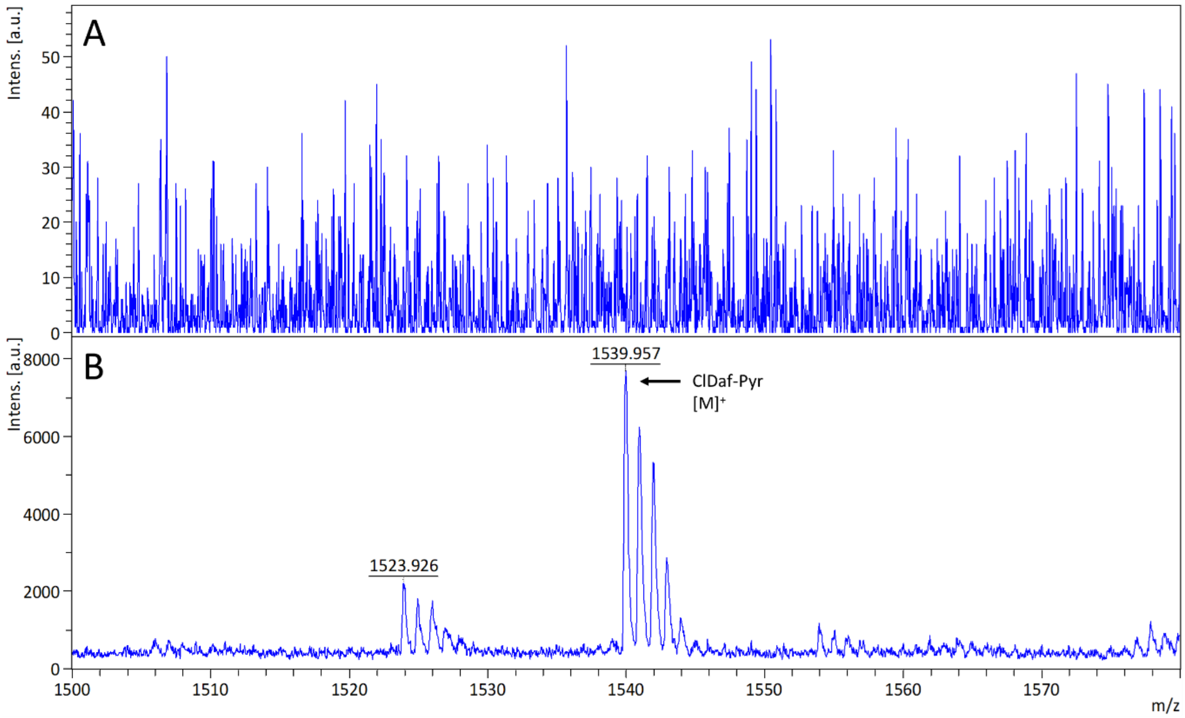

Figure S11. MALDI-TOF analysis (non- HRMS, focused on 1500-1580 Da) of bacterial cell pellet acetonitrile extracts of *D.fulvum* ΔPyrA mutant, cultivated in (**A**) GYM or (**B**) GYM supplemented with 100µM pyridomycin. Supplementation of pyridomycin leads to the formation of a ClDaf-Pyr **4** conjugate with a mass of $[M+H]^+$= 1539.96 with the isotopic profile of a chlorinated compound.

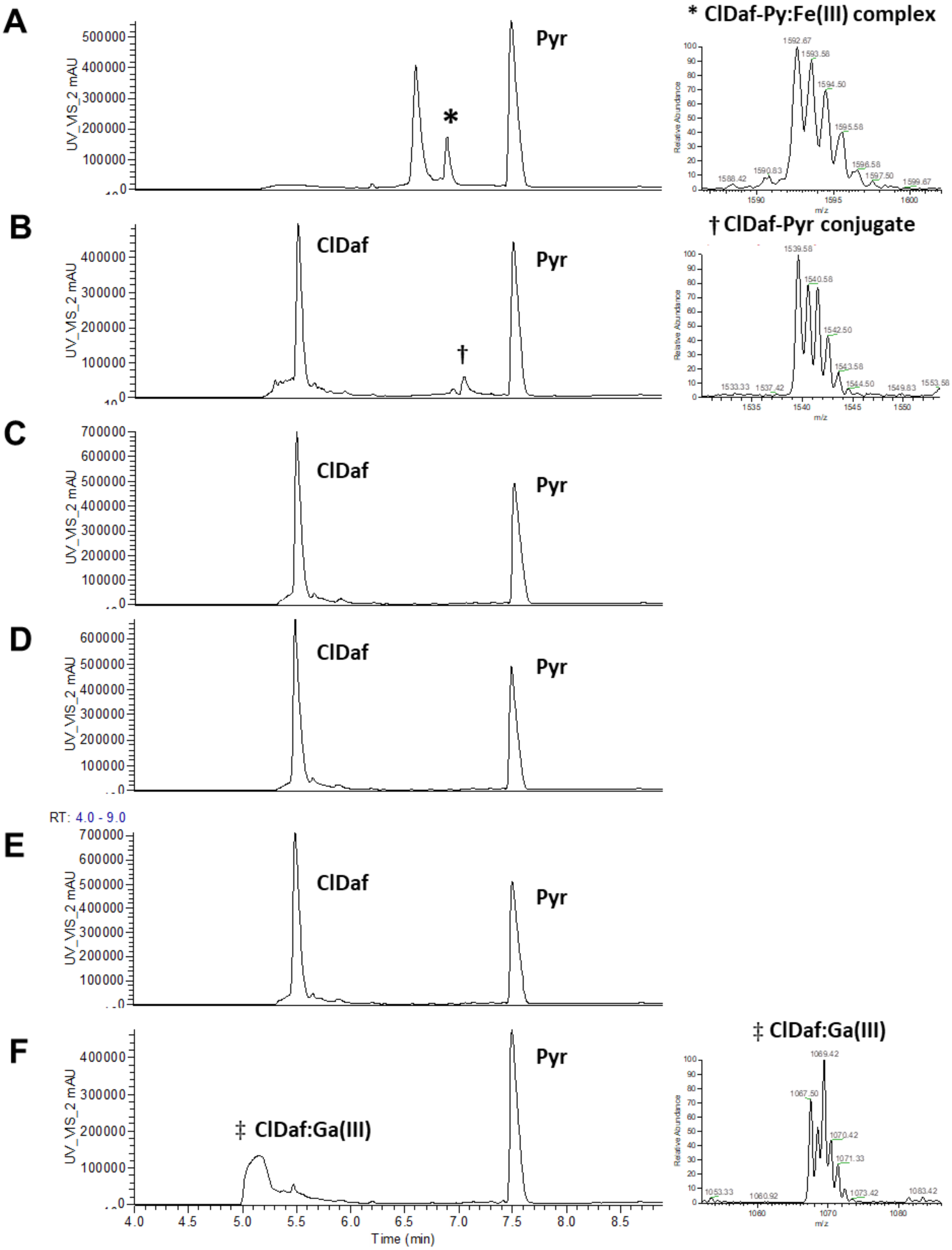

Figure S12. Conjugation of ClDaf **2** and Pyr **1** using 10 equivalents of **A**) $FeCl_3$, **B**) $CuSO_4$, **C**) $FeSO_4$, **D**), $CoCl_2$, **E**) $ZnSO_4$ and **F**) $GaCl_3$. The main chromatograph shows the UV absorbance chromatograph (254 nm) of the reaction mixture, with the peaks corresponding to Pyr **1** and ClDaf **2** indicated. Where appropriate, a specific mass spectrometry spectrum is shown on the right of the **A**) ClDaf-Pyr:Fe(III) **4:iron** complex, **B**) ClDaf-Pyr **4** non-complexed conjugate mass and **F**) ClDaf:Ga(III) **2:gallium** complex that did not undergo conjugation. For the other reactions (**C-E**) no product peaks were detected.

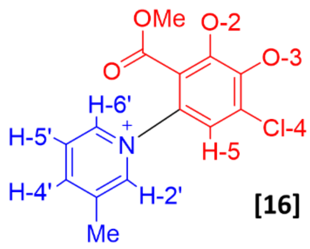

[16]

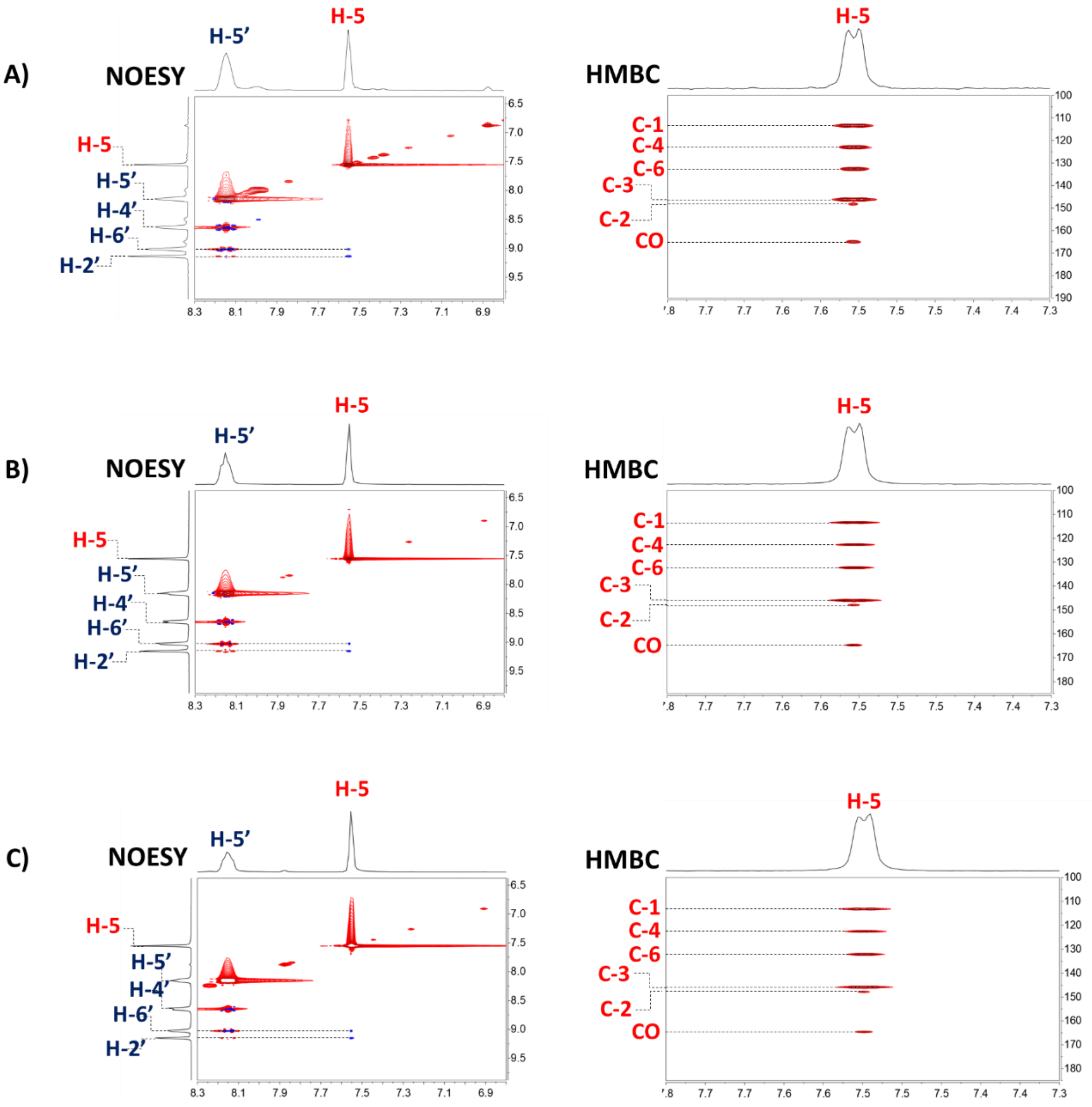

Figure S13. NOESY and ^{13}C-HMBC correlations for the purified conjugate **16** (see structure) of 4-chloro-2,3-dihydroxybenozoate methyl ester **5** with 3-methyl pyridine **12** generated using the following oxidants: **A**) 10 eq. of iron trichloride **B**) molecular iodine **C**) silver-oxide. Data shows the products to share identical regioselective configuration that is in line with that observed for ClDaf-Pyr **4**.

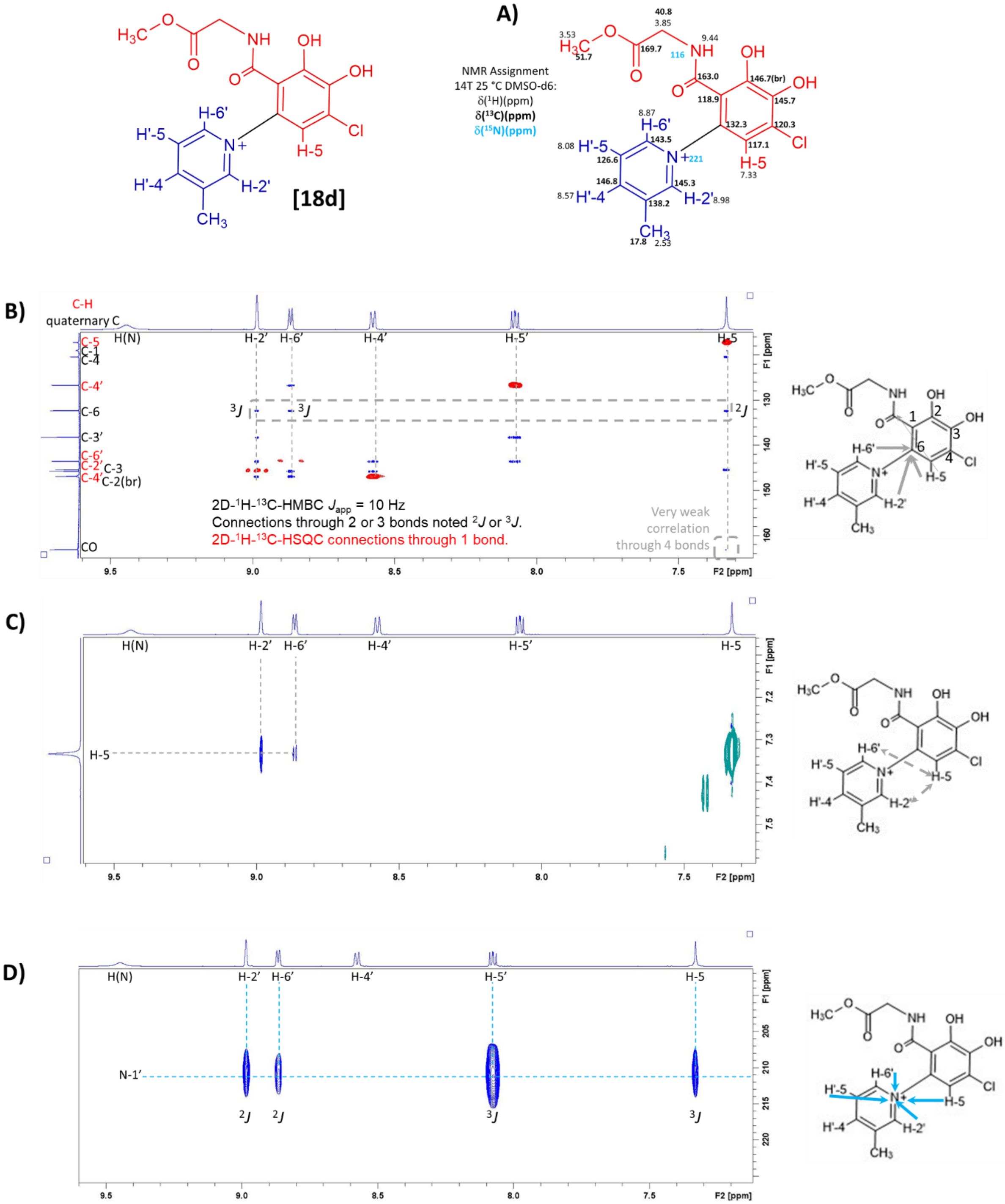

Figure S14. A) NMR assignment, **B**) ^{13}C-HMBC, **C**) ROESY and **D**) ^{15}N-HMBC correlations for the purified conjugate **18d** (see structure) of 4-chloro-2,3-dihydroxybenzoyl methyl glycinate **11** with 3-methyl pyridine **12** generated using the oxidation through electrochemistry. Data shows the product to share an identical regioselective configuration to ClDaf-Pyr **4**.

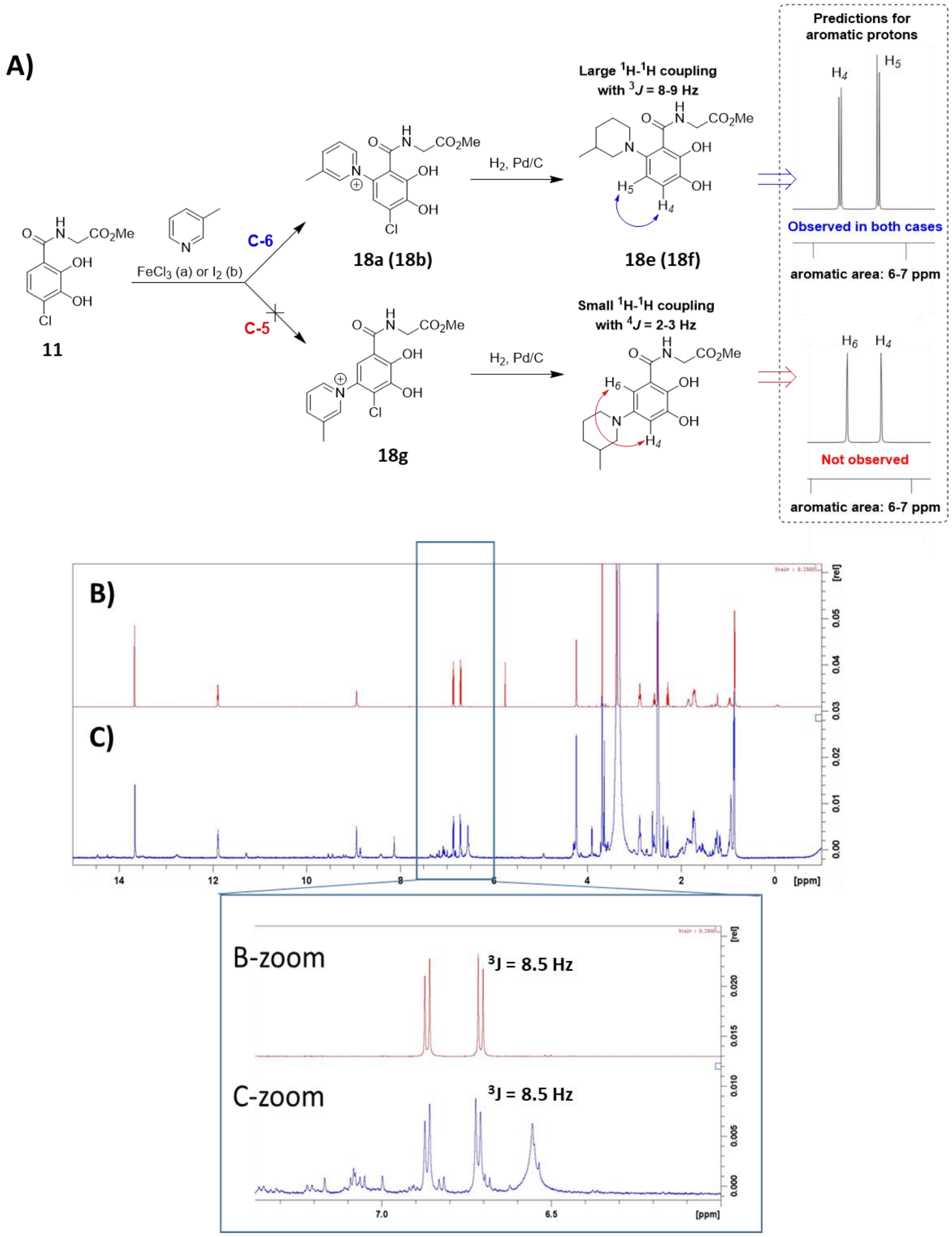

Figure S15. **A)** Schematic showing possible iron and iodine mediated chlorocatechol-pyridine conjugation of comp. **11** with 3-methylpyridine (comp **12**) through C6-N bond formation (top) or C5-N bond formation (bottom). Reductive hydrogenation on palladium of such a conjugate would lead to both dechlorination and pyridine reduction to piperazine. On the right are shown respective predicted ^{1}H-NMR spectra for the C6-N (with a large coupling constant) and C5-N (with a small coupling constant) reduced conjugates. **B)** ^{1}H-NMR data for the reduced product **18f** (from iodine generated **18b**), compared to **C)** the ^{1}H-NMR data for reduced product **18e** (from iron generated **18a**). Zoomed areas of the spectra clearly show two aromatic doublets with a large, 8.5 Hz ^{3}J coupling constant (with some minor signals visible from contaminants).

A

Mol. fragment	Atom. No.	δ H / ppm (600 MHz)	Mult. Hz	δ C / ppm (150 MHz)	δ N / ppm (61 MHz)
ClDHB (1)	1			108.4	
	2	13.68 (OH)		151.4	
	3	8.93 (OH)		143.9	
	4	6.87	d 8.6	118.1	
	5	6.71	d 8.6	111.8	
	6			144.5	
	7			171.1	
Gly (2)	1	4.24	d 5.5	40.7	
	2			170.0	
	Me-3	3.69		52.1	
	1-HN	11.90	t 5.5		110.6
3-methylpiperidine (3)	N-1				54.7
	2_{ax}	2.29	t 10.9	61.7	
	2_{eq}	2.87			
	3_{ax}	1.85		31.1	
	Me-3	0.86	d 6.6	19.4	
	4_{ax}	0.96		31.9	
	4_{eq}	1.72			
	5	1.70		25.4	
	6_{ax}	2.57	t 11.5	54.2	
	6_{eq}	2.89			

B

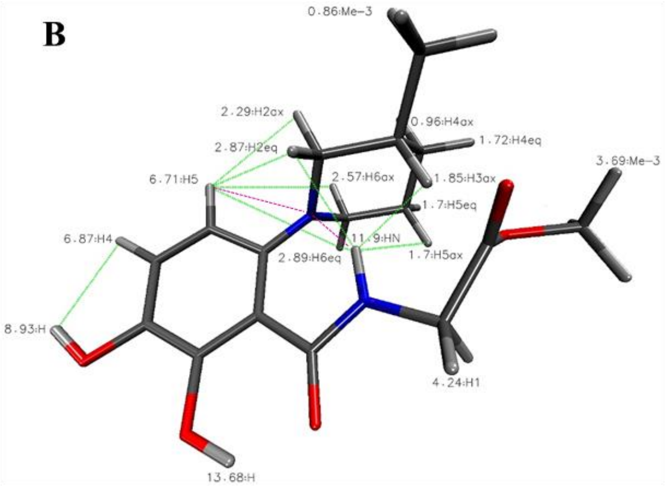

Figure S16. Key evidence by 1D and 2D NMR analysis confirming the structural arrangement of **18f**. **A)** Table of proton, carbon, and nitrogen chemical shifts with the corresponding *J*-coupling values (NMR spectra in the supporting NMR files). **B)** Stylized presentation of 2D NOEs (green dotted lines) and ^1H-^{15}N HMBC (dashed magenta lines) correlations observed, with values indicating proton chemical shifts. Image generated using Visual Molecular Dynamics (VMD) software.

Additional explanation: The observed NOE correlation between the peaks at 6.87 (CH) and 8.93 (OH) ppm clearly indicates that the CH is located close to one phenolic OH, which allows to clearly identify C-3 and C-4. The second CH (6.71 ppm) is then traced to C-5, after which the *N*-binding can be clearly linked to C-6. In the ^1H-^{15}N HMBC, the 6.71 ppm peak (H-5 of DHB) correlates to the piperidine ^{15}N with an equivalent intensity *vs.* the H on the piperidine ring (H-5_{eq} at 1.70 ppm *via* a ^3J, H-2 and H-6 *via* ^2J). The correlation of H=6.71 ppm (DHB) to N=55 ppm is clearly a ^3J and not a ^4J. Thus, this allows to assign the proton signal at 6.71 ppm to H-5 of DHB, which then allows to trace C-6 linked to the N atom. We observe a hydrogen bond between the HN of Gly and N-1 of the piperidine, which leads to a blocked perpendicular conformation of the piperidine ring *vs* the DHB ring. The piperidine ring seems to be in a chair conformation with the DHB moiety at position 1 and methyl at C-3, both equatorial. Each H of the piperidine ring has its own δ(^1H), which allows to clearly distinguish the equatorial and axial protons. In the corresponding 2D NOESY, we observe strong correlations of the HN of Gly to H-2_{eq}, H-6_{eq}, H-3_{ax}, and H-5 of the 3-methylpiperidine fragment, while H-5 of DHB strongly correlates to H-$2_{ax,}$ H-2_{eq}, H-6_{eq}, and H-6_{ax}. This can be easily explained by the respective 3D model (see **Fig. 16b**). The HN and CH_2 of Gly clearly shows correlations to N-1 of piperidine in the ^1H-^{15}N HMBC through ^2J and ^4J, which can be explained only by the hydrogen-bond formation.

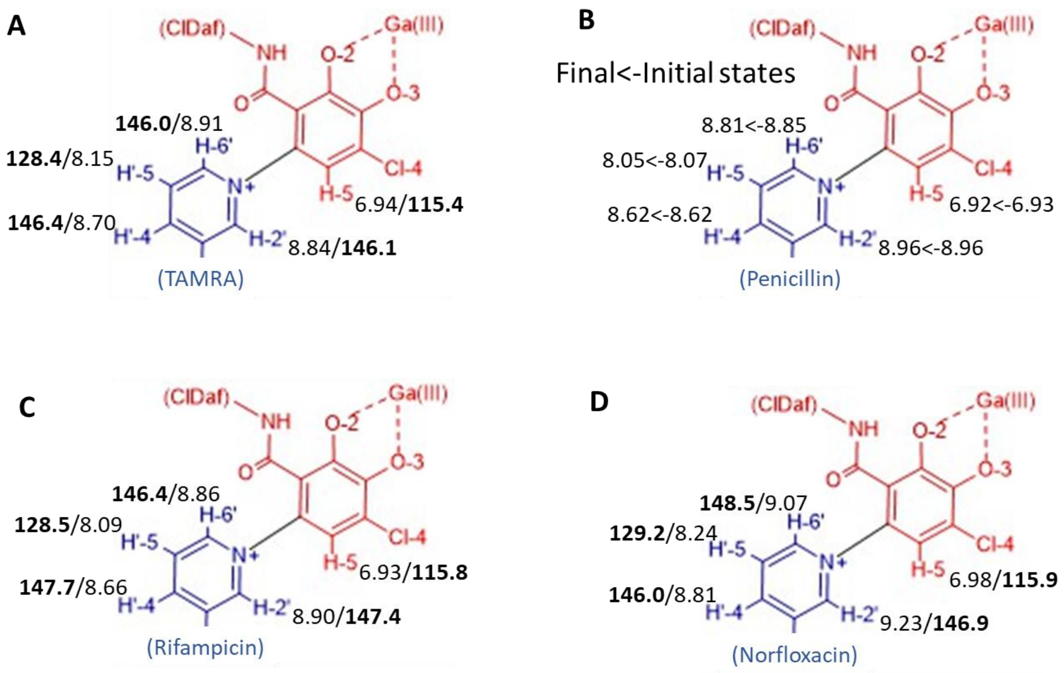

Figure S17: Partial ^{1}H and ^{13}C chemical shifts assignments in CD_3OD at 14 T and 293 K of the large conjugates **A,** ClDaf-3-pyridyl-TAMRA, **22**, **B,** ClDaf-3-pyridyl-penicillin, **24**, **C,** ClDaf-3-pyridyl-rifampicin, **26**, and **D,** ClDaf-3-pyridyl-norfloxacin, **28**. Due to the chemical instability of ClDaf-3-pyridyl-penicillin **24** (due to lactam hydrolysis), values on the right represent the starting state, and the left values for the final state.

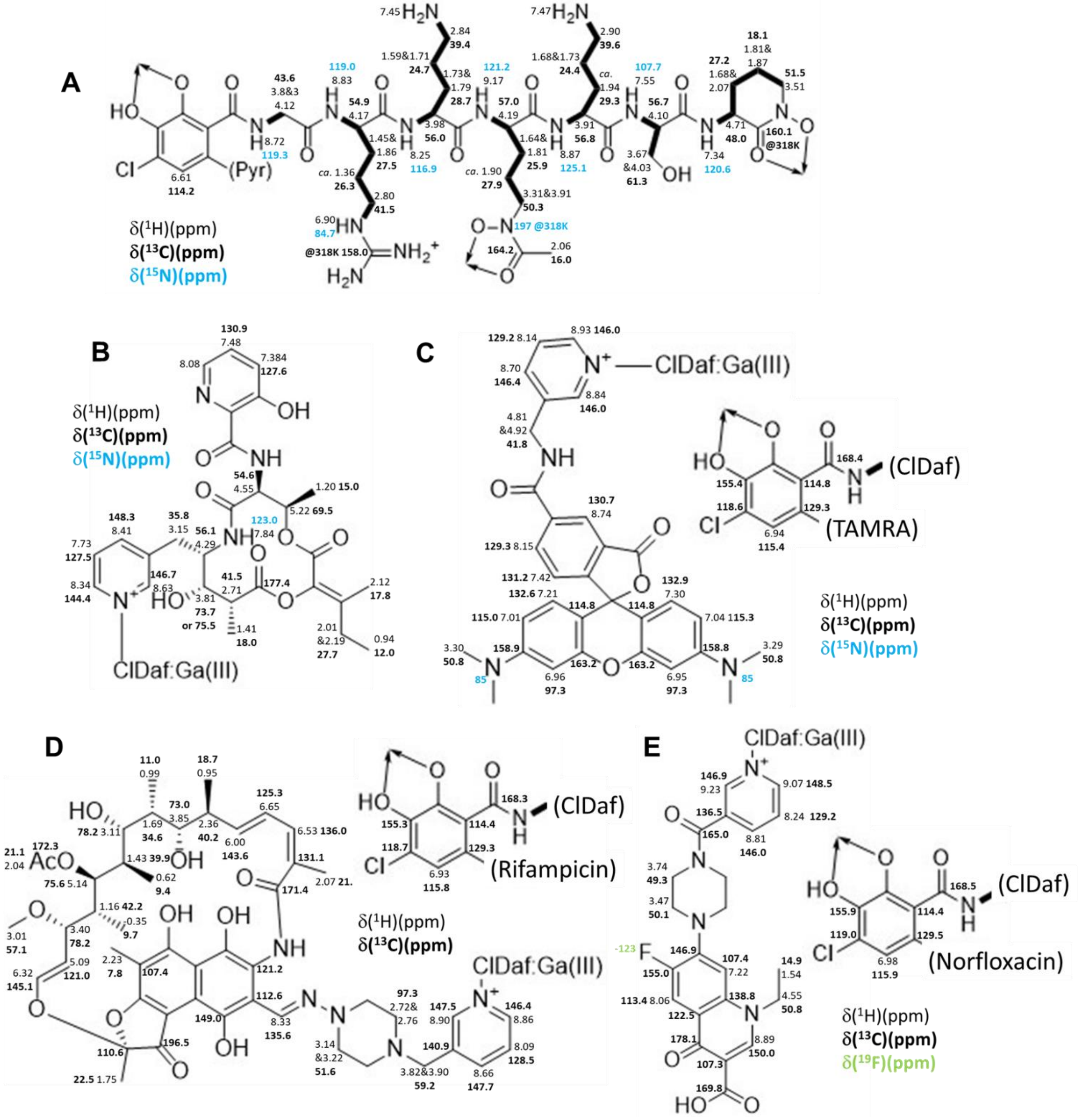

Figure S18: Partial ^{1}H/^{13}C/^{15}N/^{19}F chemical shifts assignments of gallium complexed Chlorodactyloferrin conjugates at 293 K and 14 T. **A)** The chlorodactyloferrin half, and **B**) the pyridomycin half of ClDaf-pyridomycin gallium complex, **4:gallium**, in ACN-d3/water 1/1 v/v **C**) the 3-pyridyl-TAMRA half of ClDaf-3-pyridyl-TAMRA gallium complex, **22:gallium**, in CD_3OD. **D**) The 3-pyridyl-rifampicin part of ClDaf-3-pyridyl-rifampicin gallium complex, **26:gallium**. in CD_3OD **E**) the 3-pyridyl-norfloxacin part of ClDaf-3-pyridyl-norfloxacin gallium complex, **28:gallium** in CD_3OD.

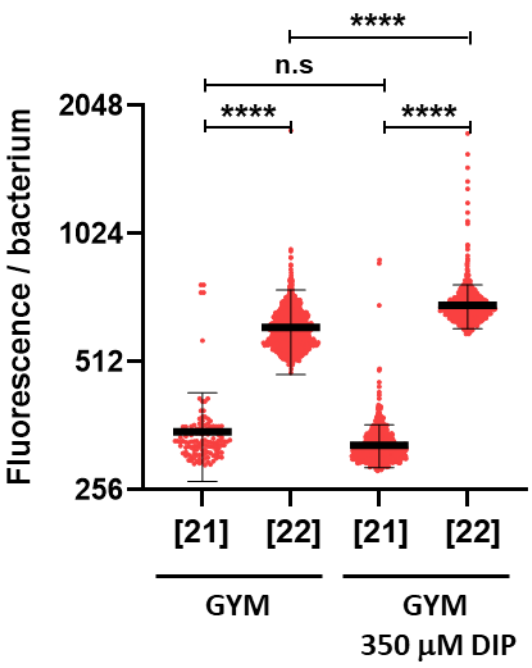

Figure S19: Quantification of 3-pyridyl-TAMRA **21** or ClDaf-3-pyridyl-TAMRA **22:iron** uptake by wild-type *D. fulvum* incubated for 4 h in GYM with or without 350 μM DIP as measured by the fluorescence intensity per bacterium in confocal images. Black lines and errors indicate mean ± SD. Asterisks denote significance by Kruskal–Wallis and Dunn's multiple comparison test: ****$P < 0.0001$. The data shown are from at least three independent images (n > 200 cells per condition). As shown, ClDaf-3-pyridyl-TAMRA **22:iron** is taken up by *D. fulvum* in both iron rich and poor conditions, with only slightly higher uptake in the latter condition (1.1 fold greater uptake). This data is in line with the constitutive expression of the ClDaf BGC (**Fig. S5**).

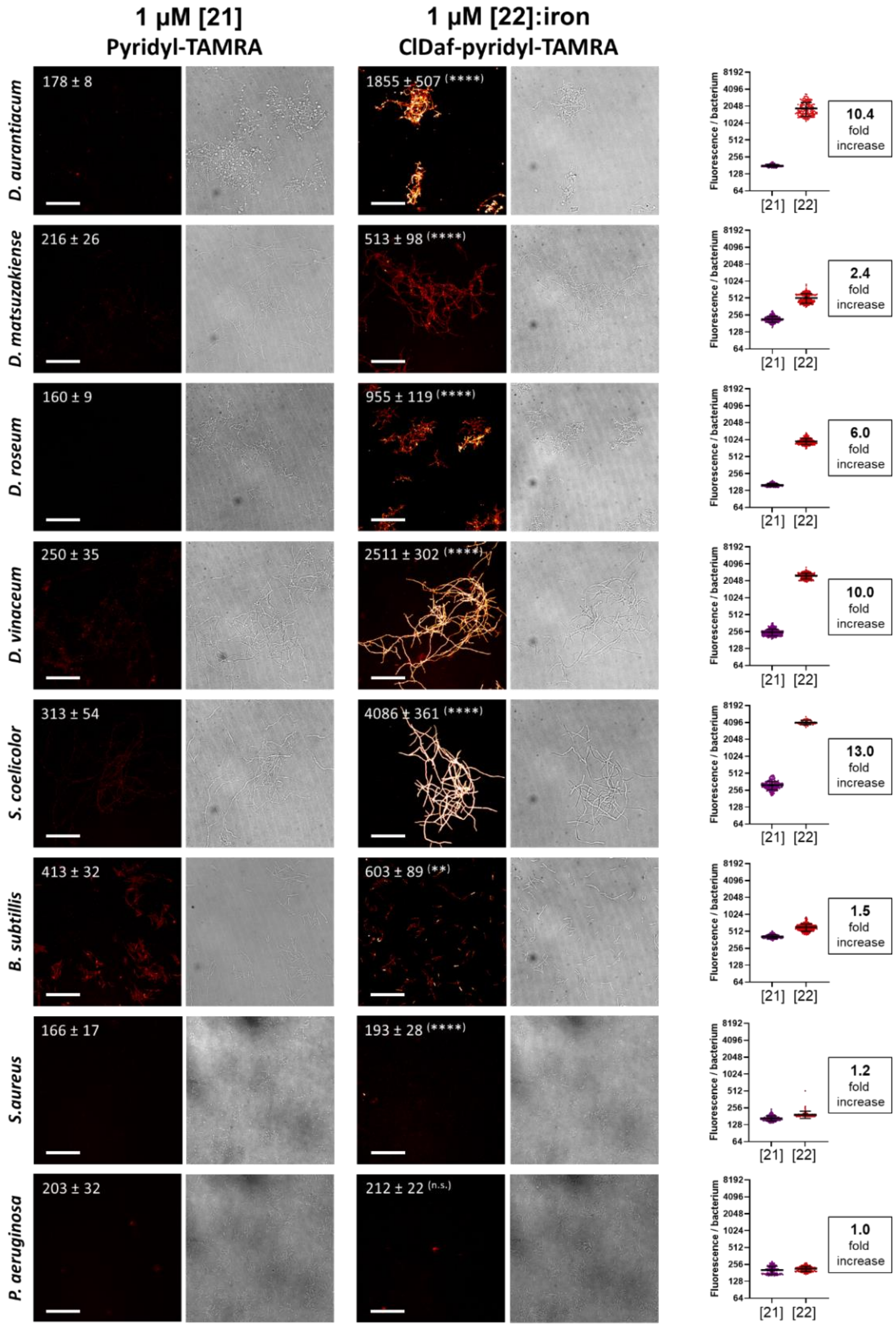

Figure S20. Representative fluorescence and phase contrast confocal images of different bacterial strains incubated in GYM supplemented with 350 µM DIP (for *Dactylosporangium* strains and *S. coelicolor*) or CAMHB with 300 µM DIP (for *E. coli*, *P. aeruginosa* and *S. aureus*) in presence of 3-pyridyl-TAMRA **21** (left images) and ClDaf-3-pyridyl-TAMRA **22:iron** (right images). Quantification of the fluorescence intensity per bacterium (mean ± SD) is indicated in the fluorescence image for each condition, as well as in the adjacent graph. Confocal microscopy images are representative for the specified condition, and the quantification data comes from the analysis of at least three independent images (*n* > 200 cells per condition). In the graphs black lines and errors indicate mean ± SD. Asterisks denote significance by Kruskal–Wallis and Dunn's multiple comparison test: n.s., not significant; **P = 0.002; ****P < 0.0001.

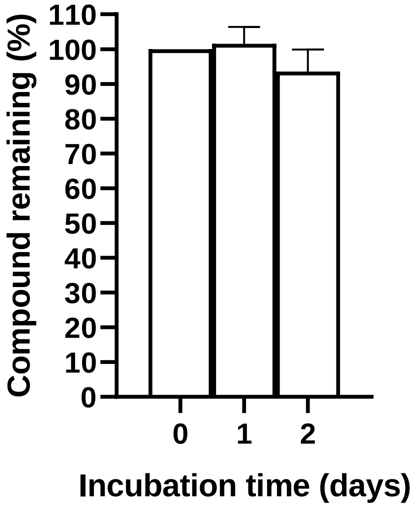

Figure S21. Stability of ClDaf-pyridyl-rifampicin **26** in bacteria free, “spent” media (*D. fulvum* culture filtrate). ClDaf-pyridyl-rifampicin **26** was spiked into “spent” media (*D. fulvum* culture filtrate obtained from a well-developed culture) and incubated at 30°C. To determine conjugate stability, samples were taken at different time points (0, 1 and 2 days), and analyzed by analytical UHPLC-MS, as described in the methods section. The amount of ClDaf-pyridyl-rifampicin **26** was quantified using the area under de peak, and presented as a percentage area compared to t=0 (paired). Data shows the mean ± SD of 3 biological replicate data.

References:

(1) Huang, T.; Wang, Y.; Yin, J.; Du, Y.; Tao, M.; Xu, J.; Chen, W.; Lin, S.; Deng, Z. Identification and Characterization of the Pyridomycin Biosynthetic Gene Cluster of Streptomyces Pyridomyceticus NRRL B-2517. *J. Biol. Chem.* **2011**, *286* (23), 20648–20657. https://doi.org/10.1074/jbc.M110.180000.

(2) Caradec, T.; Trivelli, X.; Desmecht, E.; Peucelle, V.; Khalife, J.; Hartkoorn, R. C. Dactylosporolides: Glycosylated Macrolides from Dactylosporangium Fulvum. *J. Nat. Prod.* **2022**. https://doi.org/10.1021/acs.jnatprod.2c00484.

(3) Ducret, A.; Quardokus, E. M.; Brun, Y. V. MicrobeJ, a Tool for High Throughput Bacterial Cell Detection and Quantitative Analysis. *Nat. Microbiol.* **2016**, *1* (7), 1–7. https://doi.org/10.1038/nmicrobiol.2016.77.

(4) Schindelin, J.; Arganda-Carreras, I.; Frise, E.; Kaynig, V.; Longair, M.; Pietzsch, T.; Preibisch, S.; Rueden, C.; Saalfeld, S.; Schmid, B.; et al. Fiji: An Open-Source Platform for Biological-Image Analysis. *Nat. Methods* **2012**, *9* (7), 676–682. https://doi.org/10.1038/nmeth.2019.

(5) Hartkoorn, R. C.; Sala, C.; Neres, J.; Pojer, F.; Magnet, S.; Mukherjee, R.; Uplekar, S.; Boy-Röttger, S.; Altmann, K. H.; Cole, S. T. Towards a New Tuberculosis Drug: Pyridomycin - Nature's Isoniazid. *EMBO Mol. Med.* **2012**, *4* (10), 1032–1042. https://doi.org/10.1002/emmm.201201689.

(6) Li, Y.; Liu, L.; Zhang, G.; He, N.; Guo, W.; Hong, B.; Xie, Y. Potashchelins, a Suite of Lipid Siderophores Bearing Both L-Threo and L-Erythro Beta-Hydroxyaspartic Acids, Acquired From the Potash-Salt-Ore-Derived Extremophile Halomonas Sp. MG34. *Front. Chem.* **2020**, *8* (March). https://doi.org/10.3389/fchem.2020.00197.

(7) Vind, K.; Brunati, C.; Simone, M.; Sosio, M.; Donadio, S.; Iorio, M. Megalochelin, a Tridecapeptide Siderophore from a Talented Streptomycete. *ACS Chem. Biol.* **2023**, *18* (4), 861–874. https://doi.org/10.1021/acschembio.2c00958.

(8) Marfey, P. Determination of D-amino acids. II. Use of a bifunctional reagent, 1,5-difluoro-2,4-dinitrobenzene. *Carlsb. Res. Commun.* **1984**, *49*, 591–596.

(9) Rutherford, K.; Parkhill, J.; Crook, J.; Horsnell, T.; Rice, P.; Rajandream, M.-A.; Barrell, B. Artemis: Sequence Visualization and Annotation. *Bioinformatics* **2000**, *16* (10), 944–945. https://doi.org/10.1093/bioinformatics/16.10.944.
