# Supplementary material for: A Novel Natural Siderophore Antibiotic Conjugate Reveals a Chemical Approach to Macromolecule Coupling
Source: ACS Cent Sci. 2023 Nov 10;9(11):2138–49. doi: 10.1021/acscentsci.3c00965 (PMC10683483; doi:10.1021/acscentsci.3c00965)
Supplement: Supplementary file 2 — oc3c00965_si_002.pdf [file oc3c00965_si_002.pdf]

Supplementary data

Synthetic and Analytical Chemistry

A novel natural siderophore antibiotic conjugate reveals a chemical approach to macromolecule coupling

Thibault CARADEC[1+], Ernesto ANOZ-CARBONELL[1+], Ravil PETROV[1+], Muriel BILLAMBOZ[2,3], Kevin ANTRAYGUES[4], Francois-Xavier CANTRELLE[2,5], Emmanuelle BOLL[2,5], Delphine BEURY[6], David HOT[6], Herve DROBECQ[1], Xavier TRIVELLI[7], Ruben C. HARTKOORN[1*]

[+] Joint first authorship (contributed equally)
** Corresponding Author, Ruben C. Hartkoorn: Email: ruben.hartkoorn@inserm.fr*

[1] Univ. Lille, CNRS, Inserm, CHU Lille, Institut Pasteur Lille, U1019 - UMR 9017 - CIIL - Center for Infection and Immunity of Lille, F-59000 Lille, France.
[2] Univ. Lille, Inserm, CHU Lille, Institut Pasteur de Lille, U1167 - RID-AGE - Risk Factors and Molecular Determinants of Aging-Related Diseases, F-59000 Lille, France.
[3] JUNIA, Health and Environment, Laboratory of Sustainable Chemistry and Health, Lille, F-59000, France
[4] Univ. Lille, Inserm, Institut Pasteur de Lille, U1177 - Drugs and Molecules for Living Systems, F-59000, Lille, France.
[5] CNRS, EMR9002 BSI Integrative Structural Biology, 59000 Lille,
[6] Univ. Lille, CNRS, Inserm, CHU Lille, Institut Pasteur de Lille, UMR2014 - US41 - PLBS-Plateformes Lilloises de Biologie & Santé, F-59000, Lille, France.
[7] Univ. Lille, CNRS, INRAE, Centrale Lille, Univ. Artois, FR 2638 - IMEC - Institut Michel-Eugène Chevreul, 59000, Lille, France.

Table of Contents

Synthetic and Analytical Chemistry **S4**

Materials and methods **S4**

Analytical mass spectrometry (UHPLC-MS) **S4**

High-resolution Mass spectrometry **S4**

HPLC purification **S4**

Comp. 1 (Pyridomycin) **S5**

Comp. 2 (Chlorodactyloferrin in its free, iron-, and gallium-complexed forms) **S5**

Comp. 3 (Dactyloferrin in its free, iron-, and gallium-complexed forms) **S6**

Comp. 4 (conjugate of pyridomycin 1 and chlorodactyloferrin 2 in its free, iron-, and gallium-complexed forms) **S6**

Comp. 5 (4-chloro-2,3-dihydroxybenzoate methyl ester) **S7**

Comp. 6 (2,3-dihydroxybenzoate methyl ester) **S8**

Comp. 7 (4-chloro-2,3-dimethoxybenzoate methyl ester) **S8**

Comp. 11 (4-chloro-2,3-dihydroxybenzoyl-glycinate methyl ester) **S8**

Comp. 16 (conjugate of 5 and 12 generated by oxidation to give compounds 16a/16b/16c) **S9**

Comp. 16a ($FeCl_3$ oxidation) *S9*

Comp. 16b (iodine oxidation) *S9*

Comp. 16c (silver oxide oxidation) *S10*

Comp. 17 (conjugate of 6 and 12 generated by oxidation to give compounds 17a and 17b) **S10**

Comp. 17a ($FeCl_3$ oxidation) *S10*

Comp. 17b (iodine oxidation) *S10*

Comp. 18 (conjugate of 11 and 12 generated by oxidation to give compounds 18a/18b/18d) **S11**

Comp. 18a ($FeCl_3$ oxidation) *S11*

Comp. 18b (iodine oxidation) *S11*

Comp. 18d (electrochemical oxidation) *S12*

Comp. 18e (reduction of 18a obtained by oxidation with $FeCl_3$) *S12*

Comp. 18f (reduction of 18b obtained by oxidation with iodine) *S13*

Comp. 19 (conjugate of 5 and 13 generated by oxidation to give compounds 19a/19b/19c) **S13**

Comp. 19a ($FeCl_3$ oxidation) *S13*

Comp. 19b (iodine oxidation) *S13*

Comp. 19c (silver oxide oxidation) *S14*

Comp. 20 (conjugate of 5 and 14 generated by oxidation to give compounds 20a/20b/20c) **S14**

Comp. 20a ($FeCl_3$ oxidation) *S14*

Comp. 20b (iodine oxidation) *S15*

Comp. 20c (silver oxide oxidation) *S15*

Comp. 21 (3-pyridyl-TAMRA) **S15**

Comp. 22 (conjugate of 21 and Chlorodactyloferrin [2] in its iron- and gallium-complexed forms) **S16**

Comp. 23 (3-pyridyl-penicillin) **S17**

Comp. 23-1 (Compound 23 intermediate 1) *S17*

Comp. 23-2 (Compound 23 intermediate 2) *S17*

Comp. 23 (3-pyridyl-penicillin) *S18*

Comp. 24 (conjugate of 23 and chlorodactyloferrin [2] in its iron- and gallium-complexed forms)..... S18
Comp. 25 (3-pyridyl-rifampicin) S19
Comp. 26 (conjugate of 25 and chlorodactyloferrin [2] in its iron- and gallium-complexed forms)..... S19
Comp. 27 (3-pyridyl-norfloxacin) S20
Comp. 28 (conjugate of 27 and chlorodactyloferrin [2] in its iron-, and gallium-complexed forms).... S20
References S21

Synthetic and Analytical Chemistry

Materials and methods

Reagents and solvents were obtained from commercial sources and used without further purification. Comp. **12** (3-methylpyridine, CAS 108-99-6), **13** (4-methylpyridine, CAS 108-89-4), **14** (2-methylpyridine, CAS 109-06-8), and **15** (2,6-dimethylpyridine, CAS 108-48-5) were obtained from AcrosOrganics (pur. 99%). 5-Carboxytetramethylrhodamine (5-TAMRA, CAS 91809-66-4, pur. >95%), comp. **8** (2,3-dimethoxybenzoate methyl ester, CAS 2150-42-7, pur. 97%), and 3-pyridylacetic acid hydrochloride (CAS 6419-36-9, pur. 98%) were obtained from Sigma-Aldrich. Comp. **9** (3-methoxysalicylate methyl ester, CAS 6342-70-7) and **10** (2,4-dimethoxybenzoate methyl ester, CAS 2150-47-2) were obtained from TCI (pur. >98%). 6-Aminopenicillanic acid (CAS 551-16-6, pur. 96%) was obtained from FischerScientific and used as received. 3-Formyl rifamycin (CAS: 13292-22-3) was purchased from Biosynth Ltd (UK).

The progress of reactions was routinely monitored by thin layer chromatography (TLC) using Merck commercial aluminum sheets coated with silica gel 60 F254. Visualization was achieved by monitoring fluorescence under UV light at 254 and 365 nm. Flash column chromatography was performed using prepacked silica gel cartridges (SiO_2, 30-50 µm average diameter) and Interchim PuriFlash XS 420 system. UV detection at 250 nm and 280 nm was used to direct collections of the relevant fractions.

NMR spectra were recorded on Bruker Avance III300 spectrometer equipped with a 5 mm BBO (X-^{1}H) probe or a Bruker Avance IIIHD 600 equipped with a 5 mm cryogenic QCI (^{1}H/^{2}H/^{13}C/^{15}N/^{19}F) probe. The ^{1}H and ^{13}C spectra were referenced to the signals of residual organic solvents as internal references, to the DSS trimethylsilyl signal (0.00 ppm) for aqueous solutions and to the residual TFA signal for ^{19}F spectrum of **28**. Indirect referencing was used for ^{15}N. Chemical shifts (δ) are in parts per million (ppm) downfield from tetramethylsilane (TMS). NMR coupling constants (*J*) are reported in Hertz (Hz), and splitting patterns are indicated as follows: s (singlet), br (broad singlet), d (doublet), dd (doublet of doublets), m (multiplet). Yields refer to chromatographically pure compounds as determined by TLC (single spot) or HPLC. Electrosynthesis was carried out on IKA ElectraSyn 2.0 potentiostat using the following conditions: 0.1 m NaOAc in DI water/acetonitrile (1:1, v/v), Ag/Ag+ as reference electrodes, C-glassy working electrode, and C-glassy counter electrode. Deionized water was prepared with a Milli-Q water purification system to a resistivity of 17 MΩ·cm.

Analytical mass spectrometry (UHPLC-MS)

Analytical (non-high resolution) spectrometry analysis (UHPLC-MS) were performed on an Ultimate 3000 UHPLC system, coupled with a LCQ Fleet Ion Trap Mass Spectrometer (Thermo Scientific). Chromatographic separation was achieved using an Acquity UPLC Peptide BEH C18-column (300Å, 1.7 µm, 2.1 mm x 100 mm). Mobile phase system was composed of solvent A (H_2O, 0.1% formic acid) and B (acetonitrile, 0.1% formic acid), typically run through a linear gradient from 0% to 100% of solvent B in 10 min. Elution of compounds was monitored by UV absorbance at 215 nm and 254 nm, and by mass spectrometry electrospray ionization.

High-resolution Mass spectrometry

High resolution mass spectrometry of purified compounds was performed by the ARIADNE-ADME platform (Institut Pasteur de Lille, France), using a quadrupole time-of-flight (TOF) LCT Premier XE mass spectrometry machine (Waters).

HPLC purification

Compounds were purified with a Semi-preparative RP-HPLC systems PLC 2020 (Gilson) using either a semipreparative XBridge™ Peptide BEH C18-column (10 x 250 mm, 130 Å, 5 micron) or preparative XBridge™ Peptide BEH C18-OBDTM column (19 x 150 mm, 130 Å, 5 micron) from Waters. Compounds were eluted using the same mobile phase system as described above for UHPLC-MS. The corresponding fractions were assayed by analytical UHPLC-MS to determine the molecular weights and purities, and fractions with the desired characteristics were pooled together. Acetonitrile was evaporated and the aqueous solution was lyophilized to obtain the compound of interest.

Comp. 1 (Pyridomycin)

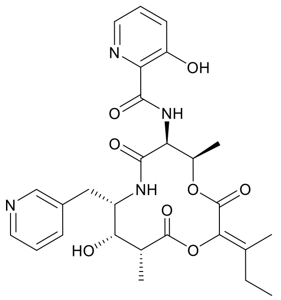

Pyridomycin was produced by and purified from *Dactylosporangium fulvum* (NRRL B-16292) with a yield of 20–40 mg/L and purity >99%. NMR spectra are consistent with the previous literature.[1]

^{1}H NMR (600 MHz, CD_3CN/water, 1/1 v/v, 293 K): δ 8.31 (br, 1H), 8.09 (d, ^{3}J = 4.5 Hz, 1H), 8.00 (d, ^{3}J = 4.3 Hz, 1H), 7.58 (d, ^{3}J = 7.6 Hz, 1H), 7.51 (dd, ^{3}J = 4.3&8.5 Hz, 1H), 7.41 (br, 1H), 7.00 (dd, ^{3}J = 4.3&7.6 Hz, 1H), 5.18(m, 1H), 4.60 (d, ^{3}J = 6.3 Hz, 1H), 4.07 (br, 1H), 3.72 (br, 1H), 2.89 (m, 2H), 2.65 (q, ^{3}J = 7.2 Hz, 1H), 2.18 (m, 1H), 2.11 (s, 3H), 2.02 (m, 1H), 1.40 (d, ^{3}J = 7.1 Hz, 3H), 1.21 (d, ^{3}J = 6.4 Hz, 3H), 0.93 (t, ^{3}J = 7.6 Hz, 3H).
^{13}C{^{1}H} NMR (151 MHz, CD_3CN/water, 1/1 v/v, 293 K): δ 177.4, 169.7, 168.3, 161.5, 158.8, 150.2, 149.3, 147.0, 139.5, 135.5, 132.1, 130.8, 130.7, 128.6, 124.5, 75.0, 69.6, 56.8, 54.1, 41.4, 35.7, 27.5, 17.9, 17.6, 14.9, 11.8.
^{15}N{^{1}H} NMR (61 MHz, CD_3CN/water, 1/1 v/v, 293 K): δ 295, 291, 123.

HRMS for **1:** formula: $C_{27}H_{33}N_4O_8+$, calculated: 541.2298, found: 541.2292.

Comp. 2 (Chlorodactyloferrin in its free, iron-, and gallium-complexed forms)

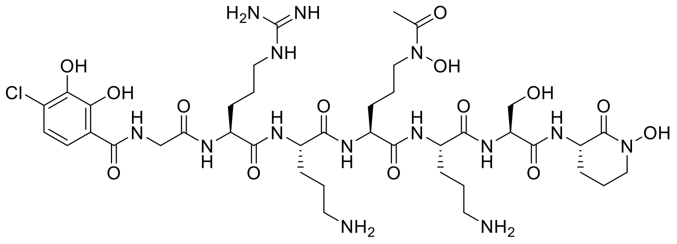

Chlorodactyloferrin [**2**] was produced and purified from cultures of *D. fulvum* as described in the materials and methods section.

NMR Comp **2**

^{1}H NMR (600 MHz, H_2O/D_2O, 293 K): δ 9.09 (br, 1H), 8.57 (d, ^{3}J = 6.2 Hz, 1H), 8.49 (d, ^{3}J = 7.0 Hz, 1H), 8.48 (d, ^{3}J = 8.1 Hz, 1H), 8.40 (d, ^{3}J = 6.6 Hz, 1H), 8.34 (d, ^{3}J = 7.1 Hz, 1H), 8.25 (d, ^{3}J = 6.5 Hz, 1H), 7.64 (br, 4H), 7.29 (d, ^{3}J = 8.8 Hz, 1H), 7.19 (t, ^{3}J = 5.3 Hz, 2H), 7.01 (d, ^{3}J = 8.8 Hz, 1H), 6.7 (br, 4H), 4.49 (m, 1H), 4.43 (m, 1H), 4.36 (m, 1H), 4.34 (m, 1H), 4.31 (m, 1H), 4.29 (m, 1H), 4.16, (m, 1H), 4.11 (m, 1H), 3.82 (m, 2H), 3.68 (m, 1H), 3.62 (m, 1H), 3.51 (m, 2H), 3.20 (m, 2H), 3.01 (t, ^{3}J = 7.5 Hz, 2H), 2.97 (t, ^{3}J = 7.4 Hz, 2H), 2.10 (s, 2.2H), 2.04 (m, 1H), 2.03 (s, 0.8H), 2.01 (m, 1H), 1.87-1.96 (m, 3H), 1.58-1.79 (m, 15H).
^{13}C{^{1}H} NMR (151 MHz, H_2O/D_2O, 293 K): δ 176.9, 176.5, 176.4, 176.1, 175.8, 174.7, 173.7, 173.0, 172.1, 169.3, 159.3, 151.5, 144.0, 128.2, 122.0, 122.2, 117.4, 63.9, 58.1, 56.7, 56.3, 56.2, 56.1, 54.4, 53.1, 49.8, 45.6, 43.2, 41.9, 41.8, 30.7, 30.6, 30.5, 30.5, 29.4, 27.3, 26.1, 25.9, 25.1, 22.7, 22.3, 22.0.
^{15}N{^{1}H} NMR (61 MHz, H_2O/D_2O, 293 K): δ 180, 178, 124.1, 122.0, 121.2, 120.8, 120.2, 117.7, 109.3, 84.7.

NMR Comp **2:gallium**

^{1}H NMR (600 MHz, H_2O/D_2O, 293 K): δ 9.30 (d, ^{3}J = 7.7 Hz, 1H), 9.25 (m, 1H), 9.24 (m, 1H), 8.85 (d, ^{3}J = 6.6 Hz, 1H), 8.74 (m, 1H), 8.00 (d, ^{3}J = 5.3 Hz, 1H), 7.64 (br, 4H), 7.47 (d, ^{3}J = 8.6 Hz, 1H), 6.70 (d, ^{3}J = 8.6 Hz, 1H), 6.74 (t, ^{3}J = 4.0 Hz, 2H), 6.63 (d, ^{3}J = 8.6 Hz, 1H), 6.5 (br, 4H), 4.79 (m, 1H), 4.50 (m, 1H), 4.38 (m, 1H), 4.28 (m, 1H),

4.23 (m, 1H), 4.21 (m, 1H), 4.11 (m, 1H), 4.08 (m, 1H), 4.05 (m, 1H), 3.86 (m, 1H), 3.81 (m, 1H), 3.66 (m, 1H), 3.60 (m, 1H), 3.39 (m, 1H), 3.04 (m, 2H), 2.99 (m, 1H), 2.96 (m, 2H), 2.93 (m, 1H), 2.18 (s, 3H), 1.87-2.10 (m, 11H), 1.76-1.81 (m, 3H), 1.70 (m, 1H), 1.60-1.62 (m, 3H), 1.52 (m, 1H).
$^{13}C\{^{1}H\}$ NMR (151 MHz, H_2O/D_2O, 293 K): δ 179.5, 178.2, 177.6, 176.6, 176.4, 175.0, 173.8, 165.8, 161.4, 159.1, 155.5, 151.6, 122.9, 120.3, 119.5, 117.2, 63.0, 58.9, 58.8, 58.7, 57.6, 57.4, 53.2, 52.3, 50.1, 45.6, 43.5, 41.4, 41.2, 30.3, 29.5, 29.3, 29.3, 28.8, 28.1, 27.6, 26.5, 26.3, 20.1, 18.3.
$^{15}N\{^{1}H\}$ NMR (61 MHz, H_2O/D_2O, 293 K): δ 203, 197, 126.7, 122.1, 121.6, 120.9, 118.2, 115.5, 109.5, 84.1, 32.5, 32.5.

HRMS for **2:** formula: $C_{40}H_{66}N_{14}O_{14}Cl^+$, calculated: 1001.4571, found: 1001.4580.

HRMS for **2:iron:** formula: $C_{40}H_{63}N_{14}O_{14}ClFe^+$, calculated: 1054.3686, found: 1054.3707.

Comp. 3 (Dactyloferrin in its free, iron-, and gallium-complexed forms)

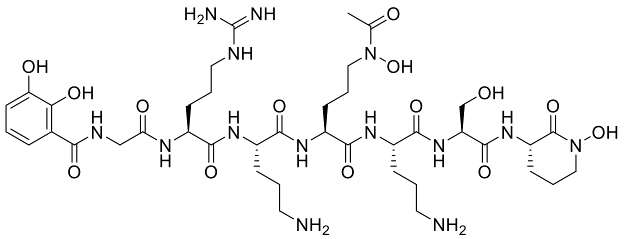

Dactyloferrin [**3**] was produced and purified from as described above.

NMR Comp **3**

^{1}H NMR (600 MHz, H_2O/D_2O, 293 K): δ 9.03 (t, ^{3}J = 5.1 Hz, 1H), 8.58 (d, ^{3}J = 6.3 Hz, 1H), 8.50 (d, ^{3}J = 7.2 Hz, 1H), 8.48 (d, ^{3}J = 8.5 Hz, 1H), 8.41 (d, ^{3}J = 6.8 Hz, 1H), 8.35 (d, ^{3}J = 7.2 Hz, 1H), 8.32 (d, ^{3}J = 6.6 Hz, 1H), 7.31 (d, ^{3}J = 8.0 Hz, 1H), 7.21 (t, ^{3}J = 5.4 Hz, 2H), 7.12 (d, ^{3}J = 8.0 Hz, 2H), 6.90 (t, ^{3}J = 8.0 Hz, 1H), 4.51 (m, 1H), 4.44 (m, 1H), 4.37 (m, 1H), 4.36 (m, 1H), 4.34 (m, 1H), 4.31 (m, 1H), 4.18, (m, 1H), 4.13 (m, 1H), 3.84 (m, 2H), 3.70 (m, 1H), 3.64 (m, 1H), 3.55 (m, 2H), 3.22 (m, 2H), 3.01 (t, ^{3}J = 7.3 Hz, 2H), 2.99 (m, 2H), 2.12 (s, 2.2H), 2.06 (m, 2H), 2.05 (s, 0.8H), 1.87-1.96 (m, 3H), 1.58-1.82 (m, 15H).
$^{13}C\{^{1}H\}$ NMR (151 MHz, D_2O, 293 K): δ 176.9, 176.5, 176.2, 175.8, 174.7, 173.7, 173.5, 172.1, 172.1, 169.3, 159.4, 149.8, 147.4, 122.6, 122.5, 122.0, 118.9, 63.9, 58.2, 56.6, 56.2, 56.1, 56.0, 54.4, 53.1, 49.8, 45.6, 43.2, 41.6, 41.5, 30.58, 30.55, 30.53, 30.48, 29.3, 27.2, 26.1, 25.9, 25.1, 22.7, 22.3, 22.0.
$^{15}N\{^{1}H\}$ NMR (61 MHz, H_2O/D_2O, 293 K): δ 180, 178, 176, 124.0, 121.9, 121.5, 121.2, 121.0, 117.6, 109.4, 84.7, 31.8, 31.5.

HRMS for **3:** formula: $C_{40}H_{67}N_{14}O_{14}^+$, calculated: 967.4961, found: 967.4956.

HRMS for **3:iron:** formula: $C_{40}H_{64}N_{14}O_{14}Fe^+$, calculated: 1020.4076, found: 1020.4075.

Comp. 4 (conjugate of pyridomycin 1 and chlorodactyloferrin 2 in its free, iron-, and gallium-complexed forms)

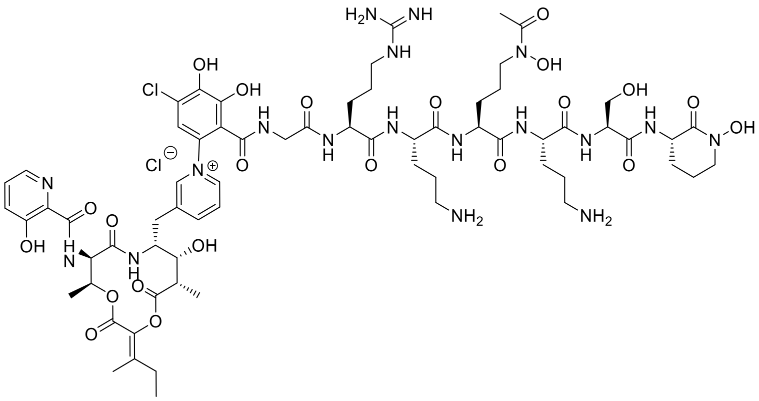

Under nitrogen atmosphere, to an ice-cold solution of comp. **2** (10 mg, 0.01 mmol) and pyridomycin **[1]** (5.4 mg, 0.01 mmol) in water/acetonitrile 1:1 (1 ml), iron trichloride (16.2 mg, 0.1 mmol) was added. The reaction mixture was diluted with a minimum amount of water, filtered and deposited on a C18-column. The target product **4:iron** was eluted by applying a water-acetonitrile gradient. A pale brownish solid was obtained after lyophilization. Yield: 3.8 mg (24%). For NMR characterization, an analytical amount of **4:iron** was dissolved in 1M solution of $GaCl_3$ to allow metal exchange and **4:gallium** was purified using preparative HPLC chromatography.

NMR Comp **4:gallium**

^{1}H NMR (600 MHz, CD_3CN/water, 1/1 v/v, 293 K): δ 9.17 (br, 1H), 8.87 (br, 1H), 8.83 (d, ^3J = 8.3 Hz, 1H), 8.72 (br, 1H), 8.63 (br, 1H), 8.41 (br, 1H), 8.34 (br, 1H), 8.25 (d, ^3J = 5.2 Hz, 1H), 8.08 (br, 1H), 7.84 (d, ^3J = 9.2 Hz, 1H), 7.73 (br, 1H), 7.55 (d, ^3J = 5.9 Hz, 1H), 7.48 (br, 1H), 7.47 (br, 2H), 7.45 (br, 2H), 7.38 (br, 1H), 7.34 (d, ^3J = 9.0 Hz, 1H), 6.90 (m, 2H), 6.61 (s, 1H), 5.22 (m, 1H), 4.71 (m, 1H), 4.55 (d, ^3J = 5.8 Hz, 1H), 4.29 (br, 1H), 4.19 (m, 1H), 4.17 (m, 1H), 4.12 (d, ^3J = 17.5 Hz, 1H), 4.10 (m, 1H), 4.03 (m, 1H), 3.98 (m, 1H), 3.91 (m, 2H), 3.81 (s, 1H), 3.67 (m, 1H), 3.63 (m, 1H), 3.51 (m, 2H), 3.31 (m, 1H), 3.15 (m, 2H), 2.90 (m, 2H), 2.84 (m, 2H), 2.80 (m, 2H), 2.71 (q, ^3J = 7.3 Hz, 1H), 2.19 (m, 1H), 2.12 (s, 3H), 2.07 (m, 1H), 2.06 (s, 3H), 2.01 (m, 1H), 1.94 (m, 2H), 1.90 (m, 2H), 1.87 (m, 1H), 1.86 (m, 1H), 1.81 (m, 2H), 1.79 (m, 1H), 1.73 (m, 2H), 1.71 (m, 1H), 1.68 (m, 2H), 1.64 (m, 1H), 1.59 (m, 1H), 1.45 (m, 1H), 1.41 (d, ^3J = 7.2 Hz, 3H), 1.36 (m, 1H), 1.20 (m, 3H), 0.94 (T, ^3J = 7.6 Hz, 3H).
^{13}C{^{1}H} NMR (151 MHz, CD_3CN/water, 1/1 v/v, 293 K): δ 177.4, 164.2, 160.1(@318K), 158.0 (@318K), 149.9, 148.4, 146.8, 144.4, 131.9, 130.9, 127.6, 127.5, 115.2, 75.5, 73.7, 69.5, 61.3, 57.0, 56.8, 56.7, 56.1, 56.0, 54.9, 54.6, 51.5, 50.3, 48.0, 43.6, 41.5, 39.6, 39.4, 35.8, 28.7, 27.9, 27.7, 27.6, 27.5, 27.2, 26.3, 25.9, 24.7, 24.4, 18.1, 18.0, 17.8, 16.0, 15.0, 12.0.
^{15}N{^{1}H} NMR (61 MHz, CD_3CN/water, 1/1 v/v, 293 K): δ 197 (@318K), 125.1, 123, 121.2, 120.6, 119.3, 119.0, 116.9, 107.7, 84.7.

HRMS for **4:iron:** formula: $C_{67}H_{93}N_{18}O_{22}ClFe+$, calculated: 1592.5750, found: 1592.5743.

Comp. 5 (4-chloro-2,3-dihydroxybenzoate methyl ester)

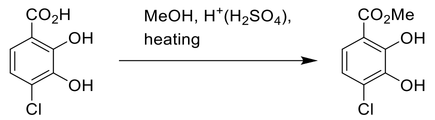

Prepared by following a similar literature protocol [2]. A solution of 4-chloro-2,3-dihydroxybenzoic acid (5.4 g, 28.64 mmol) in MeOH (50 mL) and H_2SO_4 (3 mL, 55.97 mmol) was heated at 100 °C (external temperature) with stirring in a 100 mL pressure vessel for 12 h. The reaction vessel was cooled down, the solvent was removed under reduced pressure, and the obtained residue was dissolved in EtOAc (100 mL). The organic layer was washed consecutively with a saturated solution of sodium bicarbonate, brine, and dried over magnesium sulphate. The organic solvent was evaporated *in vacuo* to afford the respective methyl ester as a pinkish solid. Yield: 5.4 g (93%); m.p.: 100-102 °C. The self-crystallized compound was sufficiently pure to be used in the next step without purification.

^{1}H NMR (300 MHz, $CDCl_3$): δ 11.08 (s, 1H), 7.31 (d, J = 8.8 Hz, 1H), 6.87 (d, J = 8.8 Hz, 1H), 5.98 (s, J = 21.7 Hz, 1H), 3.96 (s, 3H).

^{13}C NMR (75 MHz, $CDCl_3$): δ 170.43, 149.73, 141.69, 125.45, 120.53, 120.28, 111.10, 52.76.

HRMS for **5:** formula: $C_8H_6O_4Cl-$, calculated: 200.9955, found: 200.9958.

Comp. 6 (2,3-dihydroxybenzoate methyl ester)

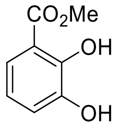

Prepared according to a literature protocol from 2,3 - dihydroxybenzoic acid,[2] m.p.: 74-76°C.

^{1}H NMR (300 MHz, $CDCl_3$): δ 10.90 (s, 2H), 7.36 (dd, *J* = 8.1, 1.5 Hz, 2H), 7.11 (dd, *J* = 7.9, 0.9 Hz, 2H), 6.79 (t, *J* = 8.0 Hz, 2H), 5.74 (s, 3H), 3.95 (s, 8H).

^{13}C NMR (75 MHz, DMSO-d_6): δ 170.90, 148.97, 145.16, 120.70, 119.99, 119.36, 112.52, 52.56.

HRMS for **6:** formula: $C_8H_7O_4$-, calculated: 167.0344, found: 167.0346.

Comp. 7 (4-chloro-2,3-dimethoxybenzoate methyl ester)

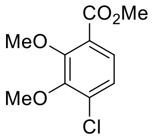

Using 4-chloro-2,3-dimethoxybenzoic acid [3,4] (380 mg, 1.75 mmol), methanol (5mL) and sulphuric acid (300 µL), the title comp. was prepared by following the same procedure described for **5** , yellowish gum. Yield: 49%.

^{1}H NMR (300 MHz, $CDCl_3$): δ 6.98 (d, *J* = 8.9 Hz, 1H), 6.79 (d, *J* = 8.9 Hz, 1H), 3.86 (s, 3H), 3.78 (s, 3H), 3.77 (s, 3H).

^{13}C NMR (75 MHz, $CDCl_3$): δ 165.80 (CO), 151.63 (Cq), 146.90 (Cq), 129.18 (Cq), 124.83 (CH), 121.67 (Cq), 114.19 (CH), 77.16, 61.72 (CH3), 56.19 (CH3), 52.74 (CH3).

HRMS for **7:** formula: $C_{10}H_{12}O_4Cl$+, calculated: 231.0424, found: 231.0426.

Comp. 11 (4-chloro-2,3-dihydroxybenzoyl-glycinate methyl ester)

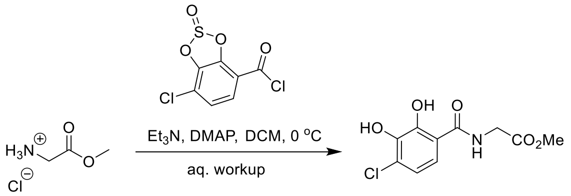

The procedure for preparing 4-chloro-2,3-dioxosulfinylbenzoyl chloride was identical to that of 2,3-dioxosulfinylbenzoyl chloride [5]. Under nitrogen atmosphere, a solution of freshly prepared 4-chloro-2,3-dioxosulfinylbenzoyl chloride (2 g, 7.94 mmol) in anhydrous DCM (20 mL) was cooled to 0 °C. This solution was then added dropwise, through a cannula, to a stirred mixture of triethylamine (9 mL, 64.53 mmol), DMAP (200 mg, 1.64 mmol) and glycine methyl ester hydrochloride (2 g, 15.93 mmol) in anhydrous DCM (40 mL) at 0 °C (ice-water bath). The reaction mixture was allowed to gradually warm to room temperature while stirring for 9 h. The volatiles were removed by rotary evaporation, and the obtained residue was dissolved in ethyl acetate (100 mL). The organic layer was then washed with 5% citric acid (2 × 30 mL), brine (1 × 30 mL), dried over magnesium sulphate, filtered, and concentrated under reduced pressure. Subjection of the obtained residue to flash chromatography (silica gel, DCM/MeOH/AcOH in 96/3/1, v/v/v) and concentration of the relevant fractions under reduced pressure afforded the title compound as an off-white solid. Yield: 900 mg (44%); m.p. 156-158 °C.

^{1}H NMR (300 MHz, DMSO-*d*6): δ 12.95 (s, 1H), 9.70 (s, 1H), 9.36 (t, *J* = 5.7 Hz, 1H), 7.36 (d, *J* = 8.8 Hz, 1H), 6.94 (d, *J* = 8.8 Hz, 1H), 4.07 (d, *J* = 5.8 Hz, 2H), 3.67 (s, 3H).

^{13}C NMR (75 MHz, DMSO-*d*6): δ 169.87 (CO), 169.80 (CO), 150.92 (Cq), 142.62 (Cq), 124.10(Cq), 119.07 (CH), 117.58 (CH), 112.92 (Cq), 51.95 (CH3), 40.97 (CH2).

HRMS for **11:** formula: $C_{10}H_{11}NO_5Cl+$, calculated: 260.0326, found: 260.0320.

Comp. 16 (conjugate of 5 and 12 generated by oxidation to give compounds 16a/16b/16c)

Comp. 16a (FeCl$_3$ oxidation)

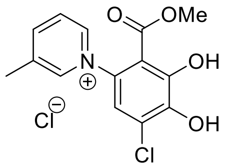

Under nitrogen atmosphere, to an ice-cold solution of catechol **5** (20 mg, 0.1 mmol) and 3-methylpyridine (40 µL, 0.42 mmol) in acetonitrile (5 ml) under vigorous stirring iron trichloride hexahydrate (80 mg, 0.29 mmol) was added in one portion. A sudden change of the color to intense blue was observed. The reaction mixture was stirred at room temperature for 12 h. The reaction mixture was concentrated by rotary evaporation, and the residue was dissolved in a minimum amount of methanol/water mixture (1:1, v/v) and purified using Sephadex LH-20 with methanol/water (8:2, v/v). Yield as a red solid (iron salt): 32 mg (59%). The obtained product was further purified by depositing the product on a C18-column and eluting first with a 5% EDTA solution of followed by water-acetonitrile gradient. To replace traces of complexed iron, an analytical amount of the product was dissolved in 1M solution of $GaCl_3$ and purified using preparative HPLC chromatography with water and acetonitrile as eluents.

^{1}H NMR (300 MHz, DMSO-*d*6): δ 10.83 (br.s, 2H), 9.14 (s, 1H), 9.01 (d, *J* = 6.0 Hz, 1H), 8.64 (d, *J* = 8.0 Hz, 1H), 8.15 (dd, *J* = 8.0, 6.1 Hz, 1H), 7.55 (s, 1H), 3.55 (s, 3H), 2.54 (s, 3H).

^{13}C NMR (75 MHz, DMSO-*d*6): δ 164.60 (CO), 147.92 (Cq), 147.52 (CH), 145.85 (CH), 145.75 (Cq), 143.73 (CH), 138.34 (Cq), 132.23 (Cq), 126.72 (CH), 122.58 (Cq), 119.15 (CH), 113.01 (Cq), 52.91 (CH3), 17.73(CH3).

HRMS for **16a:** formula: $C_{14}H_{13}ClNO_4+$, calculated: 294.0533, found: 294.0539.

Comp. 16b (iodine oxidation)

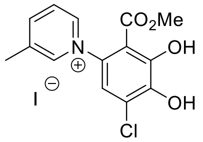

The synthetic procedure was carried according to the modified literature procedure [6]. Under nitrogen atmosphere, to an ice-cold solution of catechol **5** (304 mg, 1.5 mmol) and 3-methylpyridine (292 µL, 3 mmol) in methanol (20 mL) under vigorous stirring solid iodine (381 mg, 1.5 mmol) was added in one portion. After ca. 25 min the solution was allowed to warm to room temperature and heated for 40 min at 60 °C. The reaction mixture was then cooled and concentrated by rotary evaporation. The obtained residue was precipitated using ethyl acetate. The obtained precipitate was separated by centrifugation, washed with small amounts of ethyl acetate, and dried in high vacuum. Yield as a pale brownish solid: 381 mg (60%); m.p. 232-235 °C.

^{1}H NMR (300 MHz, DMSO-d_6): δ 8.99 (s, 1H), 8.85 (d, *J* = 5.8 Hz, 1H), 8.54 (d, *J* = 7.9 Hz, 1H), 8.03 (dd, *J* = 7.4, 6.6 Hz, 1H), 6.91 (s, *J* = 7.1 Hz, 1H), 3.45 (s, *J* = 7.8 Hz, 3H), 2.51 (s, 3H)

^{13}C NMR (75 MHz, DMSO-d_6): δ 165.7 (C=O), 155.1 (Cq), 149.7 (Cq), 146.8 (CH), 145.7 (CH), 143.6 (CH), 138.3 (Cq), 131.5 (Cq), 126.7 (CH), 117.5 (Cq), 113.9 (CH), 108.9 (Cq), 52.1 (CH3), 17.9 (CH3).

HRMS for **16b:** formula: $C_{14}H_{13}ClNO_4+$, calculated: 294.0533, found: 294.0532.

Comp. 16c (silver oxide oxidation)

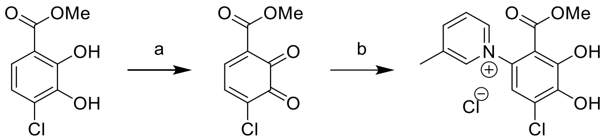

The synthetic procedure was carried by following a similar literature protocol [7]. A solution of **5** (20 mg, 0.1 mmol) in dry DMF (5 mL) was stirred with Ag_2O (91 mg, 0.39 mmol) for two hours at 0 °C. Formation of the respective *o*-quinone was confirmed by LC-MS. Using a syringe-fitted 200 nm Teflon filter, the dark-green solution was rapidly filtered into a mixture of 3-methylpyridine (40 µL, 0.41 mmol), MeOH (5 mL) and acetic acid (60 µL, 1 mmol). The reaction mixture was stirred at 0 °C for 1 hr and then allowed to warm to room temperature for 1 hr. The organic solvents were removed by rotary evaporation. Diethyl ether (10 mL) was added to the obtained residue. The resulting precipitate was washed and centrifuged several times with fresh portions of diethyl ether. The residue was then dissolved with 5 molar equivalents of hydrochloric acid in DI water (20 mL) and purified by preparative HPLC with water and acetonitrile (with 0.1% TFA) as eluents to give a white solid after lyophilization. Yield: 30 mg (92%).

^{1}H NMR (300 MHz, DMSO-*d*6): δ 8.96 (s, 1H), 8.82 (s, 1H), 8.48 (d, *J* = 6.2 Hz, 1H), 7.98 (s, 1H), 6.67 (s, 1H), 3.46 (s, 3H), 2.50 (s, 3H).

^{13}C NMR (75 MHz, DMSO-*d*6): δ 165.98 (C=O), 156.80 (Cq), 151.59 (Cq), 146.27 (CH), 145.69 (CH), 143.63 (CH), 138.20 (Cq), 130.50 (Cq), 126.56 (CH), 115.31 (Cq), 112.12 (CH), 107.50 (Cq), 51.59 (OCH3), 17.86 (CH3).

HRMS for **16c:** formula: $C_{14}H_{13}ClNO_4+$, calculated: 294.0533, found: 294.0518.

Comp. 17 (conjugate of 6 and 12 generated by oxidation to give compounds 17a and 17b)

Comp. 17a ($FeCl_3$ oxidation)

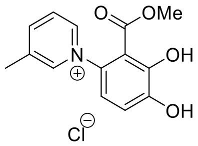

Formation of the title product was confirmed on the analytical scale and checked by LC-MS using catechol **6** (10 µL, 10 mM soln in MeOH), 3-methylpyridine (10 µL, 10 mM soln in MeOH) and iron trichloride (10 µL, 100 mM soln in DI water) as starting compounds and following the same procedure as in the case of **16a**. Formation of the title product **17a** was confirmed by LC-MS.

Comp. 17b (iodine oxidation)

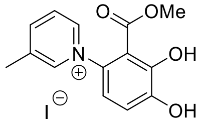

Using catechol **6** (303 mg, 1.80 mmol), 3-methylpyridine (350 µL, 3.61 mmol) and iodine (732 mg, 2.88 mmol) as starting compounds, the title compound was prepared as a brownish solid according to the method used to

prepare **16b**. Yield: 429 mg (80%); m.p.: 202-204 °C (with decomposition). Formation of the title product **17b** was confirmed by LC-MS.

^{1}H NMR (300 MHz, DMSO-*d*6): δ 10.65 (s, 1H), 10.25 (s, 1H), 9.10 (s, 1H), 8.97 (d, *J* = 6.0 Hz, 1H), 8.63 (d, *J* = 8.0 Hz, 1H), 8.14 (dd, *J* = 8.0, 6.1 Hz, 1H), 7.23 – 7.07 (m, 2H), 3.54 (s, 3H), 2.55 (s, 3H).

^{13}C NMR (75 MHz, DMSO-*d*6): δ 164.91 (CO), 148.79 (Cq), 147.21 (CH), 146.42 (Cq), 145.64 (CH), 143.54 (CH), 138.51 (Cq), 132.21 (Cq), 126.87 (CH), 117.84 (CH), 116.73 (CH), 114.79 (Cq), 52.72 (OCH3), 17.77 (CH3).

HRMS for **17b:** formula: $C_{14}H_{14}NO_4+$, calculated: 260.0923, found: 260.0923.

Comp. 18 (conjugate of 11 and 12 generated by oxidation to give compounds 18a/18b/18d)

Comp. 18a ($FeCl_3$ oxidation)

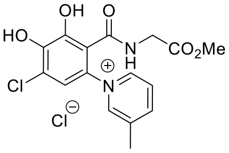

Under argon atmosphere, to an ice-cold (ice-water bath) stirring solution of catechol **11** (85 mg, 0.33 mmol) in a mixture acetonitrile-water (3 mL, 1:1, v/v) was added 3-picoline (46 µL, 0.33 mmol) followed by ground iron trichloride hexahydrate (887 mg, 3.28 mmol) suspended in a mixture acetonitrile-water (2 mL, 1:1, v/v). The reaction mixture was allowed to stir for 1 h. The obtained suspension was centrifuged, filtered, and the filtered solution was loaded on a high-pressure C18-column. The column was first washed with a solution of gallium trichloride (3 eq.) in DI water, and the target product was then eluted with a water/acetonitrile (1% TFA in both solvents) gradient. Yield as a yellowish solid after lyophilization: 32 mg, (28%).

^{1}H NMR (300 MHz, DMSO-d_6): δ 9.53 (s, 1H), 8.93 (s, 1H), 8.83 (d, *J* = 6.0 Hz, 1H), 8.56 (d, *J* = 8.0 Hz, 1H), 8.06 (dd, *J* = 8.0, 6.1 Hz, 1H), 7.26 (s, 1H), 3.87 (d, *J* = 4.9 Hz, 2H), 3.55 (s, 3H), 2.52 (s, 3H).
^{13}C NMR (75 MHz, DMSO-d_6): δ 169.80 (C=O), 163.31 (C=O), 148.33 (Cq), 146.80 (CH), 145.78 (Cq), 145.62 (CH), 143.53 (CH), 138.11 (Cq), 132.58 (Cq), 126.59 (CH), 119.76 (Cq), 117.54 (Cq), 116.42 (CH), 51.74 (OCH3), 40.81 (CH2), 17.82 (CH3).

HRMS for **18a**: formula: $C_{16}H_{16}ClN_2O_5^+$, calculated: 351.0740, found: 351.0748.

Comp. 18b (iodine oxidation)

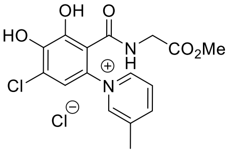

Using catechol derivative **11** (353 mg, 1.36 mmol), 3-methylpyridine (450 µL, 4.6 mmol) and iodine (588 mg, 2.3 mmol) as starting compounds, the title compound was prepared as an off-white solid according to the synthesis of **16b**. Yield as a hydrochloride salt after ion-exchange (sodium dithionate, bicarbonate/HCl): 373 mg (71%). m.p. 216-220 °C (with decomp.).

^{1}H NMR (300 MHz, DMSO-*d*6): δ 10.64 (br. s, 2H), 9.45 (s, 1H), 9.00 (s, 1H), 8.88 (d, *J* = 6.0 Hz, 1H), 8.58 (d, *J* = 8.0 Hz, 1H), 8.08 (dd, *J* = 7.9, 6.2 Hz, 1H), 7.35 (s, 1H), 3.85 (d, *J* = 5.5 Hz, 2H), 3.53 (s, 3H), 2.53 (s, *J* = 4.1 Hz, 3H).

^{13}C NMR (75 MHz, DMSO-*d*6): δ 169.64 (C=O), 163.01 (C=O), 146.87 (CH), 146.40 (Cq), 145.69 (CH), 145.27 (Cq), 143.48 (CH), 138.19 (Cq), 132.19 (Cq), 126.67 (CH), 120.50 (Cq), 119.23 (Cq), 117.29 (CH), 51.70 (CH3), 40.78 (CH2), 17.82 (CH3).

HRMS for **18b:** formula: $C_{16}H_{16}N_2O_5Cl^+$, calculated: 351.0748, found: 351.0749.

Comp. 18d (electrochemical oxidation)

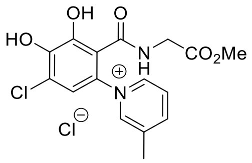

A solution of sodium acetate buffer (0.1 M; pH = 7.2) in water/acetonitrile (50:50; 4 mL) containing catechol **11** (63 mg, 0.24 mmol) and 3-methylpyridine (80 µL, 0.82 mmol) was electrolyzed in an undivided cell at a constant voltage of 0.65V (2 mA, 2F/mol) *vs.* Ag/AgCl for 7 hr at room temperature. After electrolysis, the solution was acidified with 0.2 M hydrochloric acid and lyophilized. The obtained powder was dissolved in DI water and purified by preparative HPLC using water and acetonitrile (0.1% TFA in both solvent) as eluents. The collected fractions were frozen and lyophilized. Yield as a white solid: 45 mg (48%).

^{1}H NMR (300 MHz, MeOH-*d*4): δ 8.84 (s, 1H), 8.79 (d, *J* = 6.0 Hz, 1H), 8.54 (d, *J* = 8.0 Hz, 1H), 8.03 (dd, *J* = 8.0, 6.1 Hz, 1H), 7.31 (s, 1H), 3.98 (s, 2H), 3.66 (s, 3H), 2.63 (s, 3H).

^{13}C NMR (75 MHz, MeOH-*d*4): δ 161.74, 155.78, 138.92, 138.00, 137.62, 137.22, 135.22, 131.39, 118.61, 113.75, 109.97, 109.75, 43.15, 32.49, 9.01.

HRMS for **18d:** formula: $C_{16}H_{16}N_2O_5Cl^+$, calculated: 351.0748, found: 351.0743.

Comp. 18e (reduction of 18a obtained by oxidation with $FeCl_3$)

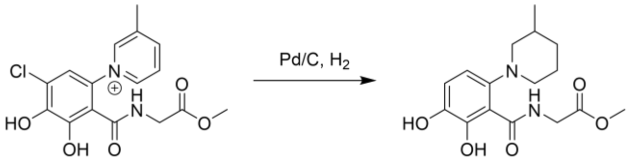

Synthesis scheme of the piperidine analogue **18e**: (i) 10% Pd/C, MeOH/EtOH (3:1, v/v), 16 hr, 0 °C to RT, 23%.

The reaction was conducted in the same manner as for comp **18f** using 10 mg of comp **18a** and 20 mg of 10% Pd/C catalyst; yield: 2.4 mg (23%).

^{1}H NMR (300 MHz, DMSO-*d*6): δ 13.67 (s, 1H),11.89 (t, *J* = 5.5 Hz, 1H), 8.93 (s, 1H), 6.87 (d, *J* = 8.5 Hz, 1H), 6.71 (d, *J* = 8.5 Hz, 1H), 4.24 (d, *J* = 5.5 Hz, 2H), 3.69 (s, 3H), 2.88 (m, 2H), 2.58 (m, 1H), 2.29 (t, *J* = 10.8 Hz, 1H), 1.84 (m, 1H), 1.71 (m, 3H), 0.95 (m, 1H), 0.86 (d, *J* = 6.6 Hz, 3H).

HRMS for **18e**: formula: $C_{16}H_{23}N_2O_5^+$, calculated: 323.1529, found: 323.1607.

Comp. 18f (reduction of 18b obtained by oxidation with iodine)

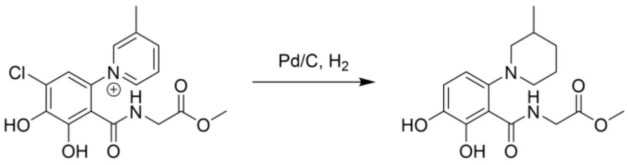

18b **18f**

Synthesis Scheme of the piperidine analogue **18f**: (i) 10% Pd/C, MeOH/EtOH (3:1, v/v), 16 hr, 0 °C to RT, 57%. A reduced version of 10% Pd/C catalyst was obtained by applying H_2 pressure (*ca.* 2 atm) to the Pd catalyst (100 mg) in a mixture of methanol/ethanol (3:1, v/v) for 1 h in a 50 mL two-chamber hydrogen generation apparatus ("H-tube")[8]. The pressure was carefully released, and solid **18 b** (70 mg, 0.18 mmol) was added to the suspension in one portion under nitrogen. The reaction mixture was cooled down with ice-water, and the hydrogen pressure was reapplied. The reaction mixture was stirred vigorously and gradually warmed to room temperature over a period of 16 h while under H_2 pressure. The hydrogen pressure was released, and the reaction mixture was transferred to a 50 mL plastic tube using methanol. The obtained suspension was centrifuged, and the supernatant was collected by decantation. The washing procedure was repeated two more times using methanol to wash the catalyst. The combined organic solution was concentrated under vacuum. The residue was extracted into ethyl acetate (100 mL), washed with aqueous sodium bicarbonate (2 × 20 mL), dried ($MgSO_4$), and concentrated by rotary evaporation. The obtained residue was resuspended in DMSO/water mixture (1:1, v/v) and applied to a C18-column. A water/acetonitrile (with 1% TFA) linear gradient system was employed to provide a cherry-like gum after lyophilization of the respective fractions; yield: 33 mg (57%).

^{1}H NMR (600 MHz, DMSO-*d*6): δ 13.68 (s, 1H),11.90 (t, *J* = 5.5 Hz, 1H), 8.93 (s, 1H), 6.87 (d, *J* = 8.6 Hz, 1H), 6.71 (d, *J* = 8.6 Hz, 1H), 4.24 (d, *J* = 5.5 Hz, 2H), 3.69 (s, 3H), 2.88 (m, 2H), 2.57 (t, *J* = 11.5 Hz, 1H), 2.29 (t, *J* = 10.9 Hz, 1H), 1.85 (m, 1H), 1.71 (m, 3H), 0.96 (m, 1H), 0.86 (d, *J* = 6.6 Hz, 3H).

^{13}C NMR (151 MHz, DMSO-*d*6): δ 171.06 (CO), 169.96 (CO), 151.41 (Cq), 144.50 (Cq), 143.88 (Cq), 118.07(CH), 111.79 (CH), 108.37 (Cq), 61.73 (CH2), 54.23 (CH2), 52.06 (CH3), 40.71 (CH2), 31.93 (CH2), 31.13 (CH), 25.40 (CH2), 19.37 (CH3).

^{15}N NMR (61 MHz, DMSO-*d*6): δ 110.6 (NH), 54.7 (Nq).

HRMS for **18f**: formula: $C_{16}H_{23}N_2O_5^+$, calculated: 323.1529, found: 323.1607.

Comp. 19 (conjugate of 5 and 13 generated by oxidation to give compounds 19a/19b/19c)

Comp. 19a ($FeCl_3$ oxidation)

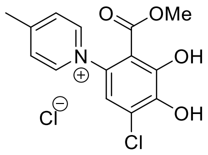

Formation of the title product was confirmed on the analytical scale by LC-MS using catechol **5** (10 µL, 10 mM soln in MeOH), 4-methylpyridine (10 µL, 10 mM soln in MeOH) and iron trichloride (10 µL, 100 mM soln in DI water) as starting compounds and following the same procedure as in the case of **17a**.

Comp. 19b (iodine oxidation)

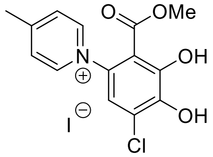

Using catechol **5** (40 mg, 0.2 mmol), 4-methylpyridine (40 µL, 0.39 mmol) and iodine (50 mg, 0.2 mmol) as starting compounds, the title compound was prepared as a brownish solid according to the same method for **16b**. Yield: 74 mg (purity *ca.* 37%, yield 28%). Formation of the title product was confirmed by analytical LC-MS.

^{1}H NMR (300 MHz, DMSO-*d*6): δ 9.02 (d, *J* = 6.6 Hz, 2H), 8.10 (d, *J* = 6.5 Hz, 2H), 7.55 (s, 1H), 3.55 (s, 3H), 2.72 (s, 3H).

HRMS for **19b:** formula: $C_{14}H_{13}ClNO_4$+, calculated: 294.0533, found: 294.0526.

Comp. 19c (silver oxide oxidation)

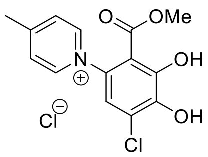

Using catechol **5** (80 mg, 0.39 mmol), 4-methylpyridine (80 µL, 0.82 mmol) and silver oxide (364 mg, 1.57 mmol) as starting compounds, the title compound was prepared by following the same protocol as described for comp. **16c**. The title product was further purified by preparative HPLC using water and acetonitrile with 0.1 % TFA as eluents. The lyophilized product dissolved in DI water with 10 eq of HCl and lyophilized again. Yield: 55 mg (43%) as an off-white solid. M.p.: 196-200 °C (with decomp.). Formation of the title product was confirmed by analytical LC-MS.

^{1}H NMR (300 MHz, DMSO-*d*6): δ 9.02 (d, *J* = 6.7 Hz, 2H), 8.08 (d, *J* = 6.5 Hz, 2H), 7.53 (s, 1H), 3.56 (s, 3H), 2.71 (s, 3H).

^{13}C NMR (75 MHz, DMSO-*d*6): δ 164.67(C=O), 161.10 (Cq), 147.86 (Cq), 145.78 (Cq), 145.32 (CH), 131.96 (Cq), 127.73 (CH), 122.59 (Cq), 119.18 (CH), 113.31 (Cq), 52.89 (CH3), 21.76 (CH3).

HRMS for **19c:** formula: $C_{14}H_{13}ClNO_4$+, calculated: 294.0533, found: 294.0504.

Comp. 20 (conjugate of 5 and 14 generated by oxidation to give compounds 20a/20b/20c)

Comp. 20a ($FeCl_3$ oxidation)

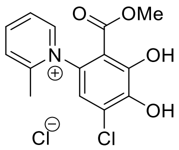

Formation of the title product was confirmed on the analytical scale by LC-MS using catechol **5** (10 µL, 10 mM soln in MeOH), 2-methylpyridine (10 µL, 10 mM soln in MeOH) and iron trichloride (10 µL, 100 mM soln in DI water) as starting compounds and following the same procedure as in the case of **17a.** Formation of the title product was confirmed by analytical LC-MS. The low synthetic throughput would require a multistage RP-HPLC purification protocol therefore preventing NMR interpretation of the title product.

Comp. 20b (iodine oxidation)

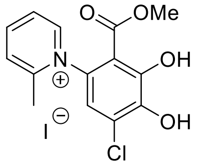

Using catechol **5** (40 mg, 0.2 mmol), 2-methylpyridine (40 µL, 0.39 mmol) and iodine (50 mg, 0.2 mmol) as starting compounds, the title compound was prepared as a brownish solid according to the method used to prepare **16b**. Yield: 14 mg (17%). M.p.: > 250 °C. Formation of the title product **20b** was confirmed by analytical LC-MS. The low synthetic throughput would require a multistage RP-HPLC purification therefore preventing NMR interpretation of the title product.

HRMS for **20b:** formula: $C_{14}H_{13}ClNO_4$+, calculated: 294.0533, found: 294.0524.

Comp. 20c (silver oxide oxidation)

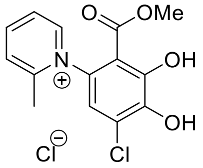

Using catechol **5** (80 mg, 0.39 mmol), 2-methylpyridine (80 µL, 0.82 mmol) and silver oxide (364 mg, 1.57 mmol) as starting compounds, the title compound was prepared by following the same protocol as described for comp. **16c**. Formation of the title product **20c** was confirmed by analytical LC-MS. Yield: 16 mg (12%) as an off-white solid.

^{1}H NMR (300 MHz, DMSO-*d*6): δ 10.90 (br. s, 2H), 9.01 (dd, *J* = 6.2, 1.1 Hz, 1H), 8.68 (ddd, *J* = 7.9, 1.4 Hz, 1H), 8.20 (d, *J* = 7.9 Hz, 1H), 8.05 (dd, *J* = 6.5 Hz, 1H), 7.50 (s, 1H), 3.52 (s, 3H), 2.52 (s, 3H).

^{13}C NMR (75 MHz, DMSO-*d*6): δ 164.75 (CO), 157.15 (Cq), 148.54 (Cq), 147.37 (CH), 146.54 (CH), 145.76 (Cq), 129.93 (Cq), 128.93 (CH), 124.99 (CH), 123.11 (Cq), 118.82 (CH), 112.48 (Cq), 52.98 (OCH3), 20.42 (CH3).

HRMS for **20c:** formula: $C_{14}H_{13}ClNO_4$+, calculated: 294.0533, found: 294.0536.

Comp. 21 (3-pyridyl-TAMRA)

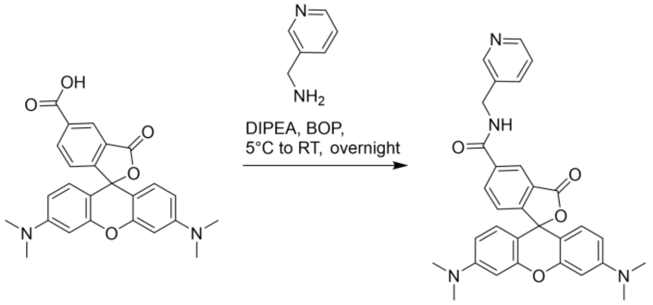

[21]

5-TAMRA (0.5g, 1.16 mmol) was dissolved in 10 mL of DCM in a 50 mL round-bottom flask. The obtained solution was cooled to 5 °C using an ice-bath, and DIPEA (404 µL, 2 eq.) followed by BOP (benzotriazol-1-yloxytris(dimethylamino)phosphonium hexafluorophosphate) (0.512 g, 1 eq.) were added portion wise while stirring. Following 10 min of stirring, 3-picolylamine (0.125 g, 1 eq.) was added to the reaction dropwise, and the

reaction was maintained at 5 °C for 1h followed by continued stirring at room temperature overnight. Full conversion was confirmed by TLC (DCM/MeOH, 9/1, v/v). The reaction mixture was concentrated under vacuum, and the title product was purified by flash chromatography on silica using a gradient of DCM/MeOH (from 0 to 20% of MeOH). The title product **21** was eluted at 15% MeOH. After drying in vacuo, comp **21** was obtained as an intense purple powder (0.270 g, 45% yield).

^{1}H NMR (300 MHz, DMSO-*d*6): δ 9.44 (t, *J* = 5.8 Hz, 1H), 8.60 (s, 1H), 8.49 (d, *J* = 5.6 Hz, 2H), 8.27 (dd, *J* = 8.1, 1.5 Hz, 1H), 7.83 – 7.71 (m, 1H), 7.38 (dd, *J* = 7.9, 4.9 Hz, 1H), 7.33 (d, *J* = 8.0 Hz, 1H), 6.58 – 6.43 (m, 6H), 4.55 (d, *J* = 5.8 Hz, 2H), 2.94 (s, 12H).

HRMS for **21:** formula: $C_{31}H_{29}N_4O_4$+, calculated: 521.2189, found: 521.2182.

Comp. 22 (conjugate of 21 and Chlorodactyloferrin [2] in its iron- and gallium-complexed forms)

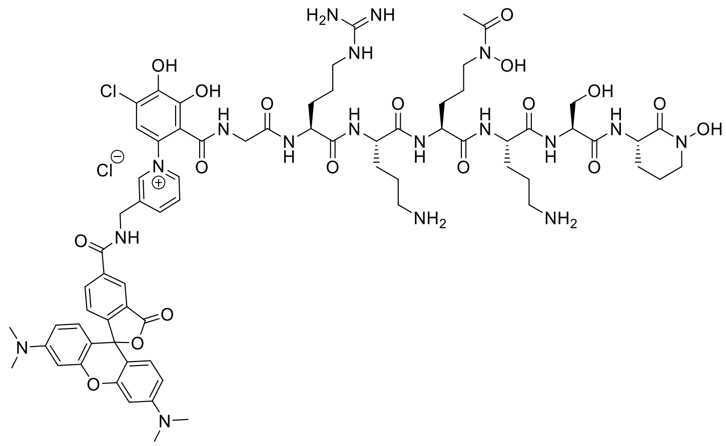

Under nitrogen atmosphere, to an ice-cold solution of **2** (10 mg, 0.01 mmol) and **21** (5.21 mg, 0.01 mmol) in water/acetonitrile (1 ml, 1:1, v/v), iron trichloride was added (16.2 mg, 0.1 mmol, 10 eq.). The reaction mixture was diluted with a minimum amount of water, filtered and deposited on a C18-column. The target product **22:iron** was eluted by applying a water-acetonitrile gradient. Deep red/purple solid was obtained after lyophilization. Yield: 3.2 mg. For NMR analysis, **22:iron** was treated with 1M solution of $GaCl_3$, and the resulting **22:gallium** complex was purified by preparative HPLC.

NMR for **22:gallium**

^{1}H NMR (600 MHz, CD_3OD, 293 K): δ 8.93 (d, ^{3}J = 5.8 Hz, 1H), 8.84 (s, 1H), 8.74 (s, 1H), 8.70 (d, ^{3}J = 8.2 Hz, 1H), 8.15 (m, 1H), 8.14 (m, 1H), 7.42 (d, ^{3}J = 7.9 Hz, 1H), 7.30 (d, ^{3}J = 9.5 Hz, 1H), 7.21 (d, ^{3}J = 9.5 Hz, 1H), 7.04 (dd, ^{3}J = 9.5 Hz, ^{4}J = 2.5 Hz, 1H), 7.01 (dd, ^{3}J = 9.5 Hz, ^{4}J = 2.5 Hz, 1H), 6.96 (d, ^{4}J = 2.5 Hz, 1H), 6.95 (d, ^{4}J = 2.5 Hz, 1H), 6.94 (s, 1H), 4.92 (m, 1H), 4.81 (d, ^{2}J = 15.0 Hz, 1H), 3.30 (s, 6H), 3.29 (s, 6H).
^{13}C{^{1}H} NMR (151 MHz, CD_3OD, 293 K): δ 168.4, 163.2, 158.9, 158.8, 155.4, 146.4, 146.0, 146.0, 137.7, 132.9, 132.6, 131.2, 130.7, 129.3, 129.2, 118.6, 115.4, 115.3, 115.0, 114.8, 97.3, 50.8, 41.8.
^{15}N{^{1}H} NMR (61 MHz, CD_3OD, 293 K): δ 85.

HRMS for **22:iron:** formula: $C_{71}H_{89}N_{18}O_{18}ClFe$+, calculated: 1572.5640, found: 1572.5665.

Comp. 23 (3-pyridyl-penicillin)

Comp. 23-1 (Compound 23 intermediate 1)

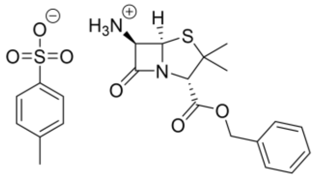

23-1

Comp **23-1** was prepared using a similar literature protocol[5]. Under nitrogen, 6-aminopenicillanic acid (2.16 g, 10 mmol) was suspended in acetone (25 mL) at 0 °C with stirring. Triethylamine (1.4 mL, 10 mmol) was added dropwise to the suspension, and the reaction mixture was stirred for 20 min at 0 °C. Benzyl bromide (1.2 mL, 10 mmol) in acetone (35 mL) was added dropwise to the reaction mixture. Stirring continued overnight while warming to room temperature. The precipitate was removed by filtration, washed with dry diethyl ether (30 mL) and discarded. The filtrate was washed with saturated sodium bicarbonate (30 mL) and dried over $MgSO_4$. The ether layer was then treated with *p*-toluenesulfonic acid (1.7 g, 8.9 mmol) in acetone (10 mL) forming the title compound (2.8 g, 59%) as a white precipitate, which was then isolated by filtration and dried in high vacuum.

^{1}H NMR (300 MHz, DMSO-*d*6): δ 8.91 (br. s, 3H), 7.50 (d, *J* = 8.1 Hz, 2H), 7.46 – 7.32 (m, 5H), 7.12 (d, *J* = 7.8 Hz, 2H), 5.53 (d, *J* = 4.3 Hz, 1H), 5.22 (s, 2H), 5.12 (d, *J* = 4.3 Hz, 1H), 4.57 (s, 1H), 2.29 (s, 3H), 1.61 (s, 3H), 1.38 (s, 3H).

^{13}C NMR (75 MHz, DMSO-*d*6): δ 168.61 (C=O), 166.97 (C=O), 145.47 (Cq), 137.82 (Cq), 135.12 (Cq), 128.68 (CH), 128.58 (CH), 128.55 (CH), 128.13 (CH), 125.53 (CH), 69.79(CH), 67.06 (CH2), 65.16 (Cq), 64.14 (CH), 57.17 (CH), 30.80 (CH3), 26.60 (CH3), 20.81 (CH3).

Comp. 23-2 (Compound 23 intermediate 2)

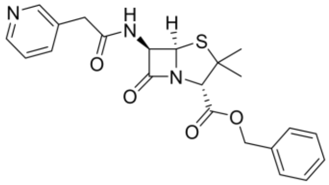

23-2

Comp **23-2** was prepared by following a similar literature protocol[5]. Comp. **23-1** (3.2 g, 6.53 mmol) was suspended in EtOAc (100 mL), washed with saturated sodium bicarbonate (2×30 mL) and dried over $MgSO_4$. The organic solvent was removed *in vacuo* to give the corresponding amine (ca. 2.3 g, quant) as an oily residue. The residue was dissolved in anhydrous DCM (50 mL), and the obtained solution was cooled down with an ice-water bath. Under nitrogen atmosphere, HOBT (1.412 g, 10.45 mmol), 3-pyridylacetic acid (2.04 g, 10.45 mmol), DIPEA (2.8 mL, 16.32 mmol), and EDAC (2 g, 10.43 mmol) were added to the obtained solution. The reaction mixture was stirred for 12 hr while warming to room temperature. The volatiles were removed using rotary evaporation, and the obtained residue was extracted into ethyl acetate. The organic layer was washed with concentrated sodium bicarbonate, brine, dried over anhydrous $MgSO_4$, and evaporated under reduced pressure. Flash chromatography (ethyl acetate) afforded the title product as a white solid. Yield: 1.9 g (68%). M.p.: 58-60 °C.

^{1}H NMR (300 MHz, CDCl3): δ 8.51 (dd, *J* = 4.8, 1.5 Hz, 1H), 8.48 (d, *J* = 1.8 Hz, 1H), 7.65 (d, *J* = 7.9 Hz, 1H), 7.39 – 7.30 (m, *J* = 6.6 Hz, 6H), 7.26 (dd, *J* = 7.8, 4.9 Hz, 1H), 5.63 (dd, *J* = 8.4, 4.1 Hz, 1H), 5.51 (d, *J* = 4.1 Hz, 1H), 5.16 (s, 2H), 4.39 (s, 1H), 3.59 (s, 2H), 1.47 (s, 3H), 1.37 (s, 3H).

^{13}C NMR (75 MHz, CDCl3): δ 173.30 (C=O), 169.59 (C=O), 167.38 (C=O), 150.16 (CH), 148.47 (CH), 136.95 (CH), 134.59 (Cq), 130.20 (Cq), 128.69 (CH), 128.65 (CH), 128.61 (CH), 123.57 (CH), 70.30 (CH), 67.99 (CH), 67.43 (CH2), 64.73 (Cq), 58.93 (CH), 39.78 (CH2), 31.53 (CH3), 26.80 (CH3).

HRMS for **23-2:** formula: $C_{22}H_{24}N_3O_4S^+$, calculated: 426.1488, found: 426.1476.

Comp. 23 (3-pyridyl-penicillin)

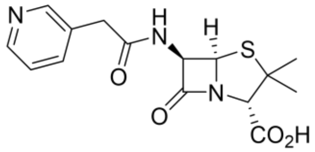

23

Comp. **23-2** (723 mg) was hydrogenated over 10% palladium on carbon (ca. 0.2 g) in a mixture of EtOH/EtOAc for 16 h using *in situ* generated hydrogen in a 20 mL Coware® apparatus[5]. After completion, the catalyst was removed by filtration through Celite and the filtrate was evaporated under vacuum. The obtained residue was purified using a gradient elution from water to 100% acetonitrile, both with 0.1% TFA. Yield: 162 mg (21%) of the title comp. **23** as a white solid.

^{1}H NMR (300 MHz, DMSO-*d*6): δ 9.10 (d, *J* = 6.6 Hz, 1H), 8.77 – 8.59 (m, 3H), 8.12 (d, *J* = 8.0 Hz, 1H), 7.74 (dd, *J* = 7.8, 5.4 Hz, 1H), 5.50 – 5.45 (m, 2H), 4.25 (s, 1H), 3.75 (s, 2H), 1.62 (s, 3H), 1.47 (s, 3H).

HRMS for **23:** formula: $C_{15}H_{18}N_3O_4S^+$, calculated: 336.09, found: 336.17.

Comp. 24 (conjugate of 23 and chlorodactyloferrin [2] in its iron- and gallium-complexed forms)

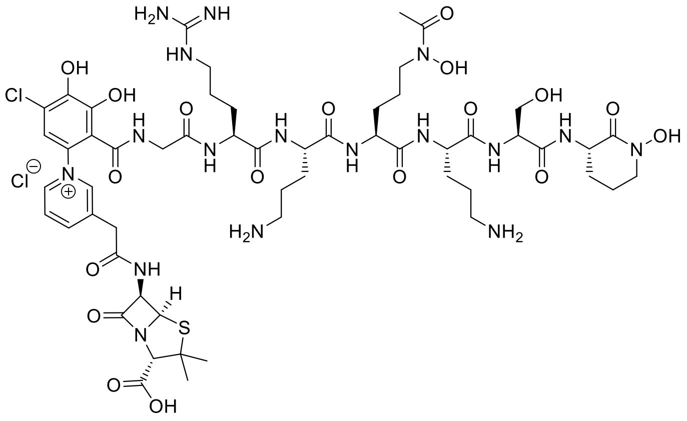

Using chlorodactyloferrin **2** (10 mg, 0.01 mmol), comp. **23** (3.3 mg, 0.01 mmol) and iron trichloride (16.3 mg, 0.1 mmol) as starting compounds, the title compound **24** was prepared in the iron-bound form according to the synthesis of comp. **22**. Yield: 1 mg (7.2 %), brownish solid. For NMR analysis, **24:gallium** complex was generated as described for **22:gallium**.

NMR for **24:gallium**

^{1}H NMR (600 MHz, CD_3OD, 293 K): δ (Final form=hydrolysed form) 8.96 (s, 1H), 8.81 (d, ^{2}J = 5.8 Hz, 1H), 8.62 (d, ^{2}J = 8.1 Hz, 1H), 8.05 (dd, ^{2}J = 5.8&8.1 Hz, 1H). δ (Starting form) 8.98 (s, 1H), 8.86 (d, ^{2}J = 5.8 Hz, 1H), 8.62 (d, ^{2}J = 8.1 Hz, 1H), 8.07 (dd, ^{2}J = 5.8&8.1 Hz, 1H).

HRMS for **24:iron:** formula: $C_{55}H_{78}N_{17}O_{18}SClFe^+$, calculated: 1387.4469, found: 1387.4453.

Comp. 25 (3-pyridyl-rifampicin)

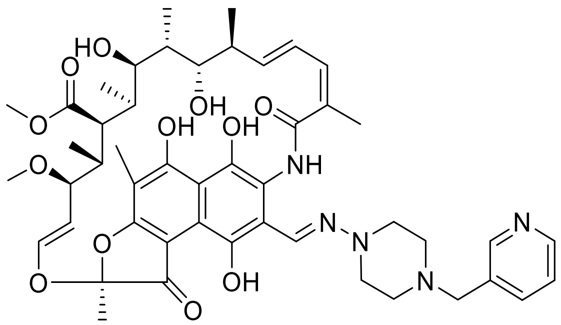

To a red solution of rifaldehyde (Biosynth, 100 mg, 138 µmol, 1.0 eq.) in 300 µL of dry THF was added a solution of 4-(3-pyridylmethyl)piperazin-1-amine (Otava Chemicals, 27 mg, 138 µmol, 1.0 eq.) in 200 µL of dry THF. The dark orange mixture was vigorously stirred 30 min at RT then another equivalent of 4-(3-pyridylmethyl)piperazin-1-amine (27 mg, 138 µmol) in 200 µL of dry THF was added. The reaction was stirred a further 30 min. The mixture was then diluted with 7 mL of DCM and washed with a solution of ascorbic acid (5.5 mL, prepared from 2.0 g of ascorbic acid in 40 mL of 3:1 H_2O/brine). The aqueous layer was then extracted with 7 mL of CH_2Cl_2 and the combined CH_2Cl_2 extracts were dried over $MgSO_4$, filtered and concentrated under reduced pressure to give **25** as an orange solid (109 mg, 88 %).

^{1}H NMR (300 MHz, CDCl3): δ 13.24 (brs, 2H), 13.12 (s, 1H), 12.00 (s, 1H), 8.57-8.51 (m, 2H), 8.27 (s, 1H), 7.76 (d, *J* = 7.8 Hz, 1H), 7.30 (dd, *J* = 7.7 Hz, 4.9 Hz, 1H), 6.54 (dd, *J* = 15.4 Hz, 11.2 Hz, 1H), 6.34 (d, *J* = 10.9 Hz, 1H), 6.20 (dd, *J* = 12.7 Hz, 0.7 Hz, 1H), 5.90 (dd, *J* = 15.4 Hz, 4.9 Hz, 1H), 5.09 (dd, *J* = 12.7 Hz, 6.8 Hz, 1H), 4.94 (d, *J* = 10.6 Hz, 1H), 3.78-3.44 (m, 6H), 3.27-3.09 (m, 4H), 3.03 (s, 3H), 3.02-2.98 (m, 1H), 2.73-2.57 (m, 4H), 2.45-2.31 (m, 1H), 2.21 (s, 3H), 2.05 (s, 6H), 1.79 (s, 3H), 1.74-1.65 (m, 1H), 1.59-1.46 (m, 1H), 1.40-1.27 (m, 1H), 1.01 (d, *J* = 6.8 Hz, 3H), 0.86 (d, *J* = 7.0 Hz, 3H), 0.59 (d, *J* = 6.9 Hz, 3H), -0.32 (d, *J* = 7.0 Hz, 3H).

^{13}C NMR (75 MHz, CDCl3): δ 195.7, 174.6, 172.2, 169.8, 169.3, 150.3, 149.1, 148.2, 142.9, 142.8, 138.7, 137.2, 135.2, 135.1, 132.7, 129.5, 123.7, 123.3, 120.6, 118.8, 118.1, 113.1, 110.8, 108.9, 106.4, 104.7, 77.0, 76.9, 74.5, 70.7, 59.7, 57.2, 51.7 (2C), 50.2 (2C), 39.6, 38.7, 37.6, 33.5, 21.6, 20.87, 20.85, 17.9, 11.0, 9.1, 8.6, 7.7.

HRMS for **25:** formula: $C_{48}H_{62}N_5O_{12}$+, calculated: 900.4395, found: 900.4393.

Comp. 26 (conjugate of 25 and chlorodactyloferrin [2] in its iron- and gallium-complexed forms)

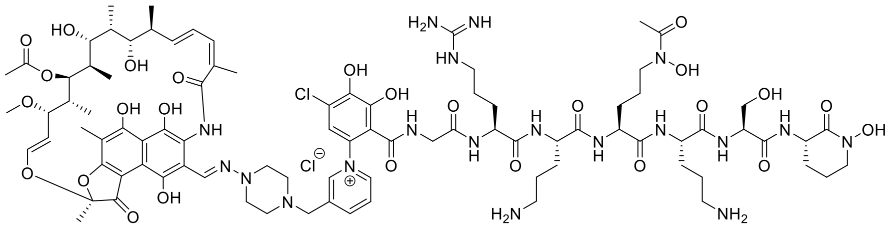

Comp. **28:iron** was generated under nitrogen atmosphere, where to an ice-cold solution of **2** (10 mg, 0.01 mmol) and **25** (9 mg, 0.01 mmol) in water/acetonitrile 1:1 (1 ml), iron trichloride was added at 100 equivalents (162 mg, 1 mmol). A sudden change of the color to dark red was observed due to oxidation of the formed **28:iron** to its quinone form. Reduction of the quinone form back to product **[28]:iron** was done by adding 100 equivalents of ascorbic acid (176 mg, 1 mmol). Purification of the conjugate, and formation of the **28:gallium** complex was carried out as described above for **22**. Yield: 4.2 mg (22 %).

NMR for **26:gallium**

^{1}H NMR (600 MHz, CD_3OD, 293 K): δ 8.90 (s, 1H), 8.86 (d, ^3J = 5.7 Hz, 1H), 8.66 (d, ^3J = 8.5 Hz, 1H), 8.33 (s, 1H), 8.09 (m, 1H), 6.93 (s, 1H), 6.65 (dd, ^3J = 11.2&15.4 Hz, 1H), 6.53 (d, ^3J = 11.2 Hz, 1H), 6.32 (dd, ^3J = 12.8 Hz, ^4J = 0.7 Hz, 1H), 6.00 (dd, ^3J = 5.0&15.4 Hz, 1H), 5.14 (d, ^3J = 10.9 Hz, 1H), 5.09 (dd, ^3J = 8.0&12.8 Hz, 1H),

3.90 (d, ^2J = 14.7 Hz, 1H), 3.85 (m, 1H), 3.82 (d, ^2J = 14.7 Hz, 1H), 3.40 (d, ^3J = 8.0 Hz, 1H), 3.22 (m, 2H), 3.14 (m, 2H), 3.11 (dd, ^3J = 2.3&10.3 Hz, 1H), 3.01 (s, 3H), 2.76 (m, 2H), 2.72 (m, 2H), 2.36 (m, 1H), 2.23 (s, 3H), 2.07 (s, 3H), 2.04 (s, 3H), 1.75 (s, 3H), 1.69 (m, 1H), 1.43 (m, 1H), 1.16 (m, 1H), 0.99 (d, ^3J = 7.1 Hz, 3H), 0.95 (d, ^3J = 7.1 Hz, 3H), 0.62 (d, ^3J = 7.0 Hz, 3H), -0.35 (d, ^3J = 6.9 Hz, 1H).
^{13}C{^{1}H} NMR (151 MHz, CD_3OD, 293 K): δ 196.8, 175.1, 172.6, 171.4, 168.6, 155.7, 149.2, 147.7, 147.5, 146.4, 145.1, 143.6, 140.9, 136.3, 135.6, 131.3, 129.6, 128.5, 125.3, 121.5, 121.0, 119.0, 115.8, 114.7, 112.9, 111,0, 107.', 78.2, 78.2, 75.6, 73.0, 59.2, 57.1, 53.2, 51.6, 42.2, 40.2, 39.9, 34.6, 22.5, 21.1, 21.1, 18.7, 11.0, 9.7, 9.4, 7.8.

HRMS for **26:iron:** formula: $C_{88}H_{122}N_{19}O_{26}ClFe+$, calculated: 1951.7846, found: 1951.7870.

Comp. 27 (3-pyridyl-norfloxacin)

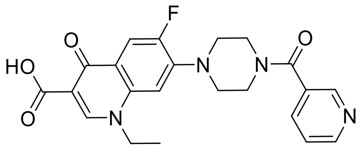

The title compound was obtained from Ambinter (Amb20035724, 1-ethyl-6-fluoro-7-(4-nicotinoylpiperazin-1-yl)-4-oxo-1,4-dihydroquinoline-3-carboxylic acid).

Comp. 28 (conjugate of 27 and chlorodactyloferrin [2] in its iron-, and gallium-complexed forms)

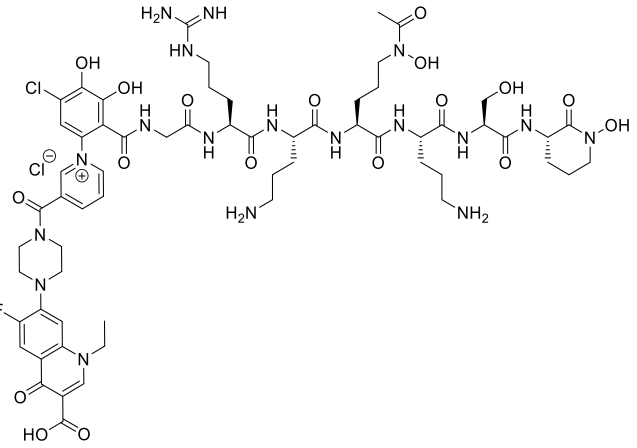

Using **2** (6 mg, 0.006 mmol), **27** (2.54 mg, 0.006 mmol) and iron trichloride (9.7 mg, 0.06 mmol) as starting compounds, the title compound **28:iron** was prepared as a brownish solid according to the synthesis of **22**. Yield: 2.9 mg (33 %). The respective **28:gallium** was prepared for NMR analysis as described for **22:gallium**.

NMR for **28:gallium**

^{1}H NMR (600 MHz, CD_3OD, 293 K): δ 9.23 (s, 1H), 9.07 (d, ^2J = 5.4 Hz, 1H), 8.89 (s, 1H), 8.81 (d, ^2J = 8.1 Hz, 1H), 8.24 (t, ^2J = 5.4&8.1 Hz, 1H), 8.06 d, ^3J(^{1}H-^{19}F) = 13.1 Hz, 1H), 7.22 (d, ^4J = 6.8 Hz, 1H), 6.98 (s, 1H), 4.55 (q, ^2J = 7.2 Hz, 2H), 3.74 (m, 4H), 3.47 (m, 4H), 1.54 (t, ^2J = 7.2 Hz, 3H).
^{13}C{^{1}H/^{19}F} NMR (151 MHz, CD_3OD, 293 K): δ 178.1, 173.5, 169.8, 168.5, 165.0, 155.9, 155.0, 150.0, 148.5, 146.9, 146.9, 146.0, 138.8, 136.5, 129.5, 129.2, 122.5, 119.0, 115.9, 114.4, 113.4, 107.4, 107.3, 50.8, 50.1, 49.3, 14.9.
^{19}F{^{1}H} NMR (565 MHz, CD_3OD, 293 K): δ -76.55 (from TFA), -123.0.

HRMS for **28:iron:** formula: $C_{62}H_{81}ClFFeN_{18}O_{18}+$, calculated: 1476.5076, found: 1476.5092.

References

(1) Kinoshita, M.; Nakata, M.; Takarada, K.; Tatsuta, K. Total Synthesis of Pyridomycin. *Tetrahedron Lett.* **1989**, *30* (52), 7419–7422. https://doi.org/10.1016/S0040-4039(00)70713-1.

(2) Rubino, M. T.; Maggi, D.; Laghezza, A.; Loiodice, F.; Tortorella, P. Identification of Novel Matrix Metalloproteinase Inhibitors by Screening of Phenol Fragments Library. *Arch. Pharm. (Weinheim).* **2011**, *344* (9), 557–563. https://doi.org/10.1002/ardp.201000350.

(3) Aoki, T.; Yoshizawa, H.; Yamawaki, K.; Yokoo, K.; Sato, J.; Hisakawa, S.; Hasegawa, Y.; Kusano, H.; Sano, M.; Sugimoto, H.; et al. Cefiderocol (S-649266), A New Siderophore Cephalosporin Exhibiting Potent Activities against Pseudomonas Aeruginosa and Other Gram-Negative Pathogens Including Multi-Drug Resistant Bacteria: Structure Activity Relationship. *Eur. J. Med. Chem.* **2018**, *155*, 847–868. https://doi.org/10.1016/j.ejmech.2018.06.014.

(4) Jia, X.; Wang, Z.; Xia, C.; Ding, K. Spiroketal-Based Phosphorus Ligands for Highly Regioselective Hydroformylation of Terminal and Internal Olefins. *Chem. - A Eur. J.* **2012**, *18* (48), 15288–15295. https://doi.org/10.1002/chem.201203042.

(5) Weitl, F. L.; Raymond, K. N. 1,5,9-Triazacyclotridecane. *Synthesis (Stuttg).* **1979**, 2728–2731.

(6) Adolphs, M.; Taraz, K.; Budzikiewicz, H. Catecholate Siderophores from Chryseomonas Luteola. *Zeitschrift fur Naturforsch. Sect. C - J. Biosci.* **1996**, *51* (5–6), 281–285. https://doi.org/10.1515/znc-1996-5-603.

(7) Cavalieri, E. L.; Li, K. M.; Balu, N.; Saeed, M.; Devanesan, P.; Higginbotham, S.; Zhao, J.; Gross, M. L.; Rogan, E. G. Catechol Ortho-Quinones: The Electrophilic Compounds That Form Depurinating DNA Adducts and Could Initiate Cancer and Other Diseases. *Carcinogenesis* **2002**, *23* (6), 1071–1077. https://doi.org/10.1093/carcin/23.6.1071.

(8) Demaerel, J.; Veryser, C.; De Borggraeve, W. M. Ex Situ Gas Generation for Lab Scale Organic Synthesis. *React. Chem. Eng.* **2020**, *5* (4), 615–621. https://doi.org/10.1039/c9re00497a.
