# Supplementary material for: A Novel Natural Siderophore Antibiotic Conjugate Reveals a Chemical Approach to Macromolecule Coupling
Source: ACS Cent Sci. 2023 Nov 10;9(11):2138–49. doi: 10.1021/acscentsci.3c00965 (PMC10683483; doi:10.1021/acscentsci.3c00965)
Supplement: Supplementary file 3 — oc3c00965_si_003.pdf [file oc3c00965_si_003.pdf]

Supplementary data

HRMS and NMR data

A novel natural siderophore antibiotic conjugate reveals a chemical approach to macromolecule coupling

Thibault CARADEC[1+], Ernesto ANOZ-CARBONELL[1+], Ravil PETROV[1+], Muriel BILLAMBOZ[2,3], Kevin ANTRAYGUES[4], Francois-Xavier CANTRELLE[2,5], Emmanuelle BOLL[2,5], Delphine BEURY[6], David HOT[6], Herve DROBECQ[1], Xavier TRIVELLI[7], Ruben C. HARTKOORN[1*]

[+] Joint first authorship (contributed equally)
** Corresponding Author, Ruben C. Hartkoorn: Email: ruben.hartkoorn@inserm.fr*

[1] Univ. Lille, CNRS, Inserm, CHU Lille, Institut Pasteur Lille, U1019 - UMR 9017 - CIIL - Center for Infection and Immunity of Lille, F-59000 Lille, France.
[2] Univ. Lille, Inserm, CHU Lille, Institut Pasteur de Lille, U1167 - RID-AGE - Risk Factors and Molecular Determinants of Aging-Related Diseases, F-59000 Lille, France.
[3] JUNIA, Health and Environment, Laboratory of Sustainable Chemistry and Health, Lille, F-59000, France
[4] Univ. Lille, Inserm, Institut Pasteur de Lille, U1177 - Drugs and Molecules for Living Systems, F-59000, Lille, France.
[5] CNRS, EMR9002 BSI Integrative Structural Biology, 59000 Lille,
[6] Univ. Lille, CNRS, Inserm, CHU Lille, Institut Pasteur de Lille, UMR2014 - US41 - PLBS-Plateformes Lilloises de Biologie & Santé, F-59000, Lille, France.
[7] Univ. Lille, CNRS, INRAE, Centrale Lille, Univ. Artois, FR 2638 - IMEC - Institut Michel-Eugène Chevreul, 59000, Lille, France.

Table of Contents

NMR and HRMS Compound 1, Pyridomycin Bruker-300 .... S6

A) Comp 1: ^{1}H NMR of 1 in MeOH-d4, Bruker-300 .... *S6*
B) Comp 1: ^{13}C jmod NMR of 1 in MeOH-d4, Bruker-300 .... *S6*
C) Comp 1: HRMS (ES+) analysis *S7*

NMR and HRMS Compound 2, Chlorodactyloferrin Bruker-600 .... S8

A) Comp 2: 1D-^{1}H-wet with 1 ms pulses NMR spectrum at 14 T and 293 K in H_2O/D_2O (90/10 v/v). *S8*
B) Comp 2: 2D-^{1}H-COSY NMR spectrum at 14 T and 293 K in H_2O/D_2O (90/10 v/v). *S8*
C) Comp 2: 2D-^{1}H-TOCSY (Tm = 58 ms) NMR spectrum at 14 T and 293 K in H_2O/D_2O (90/10 v/v). *S8*
D) Comp 2: 2D-^{1}H-ROESY (Tm = 400 ms) NMR spectrum at 14 T and 293 K in H_2O/D_2O (90/10 v/v). *S9*
E) Comp 2: 2D-^{1}H-^{13}C-HSQC-DEPT NMR spectrum at 14 T and 293 K in H_2O/D_2O (90/10 v/v). *S9*
G) Comp 2: 1D-^{1}H-wet with 20 ms pulses NMR spectrum at 14 T and 293 K in D_2O. *S10*
H) Comp 2: 2D-^{1}H-ROESY (Tm = 400 ms) NMR spectrum at 14 T and 293 K in D_2O. *S10*
I) Comp 2: 2D-1H-13C-HMBC (Japp = 10 Hz) NMR spectrum at 14 T and 293 K in D_2O. *S10*
J) Comp 2: 2D-1H-15N-HMBC (Japp = 3 Hz) NMR spectrum at 14 T and 293 K in D_2O. *S11*
K) Comp 2: HRMS (ES+) analysis *S11*

NMR and HRMS Compound 2:Gallium, Chlorodactyloferrin gallium complex, Bruker-600 .... S12

A) Comp 2:gallium, 1D-^{1}H-wet with 1 ms pulses NMR spectrum at 14 T and 293 K in H_2O/D_2O (90/10 v/v). *S12*
B) Comp 2:gallium, 2D-^{1}H-TOCSY (Tm = 117 ms) NMR spectrum at 14 T and 293 K in H_2O/D_2O (90/10 v/v). *S12*
C) Comp 2:gallium, 2D-^{1}H-ROESY (Tm = 400 ms) NMR spectrum at 14 T and 293 K in H_2O/D_2O (90/10 v/v). *S13*
D) Comp 2:gallium, 2D-^{1}H-^{15}N-HSQC NMR spectrum at 14 T and 293 K in H_2O/D_2O (90/10 v/v). *S13*
E) Comp 2:gallium, 2D-^{1}H-^{13}C-HSQC-DEPT NMR spectrum at 14 T and 293 K in D_2O. *S13*
F) Comp 2:gallium, 2D-^{1}H-^{13}C-HSQC-TOCSY (Tm = 80 ms) NMR spectrum at 14 T and 293 K in D_2O. *S14*
G) Comp 2:gallium, 2D-^{1}H-^{13}C-HMBC (Japp = 10 Hz) NMR spectrum at 14 T and 293 K in D_2O. *S14*
H) Comp 2:gallium, 2D-^{1}H-^{15}N-HMBC (Japp = 3 Hz) NMR spectrum at 14 T and 293 K in D_2O. *S14*
I) Comp 2:iron, HRMS (ES+) analysis *S15*

NMR and HRMS Compound 3, Dactyloferrin Bruker-600 .... S16

A) Comp 3: 1D-^{1}H-wet with 1 ms pulses NMR spectrum at 14 T and 293 K in H_2O/D_2O (90/10 v/v). *S16*
B) Comp 3: 2D-^{1}H-COSY NMR spectrum at 14 T and 293 K in H_2O/D_2O (90/10 v/v). *S16*
C) Comp 3: 2D-^{1}H-TOCSY (Tm = 100 ms) NMR spectrum at 14 T and 293 K in H_2O/D_2O (90/10 v/v). *S16*
D) Comp 3: 2D-^{1}H-ROESY (Tm = 300 ms) NMR spectrum at 14 T and 293 K in H_2O/D_2O (90/10 v/v). *S17*
E) Comp 3:1D-$^{13}C\{^{1}H\}$ NMR spectrum in H_2O/D_2O (90/10 v/v) at 14 T and 293 K. *S17*
F) Comp 3:1D-^{13}C-DEPT135 NMR spectrum in H_2O/D_2O (90/10 v/v) at 14 T and 293 K. *S17*
G) Comp 3: 2D-^{1}H-^{13}C-HSQC-TOCSY (Tm = 77,5 ms) NMR spectrum at 14 T and 293 K in H_2O/D_2O (90/10 v/v). *S18*
H) Comp 3: 2D-^{1}H-^{15}N-HSQC NMR spectrum at 14 T and 293 K in H_2O/D_2O (90/10 v/v). *S18*
I) Comp 3:1D-^{1}H NMR spectrum of Daf [3] in D_2O at 14 T and 293 K. *S18*
J) Comp 3: 2D-^{1}H-^{13}C-HSQC-DEPT NMR spectrum at 14 T and 293 K in D_2O. *S19*
K) Comp 3: 2D-^{1}H-^{13}C-HMBC (Japp = 10 Hz) NMR spectrum at 14 T and 293 K in D_2O. *S19*
L) Comp 3: 2D-^{1}H-^{15}N-HMBC (Japp = 8 Hz) NMR spectrum at 14 T and 293 K in D_2O. *S19*
M) Comp 3 : HRMS (ES+) analysis *S20*
N) Comp 3:iron. HRMS (ES+) analysis *S20*

NMR and HRMS Compound 4 :gallium, Chlorodactyloferrin-Pyridomycin conjugate Bruker-600. S21

A) Comp. 4:gallium: 1D-^{1}H-w5. NMR spectrum in H_2O/CD_3CN (1/1 v/v) at 14 T and 293 K. *S21*
B) Comp. 4:gallium:2D-^{1}H-COSY. NMR spectrum in H_2O/CD_3CN (1/1 v/v) at 14 T and 293 K *S21*
C) Comp. 4:gallium:2D-^{1}H-TOCSY (Tm = 58 ms). NMR spectrum in H_2O/CD_3CN (1/1 v/v) at 14 T and 293 K *S22*
D) Comp. 4:gallium:2D-^{1}H-ROESY (Tm = 400 ms). NMR spectrum in H_2O/CD_3CN (1/1 v/v) at 14 T and 293 K *S22*
E) Comp. 4:gallium:2D-^{1}H-^{13}Caliphatic-HSQC. NMR spectrum in H_2O/CD_3CN (1/1 v/v) at 14 T and 293 K *S22*
F) Comp. 4:gallium:2D-^{1}H-^{13}Caliphatic-HSQC-TOCSY (Tm = 58 ms). NMR spectrum in H_2O/CD_3CN (1/1 v/v) at 14 T and 293 K. *S22*
G) Comp. 4:gallium:2D-^{1}H-^{13}Caromatic-HSQC. NMR spectrum in H_2O/CD_3CN (1/1 v/v) at 14 T and 293 K. *S23*
H) Comp. 4:gallium:2D-^{1}H-^{13}C-HMBC (Japp = 10 Hz). NMR spectrum in H_2O/CD_3CN (1/1 v/v) at 14 T and 293 K *S23*
I) Comp. 4:gallium:2D-^{1}H-^{15}N-HSQC. NMR spectrum in H_2O/CD_3CN (1/1 v/v) at 14 T and 293 K. *S23*
J) Comp 4:iron . HRMS (ES+) analysis *S24*

NMR and HRMS Compound 5, Bruker-300 .... S25
Comp 5. ^{1}H NMR of 5 in $CDCl_3$, Bruker-300 .... *S25*
Comp 5.^{13}C NMR of 5 in $CDCl_3$, Bruker-300 .... *S25*
Comp 5. HRMS (ES+) analysis *S26*
NMR and HRMS Compound 6, Bruker-300 .... S27
Comp 6. ^{1}H NMR of 6 in $CDCl_3$, Bruker-300 .... *S27*
Comp 6. ^{13}C NMR of 6 in DMSO-d_6, Bruker-300 .... *S27*
Comp 6. HRMS (ES+) analysis. *S28*
NMR and HRMS Compound 7, Bruker-300 .... S29
Comp 7. ^{1}H NMR of 7 in $CDCl_3$, Bruker-300 .... *S29*
Comp 7.^{13}C NMR of 7 in $CDCl_3$, Bruker-300 .... *S29*
Comp 7. HRMS (ES+) analysis. *S30*
NMR and HRMS Compound 11, Bruker-300 .... S31
Comp 11. ^{1}H NMR of 11 in DMSO-d_6, Bruker-300 .... *S31*
Comp 11.^{13}C NMR of in 11 in DMSO-d_6, Bruker-300 .... *S31*
Comp 11. HRMS (ES+) analysis *S32*
NMR and HRMS Compound 16, Bruker-300 .... S33
Comp 16a . ^{1}H NMR of 16a in DMSO-d_6, Bruker-300 .... *S33*
Comp 16a.^{13}C NMR of 16a in DMSO-d_6, Bruker-300 .... *S33*
Comp 16a. HRMS (ES+) analysis *S34*
Comp 16b. ^{1}H NMR of 16b in DMSO-d_6, Bruker-300 .... *S35*
Comp 16b. ^{13}C jmod NMR of 16b in DMSO-d_6, Bruker-300 .... *S35*
Comp 16b.HRMS (ESI+) analysis *S36*
Comp 16c. ^{1}H NMR of 16c in DMSO-d_6, Bruker-300 .... *S37*
Comp 16c. ^{13}C NMR of 16c in DMSO-d_6, Bruker-300 .... *S37*
Comp 16c. HRMS (ESI+) analysis. *S38*
NMR and HRMS Compound 17, Bruker-300 .... S39
Comp 17b. ^{1}H NMR of 17b in DMSO-d_6, Bruker-300 .... *S39*
Comp 17b. ^{13}C NMR of 17b in DMSO-d_6, Bruker-300 .... *S39*
Comp 17b. HRMS (ESI+) analysis *S40*
NMR and HRMS Compound 18, Bruker-300 .... S41
Comp 18a. ^{1}H NMR of 18a in DMSO-d_6, Bruker-300 .... *S41*
^{13}C NMR of 18a in DMSO-d_6, Bruker-300 .... *S41*
Comp 18a. HRMS (ES+) analysis *S42*
Comp 18b. ^{1}H NMR of 18b in DMSO-d_6, Bruker-300 .... *S43*
Comp 18b. ^{13}C jmod NMR of 18b in DMSO-d_6, Bruker-300 .... *S43*
Comp 18b. HRMS (ES+) analysis *S44*
Comp 18d. ^{1}H NMR of 18d in MeOH-d_4, Bruker-300 .... *S45*
Comp 18d. ^{13}C NMR of 18d in MeOH-d_4, Bruker-300 .... *S45*
Comp 18d. HRMS (ESI+) analysis *S46*
Comp 18e. ^{1}H NMR of 18e in DMSO-d_6, Bruker-600 .... *S47*
Superimposition of ^{1}H NMR of 18e (blue) vs. 18f (red) in DMSO-d_6, Bruker-600 .... *S47*
2D ^{1}H-COSY NMR of 18e in DMSO-d_6, Bruker-600 .... *S48*
2D ^{1}H-^{13}C-HSQC-DEPT NMR of 18e in DMSO-d_6, Bruker-600 .... *S48*
Superimposition of 2D ^{1}H-^{13}C-HSQC-DEPT NMR of 18e vs. 18f in DMSO-d_6, Bruker-600: CH/CH_3 (red), CH_2 (purple) *S49*
2D ^{1}H-^{13}C-HSQC-TOCSY (T_m = 27 ms) NMR of 18e in DMSO-d_6, Bruker-600 .... *S49*
HRMS (ES+) analysis of 18e *S50*
Comp 18f. ^{1}H NMR of 18f in DMSO-d_6, Bruker-300 .... *S51*
Comp 18f. ^{13}C jmod NMR of 18f in DMSO-d_6, Bruker-300 .... *S51*
Comp 18f. ^{1}H NMR of 18f in DMSO-d_6, Bruker-600 .... *S52*
Comp 18f. 2D ^{1}H-COSY NMR of 18f in DMSO-d_6, Bruker-600 .... *S52*
Comp 18f. 2D ^{1}H-NOESY (T_m = 300 ms) NMR of 18f in DMSO-d_6, Bruker-600 .... *S53*
Comp 18f. ^{13}C NMR of 18f in DMSO-d_6, Bruker-600 .... *S53*
Comp 18f. ^{13}C DEPT-135 NMR of 18f in DMSO-d_6, Bruker-600 .... *S53*
Comp 18f. 2D ^{1}H-^{13}C-HSQC-DEPT of 18f in DMSO-d_6, Bruker-600 .... *S54*

Comp 18f. 2D ^{1}H-^{13}C-HSQC-TOCSY (Tm = 27 ms) of 18f in DMSO-d$_6$, Bruker-600 .... *S54*
Comp 18f. 2D ^{1}H-^{15}N-HSQC NMR of 18f in DMSO-d$_6$, Bruker-600 .... *S54*
Comp 18f. 2D ^{1}H-^{13}C-HMBC (J_{app} = 10 Hz) NMR of 18f in DMSO-d$_6$, Bruker-600 .... *S55*
Comp 18f. 2D ^{1}H-^{15}N-HMBC (J_{app} = 8 Hz) NMR of 18f in DMSO-d$_6$, Bruker-600 .... *S55*
Comp 18f. HRMS (ES+) analysis of 18f *S56*
NMR and HRMS Compound 19, Bruker-300 .... S57
Comp 19b. ^{1}H NMR of 19b in DMSO-d$_6$, Bruker-300 .... *S57*
Comp 19b. HRMS (ES+) analysis of comp 19b *S57*
Comp 19c. ^{1}H NMR of 19c in DMSO-d$_6$, Bruker-300 .... *S58*
Comp 19c. ^{13}C NMR of 19c in DMSO-d$_6$, Bruker-300 .... *S58*
Comp 19c. HRMS (ES+) analysis *S59*
NMR and HRMS Compound 20, Bruker-300 .... S60
Comp 20b. HRMS (ES+) analysis *S60*
Comp 20c. ^{1}H NMR of 20c in DMSO-d$_6$, Bruker-300 .... *S61*
Comp 20c. ^{13}C NMR of 20c in DMSO-d$_6$, Bruker-300 .... *S61*
Comp 20c. HRMS (ES+) analysis *S62*
NMR and HRMS Compound 21, 3-pyridyl TAMRA Bruker-300 .... S63
Comp 21. ^{1}H NMR of 21 in DMSO-d$_6$, Bruker-300 .... *S63*
Comp 21. HRMS (ES+) analysis *S63*
NMR and HRMS Compound 22:gallium, Chlorodactyloferrin-TAMRA gallium complex Bruker-600 .... S64
A) Comp 22:galium: 1D-^{1}H. NMR spectrum in CD_3OD at 14 T and 293 K. *S64*
B) Comp 22:galium:2D-^{1}H-ROESY (Tm = 400 ms). NMR spectrum in CD_3OD at 14 T and 293 K. *S64*
C) Comp 22:galium:2D-^{1}H-^{13}Caromatic-HSQC. NMR spectrum in CD_3OD at 14 T and 293 K. *S65*
D) Comp 22:galium:2D-^{1}H-^{13}C-HSQC-TOCSY (Tm = 59 ms). NMR spectrum in CD_3OD at 14 T and 293 K. *S65*
E) Comp 22:galium:2D-^{1}H-^{13}C-HMBC (Japp = 10 Hz). NMR spectrum in CD_3OD at 14 T and 293 K. *S65*
F) Comp 22:galium: 2D-^{1}H-^{15}N-HMBC (Japp = 8 Hz). NMR spectrum in CD_3OD at 14 T and 293 K. *S65*
G) Comp 22:iron: HRMS (ES+) analysis *S66*
NMR and HRMS Compound 23: 3-pyridyl penicillin Bruker-300 .... S67
Comp 23-1: intermediate 1 ^{1}H NMR of 23-1 in DMSO-d$_6$, Bruker-300 .... *S67*
Comp 23-1: intermediate 1 ^{13}C NMR of 23-1 in DMSO-d$_6$, Bruker-300 .... *S67*
Comp 23-2 intermediate 2: ^{1}H NMR of 23-2 in $CDCl_3$, Bruker-300 .... *S68*
Comp 23-2 intermediate 2: ^{13}C NMR of 23-2 in $CDCl_3$, Bruker-300 .... *S68*
Comp 23-2 intermediate 2: HRMS (ES+) analysis *S69*
Comp 23. ^{1}H NMR of 26 in DMSO-d$_6$, Bruker-300 .... *S70*
NMR and HRMS Compound 24:gallium, Chlorodactyloferrin-penicillin gallium complex Bruker-600 .... S71
A) Comp. 24:gallium: 1D-^{1}H Final. NMR spectrum in CD_3OD at 14 T and 293 K. *S71*
B) Comp. 24:gallium: 1D-^{1}H at t_0, t_0+3,25hours, t_0+1day21hours, t_0+6days+20hours (from bottom to top). NMR spectra in CD_3OD at 14 T and 293 K. *S71*
Comp. 24::iron. HRMS (ES+) analysis *S72*
NMR and HRMS Compound 25: 3-pyridyl rifampicin Bruker-300 .... S73
Comp 25. ^{1}H NMR spectrum in $CDCl_3$ *S73*
Comp 25. ^{13}C NMR spectrum *S74*
Comp 25. HRMS (ESI+) analysis *S75*
NMR and HRMS Compound 26: Chlorodactyloferrin-3-pyridyl rifampicin gallium complex Bruker-600 .... S76
A) Comp. 26:gallium: 1D-^{1}H wet (11ms). NMR spectrum in CD_3OD at 14 T and 293 K. *S76*
B) Comp. 26:gallium: 2D-^{1}H-ROESY (Tm = 400 ms). NMR spectrum in CD_3OD at 14 T and 293 K. *S76*
C). Comp. 26:gallium: 2D-^{1}H-^{13}Caliphatic-HSQC. NMR spectrum in CD_3OD at 14 T and 293 K. *S77*
D). Comp. 26:gallium: 2D-^{1}H-^{13}Caromatic-HSQC. NMR spectrum in CD_3OD at 14 T and 293 K. *S77*
E) Comp. 26:gallium: 2D-^{1}H-^{13}C-HMBC (Japp = 10 Hz). NMR spectrum in CD_3OD at 14 T and 293 K. *S77*
Comp 26:iron. HRMS (ESI+) analysis *S78*
NMR and HRMS Compound 28: Chlorodactyloferrin 3-pyridyl norfloxacin gallium complex Bruker-600 .... S79
A) Comp 28:gallium: 1D-^{1}H. NMR spectrum in CD_3OD at 14 T and 293 K. *S79*

B) Comp 28:gallium: 2D-^{1}H-ROESY (Tm = 400 ms). NMR spectrum in CD_3OD at 14 T and 293 K.......................S79
C). Comp 28:gallium: 2D-^{1}H-^{13}Caliphatic-HSQC. NMR spectrum in CD_3OD at 14 T and 293 K...........................S80
D). Comp 28:gallium: 2D-^{1}H-^{13}Caromatic-HSQC. NMR spectrum in CD_3OD at 14 T and 293 K...........................S80
E) Comp 28:gallium: 2D-^{1}H-^{13}C-HMBC (Japp = 10 Hz). NMR spectrum in CD_3OD at 14 T and 293 K..................S80
F) Comp 28:gallium: 1D-^{19}F{^{1}H}. NMR spectrum in CD_3OD at 14 T and 293 K. S81
Comp 28:iron. HRMS (ESI+) analysis S81

NMR and HRMS Compound 1, Pyridomycin Bruker-300

A) Comp 1: ^{1}H NMR of 1 in MeOH-d4, Bruker-300

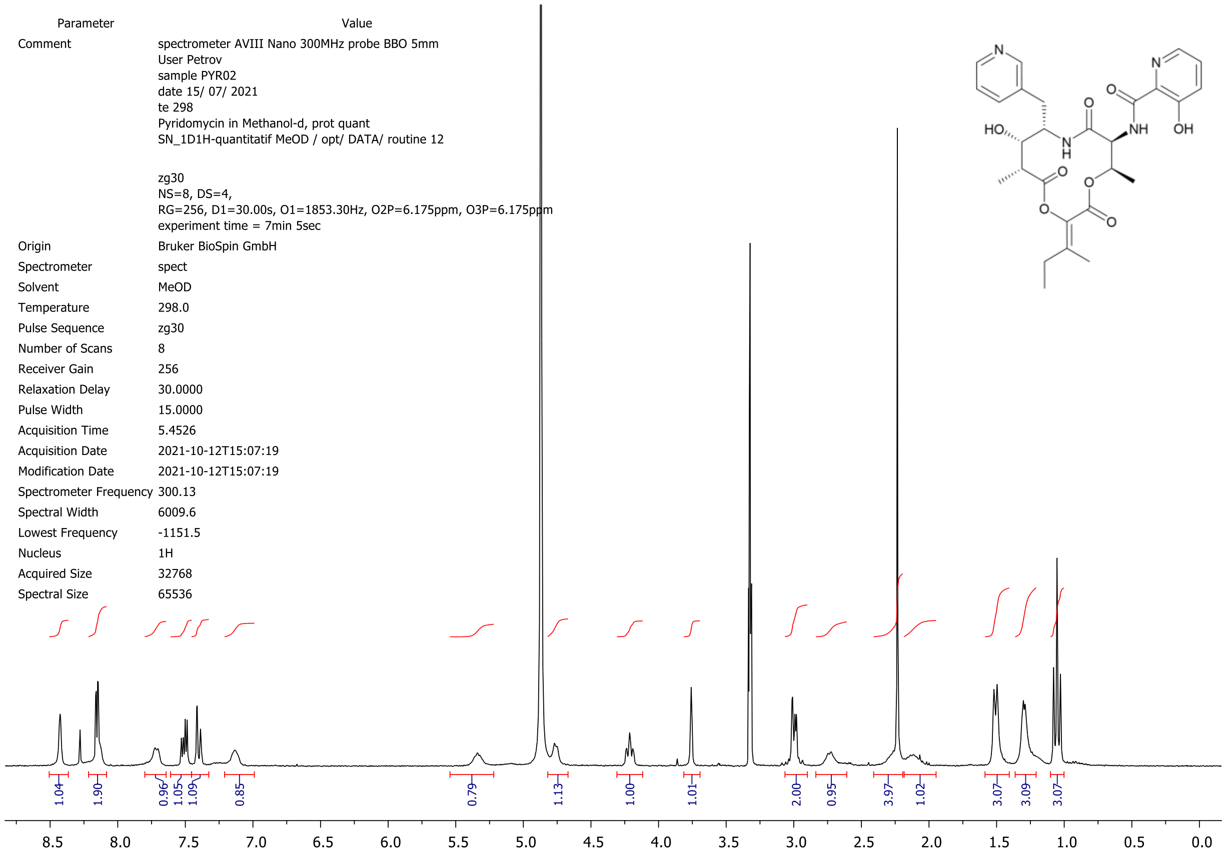

B) Comp 1: ^{13}C jmod NMR of 1 in MeOH-d4, Bruker-300

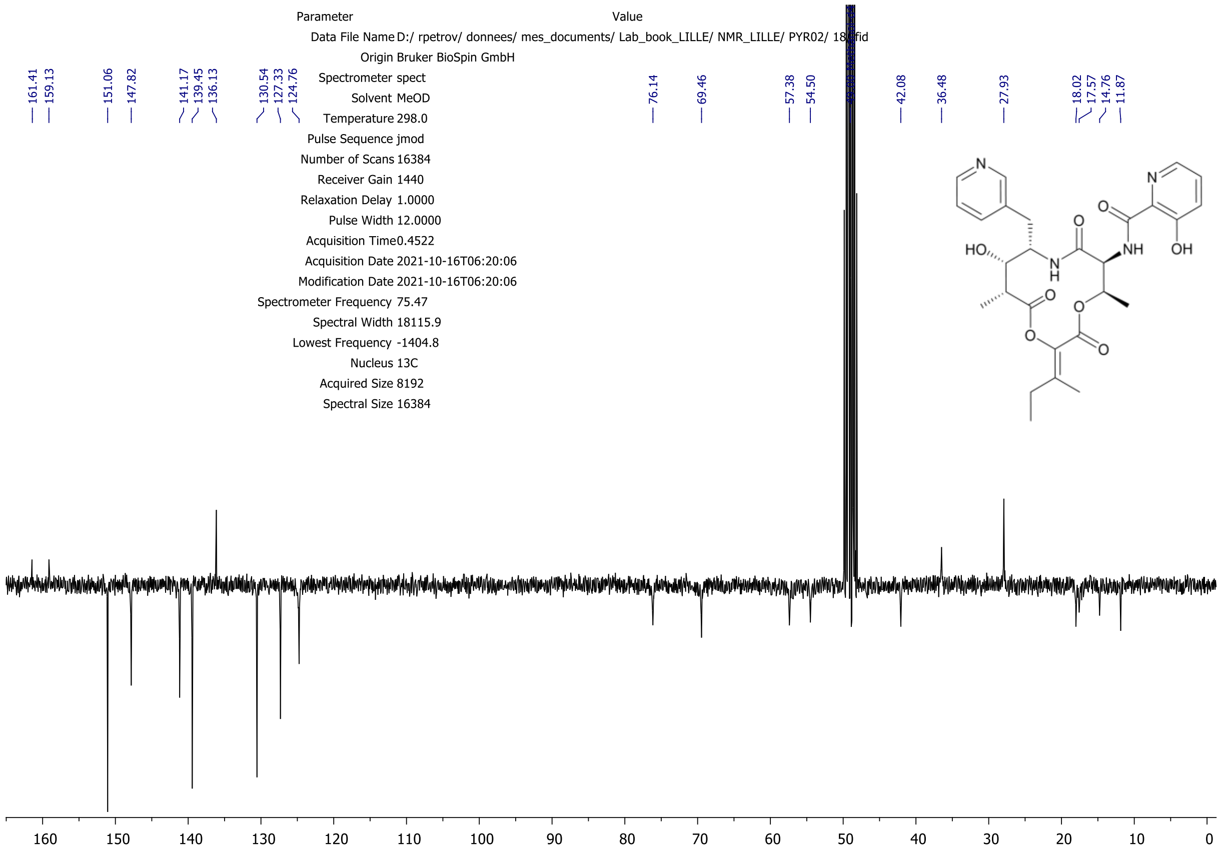

C) Comp 1: HRMS (ES+) analysis

Single Mass Analysis

Tolerance = 10.0 mDa / DBE: min = -1.5, max = 50.0

Element prediction: Off

Number of isotope peaks used for i-FIT = 3

Monoisotopic Mass, Even Electron Ions

91 formula(e) evaluated with 1 results within limits (up to 50 best isotopic matches for each mass)

Elements Used:

C: 0-30 H: 0-40 N: 0-5 O: 0-10

Pyr 96 (2.565)

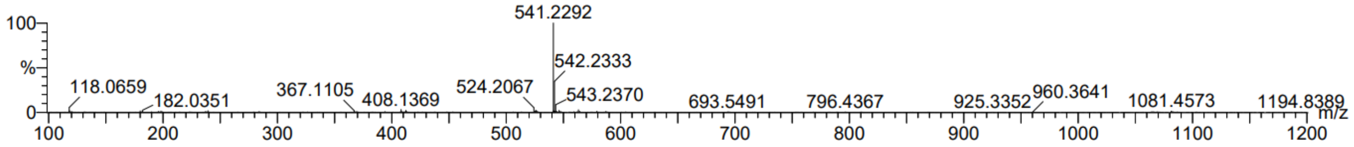

	Calc. Mass	mDa	PPM	DBE	i-FIT	Formula
Minimum:				-1.5		
Maximum:		10.0	10.0	50.0		
Mass	Calc. Mass	mDa	PPM	DBE	i-FIT	Formula
541.2292	541.2298	-0.6	-1.1	13.5	26.5	C27 H33 N4 O8

NMR and HRMS Compound 2, Chlorodactyloferrin Bruker-600

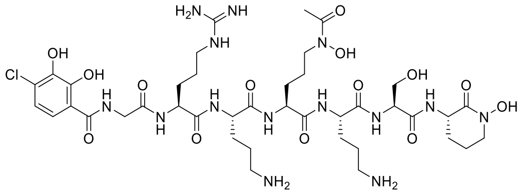

A) Comp 2: 1D-^{1}H-wet with 1 ms pulses NMR spectrum at 14 T and 293 K in H_2O/D_2O (90/10 v/v).

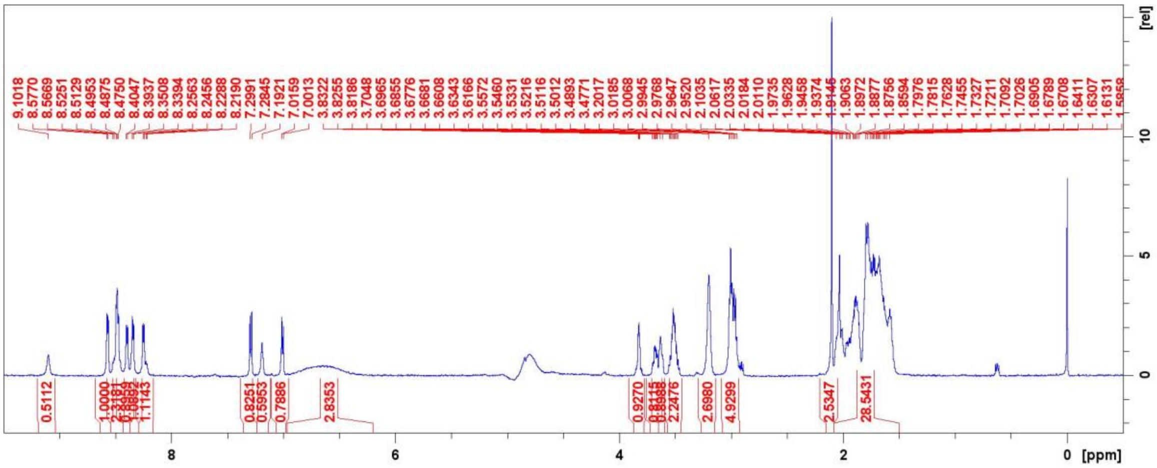

B) Comp 2: 2D-^{1}H-COSY NMR spectrum at 14 T and 293 K in H_2O/D_2O (90/10 v/v).

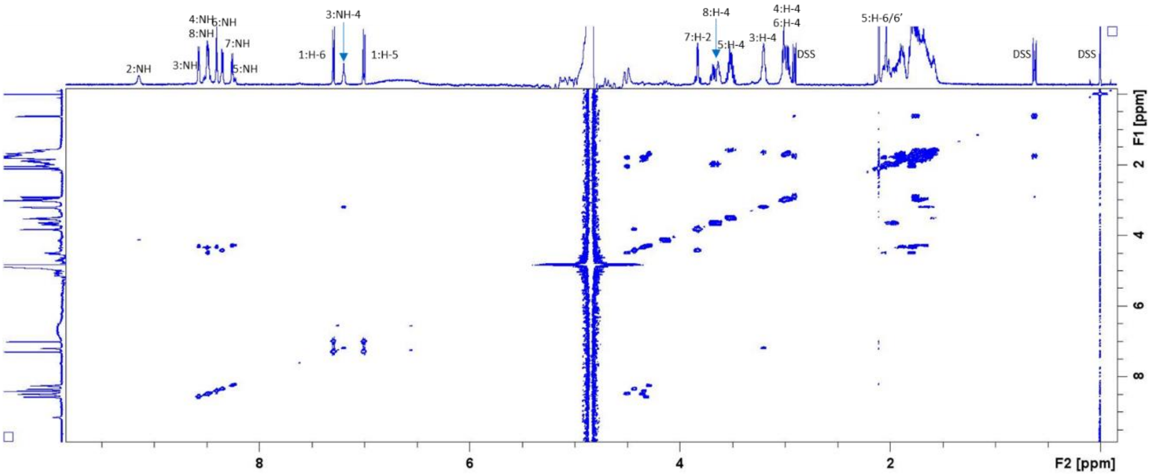

C) Comp 2: 2D-^{1}H-TOCSY (*T*m = 58 ms) NMR spectrum at 14 T and 293 K in H_2O/D_2O (90/10 v/v).

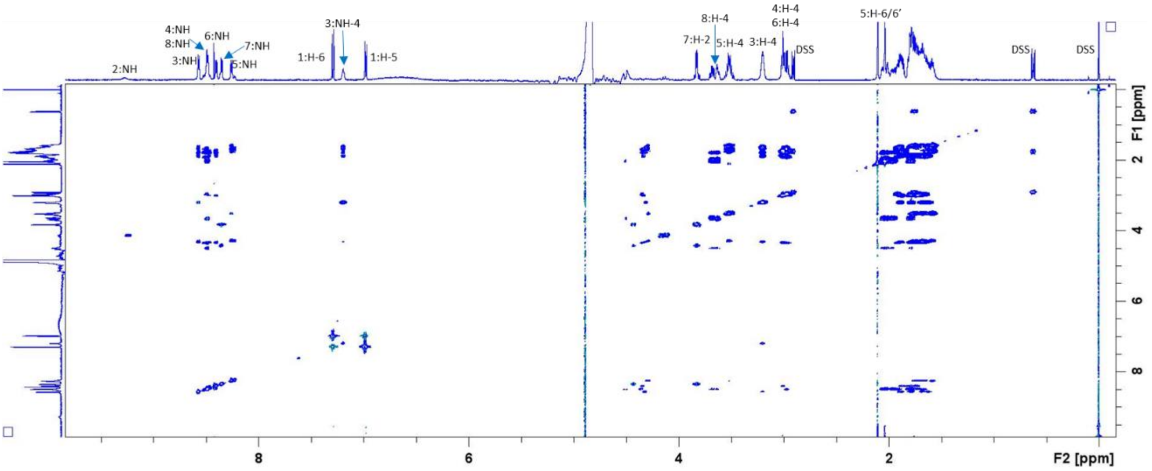

D) Comp 2: 2D-^{1}H-ROESY (Tm = 400 ms) NMR spectrum at 14 T and 293 K in H_2O/D_2O (90/10 v/v).

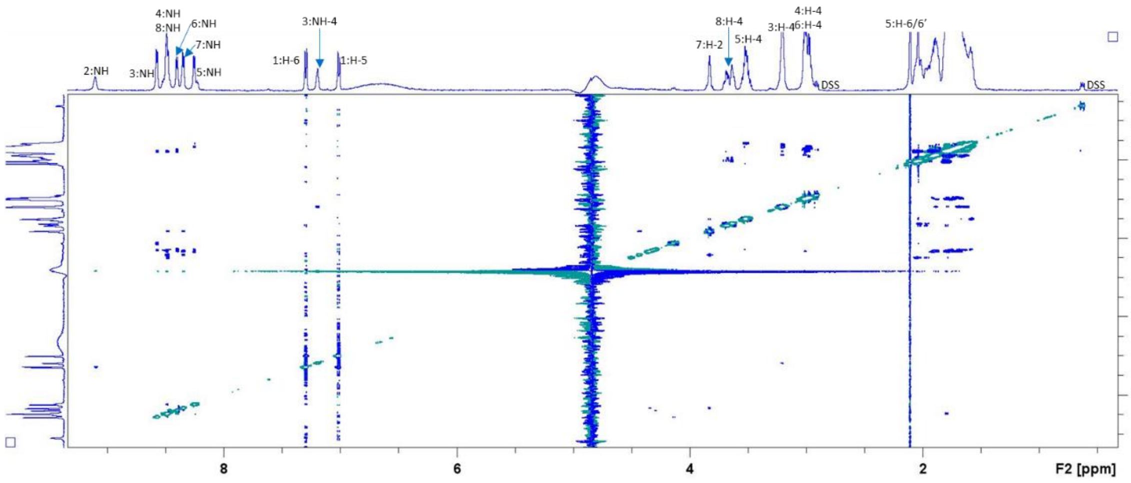

E) Comp 2: 2D-^{1}H-^{13}C-HSQC-DEPT NMR spectrum at 14 T and 293 K in H_2O/D_2O (90/10 v/v).

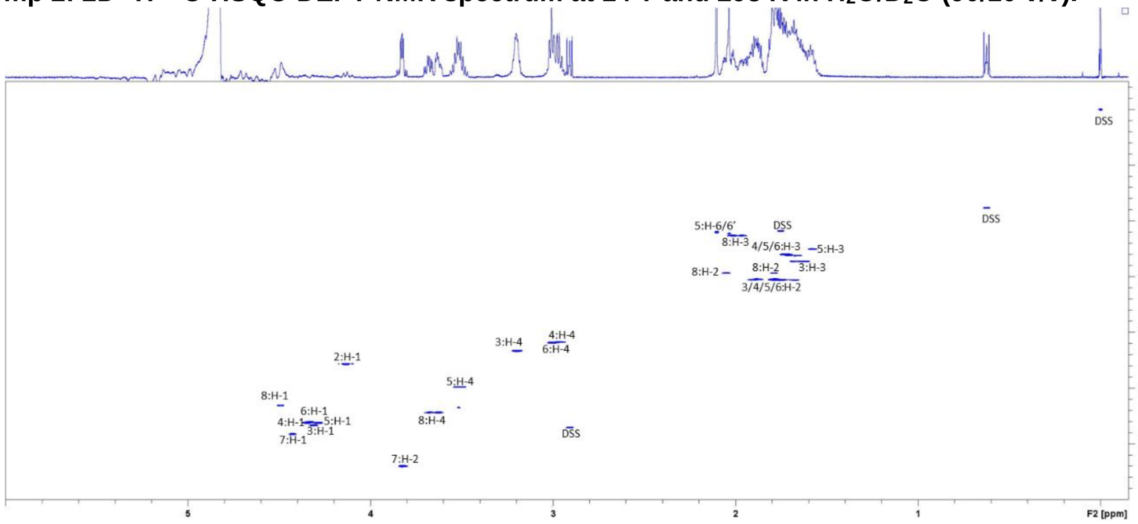

F) Comp 2: 2D-^{1}H-^{15}N-HSQC NMR spectrum at 14 T and 293 K in H_2O/D_2O (90/10 v/v).

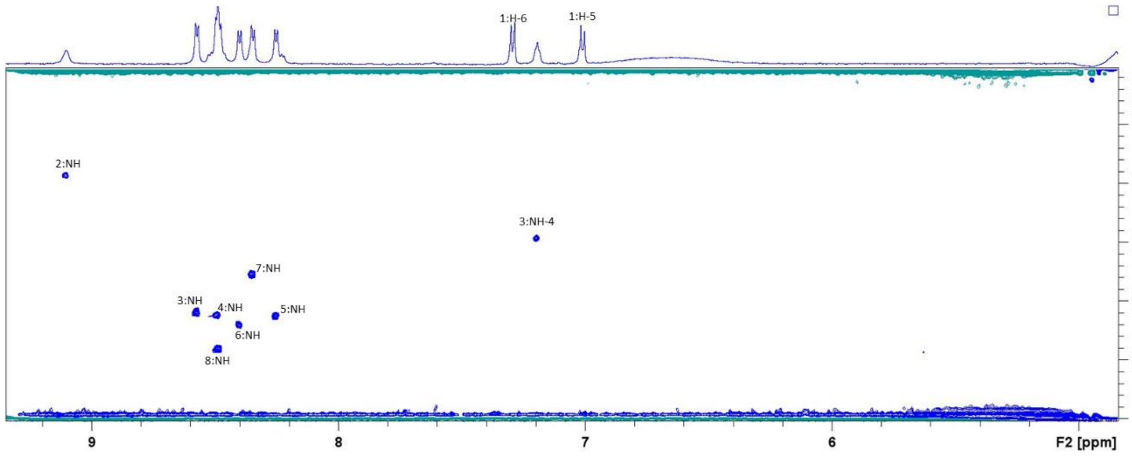

G) Comp 2: 1D-^{1}H-wet with 20 ms pulses NMR spectrum at 14 T and 293 K in D_2O.

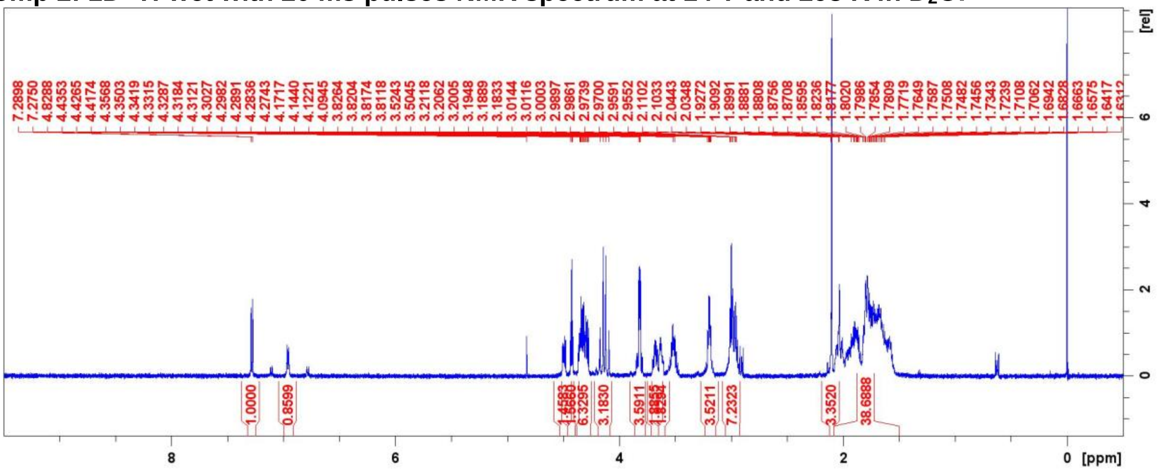

H) Comp 2: 2D-^{1}H-ROESY (*T*m = 400 ms) NMR spectrum at 14 T and 293 K in D_2O.

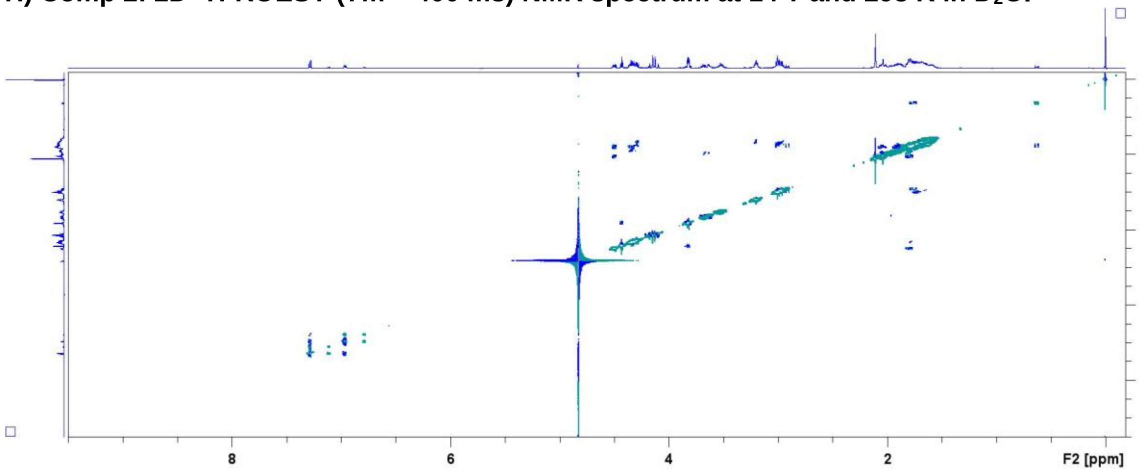

I) Comp 2: 2D-1H-13C-HMBC (*J*app = 10 Hz) NMR spectrum at 14 T and 293 K in D_2O.

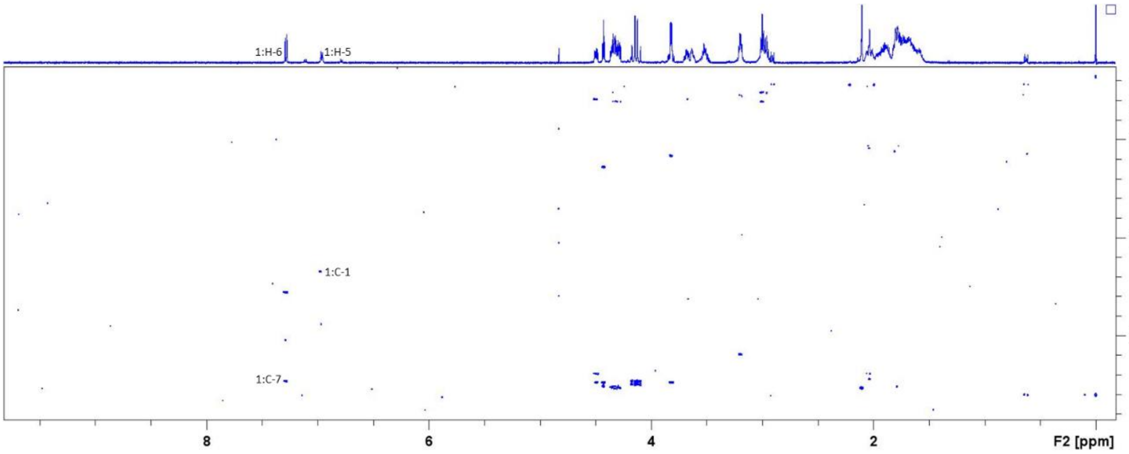

J) Comp 2: 2D-1H-15N-HMBC (*J*app = 3 Hz) NMR spectrum at 14 T and 293 K in D_2O.

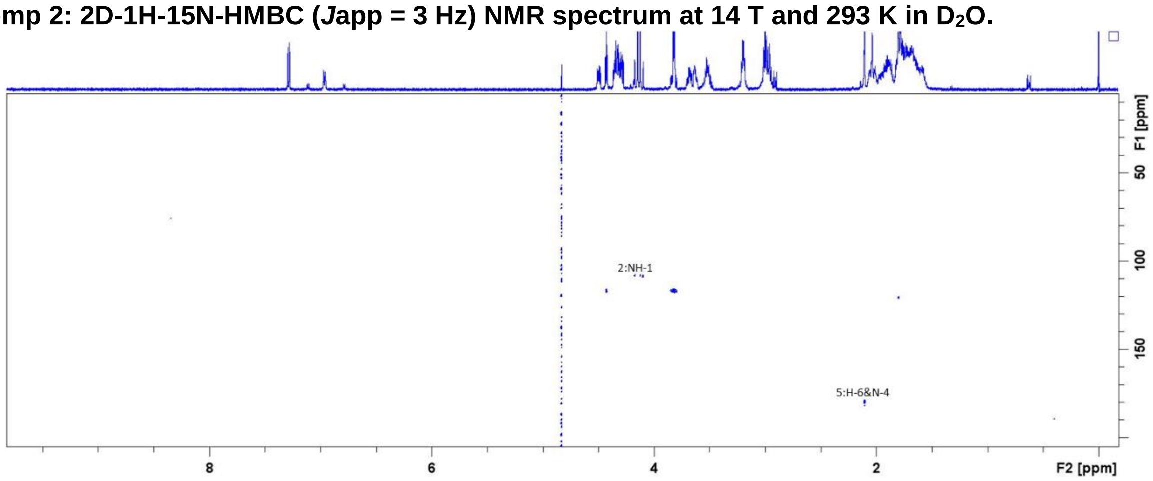

K) Comp 2: HRMS (ES+) analysis

Single Mass Analysis
Tolerance = 10.0 mDa / DBE: min = -1.5, max = 50.0
Element prediction: Off
Number of isotope peaks used for i-FIT = 2

Monoisotopic Mass, Even Electron Ions
748 formula(e) evaluated with 10 results within limits (up to 50 closest results for each mass)
Elements Used:
C: 0-50 H: 0-70 N: 0-15 O: 0-15 Cl: 0-1

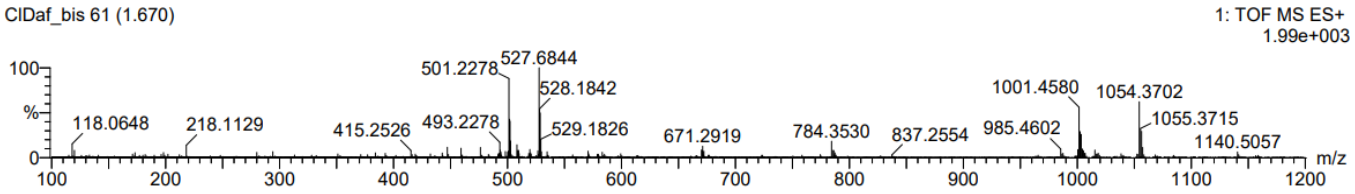

Minimum:				-1.5		
Maximum:		10.0	10.0	50.0		
Mass	Calc. Mass	mDa	PPM	DBE	i-FIT	Formula
1001.4580	1001.4571	0.9	0.9	14.5	0.0	C40 H66 N14 O14 Cl
	1001.4593	-1.3	-1.3	23.5	2.4	C46 H61 N14 O12
	1001.4612	-3.2	-3.2	18.5	1.3	C45 H66 N12 O12 Cl
	1001.4620	-4.0	-4.0	22.5	4.6	C50 H65 N8 O14
	1001.4526	5.4	5.4	17.5	3.3	C50 H70 N4 O15 Cl

NMR and HRMS Compound 2:Gallium, Chlorodactyloferrin gallium complex, Bruker-600

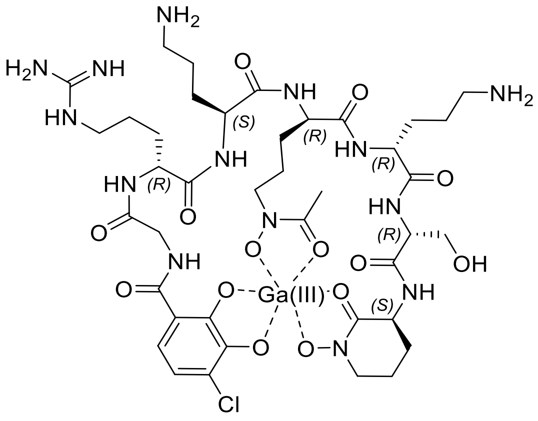

A) Comp 2:gallium, 1D-^{1}H-wet with 1 ms pulses NMR spectrum at 14 T and 293 K in H_2O/D_2O (90/10 v/v).

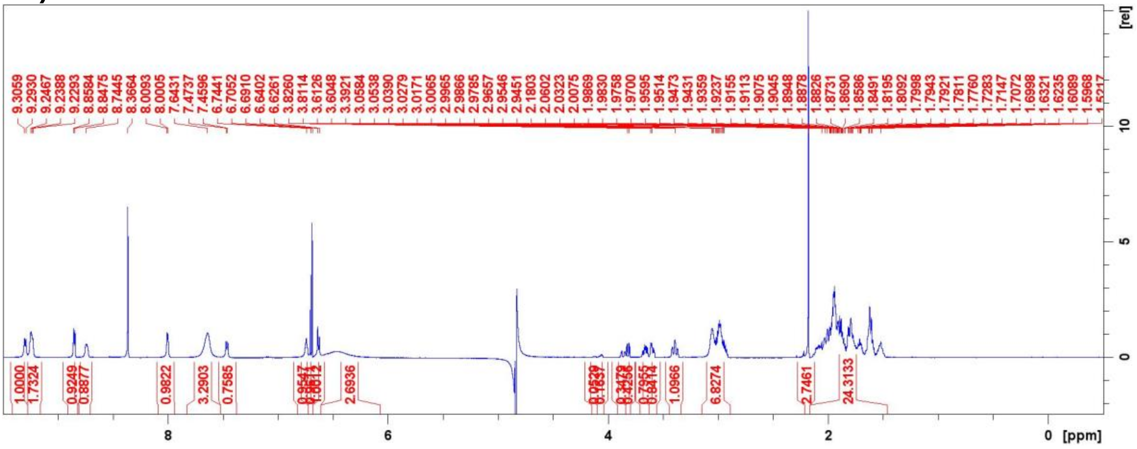

B) Comp 2:gallium, 2D-^{1}H-TOCSY (*T*m = 117 ms) NMR spectrum at 14 T and 293 K in H_2O/D_2O (90/10 v/v).

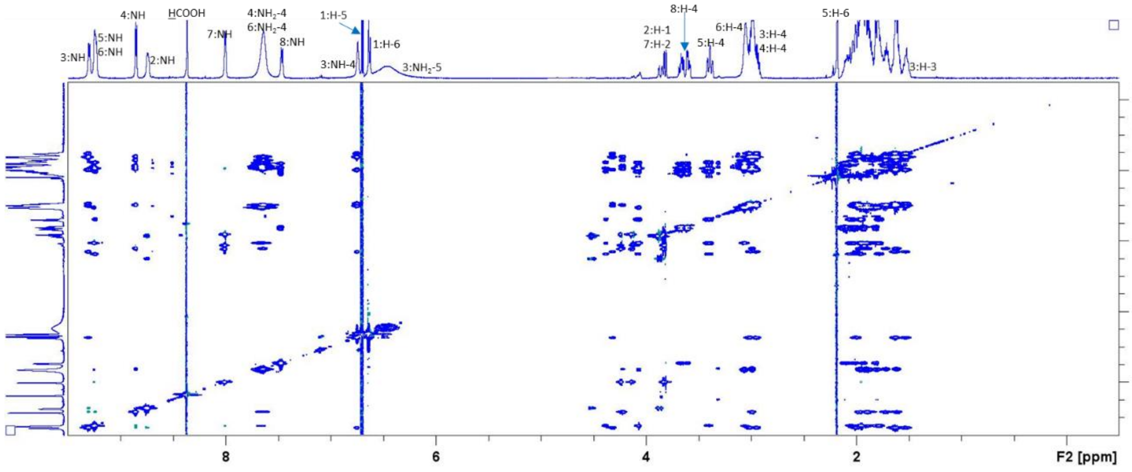

C) Comp 2:gallium, 2D-^{1}H-ROESY (*T*m = 400 ms) NMR spectrum at 14 T and 293 K in H_2O/D_2O (90/10 v/v).

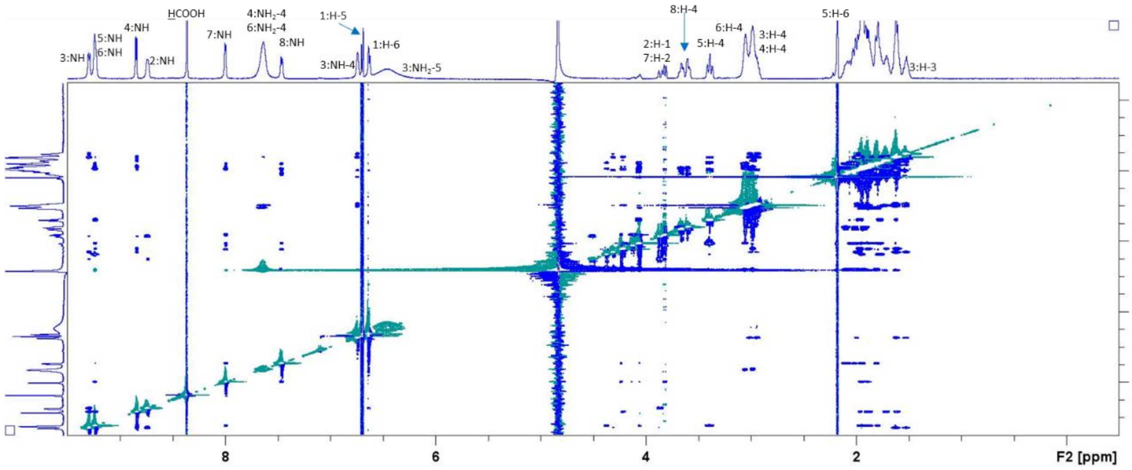

D) Comp 2:gallium, 2D-^{1}H-^{15}N-HSQC NMR spectrum at 14 T and 293 K in H_2O/D_2O (90/10 v/v).

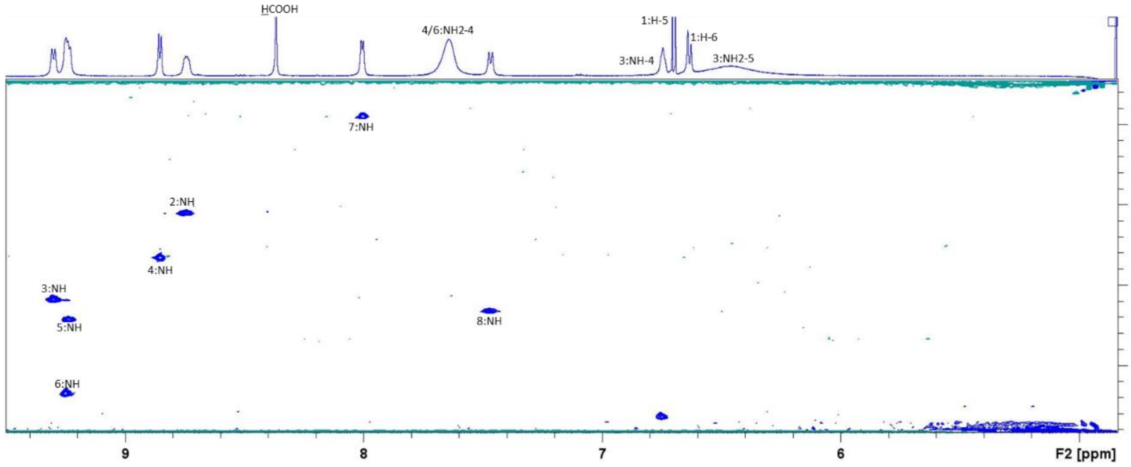

E) Comp 2:gallium, 2D-^{1}H-^{13}C-HSQC-DEPT NMR spectrum at 14 T and 293 K in D_2O.

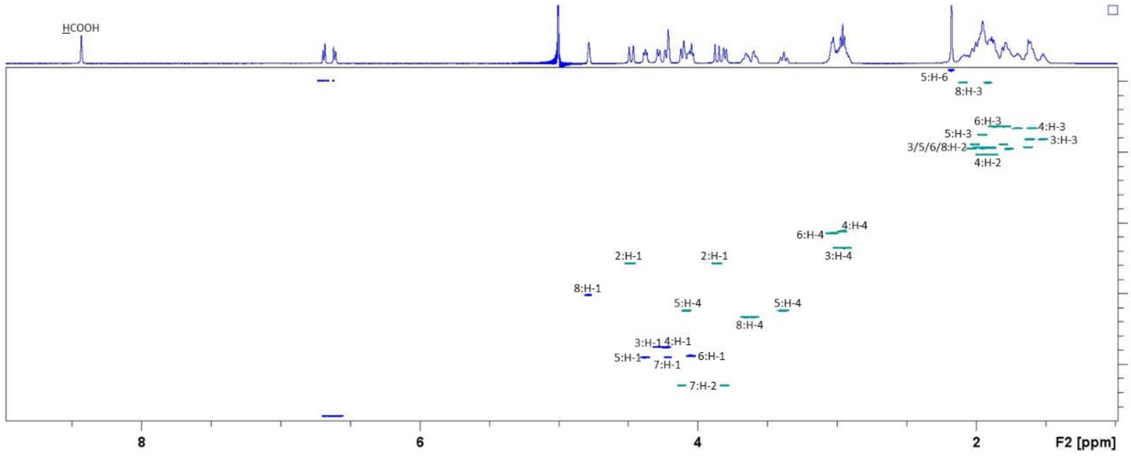

F) Comp 2:gallium, 2D-^{1}H-^{13}C-HSQC-TOCSY (*T*m = 80 ms) NMR spectrum at 14 T and 293 K in D$_2$O.

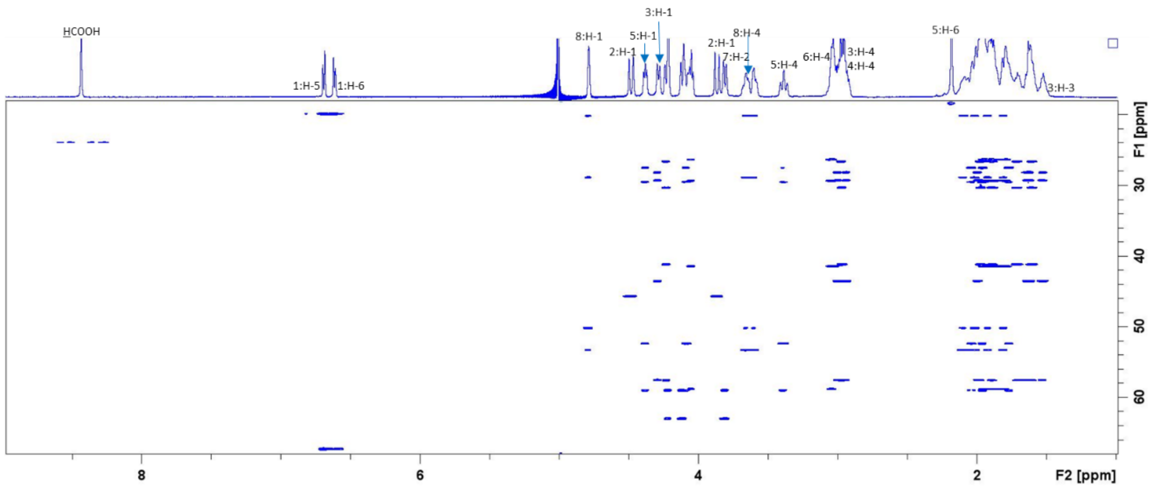

G) Comp 2:gallium, 2D-^{1}H-^{13}C-HMBC (*J*app = 10 Hz) NMR spectrum at 14 T and 293 K in D$_2$O.

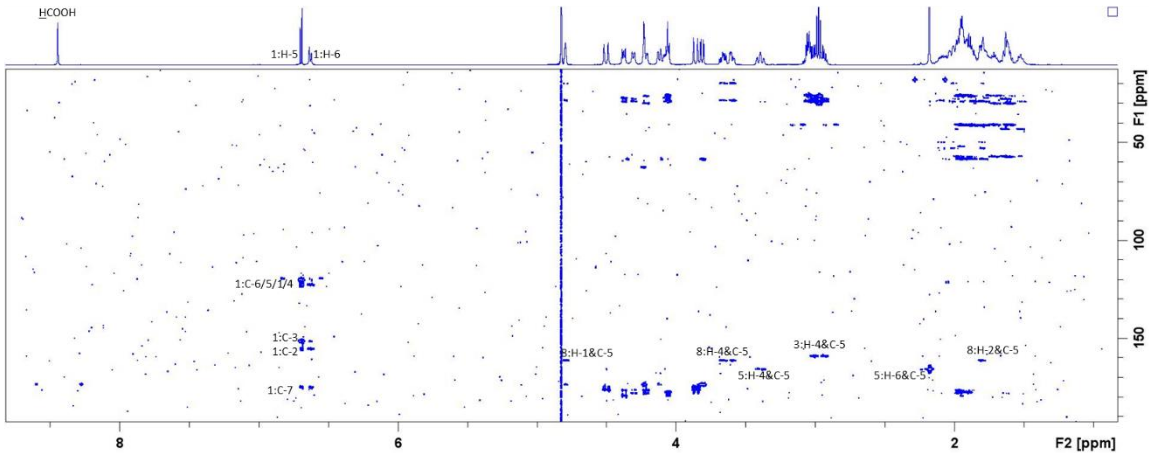

H) Comp 2:gallium, 2D-^{1}H-^{15}N-HMBC (*J*app = 3 Hz) NMR spectrum at 14 T and 293 K in D$_2$O.

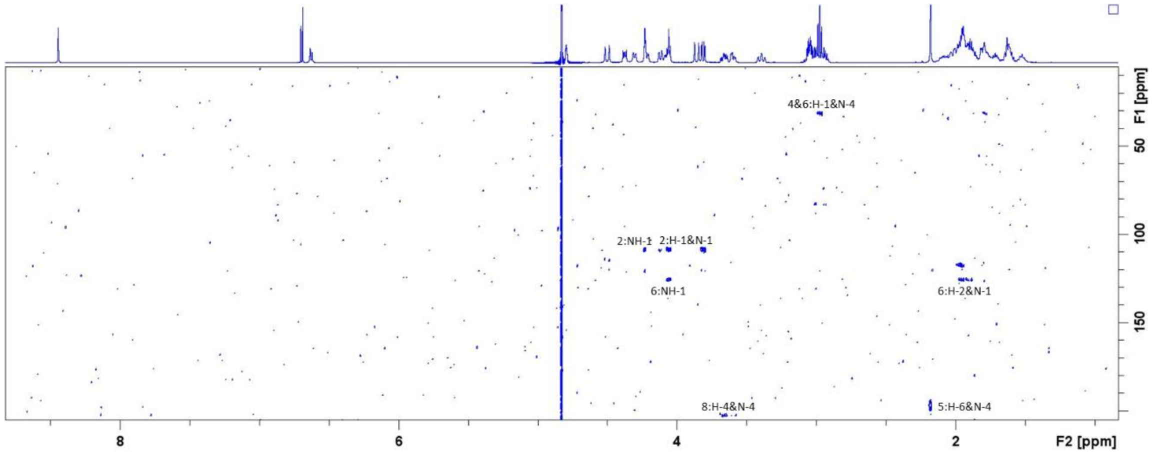

I) Comp 2:iron, HRMS (ES+) analysis

Single Mass Analysis
Tolerance = 10.0 mDa / DBE: min = -1.5, max = 50.0
Element prediction: Off
Number of isotope peaks used for i-FIT = 2

Monoisotopic Mass, Odd and Even Electron Ions
1316 formula(e) evaluated with 29 results within limits (up to 50 closest results for each mass)
Elements Used:
C: 0-50 H: 0-70 N: 0-15 O: 0-15 Cl: 0-1 Fe: 0-1

ClDaf_bis 60 (1.646) Cm (60:63)
1: TOF MS ES+
7.57e+003

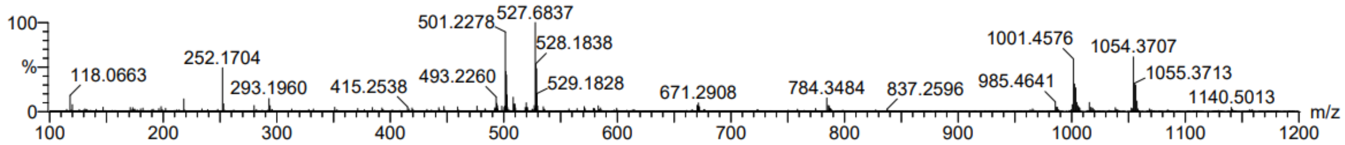

Minimum:				-1.5		
Maximum:		10.0	10.0	50.0		
Mass	Calc. Mass	mDa	PPM	DBE	i-FIT	Formula
1054.3707	1054.3686	2.1	2.0	16.0	0.8	C40 H63 N14 O14 Cl Fe
	1054.3700	0.7	0.7	15.5	2.2	C42 H65 N11 O15 Cl Fe
	1054.3660	4.7	4.5	24.0	4.6	C44 H55 N14 O15 Cl
	1054.3681	2.6	2.5	20.5	5.9	C43 H60 N13 O15 Fe
	1054.3794	-8.7	-8.3	20.5	6.4	C42 H60 N15 O14 Fe

NMR and HRMS Compound 3, Dactyloferrin Bruker-600

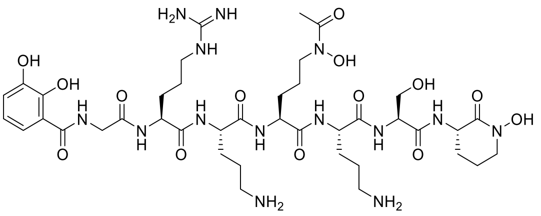

A) Comp 3: 1D-¹H-wet with 1 ms pulses NMR spectrum at 14 T and 293 K in H_2O/D_2O (90/10 v/v).

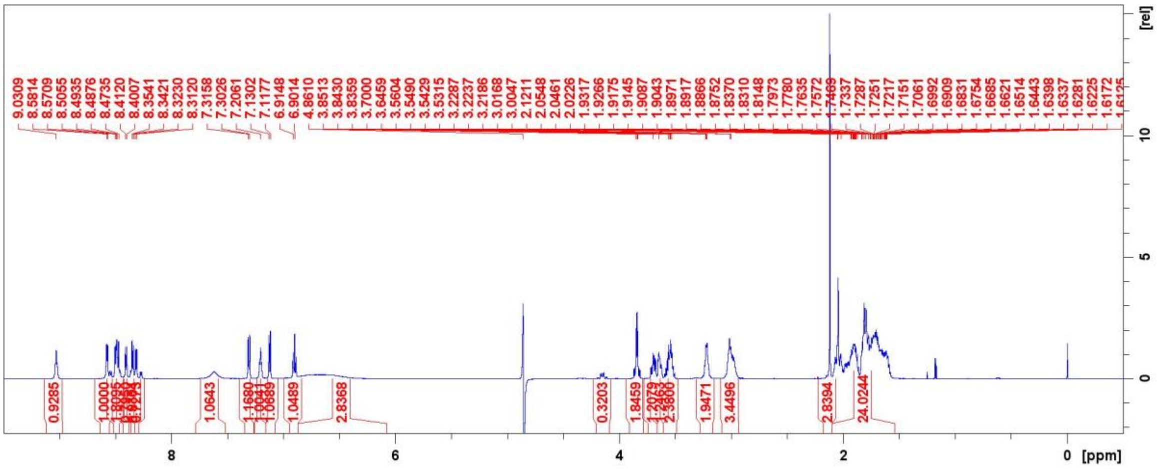

B) Comp 3: 2D-¹H-COSY NMR spectrum at 14 T and 293 K in H_2O/D_2O (90/10 v/v).

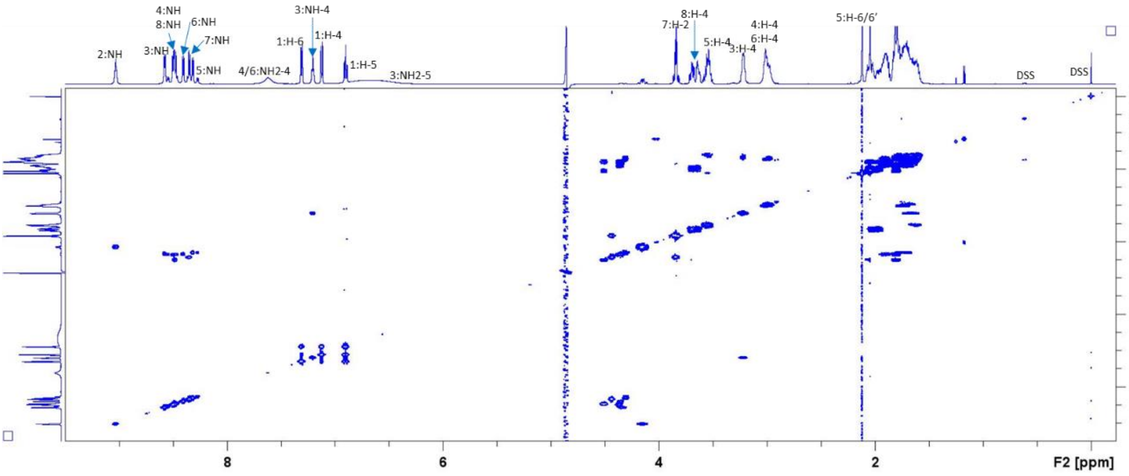

C) Comp 3: 2D-¹H-TOCSY (*T*m = 100 ms) NMR spectrum at 14 T and 293 K in H_2O/D_2O (90/10 v/v).

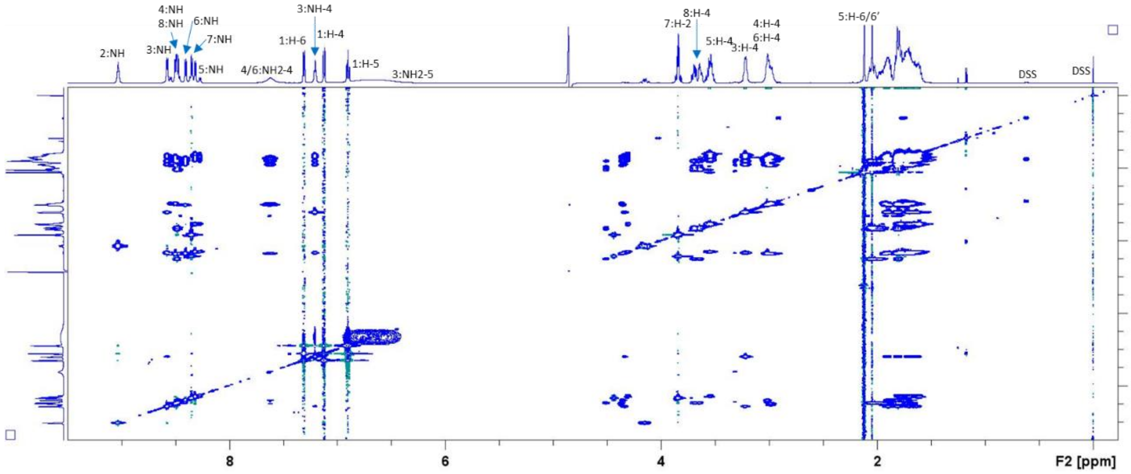

D) Comp 3: 2D-^{1}H-ROESY (*T*m = 300 ms) NMR spectrum at 14 T and 293 K in H_2O/D_2O (90/10 v/v).

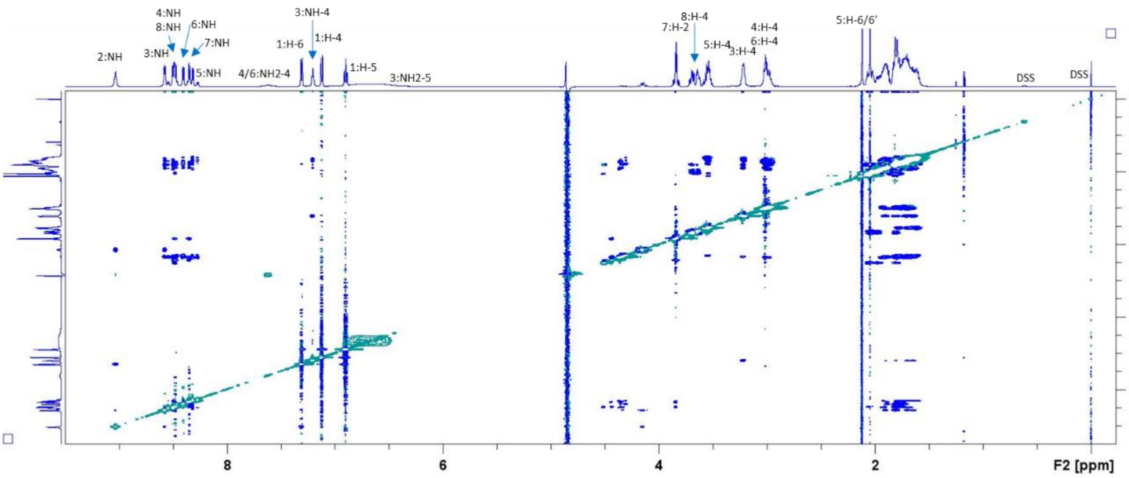

E) Comp 3:1D-^{13}C{^{1}H} NMR spectrum in H_2O/D_2O (90/10 v/v) at 14 T and 293 K.

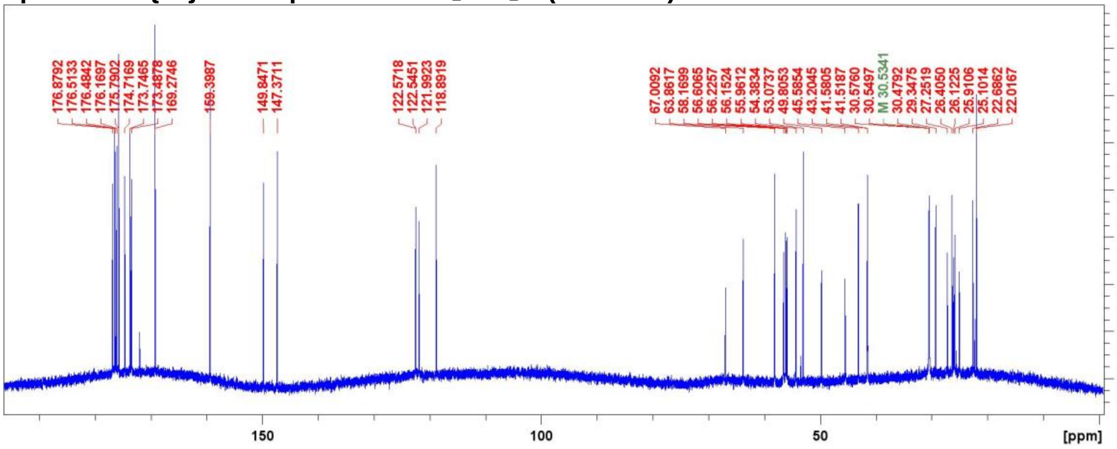

F) Comp 3:1D-^{13}C-DEPT135 NMR spectrum in H_2O/D_2O (90/10 v/v) at 14 T and 293 K.

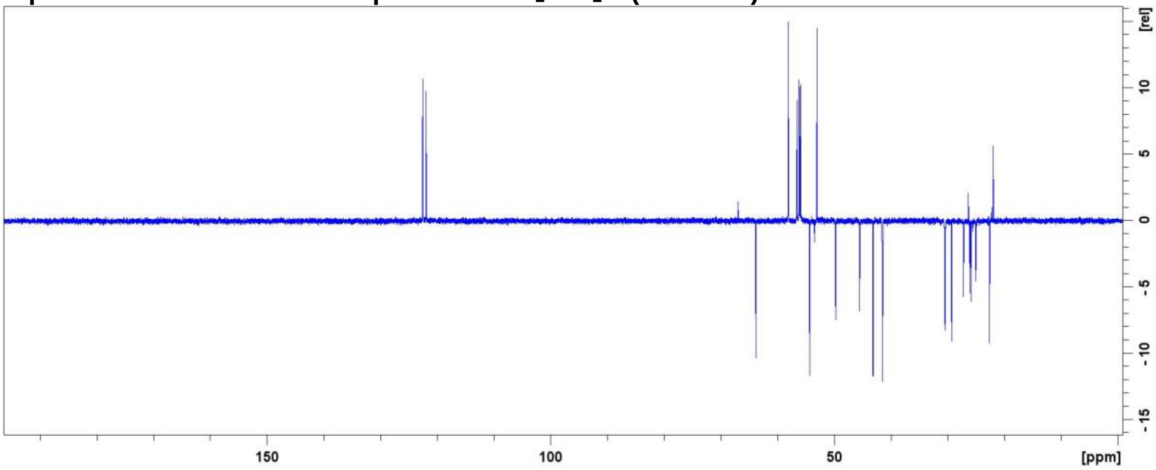

G) Comp 3: 2D-^{1}H-^{13}C-HSQC-TOCSY (*T*m = 77,5 ms) NMR spectrum at 14 T and 293 K in H_2O/D_2O (90/10 v/v).

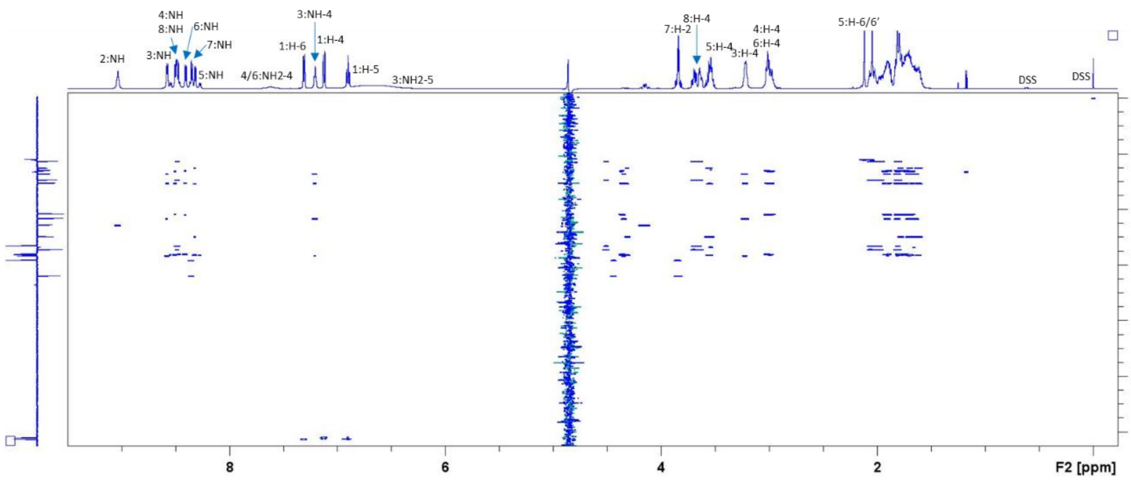

H) Comp 3: 2D-^{1}H-^{15}N-HSQC NMR spectrum at 14 T and 293 K in H_2O/D_2O (90/10 v/v).

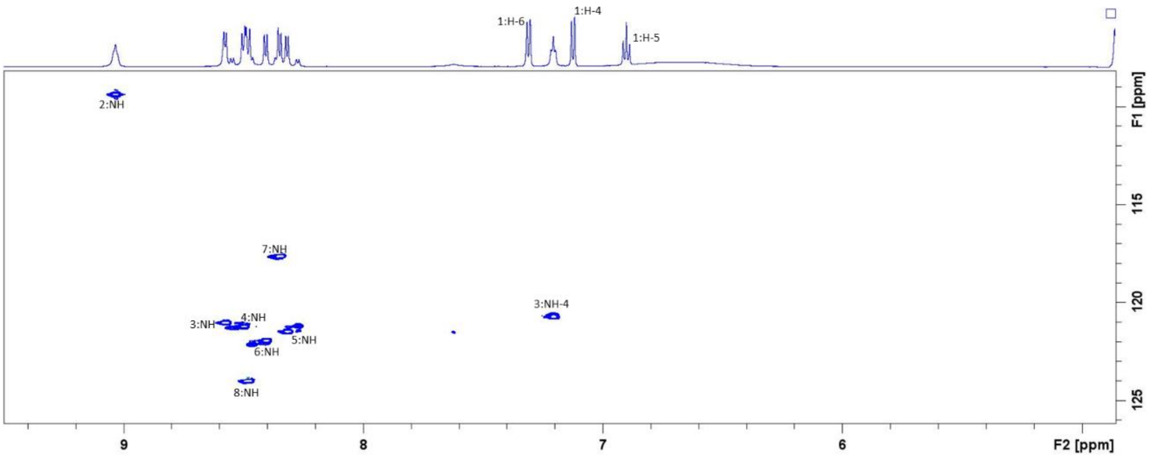

I) Comp 3:1D-^{1}H NMR spectrum of Daf [3] in D_2O at 14 T and 293 K.

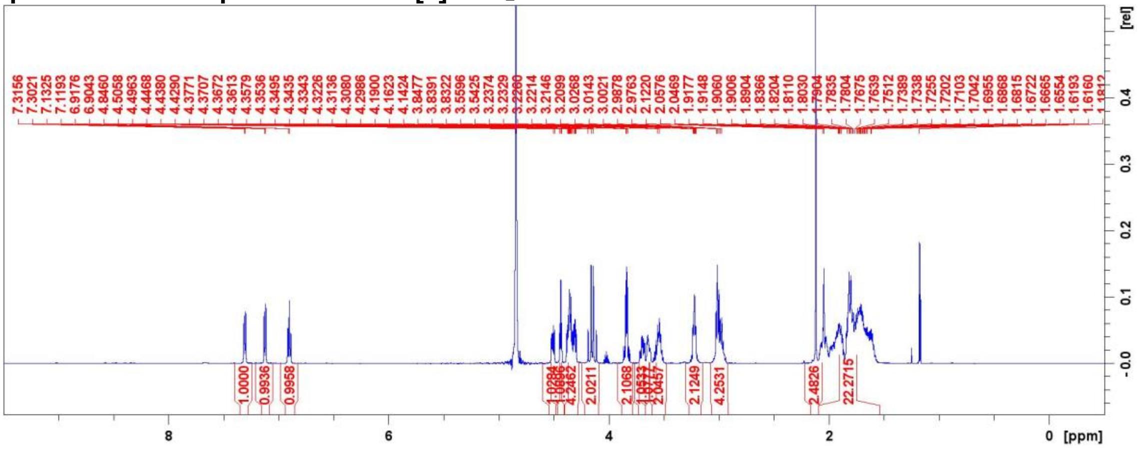

J) Comp 3: 2D-^{1}H-^{13}C-HSQC-DEPT NMR spectrum at 14 T and 293 K in D_2O.

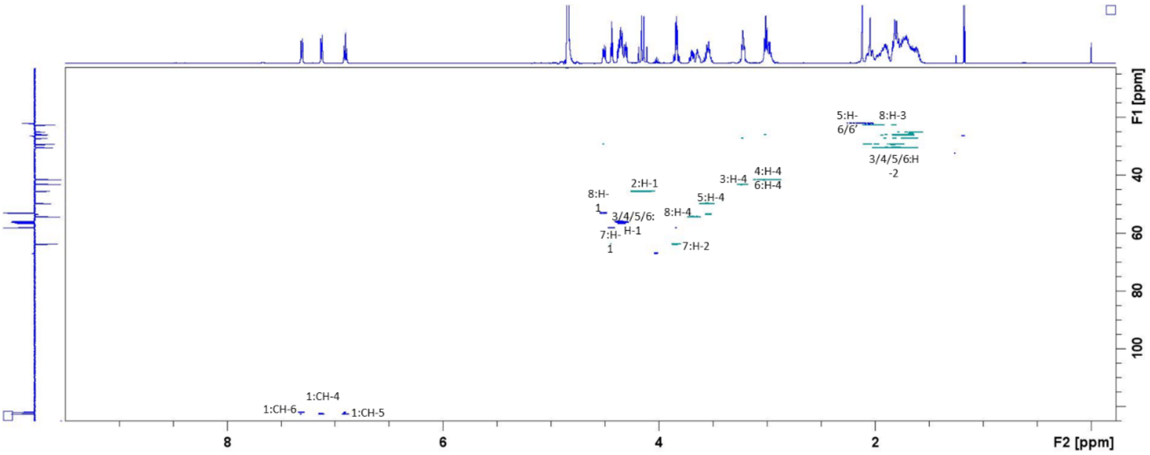

K) Comp 3: 2D-^{1}H-^{13}C-HMBC (*J*app = 10 Hz) NMR spectrum at 14 T and 293 K in D_2O.

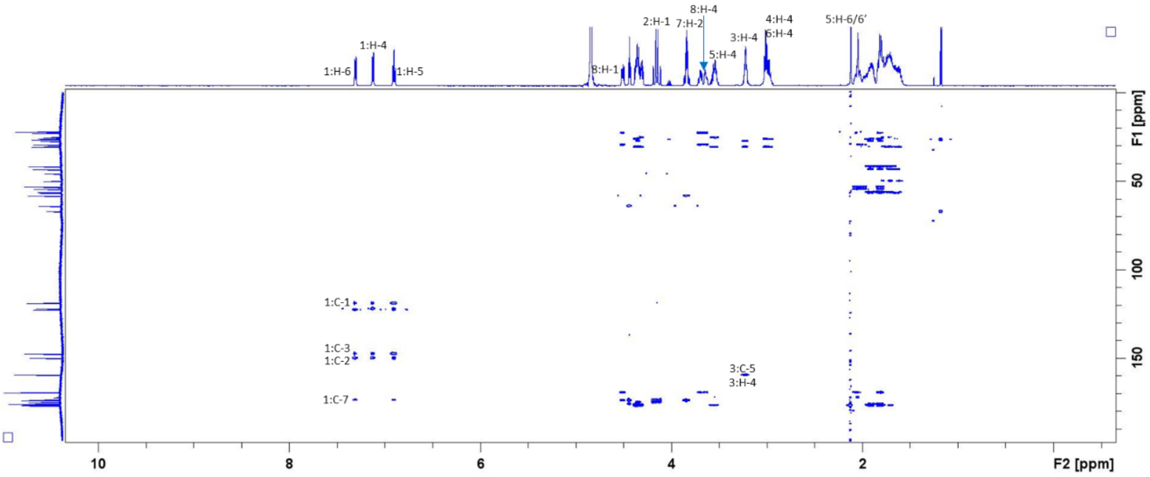

L) Comp 3: 2D-^{1}H-^{15}N-HMBC (*J*app = 8 Hz) NMR spectrum at 14 T and 293 K in D_2O.

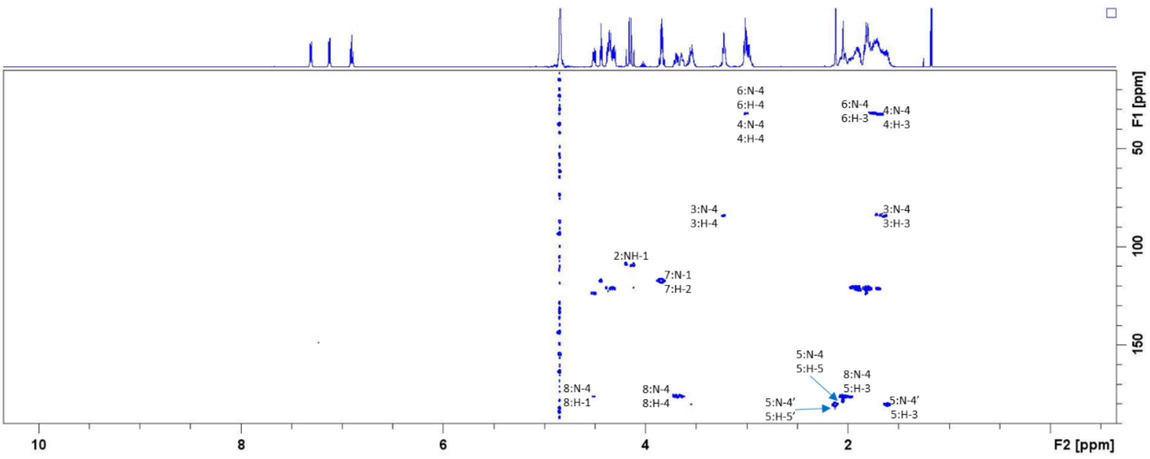

M) Comp 3 : HRMS (ES+) analysis

Single Mass Analysis
Tolerance = 10.0 mDa / DBE: min = -1.5, max = 50.0
Element prediction: Off
Number of isotope peaks used for i-FIT = 2

Monoisotopic Mass, Even Electron Ions
2868 formula(e) evaluated with 21 results within limits (up to 50 closest results for each mass)
Elements Used:
C: 0-50 H: 0-70 N: 0-20 O: 1-20 Fe: 0-1
Daf_bis 78 (2.107)

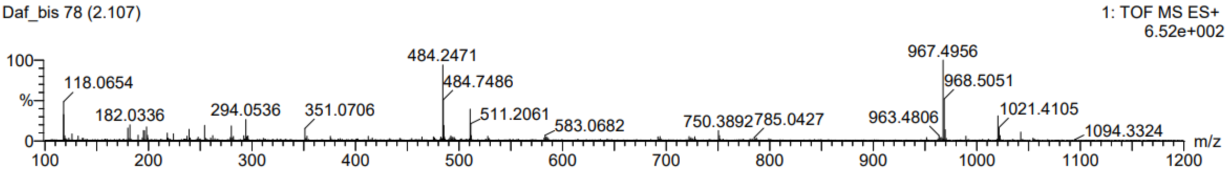

Minimum:				-1.5		
Maximum:		10.0	10.0	50.0		
Mass	Calc. Mass	mDa	PPM	DBE	i-FIT	Formula
967.4956	967.4961	-0.5	-0.5	14.5	0.1	C40 H67 N14 O14
	967.4942	1.4	1.4	20.5	1.9	C44 H67 N18 O4 Fe
	967.4975	-1.9	-2.0	19.5	0.2	C41 H63 N18 O10
	967.4934	2.2	2.3	15.5	0.5	C36 H63 N20 O12
	967.4982	-2.6	-2.7	24.5	4.8	C49 H67 N16 O2 Fe

N) Comp 3:iron. HRMS (ES+) analysis

Single Mass Analysis
Tolerance = 10.0 mDa / DBE: min = -1.5, max = 50.0
Element prediction: Off
Number of isotope peaks used for i-FIT = 2

Monoisotopic Mass, Odd and Even Electron Ions
2240 formula(e) evaluated with 76 results within limits (up to 50 closest results for each mass)
Elements Used:
C: 0-50 H: 0-70 N: 0-20 O: 1-20 Fe: 0-1
Daf_bis 78 (2.107)

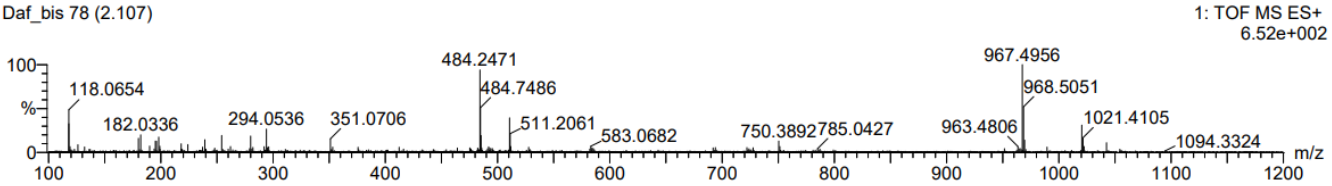

Minimum:				-1.5		
Maximum:		10.0	10.0	50.0		
Mass	Calc. Mass	mDa	PPM	DBE	i-FIT	Formula
1020.4075	1020.4076	-0.1	-0.1	16.0	0.0	C40 H64 N14 O14 Fe
	1020.4036	3.9	3.8	19.0	0.0	C43 H60 N10 O19
	1020.4089	-1.4	-1.4	15.5	0.0	C42 H66 N11 O15 Fe
	1020.4050	2.5	2.5	18.5	0.0	C45 H62 N7 O20
	1020.4076	-0.1	-0.1	10.5	0.0	C41 H70 N7 O19 Fe

NMR and HRMS Compound 4 :gallium, Chlorodactyloferrin-Pyridomycin conjugate Bruker-600

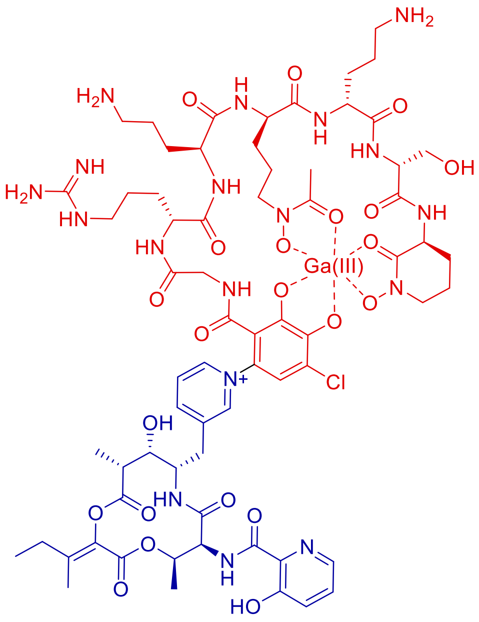

A) Comp. 4:gallium: 1D-^{1}H-w5. NMR spectrum in H_2O/CD_3CN (1/1 v/v) at 14 T and 293 K

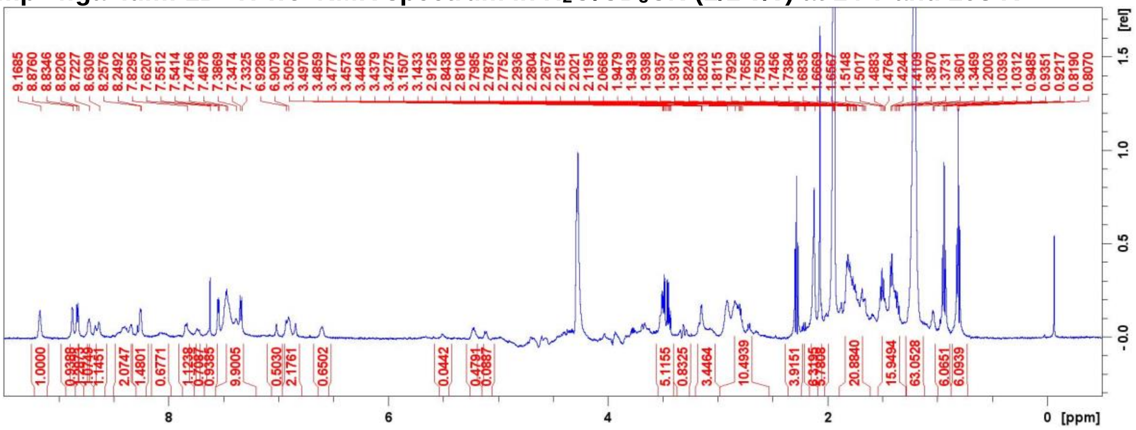

B) Comp. 4:gallium:2D-^{1}H-COSY. NMR spectrum in H_2O/CD_3CN (1/1 v/v) at 14 T and 293 K

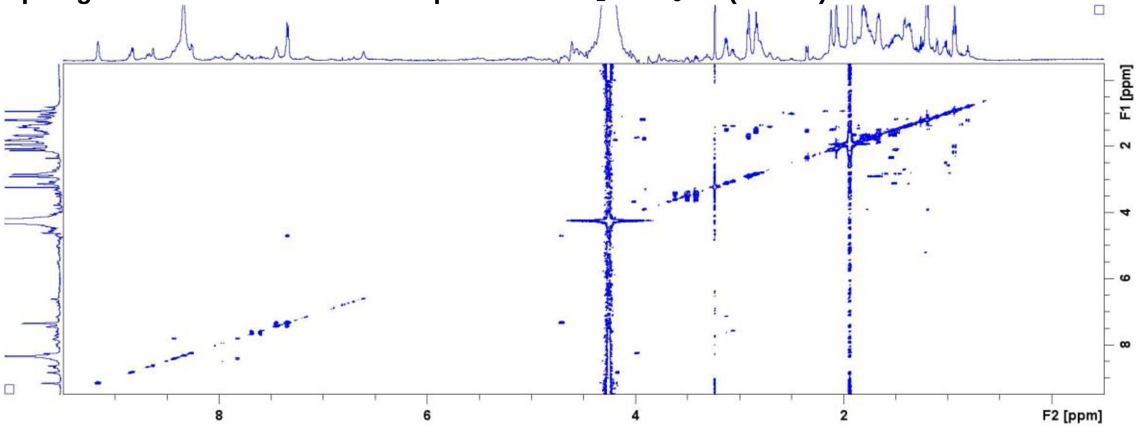

C) Comp. 4:gallium:2D-^{1}H-TOCSY (*T*m = 58 ms). NMR spectrum in H_2O/CD_3CN (1/1 v/v) at 14 T and 293 K

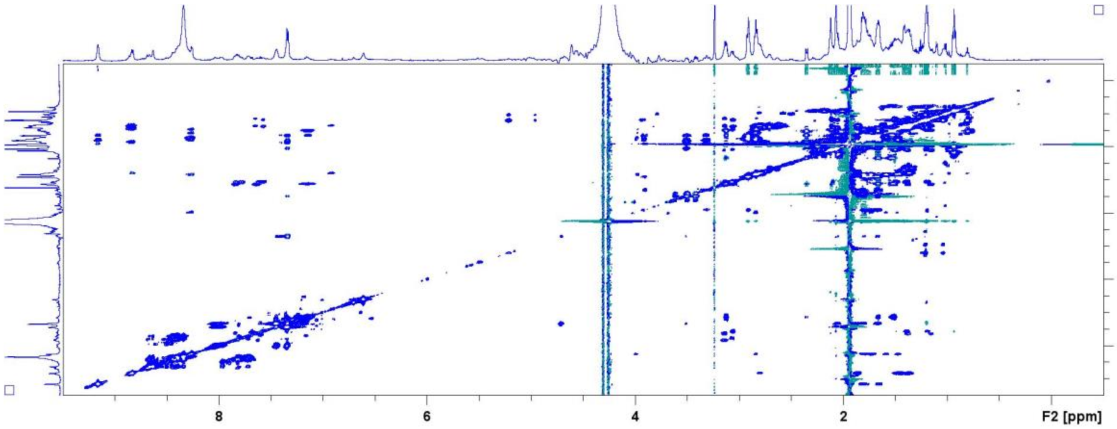

D) Comp. 4:gallium:2D-^{1}H-ROESY (*T*m = 400 ms). NMR spectrum in H_2O/CD_3CN (1/1 v/v) at 14 T and 293 K

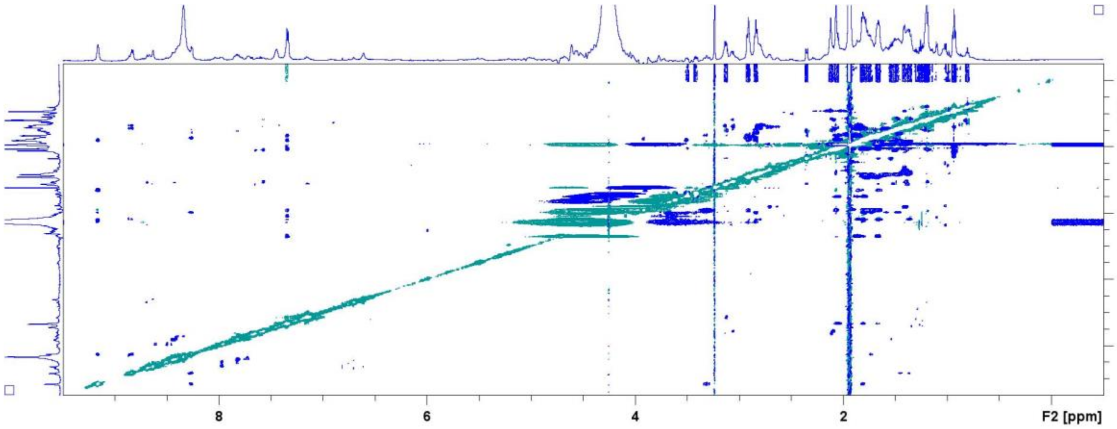

E) Comp. 4:gallium:2D-^{1}H-^{13}Caliphatic-HSQC. NMR spectrum in H_2O/CD_3CN (1/1 v/v) at 14 T and 293 K

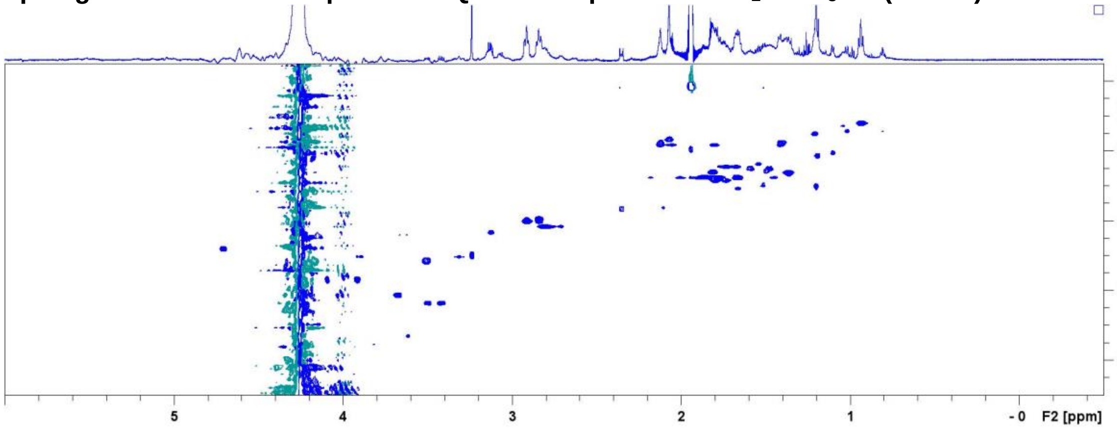

F) Comp. 4:gallium:2D-^{1}H-^{13}Caliphatic-HSQC-TOCSY (*T*m = 58 ms). NMR spectrum in H_2O/CD_3CN (1/1 v/v) at 14 T and 293 K

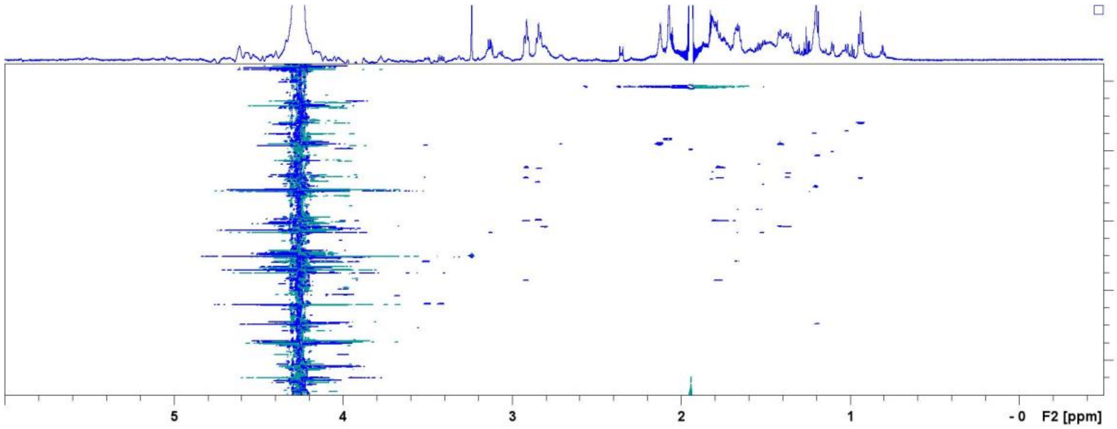

G) Comp. 4:gallium:2D-^{1}H-^{13}Caromatic-HSQC. NMR spectrum in H_2O/CD_3CN (1/1 v/v) at 14 T and 293 K

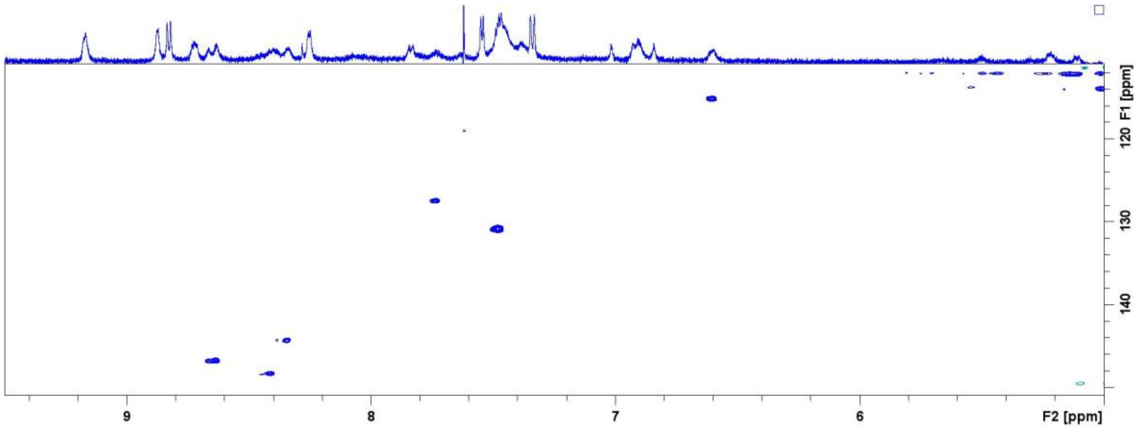

H) Comp. 4:gallium:2D-^{1}H-^{13}C-HMBC (*J*app = 10 Hz). NMR spectrum in H_2O/CD_3CN (1/1 v/v) at 14 T and 293 K

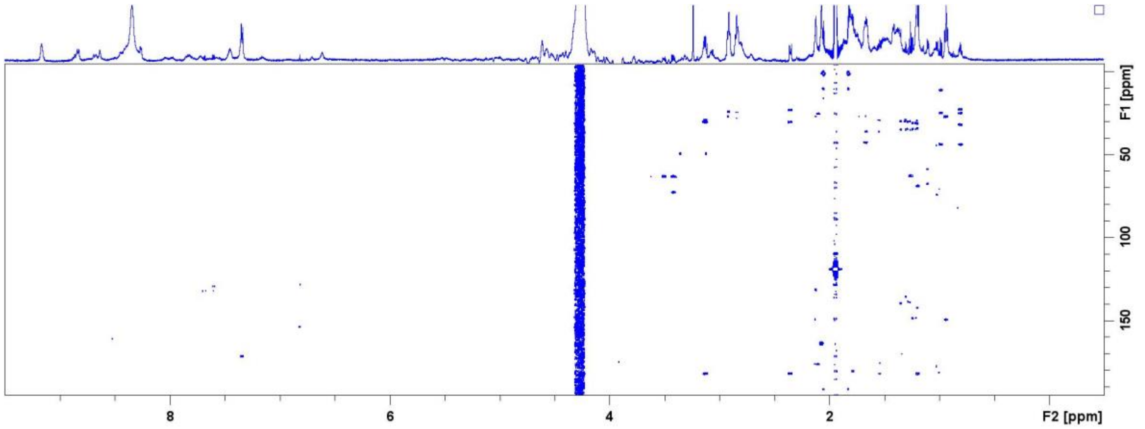

I) Comp. 4:gallium:2D-^{1}H-^{15}N-HSQC. NMR spectrum in H_2O/CD_3CN (1/1 v/v) at 14 T and 293 K

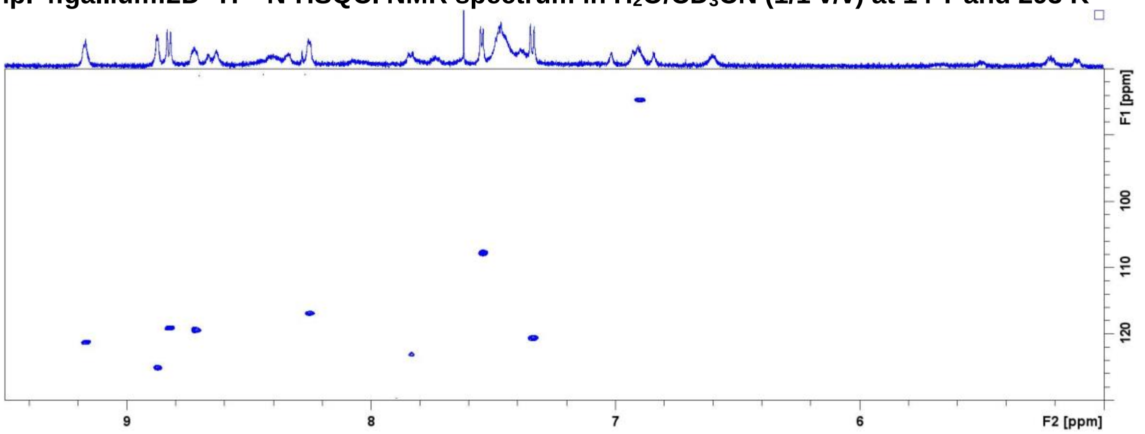

J) Comp 4:iron . HRMS (ES+) analysis

Single Mass Analysis
Tolerance = 5.0 mDa / DBE: min = -1.5, max = 50.0
Element prediction: Off
Number of isotope peaks used for i-FIT = 3

Monoisotopic Mass, Odd and Even Electron Ions
1919 formula(e) evaluated with 2 results within limits (up to 50 best isotopic matches for each mass)
Elements Used:
C: 67-69 H: 0-100 N: 0-18 O: 0-25 Cl: 0-1 Fe: 0-1

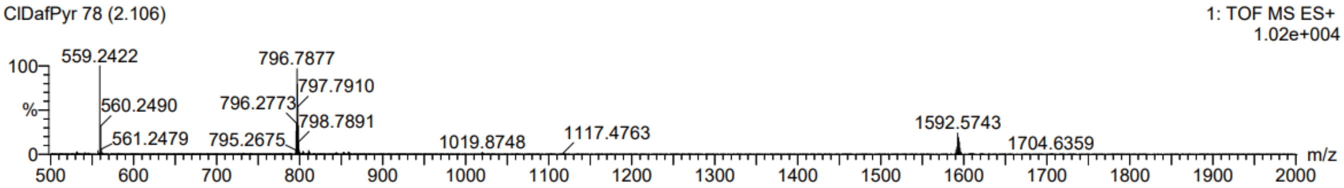

Minimum:				-1.5		
Maximum:		5.0	10.0	50.0		

Mass	Calc. Mass	mDa	PPM	DBE	i-FIT	Formula
1592.5743	1592.5750	-0.7	-0.4	30.0	22.4	C67 H93 N18 O22 Cl Fe
	1592.5763	-2.0	-1.3	29.5	25.6	C69 H95 N15 O23 Cl Fe

NMR and HRMS Compound 5, Bruker-300

Comp 5. ^{1}H NMR of 5 in $CDCl_3$, Bruker-300

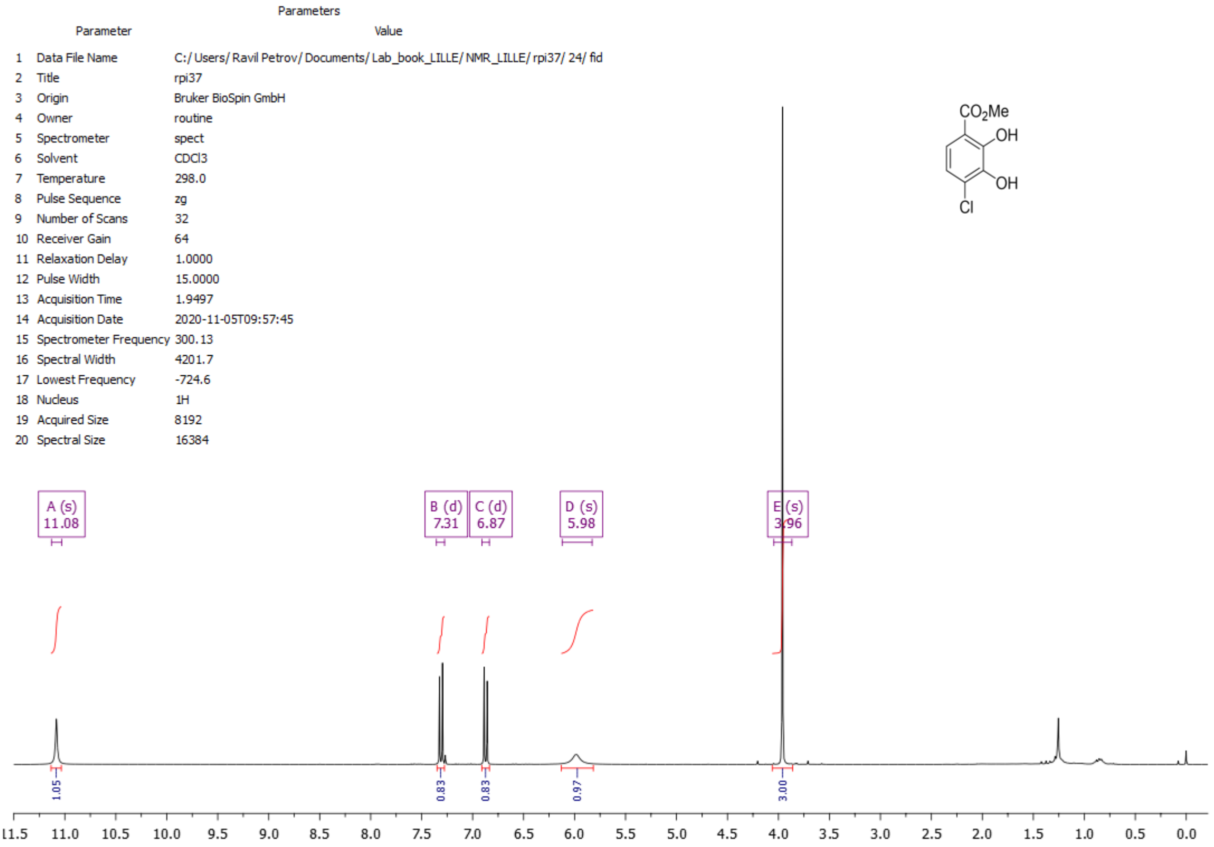

Comp 5.^{13}C NMR of 5 in $CDCl_3$, Bruker-300

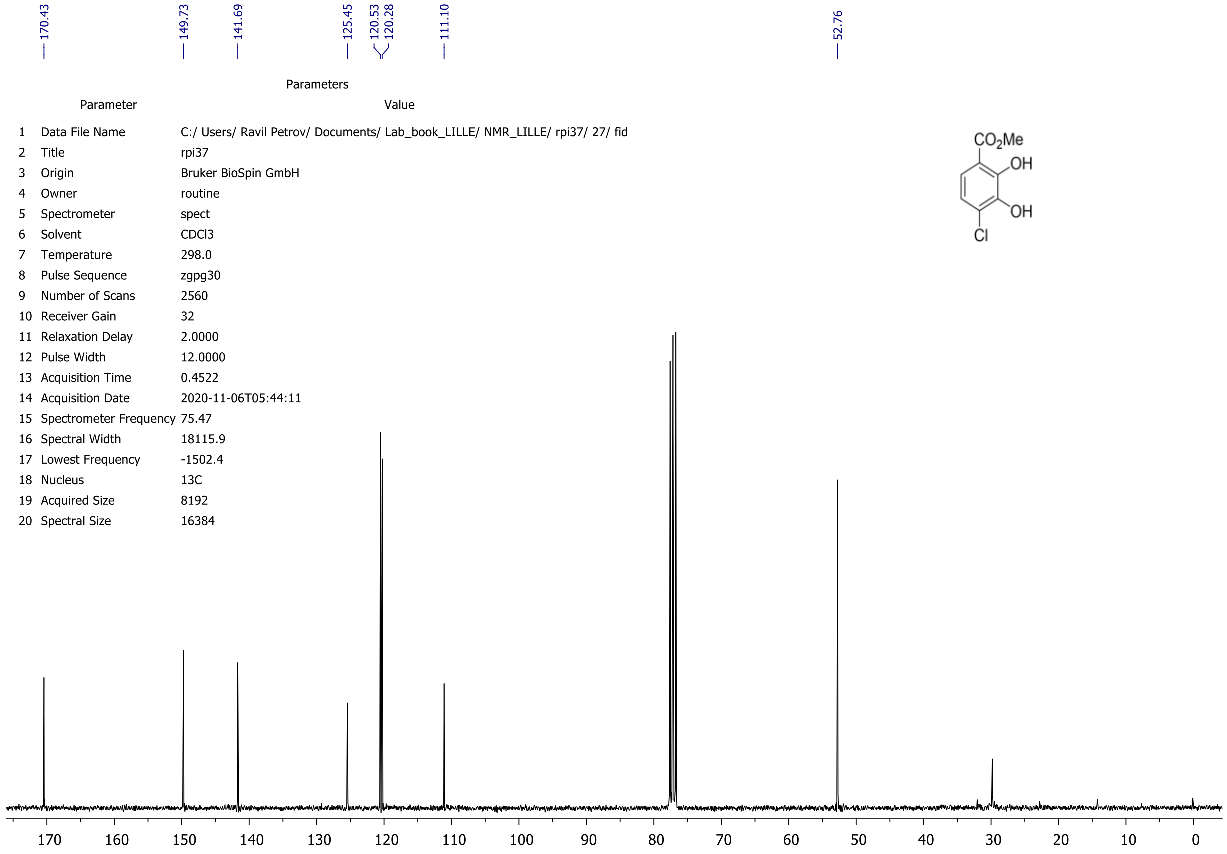

Comp 5. HRMS (ES+) analysis

Single Mass Analysis
Tolerance = 10.0 mDa / DBE: min = -1.5, max = 50.0
Element prediction: Off
Number of isotope peaks used for i-FIT = 2

Monoisotopic Mass, Even Electron Ions
274 formula(e) evaluated with 14 results within limits (up to 50 closest results for each mass)
Elements Used:
C: 0-20 H: 0-20 N: 0-10 O: 0-10 Cl: 0-1

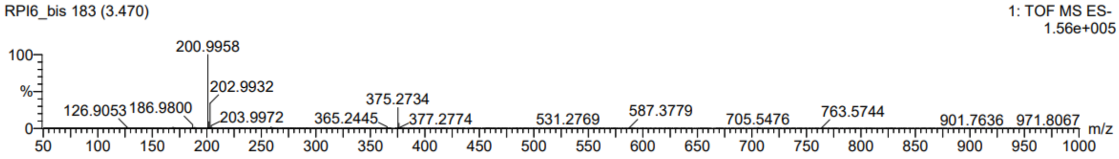

Mass	Calc. Mass	mDa	PPM	DBE	i-FIT	Formula
Minimum:				-1.5		
Maximum:		10.0	10.0	50.0		
200.9958	200.9955	0.3	1.5	5.5	2.0	C8 H6 O4 Cl
	200.9968	-1.0	-5.0	10.5	470.1	C9 H2 N4 Cl
	200.9977	-1.9	-9.5	14.5	3017.2	C14 H O2
	200.9936	2.2	10.9	10.5	325.6	C9 H N2 O4
	200.9928	3.0	14.9	6.5	546.0	C4 H2 N6 O2 Cl

NMR and HRMS Compound 6, Bruker-300

Comp 6. ^{1}H NMR of 6 in $CDCl_3$, Bruker-300

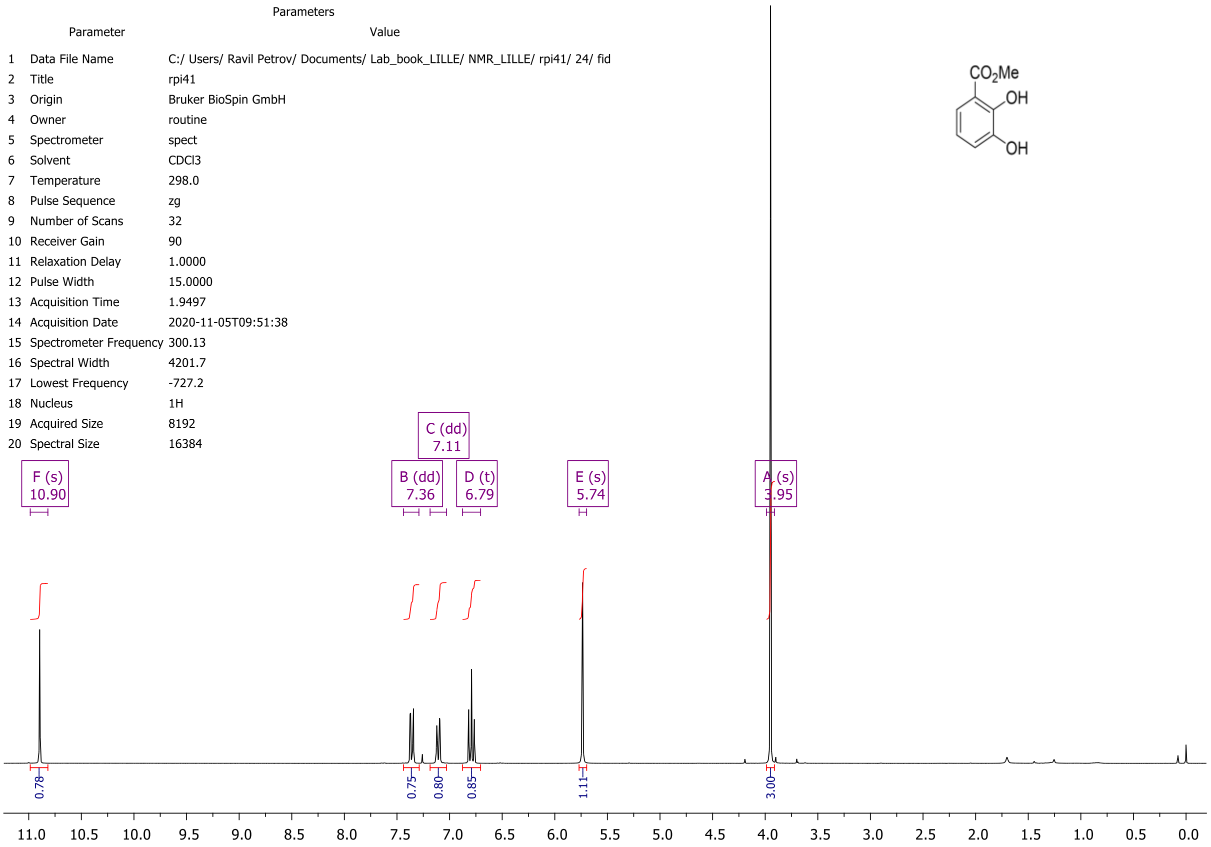

Comp 6. ^{13}C NMR of 6 in DMSO-d_6, Bruker-300

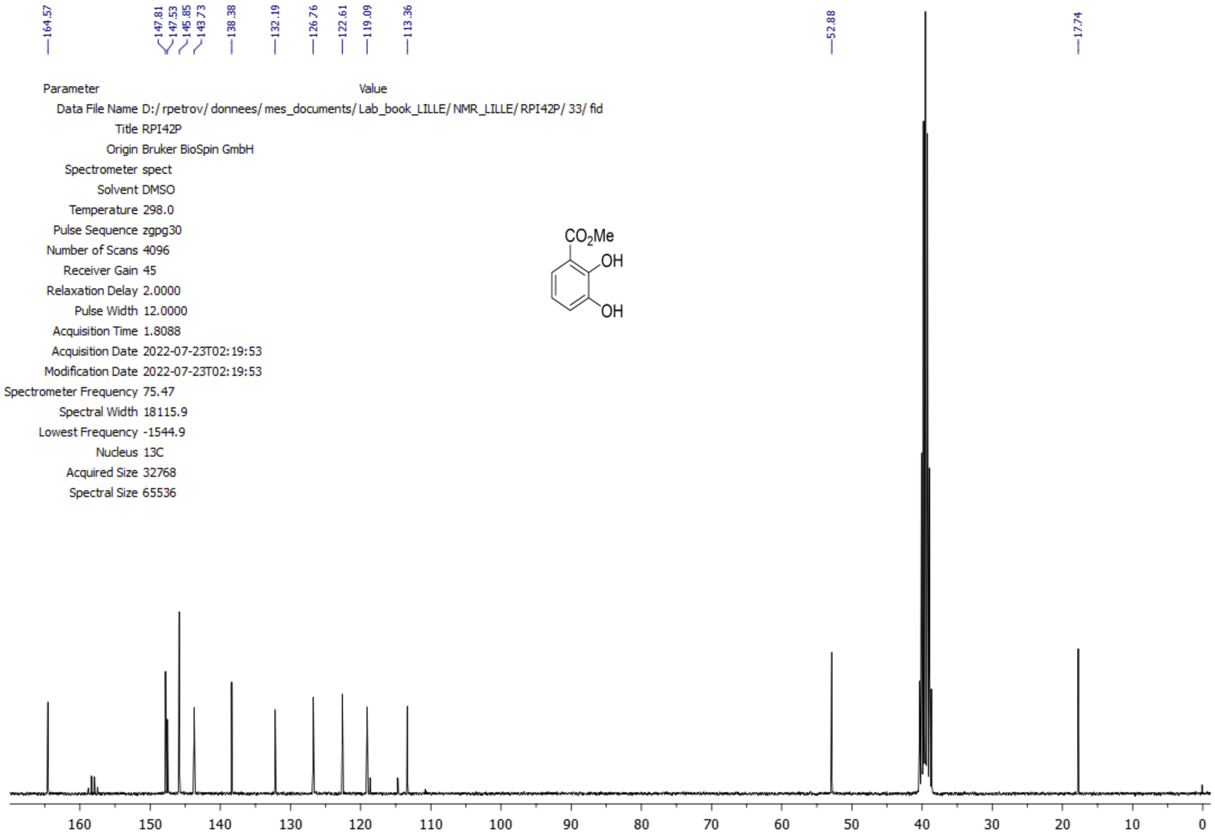

Comp 6. HRMS (ES+) analysis.

Single Mass Analysis
Tolerance = 10.0 mDa / DBE: min = -1.5, max = 50.0
Element prediction: Off
Number of isotope peaks used for i-FIT = 2

Monoisotopic Mass, Even Electron Ions
202 formula(e) evaluated with 9 results within limits (up to 50 closest results for each mass)
Elements Used:
C: 0-20 H: 0-20 N: 0-10 O: 0-10 Cl: 0-1
RPI141_bis 106 (2.061)

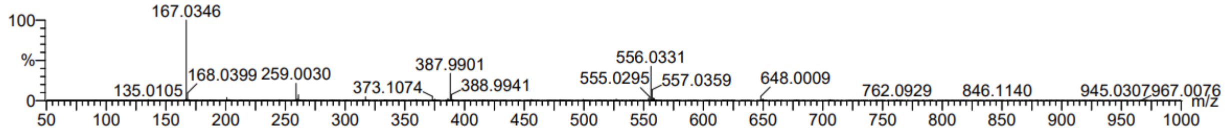

Minimum:				-1.5		
Maximum:		10.0	10.0	50.0		
Mass	Calc. Mass	mDa	PPM	DBE	i-FIT	Formula
167.0346	167.0344	0.2	1.2	5.5	13.0	C8 H7 O4
	167.0336	1.0	6.0	1.5	2762.9	C3 H8 N4 O2 Cl
	167.0358	-1.2	-7.2	10.5	832.8	C9 H3 N4
	167.0317	2.9	17.4	6.5	1210.3	C4 H3 N6 O2
	167.0376	-3.0	-18.0	5.5	312.5	C8 H8 N2 Cl

NMR and HRMS Compound 7, Bruker-300

Comp 7. ^{1}H NMR of 7 in $CDCl_3$, Bruker-300

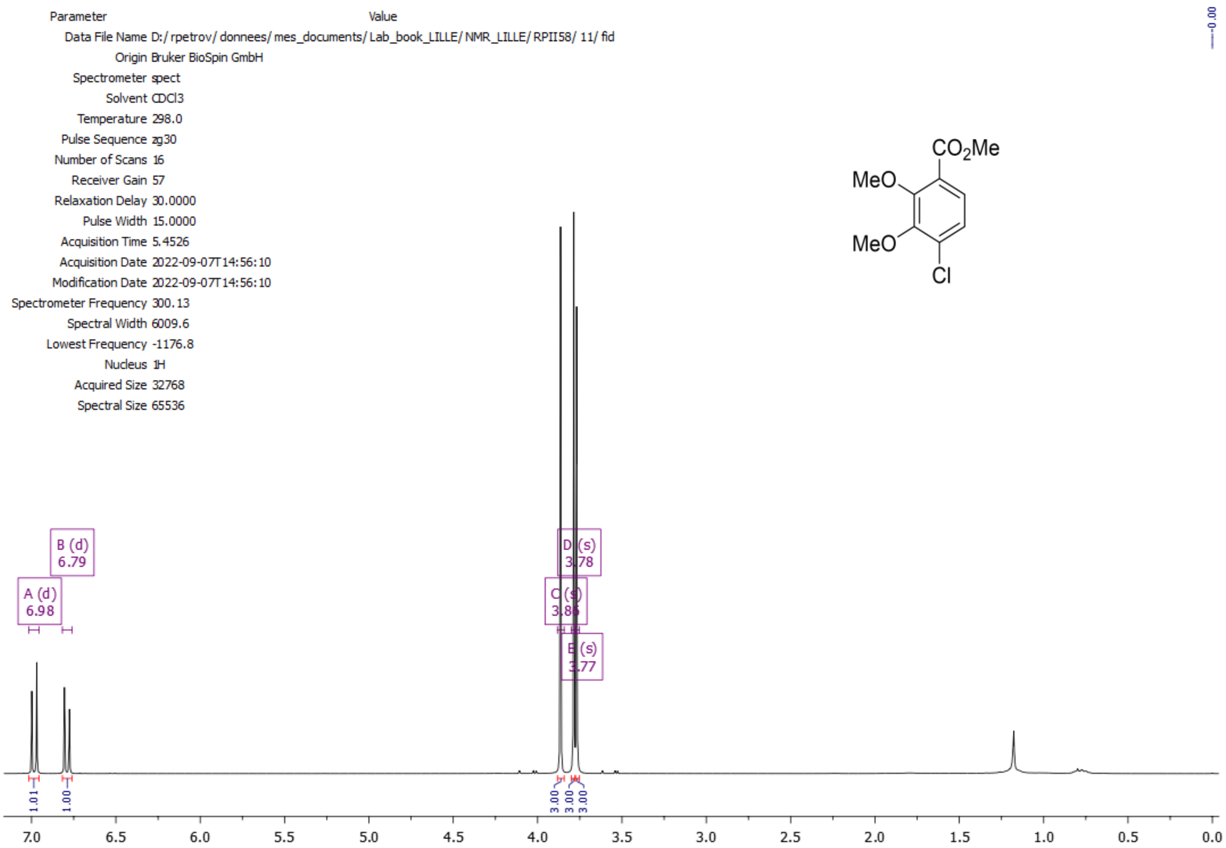

Comp 7. ^{13}C NMR of 7 in $CDCl_3$, Bruker-300

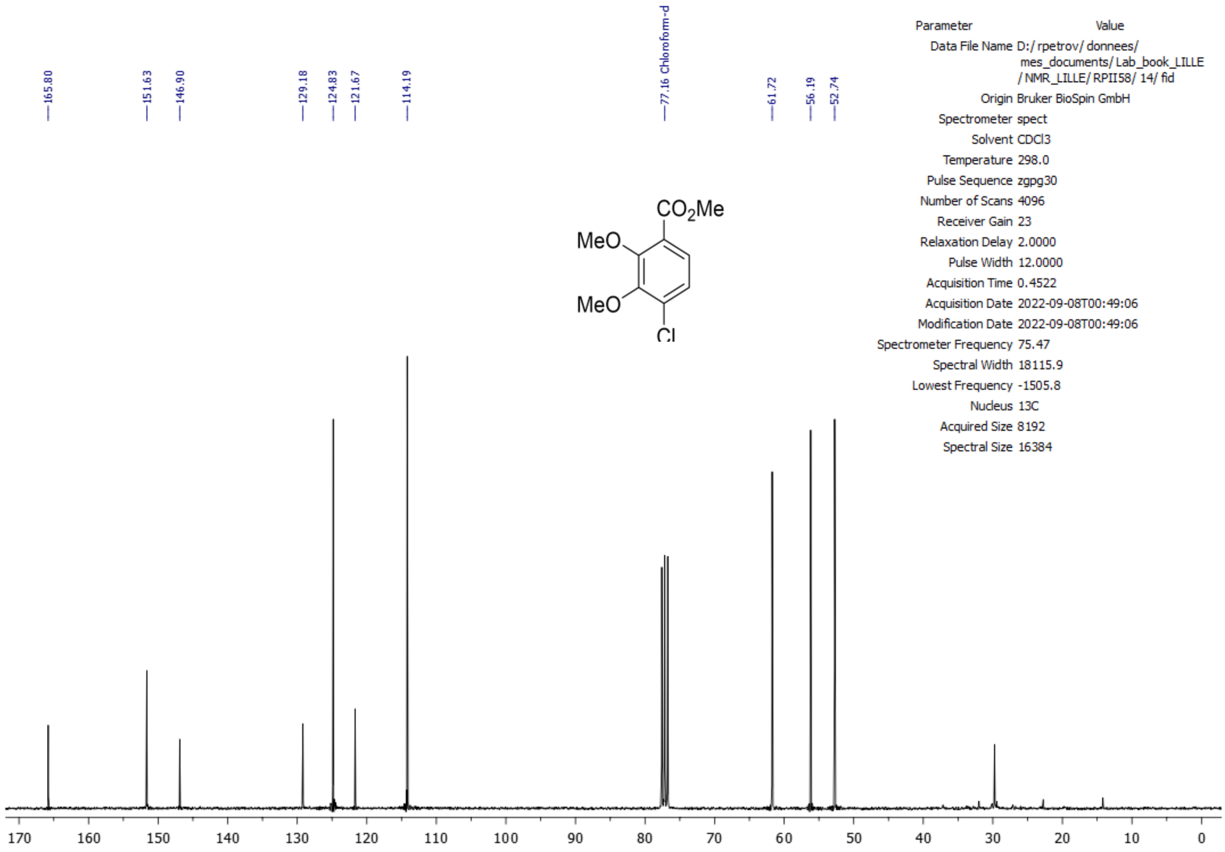

Comp 7. HRMS (ES+) analysis.

Single Mass Analysis

Tolerance = 10.0 mDa / DBE: min = -1.5, max = 50.0
Element prediction: Off
Number of isotope peaks used for i-FIT = 2

Monoisotopic Mass, Even Electron Ions
59 formula(e) evaluated with 4 results within limits (up to 50 closest results for each mass)
Elements Used:
C: 0-50 H: 0-70 O: 1-20 Cl: 0-1

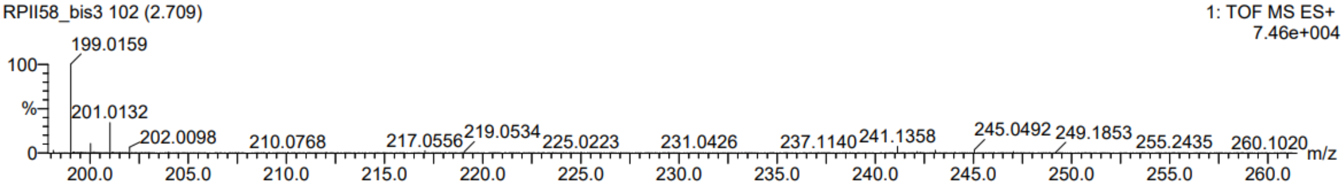

Minimum:				-1.5		
Maximum:		10.0	10.0	50.0		
Mass	Calc. Mass	mDa	PPM	DBE	i-FIT	Formula
231.0426	231.0424	0.2	0.9	4.5	5.4	C10 H12 O4 Cl
	231.0446	-2.0	-8.7	13.5	0.9	C16 H7 O2
	231.0352	7.4	32.0	0.5	15.0	C5 H11 O10
	231.0505	-7.9	-34.2	4.5	8.6	C9 H11 O7

NMR and HRMS Compound 11, Bruker-300

Comp 11. ^{1}H NMR of 11 in DMSO-d_6, Bruker-300

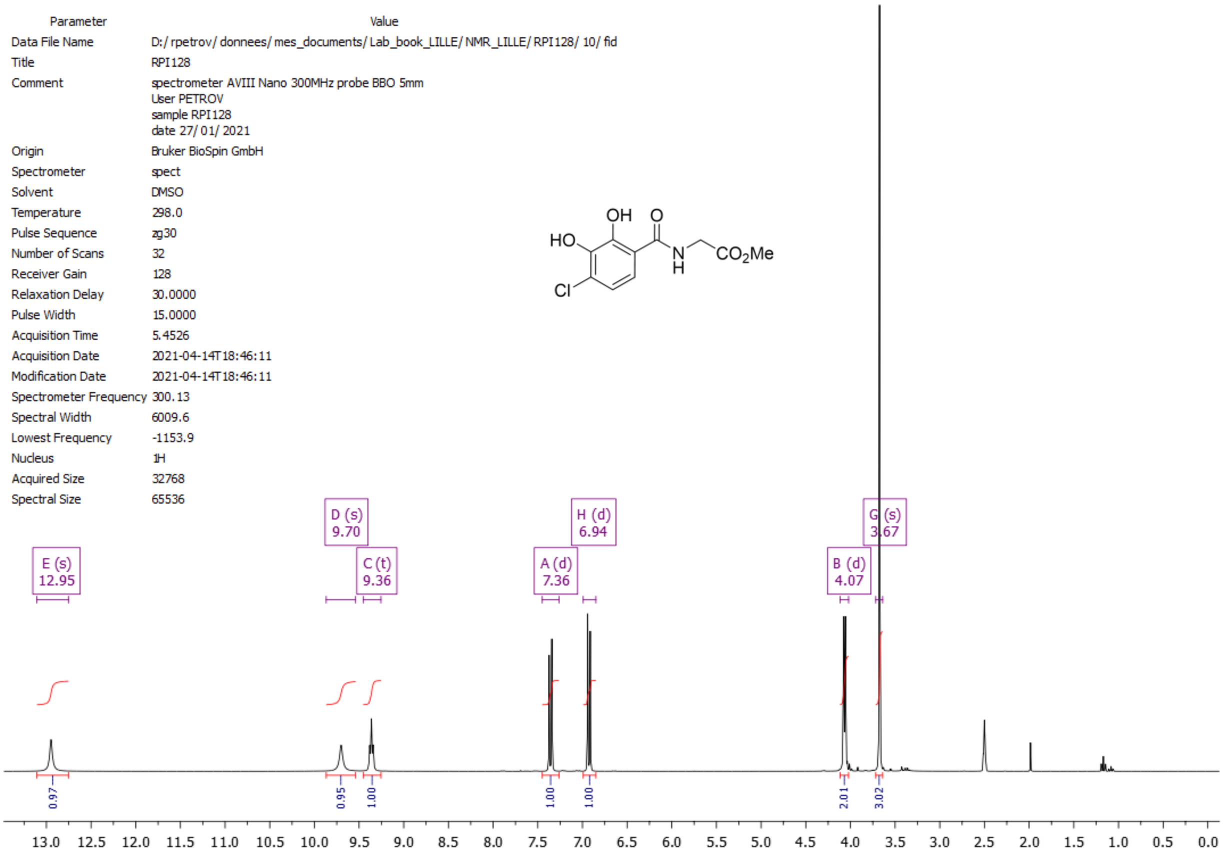

Comp 11.^{13}C NMR of in 11 in DMSO-d_6, Bruker-300

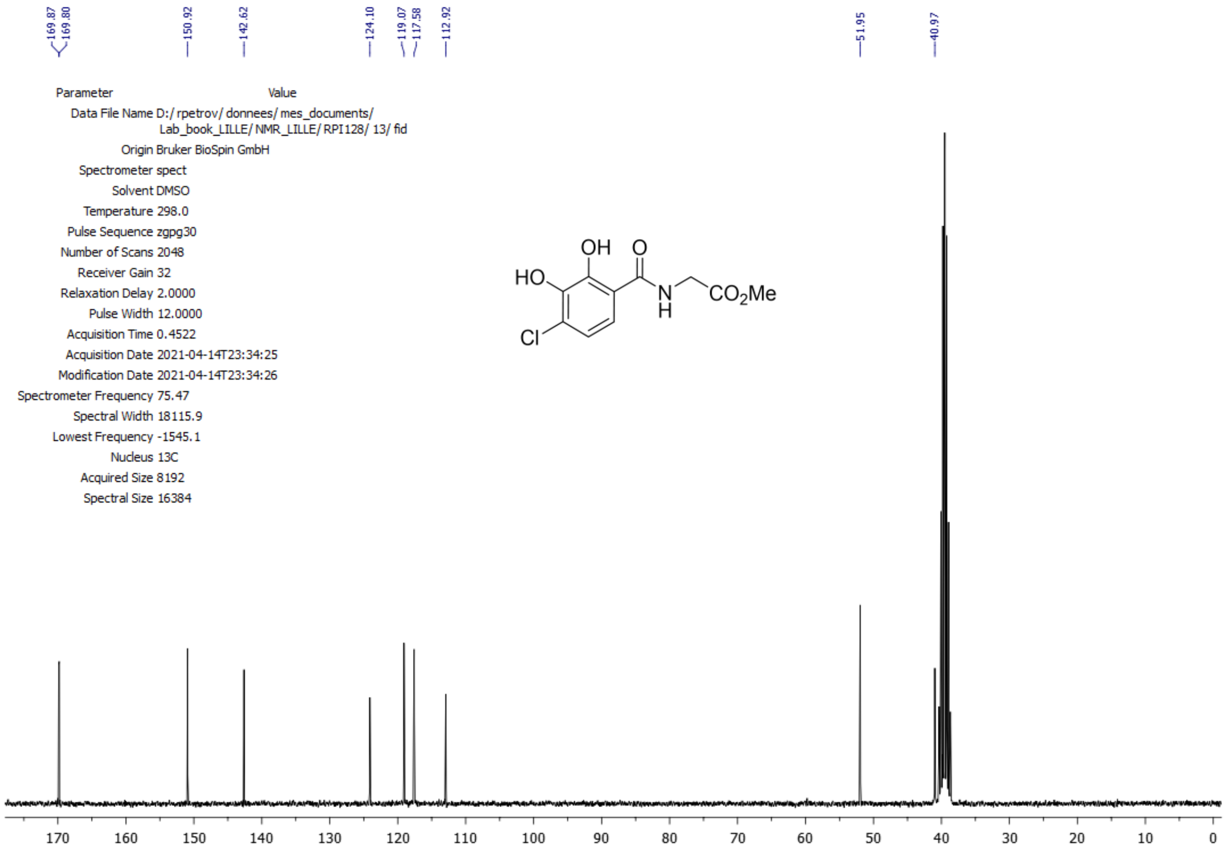

Comp 11. HRMS (ES+) analysis

Single Mass Analysis
Tolerance = 5.0 mDa / DBE: min = -1.5, max = 50.0
Element prediction: Off
Number of isotope peaks used for i-FIT = 3

Monoisotopic Mass, Odd and Even Electron Ions
159 formula(e) evaluated with 5 results within limits (up to 50 best isotopic matches for each mass)
Elements Used:
C: 0-15 H: 0-15 N: 0-2 O: 0-10 Cl: 0-1 Fe: 0-1

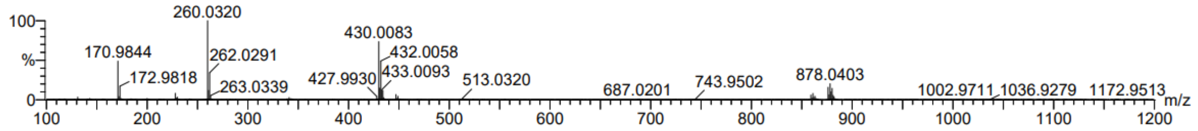

		mDa	PPM	DBE	i-FIT	Formula
Minimum:				-1.5		
Maximum:		5.0	10.0	50.0		
**Mass**	**Calc. Mass**	**mDa**	**PPM**	**DBE**	**i-FIT**	**Formula**
260.0320	260.0326	-0.6	-2.3	5.5	5.5	C10 H11 N O5 Cl
	260.0299	2.1	8.1	1.0	848.7	C7 H13 O8 Cl
	260.0353	-3.3	-12.7	10.0	924.3	C13 H9 N2 O2 Cl
	260.0281	3.9	15.0	6.0	24716.6	C8 H8 N2 O8
	260.0321	-0.1	-0.4	10.0	24988.8	C13 H8 O6

NMR and HRMS Compound 16, Bruker-300

Comp 16a . ^{1}H NMR of 16a in DMSO-d_6, Bruker-300

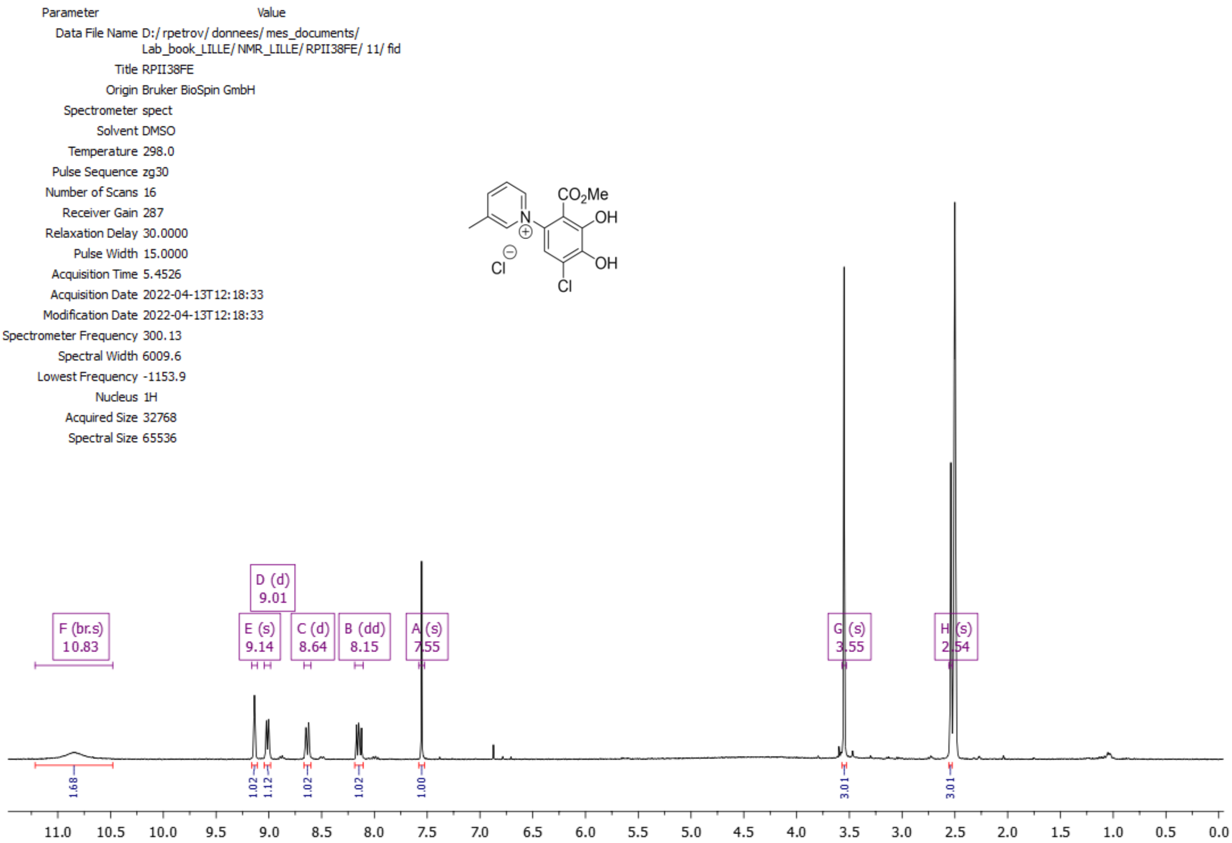

Comp 16a.^{13}C NMR of 16a in DMSO-d_6, Bruker-300

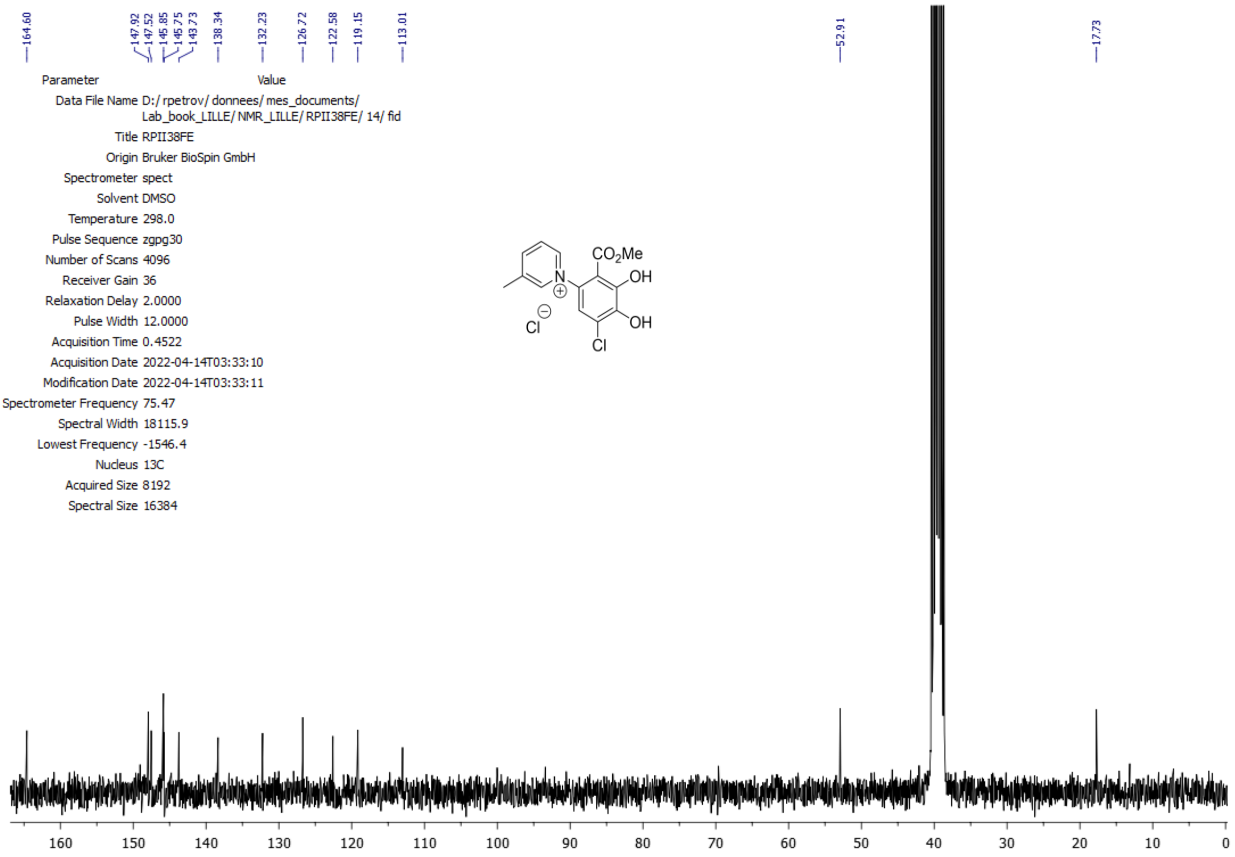

Comp 16a. HRMS (ES+) analysis

Single Mass Analysis
Tolerance = 5.0 mDa / DBE: min = -1.5, max = 50.0
Element prediction: Off
Number of isotope peaks used for i-FIT = 3

Monoisotopic Mass, Even Electron Ions
501 formula(e) evaluated with 11 results within limits (up to 50 best isotopic matches for each mass)
Elements Used:
C: 0-25 H: 0-25 N: 0-10 O: 0-10 Cl: 0-1

RPII38 66 (1.791)

1: TOF MS ES+
4.59e+004

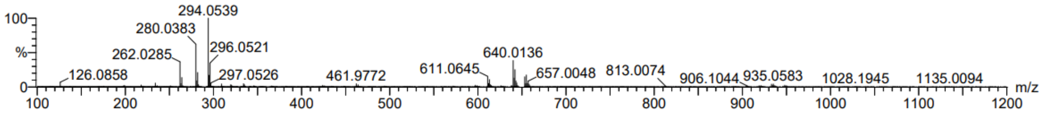

Minimum:				-1.5		
Maximum:		5.0	10.0	50.0		
Mass	Calc. Mass	mDa	PPM	DBE	i-FIT	Formula
294.0539	294.0533	0.6	2.0	8.5	6.9	C14 H13 N O4 Cl
	294.0546	-0.7	-2.4	13.5	47.7	C15 H9 N5 Cl
	294.0506	3.3	11.2	9.5	139.1	C10 H9 N7 O2 Cl
	294.0493	4.6	15.6	4.5	413.4	C9 H13 N3 O6 Cl
	294.0565	-2.6	-8.8	0.5	1484.5	C3 H13 N7 O7 Cl

Comp 16b. ^{1}H NMR of 16b in DMSO-d_6, Bruker-300

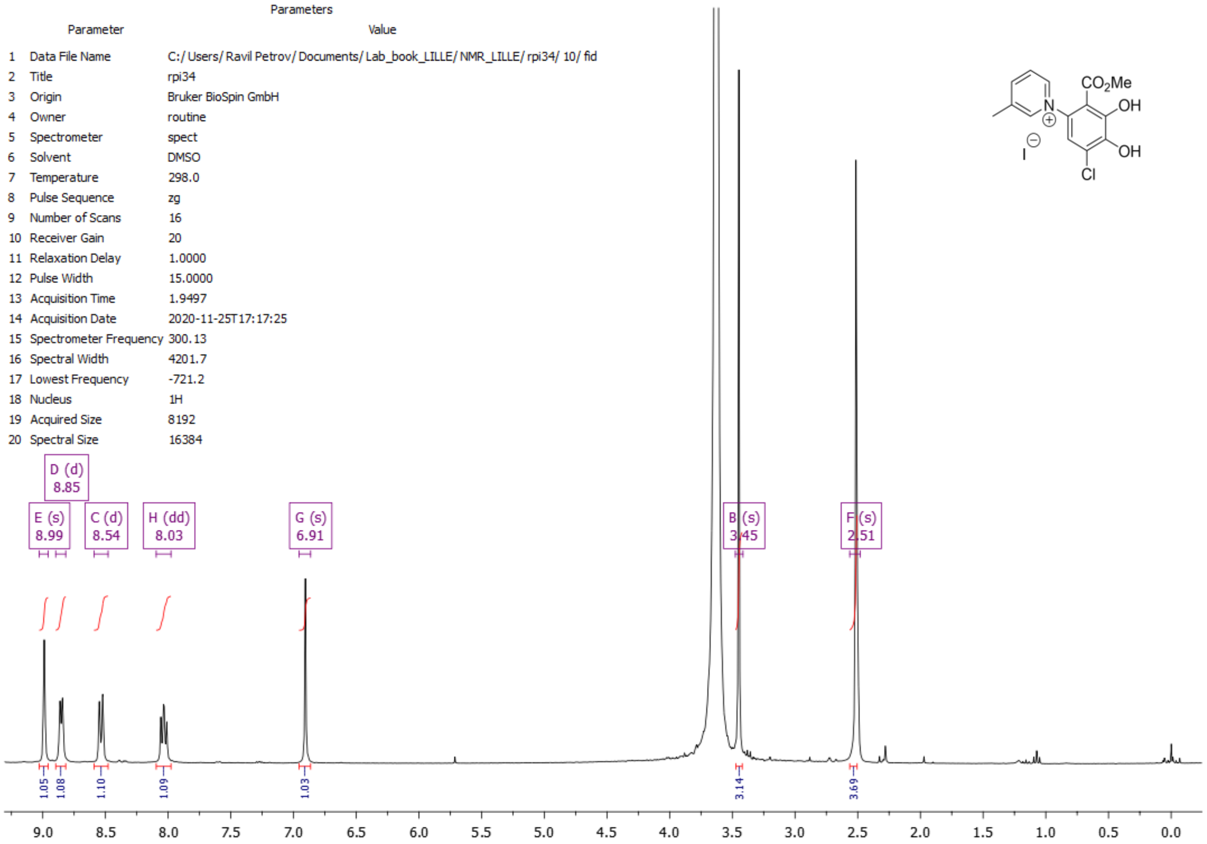

Comp 16b. ^{13}C jmod NMR of 16b in DMSO-d_6, Bruker-300

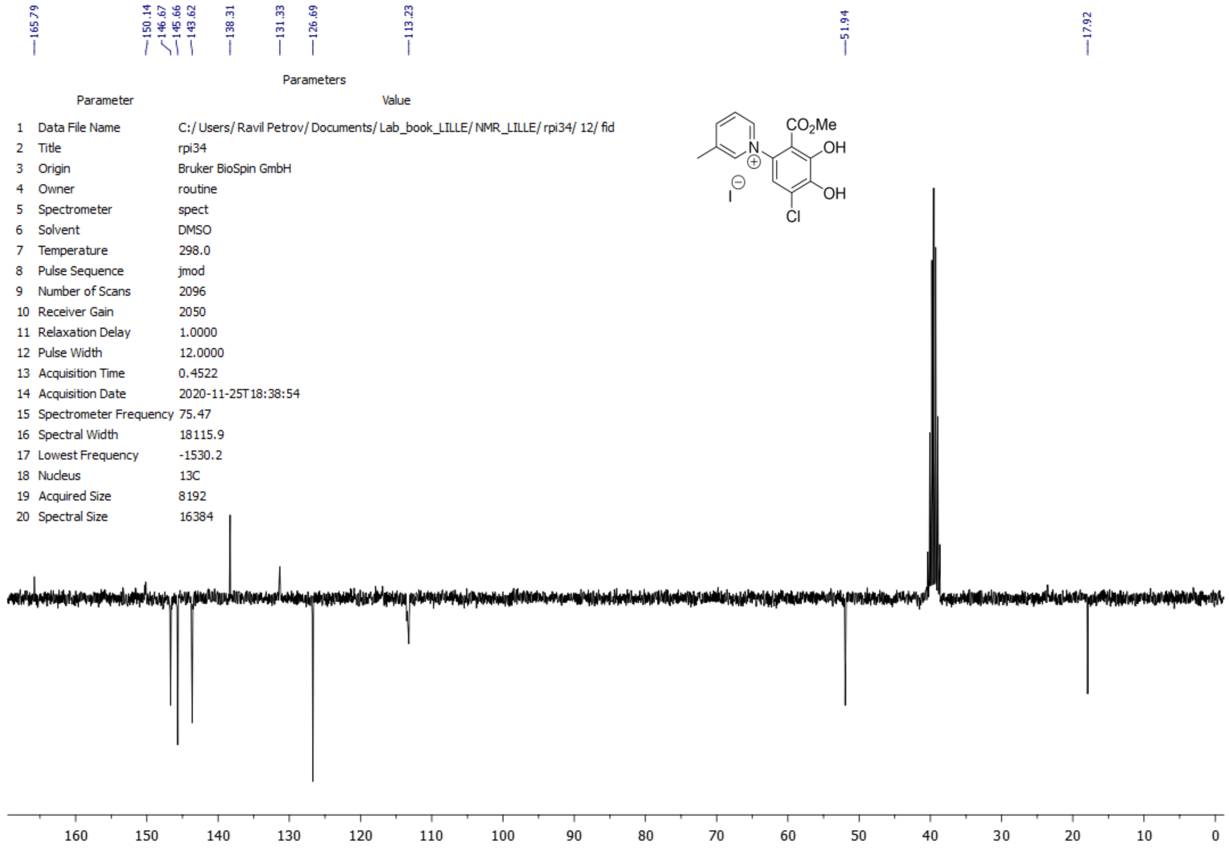

Comp 16b.HRMS (ESI+) analysis

Single Mass Analysis
Tolerance = 5.0 mDa / DBE: min = -1.5, max = 50.0
Element prediction: Off
Number of isotope peaks used for i-FIT = 3

Monoisotopic Mass, Even Electron Ions
501 formula(e) evaluated with 11 results within limits (up to 50 best isotopic matches for each mass)
Elements Used:
C: 0-25 H: 0-25 N: 0-10 O: 0-10 Cl: 0-1

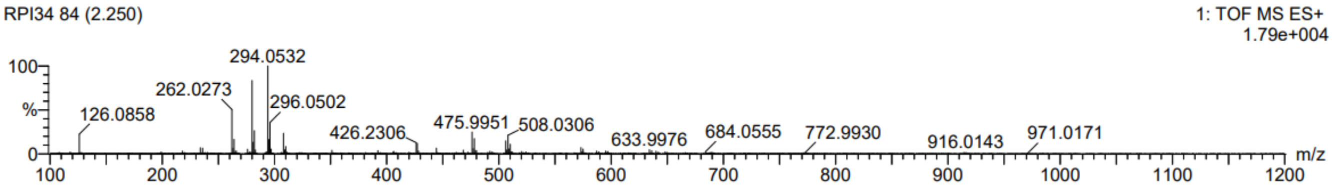

Mass	Calc. Mass	mDa	PPM	DBE	i-FIT	Formula
Minimum:				-1.5		
Maximum:		5.0	10.0	50.0		
294.0532	294.0533	-0.1	-0.3	8.5	4.6	C14 H13 N O4 Cl
	294.0506	2.6	8.8	9.5	36.2	C10 H9 N7 O2 Cl
	294.0546	-1.4	-4.8	13.5	43.8	C15 H9 N5 Cl
	294.0493	3.9	13.3	4.5	123.4	C9 H13 N3 O6 Cl
	294.0565	-3.3	-11.2	0.5	529.7	C3 H13 N7 O7 Cl

Comp 16c. ^{1}H NMR of 16c in DMSO-d_6, Bruker-300

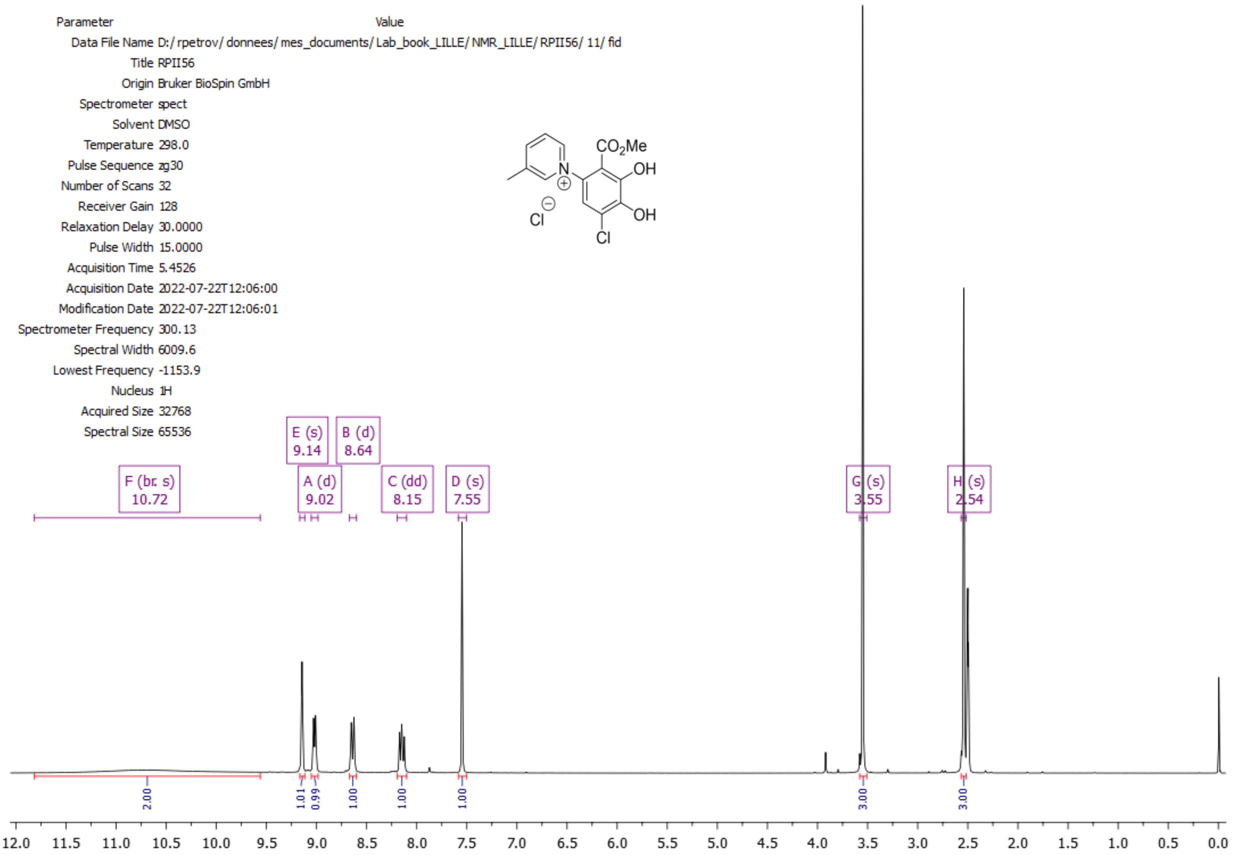

Comp 16c. ^{13}C NMR of 16c in DMSO-d_6, Bruker-300

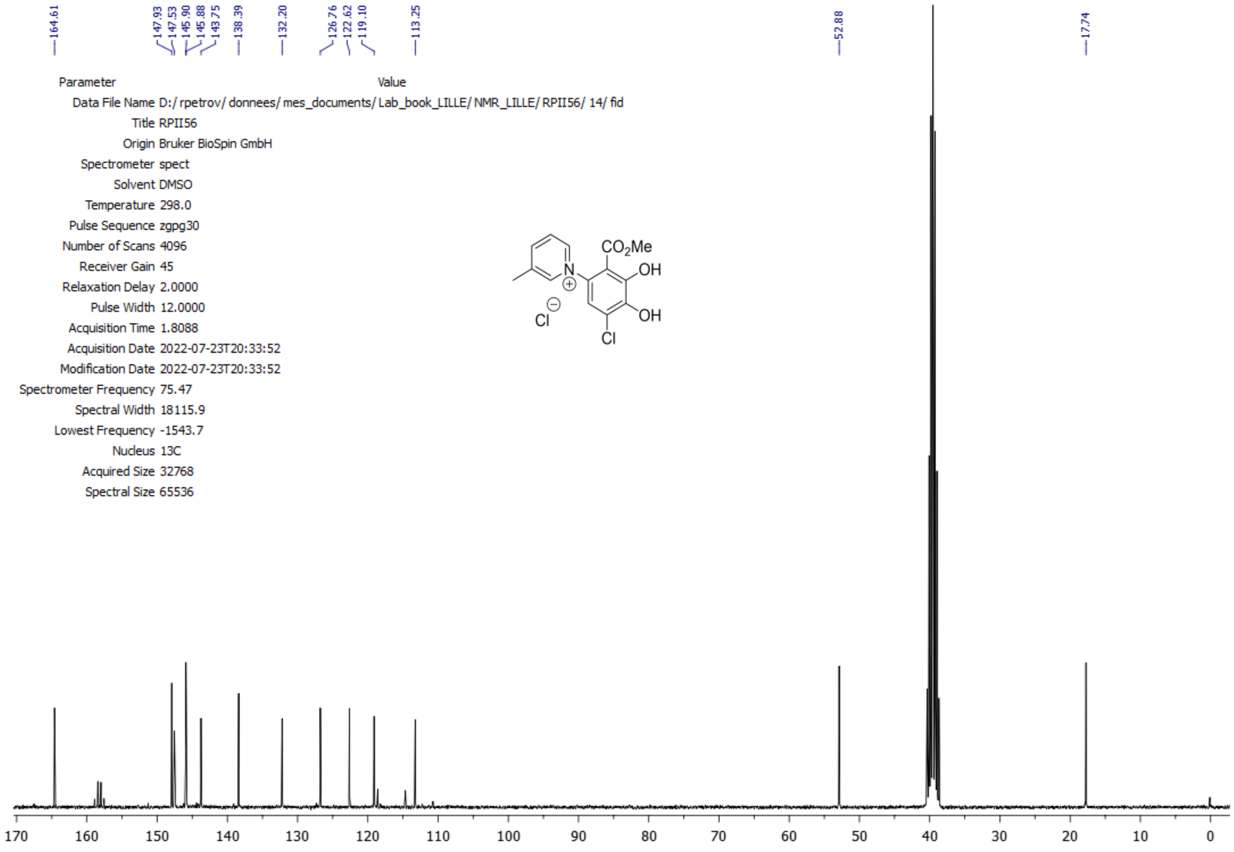

Comp 16c. HRMS (ESI+) analysis.

Single Mass Analysis
Tolerance = 5.0 mDa / DBE: min = -1.5, max = 50.0
Element prediction: Off
Number of isotope peaks used for i-FIT = 3

Monoisotopic Mass, Even Electron Ions
501 formula(e) evaluated with 11 results within limits (up to 50 best isotopic matches for each mass)
Elements Used:
C: 0-25 H: 0-25 N: 0-10 O: 0-10 Cl: 0-1
RPII56 86 (2.297)

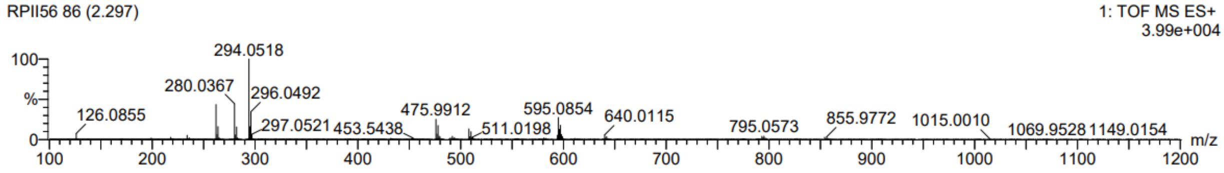

Minimum:				-1.5		
Maximum:		5.0	10.0	50.0		

Mass	Calc. Mass	mDa	PPM	DBE	i-FIT	Formula
294.0518	294.0533	-1.5	-5.1	8.5	9.0	C14 H13 N O4 Cl
	294.0506	1.2	4.1	9.5	49.9	C10 H9 N7 O2 Cl
	294.0546	-2.8	-9.5	13.5	103.3	C15 H9 N5 Cl
	294.0493	2.5	8.5	4.5	237.1	C9 H13 N3 O6 Cl
	294.0565	-4.7	-16.0	0.5	1162.8	C3 H13 N7 O7 Cl

NMR and HRMS Compound 17, Bruker-300

Comp 17b. ^{1}H NMR of 17b in DMSO-d_6, Bruker-300

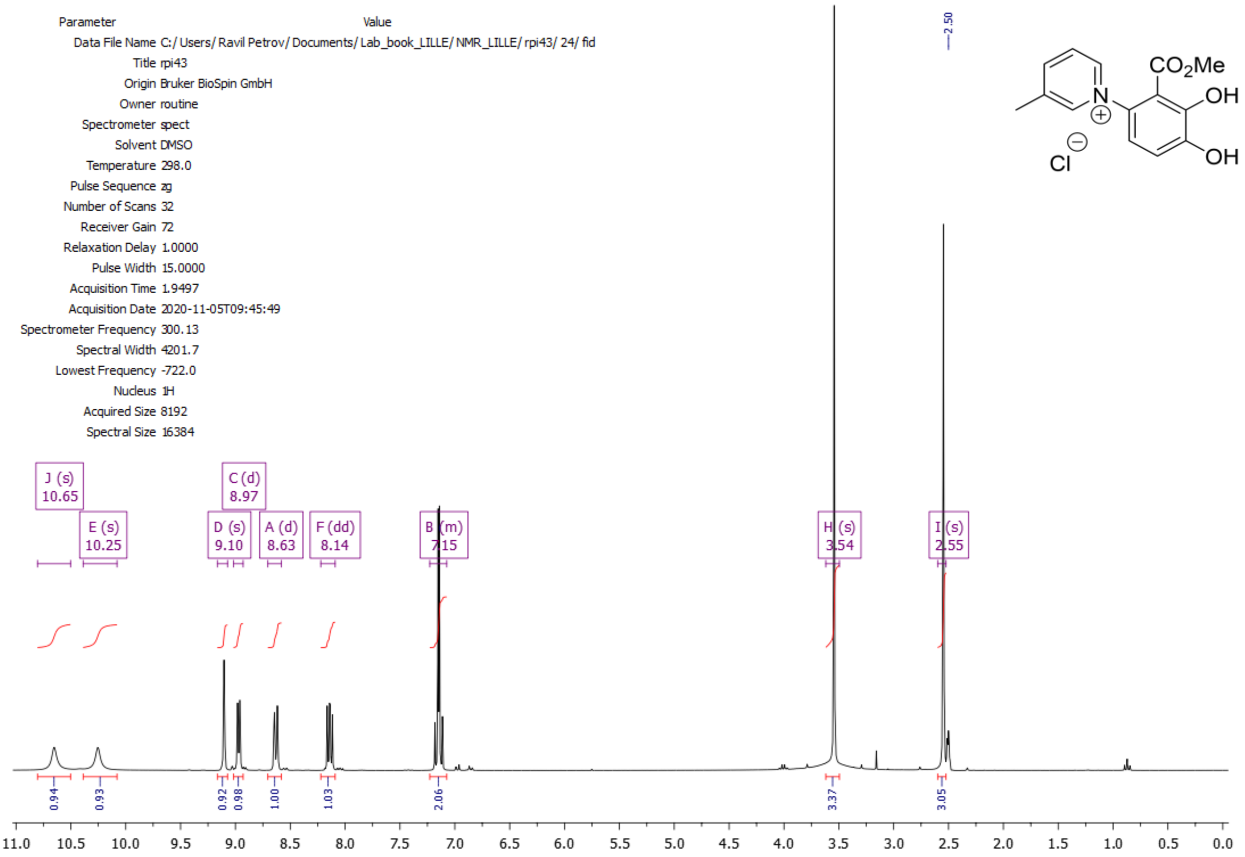

Comp 17b. ^{13}C NMR of 17b in DMSO-d_6, Bruker-300

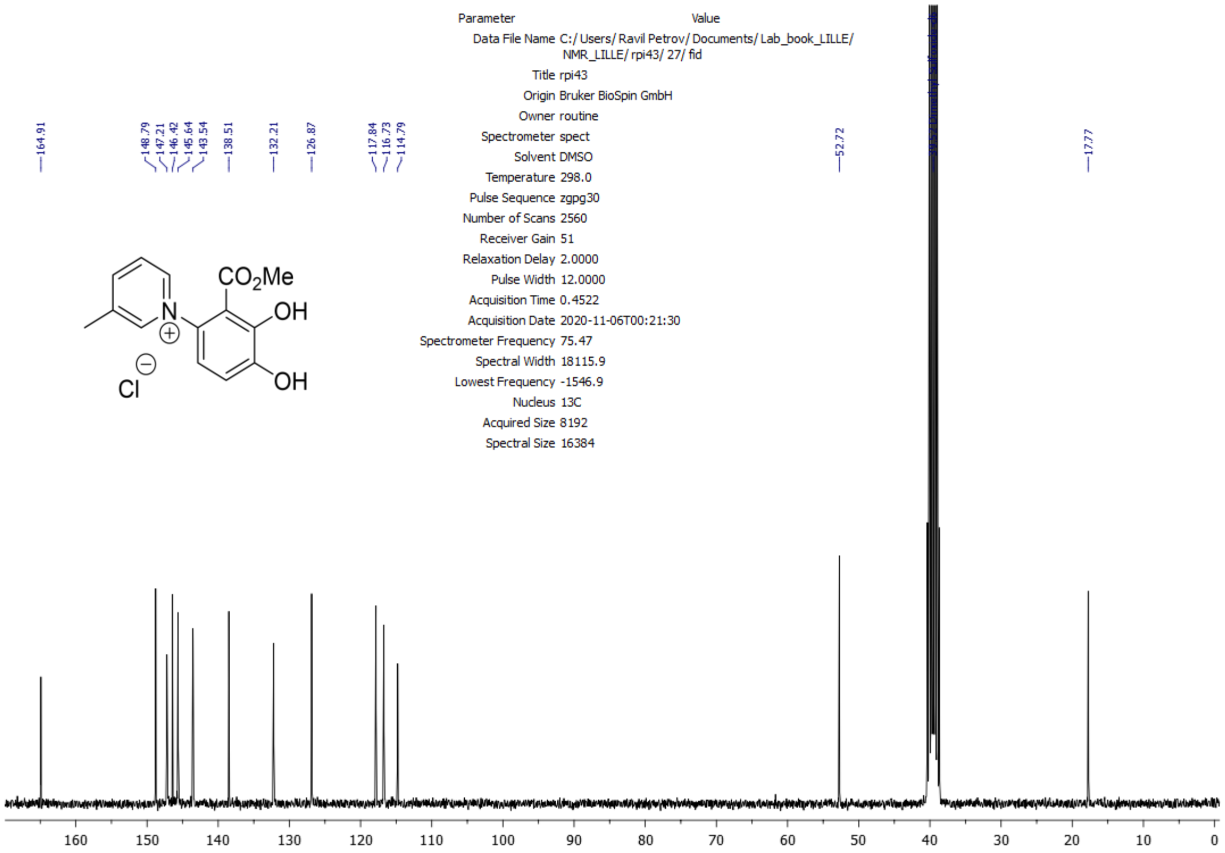

Comp 17b. HRMS (ESI+) analysis

Single Mass Analysis
Tolerance = 10.0 mDa / DBE: min = -1.5, max = 50.0
Element prediction: Off
Number of isotope peaks used for i-FIT = 2

Monoisotopic Mass, Even Electron Ions
351 formula(e) evaluated with 16 results within limits (up to 50 closest results for each mass)
Elements Used:
C: 0-20 H: 0-20 N: 0-10 O: 1-10 Cl: 0-1
RPI43 51 (1.402)

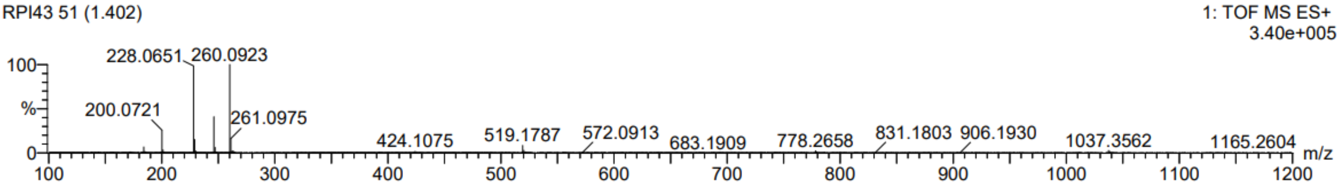

Minimum:				-1.5		
Maximum:		10.0	10.0	50.0		
Mass	Calc. Mass	mDa	PPM	DBE	i-FIT	Formula
260.0923	260.0923	0.0	0.0	8.5	20.4	C14 H14 N O4
	260.0914	0.9	3.5	4.5	1434.0	C9 H15 N5 O2 Cl
	260.0901	2.2	8.5	-0.5	3912.3	C8 H19 N O6 Cl
	260.0896	2.7	10.4	9.5	556.7	C10 H10 N7 O2
	260.0955	-3.2	-12.3	0.5	9620.7	C3 H14 N7 O7

NMR and HRMS Compound 18, Bruker-300

Comp 18a. ^{1}H NMR of 18a in DMSO-d_6, Bruker-300

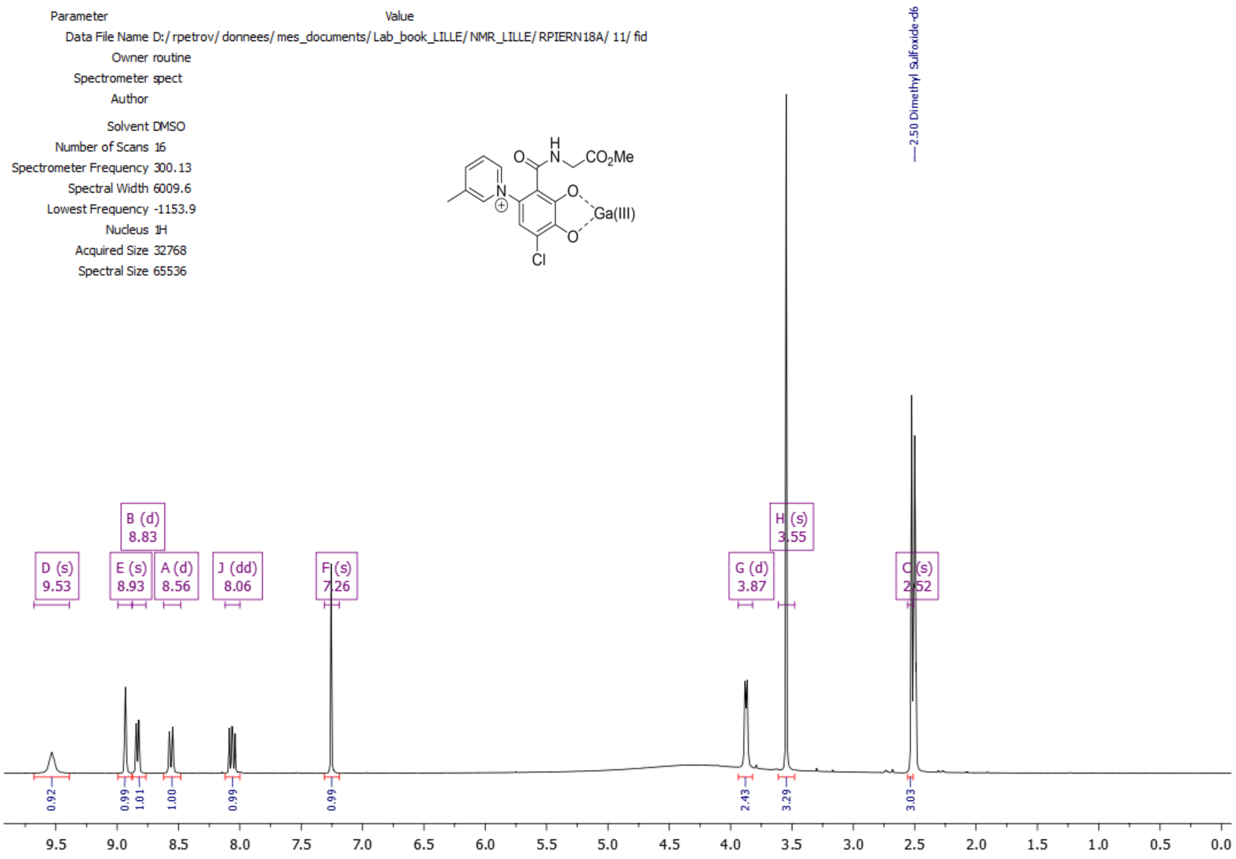

^{13}C NMR of 18a in DMSO-d_6, Bruker-300

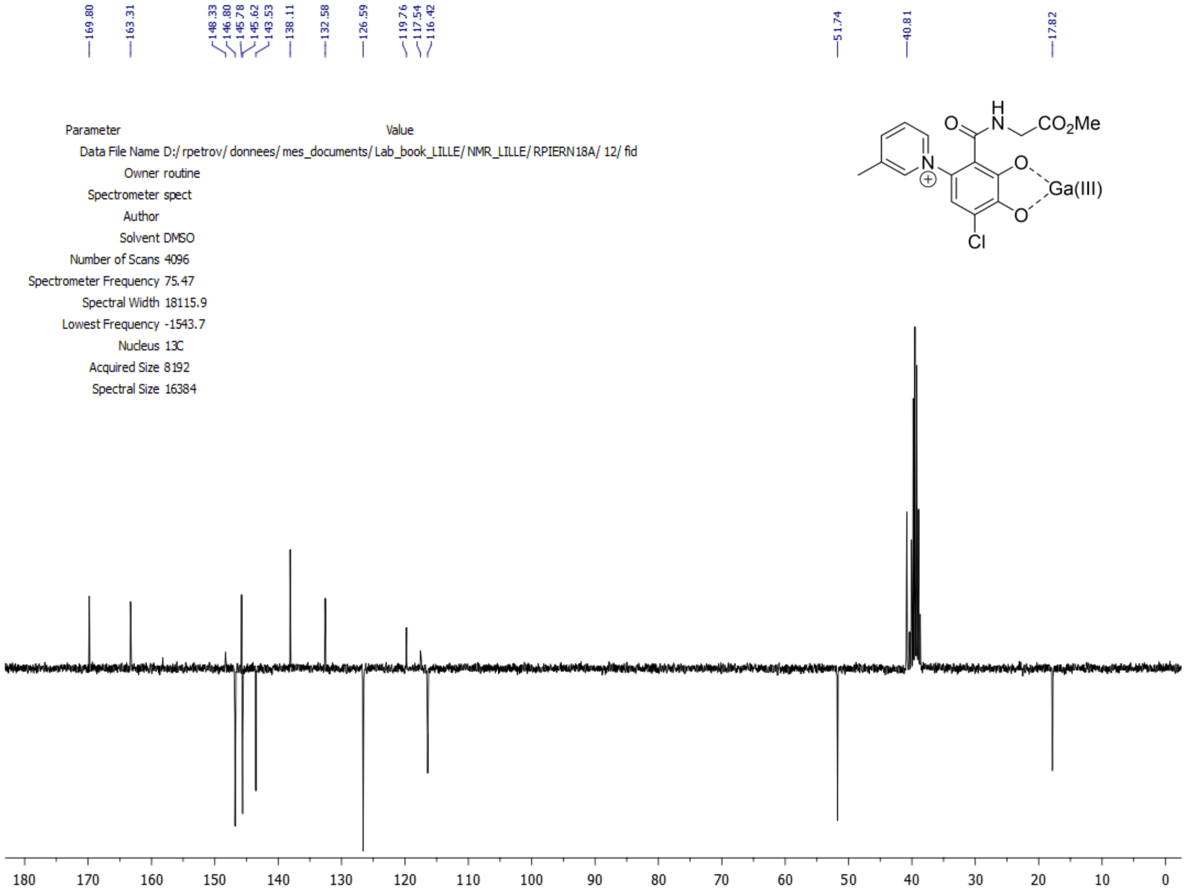

Comp 18a. HRMS (ES+) analysis

Single Mass Analysis
Tolerance = 10.0 mDa / DBE: min = -1.5, max = 50.0
Element prediction: Off
Number of isotope peaks used for i-FIT = 2

Monoisotopic Mass, Even Electron Ions
372 formula(e) evaluated with 13 results within limits (up to 50 best isotopic matches for each mass)
Elements Used:
C: 0-20 H: 0-25 N: 0-5 O: 0-10 Cl: 0-2

NCat3Pic_pre 67 (1.814) 1: TOF MS ES+ 1.34e+005

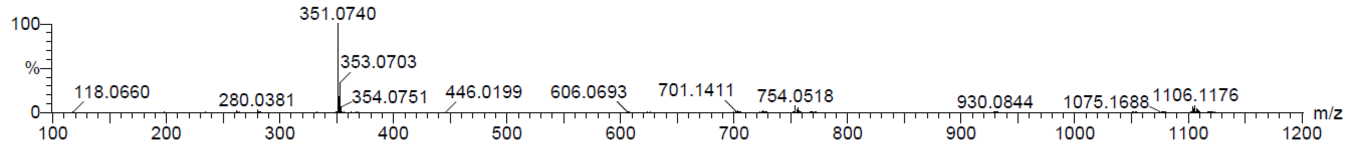

Mass	Calc. Mass	mDa	PPM	DBE	i-FIT	Formula
Minimum:				-1.5		
Maximum:		10.0	10.0	50.0		
351.0740	351.0748	-0.8	-2.3	9.5	37.8	C16 H16 N2 O5 Cl
	351.0729	1.1	3.1	14.5	247.4	C17 H11 N4 O5
	351.0726	1.4	4.0	0.5	1031.4	C10 H21 N2 O7 Cl2
	351.0716	2.4	6.8	9.5	26.8	C16 H15 O9
	351.0766	-2.6	-7.4	4.5	40.8	C15 H21 O5 Cl2
	351.0708	3.2	9.1	5.5	495.2	C11 H16 N4 O7 Cl
	351.0779	-3.9	-11.1	9.5	139.3	C16 H17 N4 O Cl2
	351.0788	-4.8	-13.7	5.5	833.2	C10 H15 N4 O10
	351.0806	-6.6	-18.8	0.5	1617.1	C9 H20 N2 O10 Cl
	351.0667	7.3	20.8	9.5	320.1	C17 H17 N2 O2 Cl2
	351.0828	-8.8	-25.1	9.5	280.1	C15 H15 N2 O8
	351.0649	9.1	25.9	14.5	734.8	C18 H12 N4 O2 Cl
	351.0838	-9.8	-27.9	0.5	1530.0	C9 H21 N4 O6 Cl2

Comp 18b. ^{1}H NMR of 18b in DMSO-d_6, Bruker-300

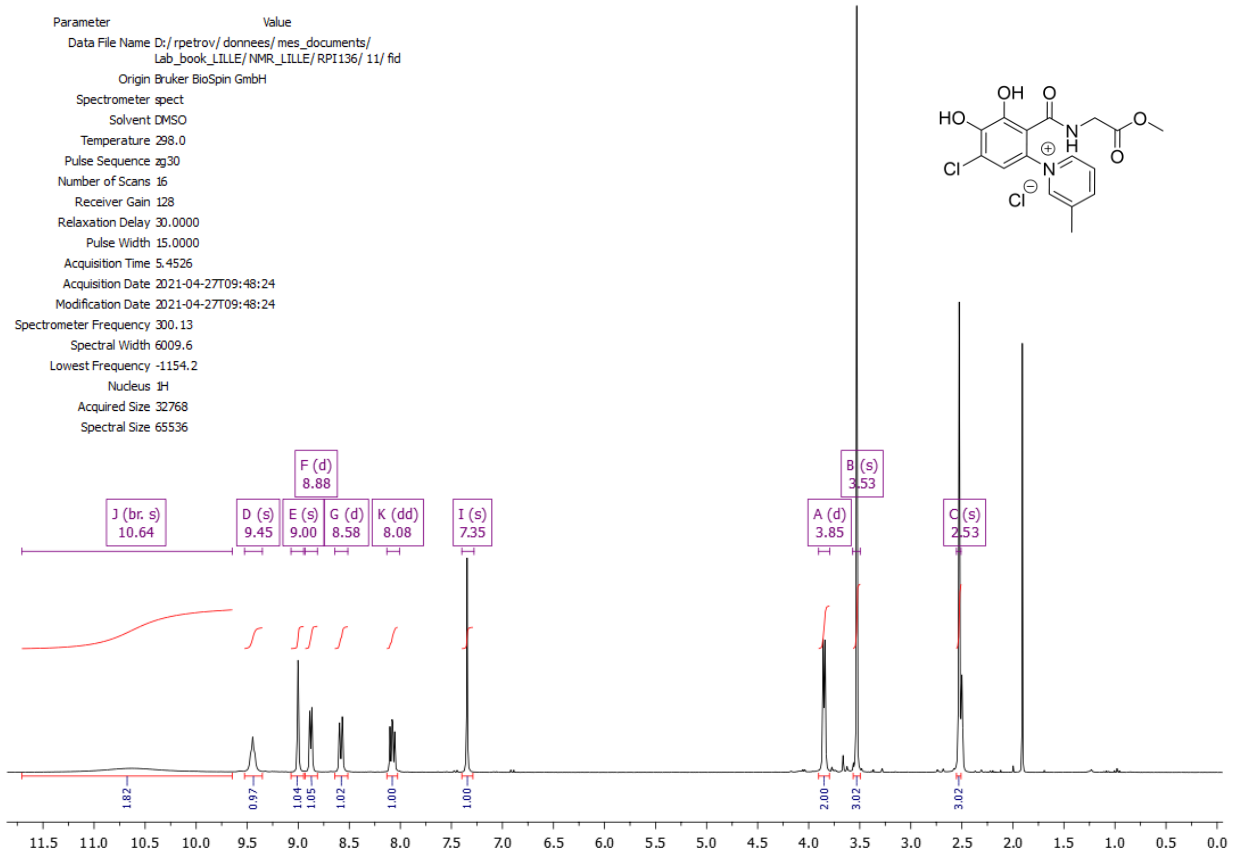

Comp 18b. ^{13}C jmod NMR of 18b in DMSO-d_6, Bruker-300

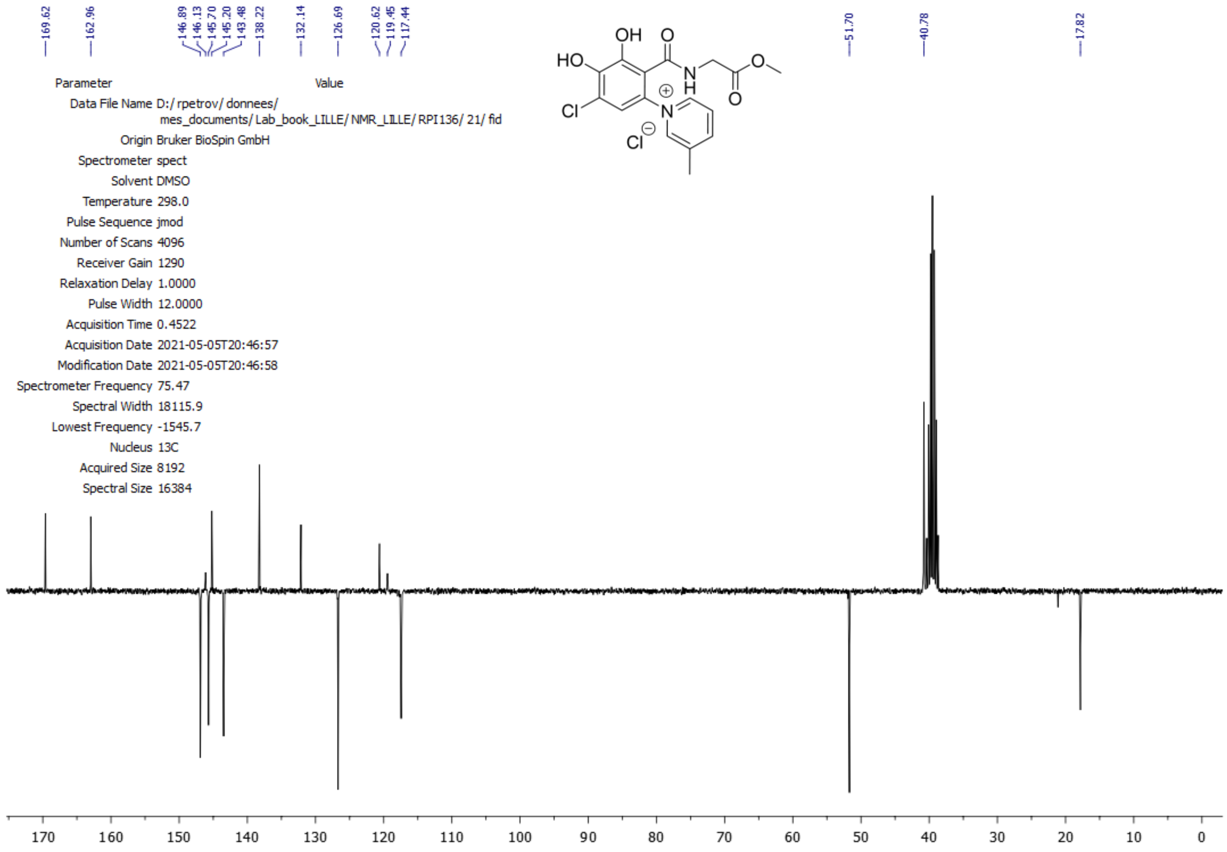

Comp 18b. HRMS (ES+) analysis

Single Mass Analysis

Tolerance = 5.0 mDa / DBE: min = -1.5, max = 50.0
Element prediction: Off
Number of isotope peaks used for i-FIT = 3

Monoisotopic Mass, Even Electron Ions
471 formula(e) evaluated with 11 results within limits (up to 50 best isotopic matches for each mass)
Elements Used:
C: 0-20 H: 0-25 N: 0-10 O: 0-10 Cl: 0-1

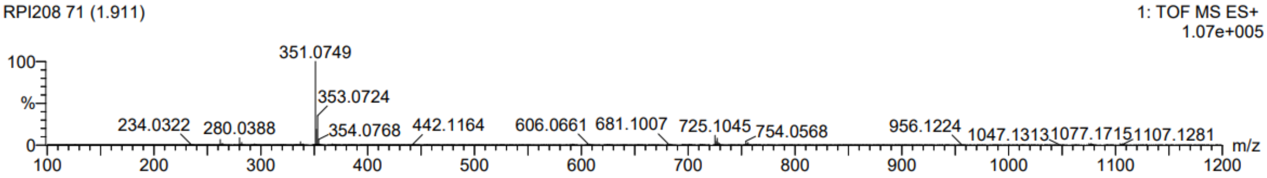

Minimum:				-1.5		
Maximum:		5.0	10.0	50.0		
Mass	Calc. Mass	mDa	PPM	DBE	i-FIT	Formula
351.0749	351.0748	0.1	0.3	9.5	1.3	C16 H16 N2 O5 Cl
	351.0761	-1.2	-3.4	14.5	157.5	C17 H12 N6 O Cl
	351.0721	2.8	8.0	10.5	172.5	C12 H12 N8 O3 Cl
	351.0708	4.1	11.7	5.5	639.7	C11 H16 N4 O7 Cl
	351.0780	-3.1	-8.8	1.5	2679.1	C5 H16 N8 O8 Cl
	[illegible]	[illegible]	[illegible]	[illegible]	[illegible]	[illegible]

Comp 18d. ^{1}H NMR of 18d in MeOH-d_4, Bruker-300

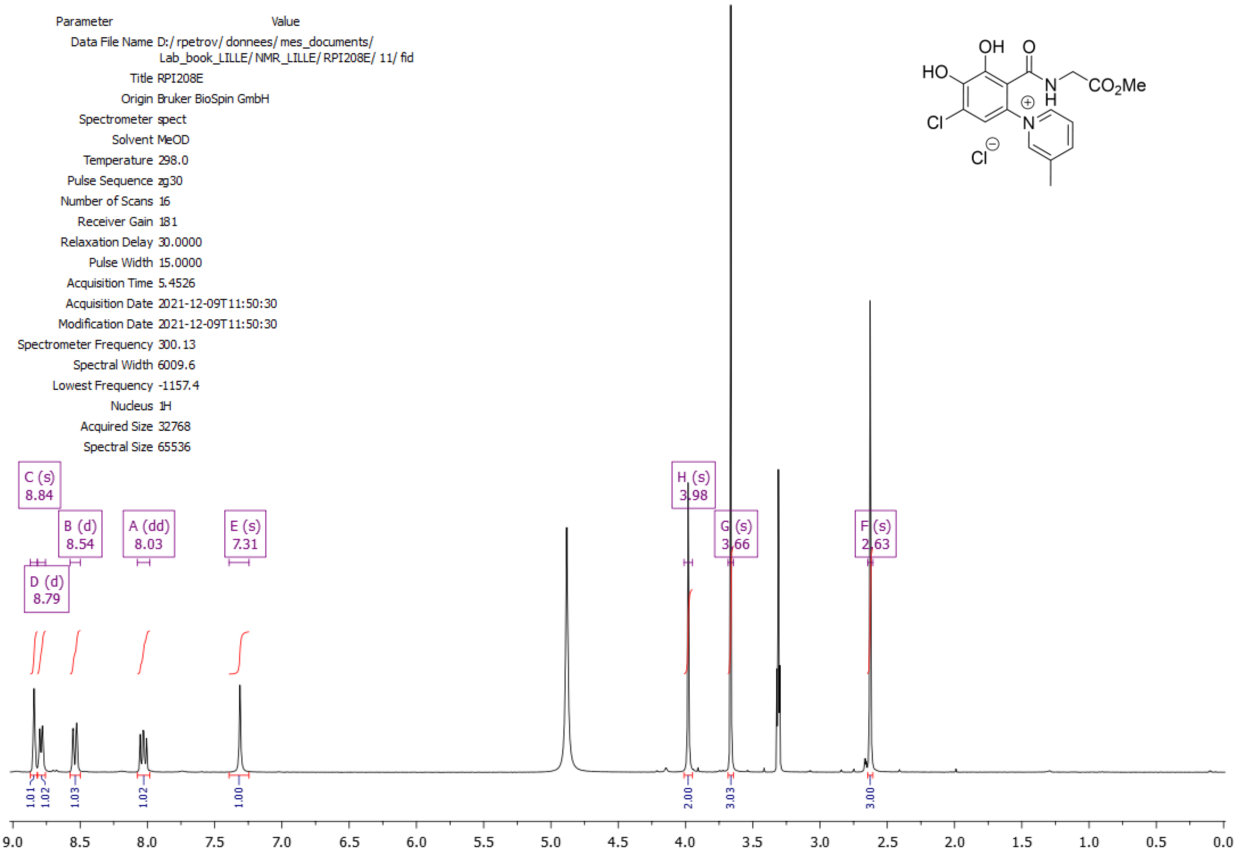

Comp 18d. ^{13}C NMR of 18d in MeOH-d_4, Bruker-300

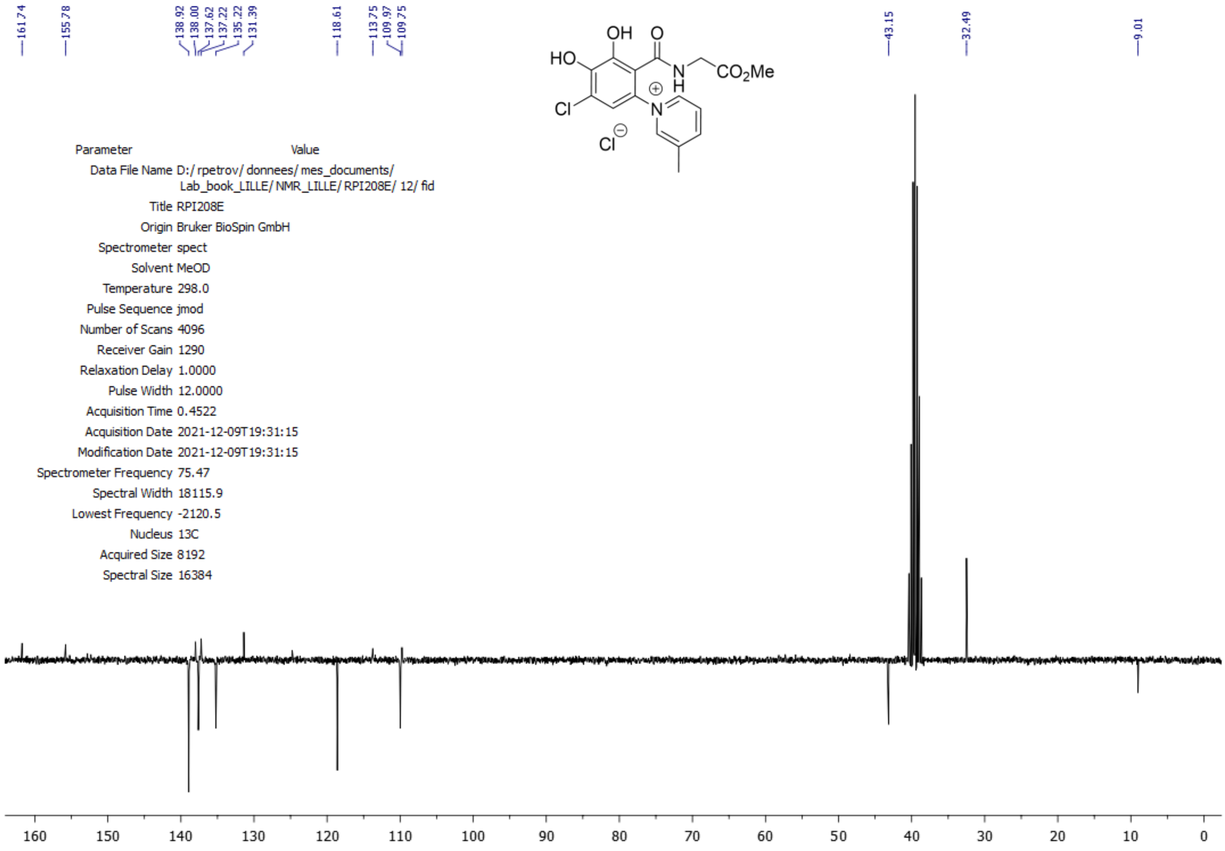

Comp 18d. HRMS (ESI+) analysis

Single Mass Analysis
Tolerance = 5.0 mDa / DBE: min = -1.5, max = 50.0
Element prediction: Off
Number of isotope peaks used for i-FIT = 3

Monoisotopic Mass, Even Electron Ions
471 formula(e) evaluated with 11 results within limits (up to 50 best isotopic matches for each mass)
Elements Used:
C: 0-20 H: 0-25 N: 0-10 O: 0-10 Cl: 0-1
RPI208 73 (1.958)

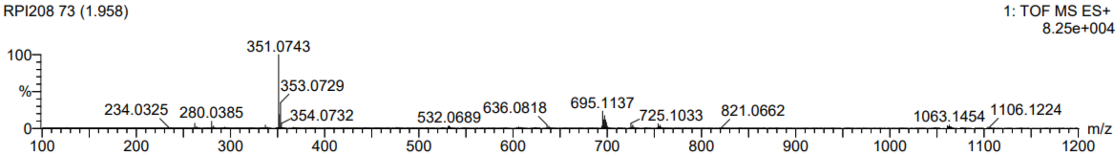

Minimum:				-1.5		
Maximum:		5.0	10.0	50.0		
Mass	Calc. Mass	mDa	PPM	DBE	i-FIT	Formula
351.0743	351.0748	-0.5	-1.4	9.5	2.1	C16 H16 N2 O5 Cl
	351.0721	2.2	6.3	10.5	104.9	C12 H12 N8 O3 Cl
	351.0761	-1.8	-5.1	14.5	142.9	C17 H12 N6 O Cl
	351.0708	3.5	10.0	5.5	447.4	C11 H16 N4 O7 Cl
	351.0780	-3.7	-10.5	1.5	2002.4	C5 H16 N8 O8 Cl

Comp 18e. ^{1}H NMR of 18e in DMSO-d_6, Bruker-600

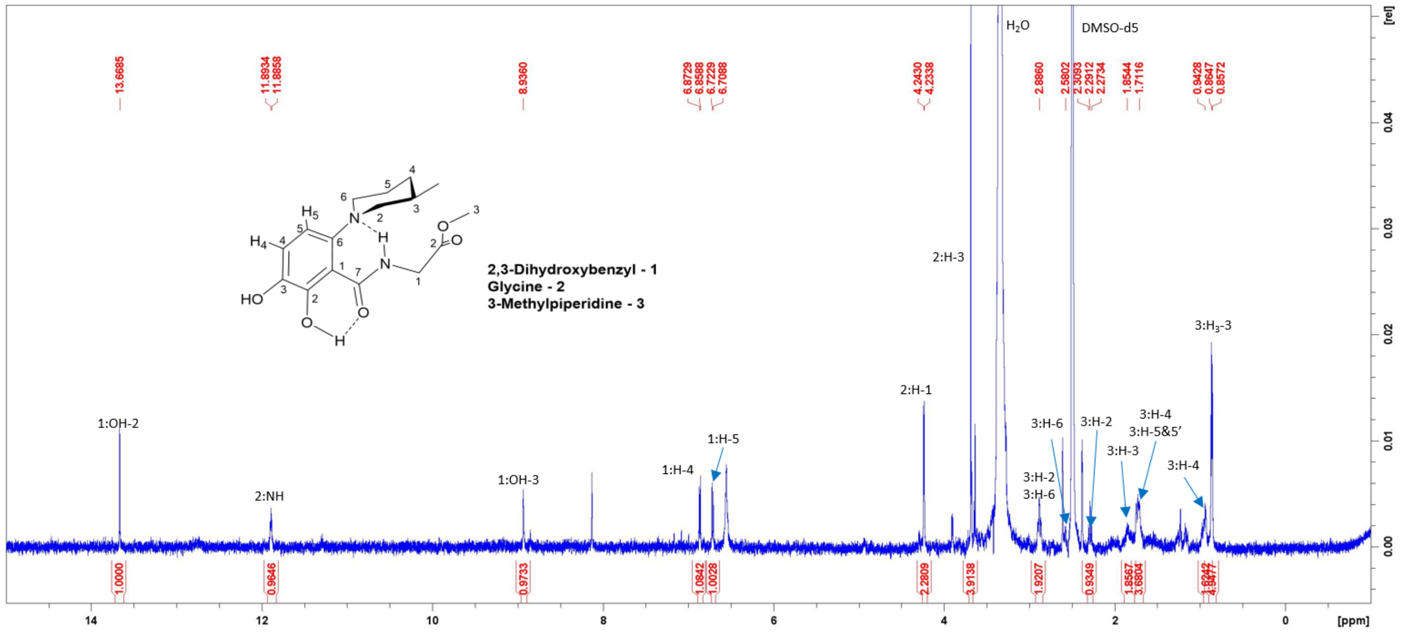

Superimposition of ^{1}H NMR of 18e (blue) *vs*. 18f (red) in DMSO-d_6, Bruker-600

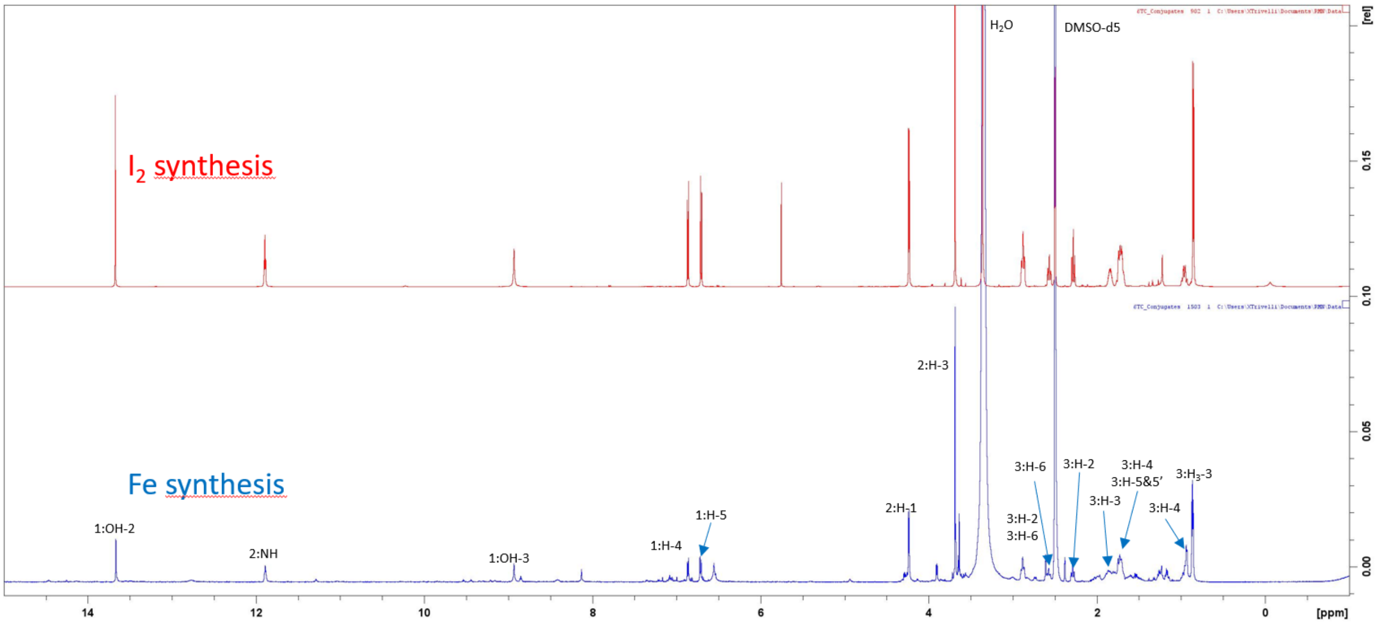

2D ^{1}H-COSY NMR of 18e in DMSO-d_6, Bruker-600

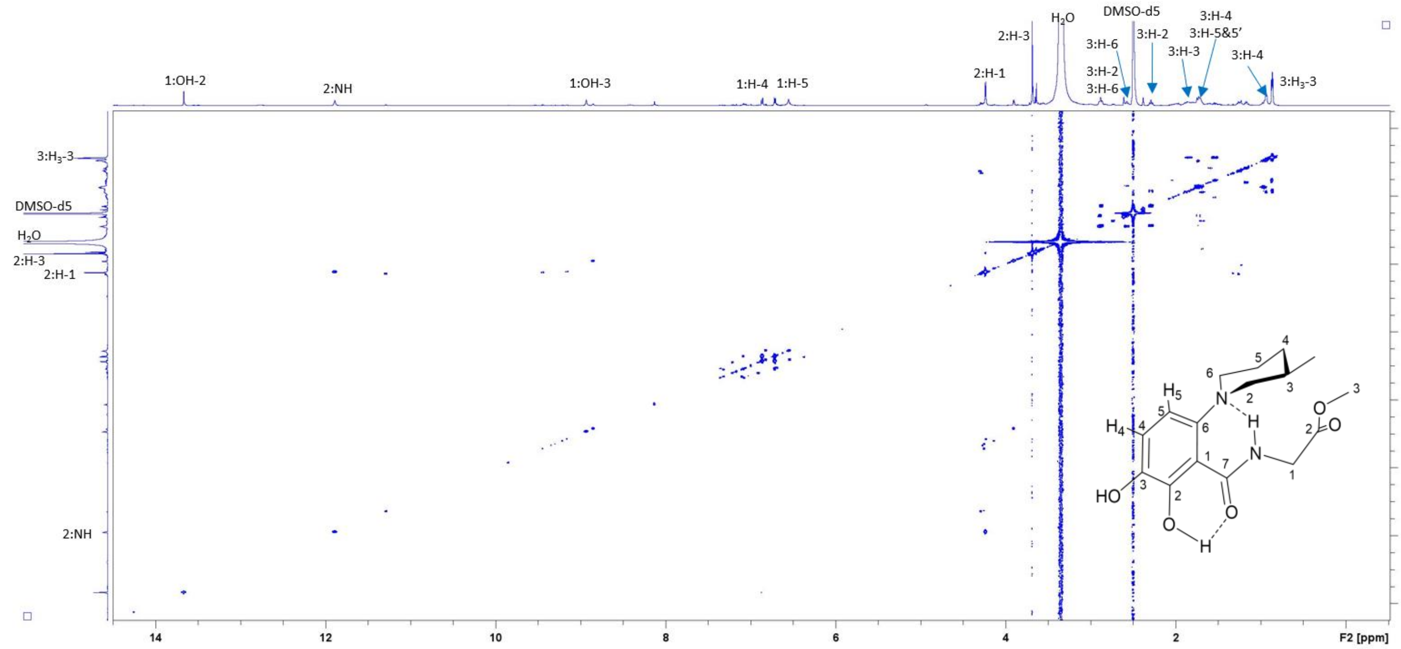

2D ^{1}H-^{13}C-HSQC-DEPT NMR of 18e in DMSO-d_6, Bruker-600

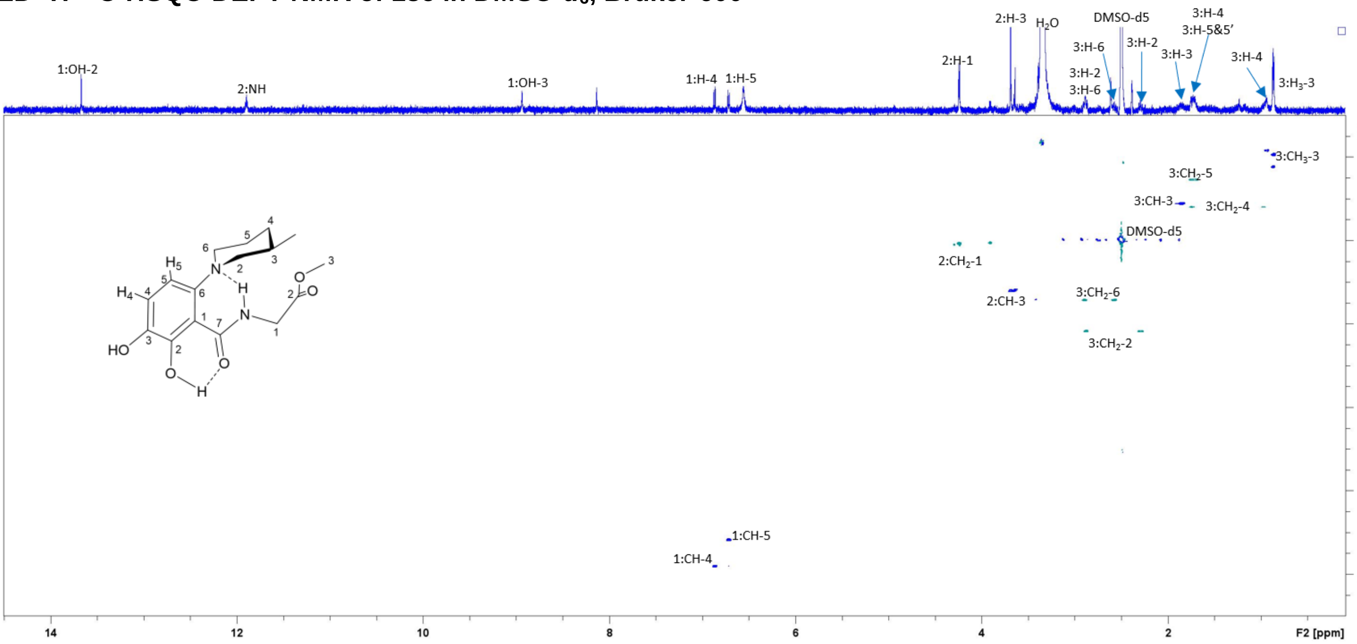

Superimposition of 2D ^{1}H-^{13}C-HSQC-DEPT NMR of 18e *vs*. 18f in DMSO-d_6, Bruker-600: CH/CH_3 (red), CH_2 (purple)

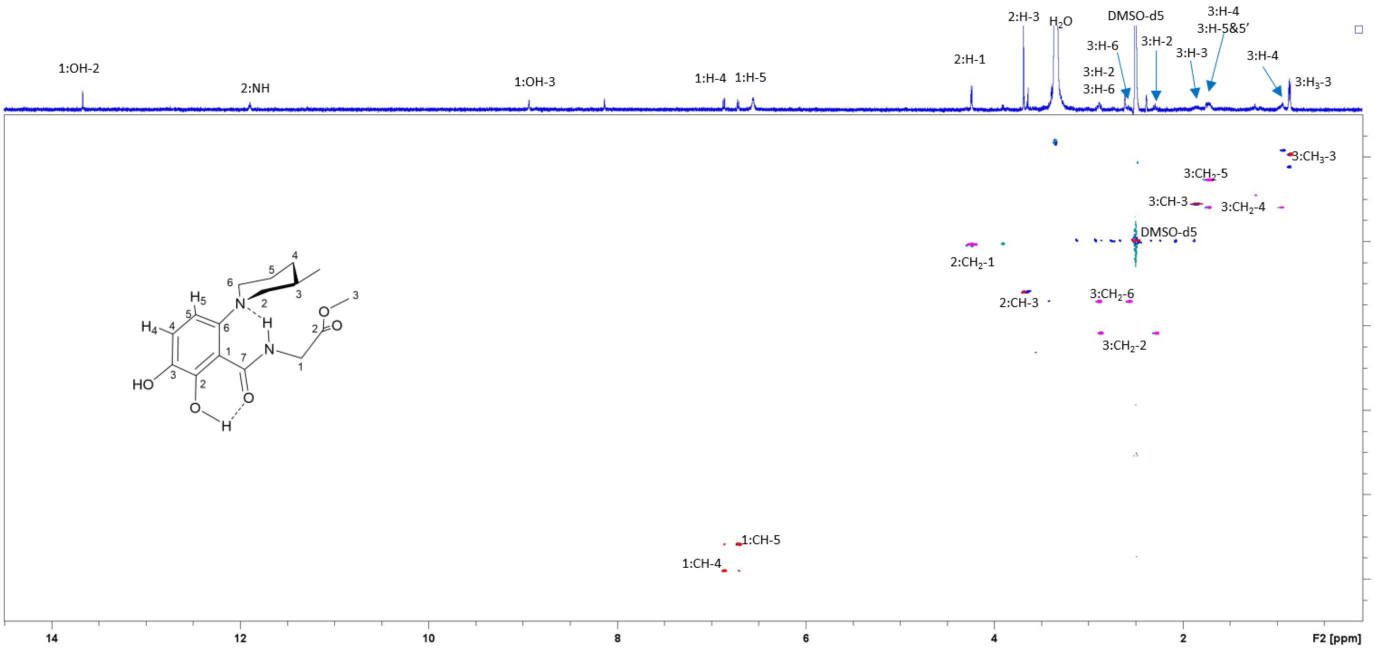

2D ^{1}H-^{13}C-HSQC-TOCSY (T_m = 27 ms) NMR of 18e in DMSO-d_6, Bruker-600

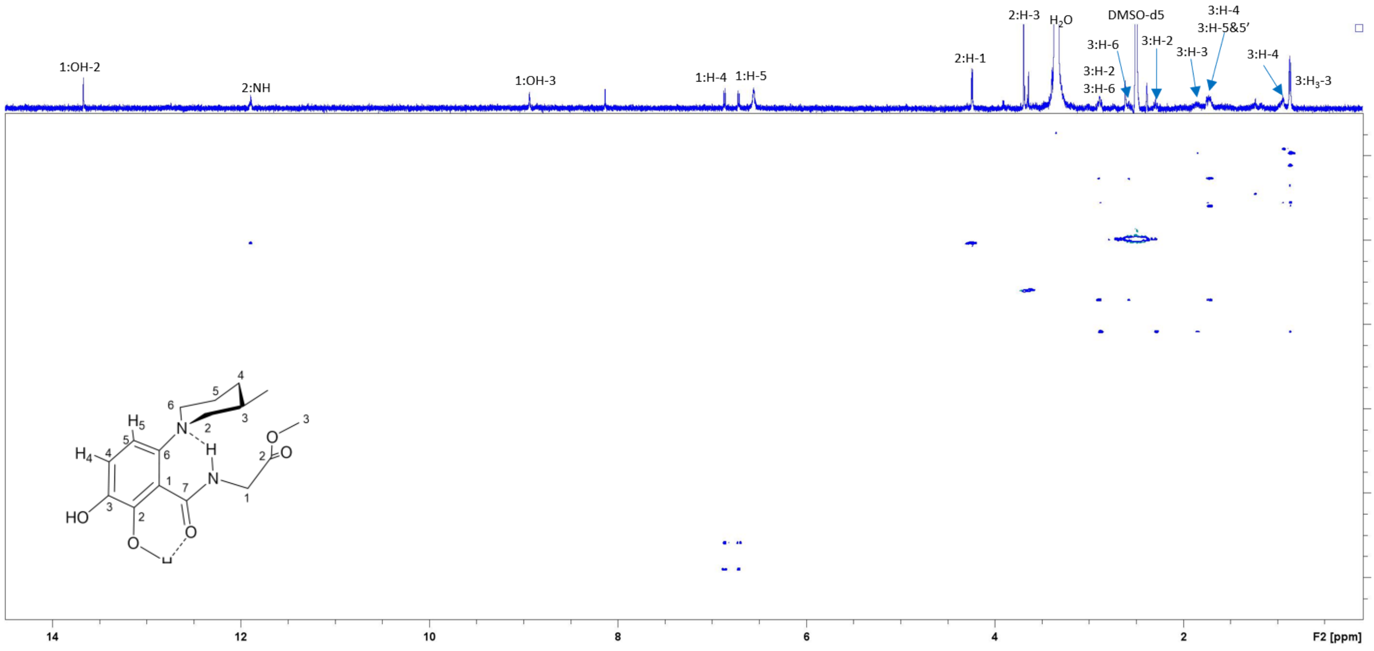

HRMS (ES+) analysis of 18e

Single Mass Analysis
Tolerance = 10.0 mDa / DBE: min = -1.5, max = 50.0
Element prediction: Off
Number of isotope peaks used for i-FIT = 2

Monoisotopic Mass, Even Electron Ions
125 formula(e) evaluated with 3 results within limits (up to 50 best isotopic matches for each mass)
Elements Used:
C: 0-20 H: 0-25 N: 0-5 O: 0-10

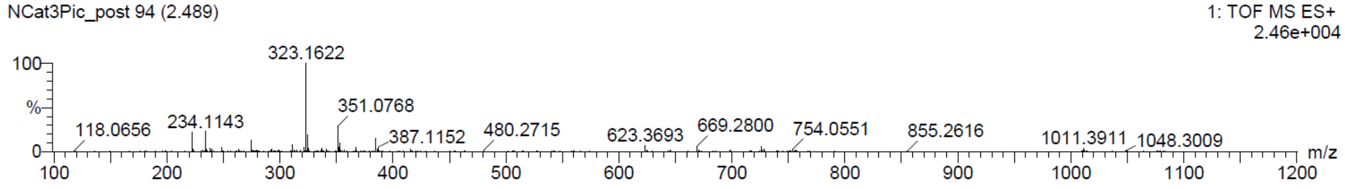

Minimum:				-1.5		
Maximum:		10.0	10.0	50.0		
Mass	Calc. Mass	mDa	PPM	DBE	i-FIT	Formula
323.1622	323.1607	1.5	4.6	6.5	2.2	C16 H23 N2 O5
	323.1719	-9.7	-30.0	6.5	77.6	C15 H23 N4 O4
	323.1567	5.5	17.0	2.5	172.9	C11 H23 N4 O7

Comp 18f. ^{1}H NMR of 18f in DMSO-d_6, Bruker-300

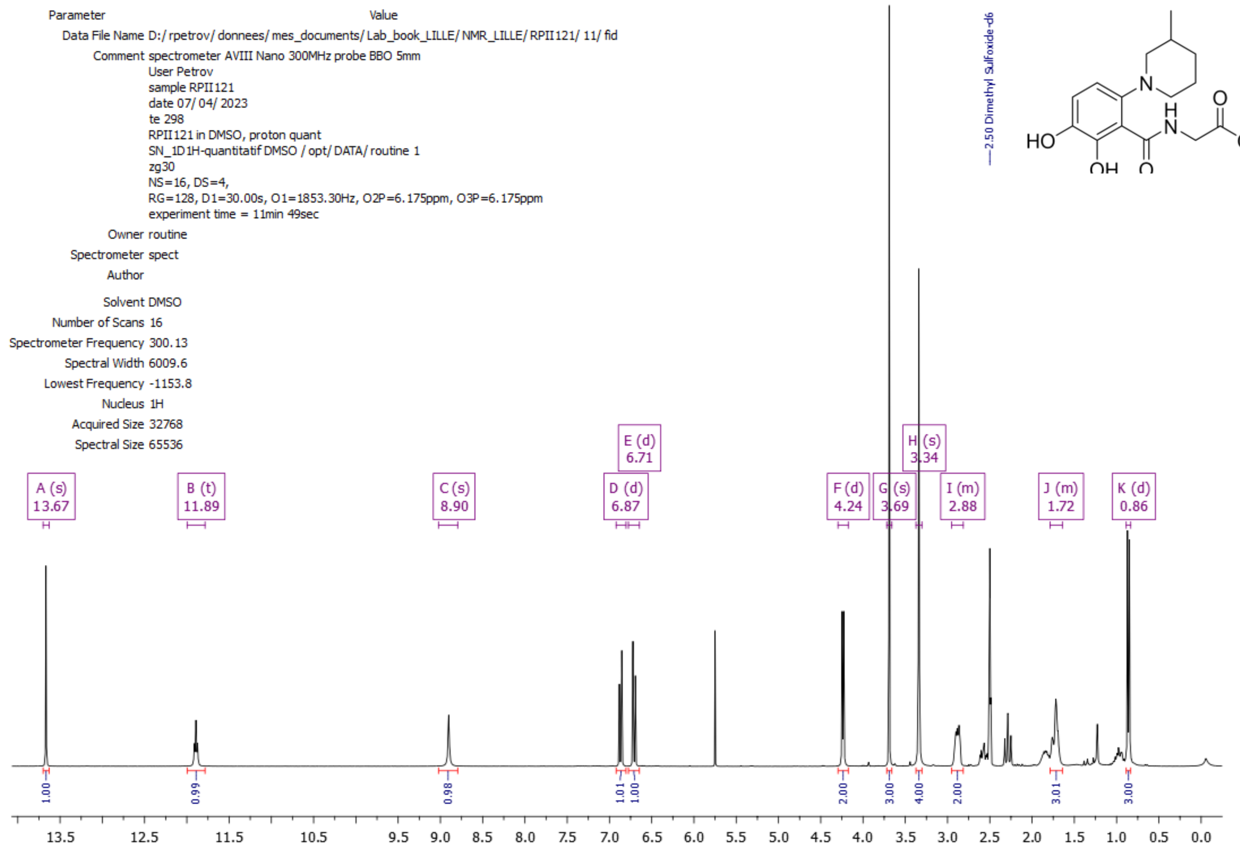

Comp 18f. ^{13}C jmod NMR of 18f in DMSO-d_6, Bruker-300

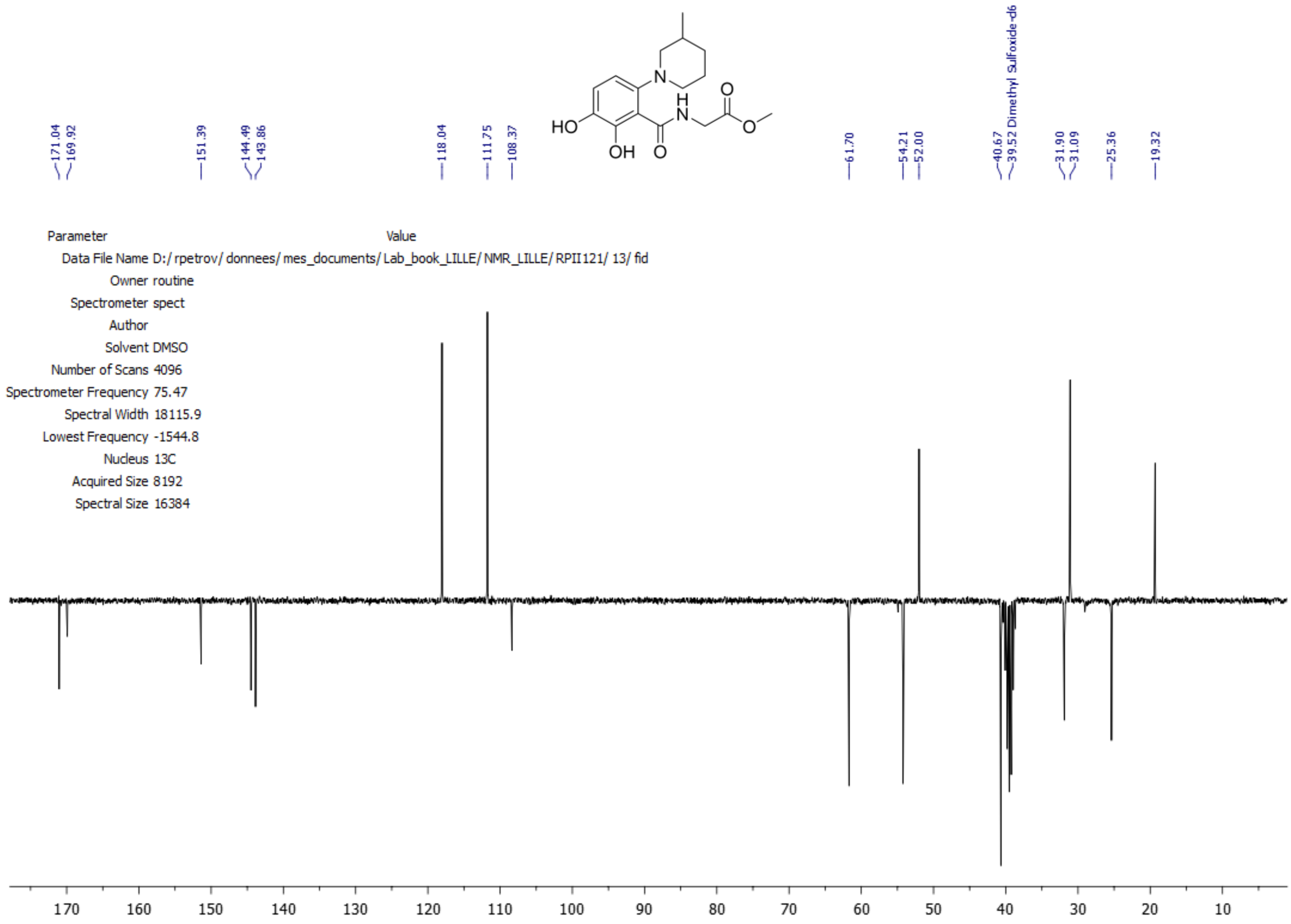

Comp 18f. ^{1}H NMR of 18f in DMSO-d_6, Bruker-600

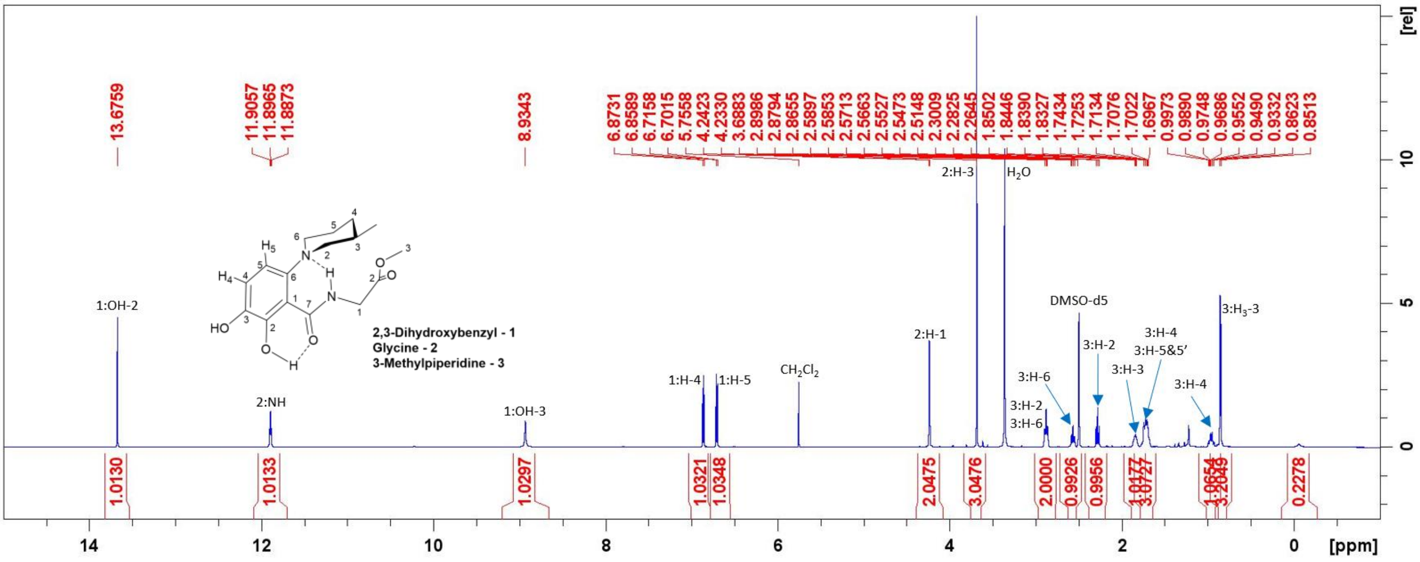

Comp 18f. 2D ^{1}H-COSY NMR of 18f in DMSO-d_6, Bruker-600

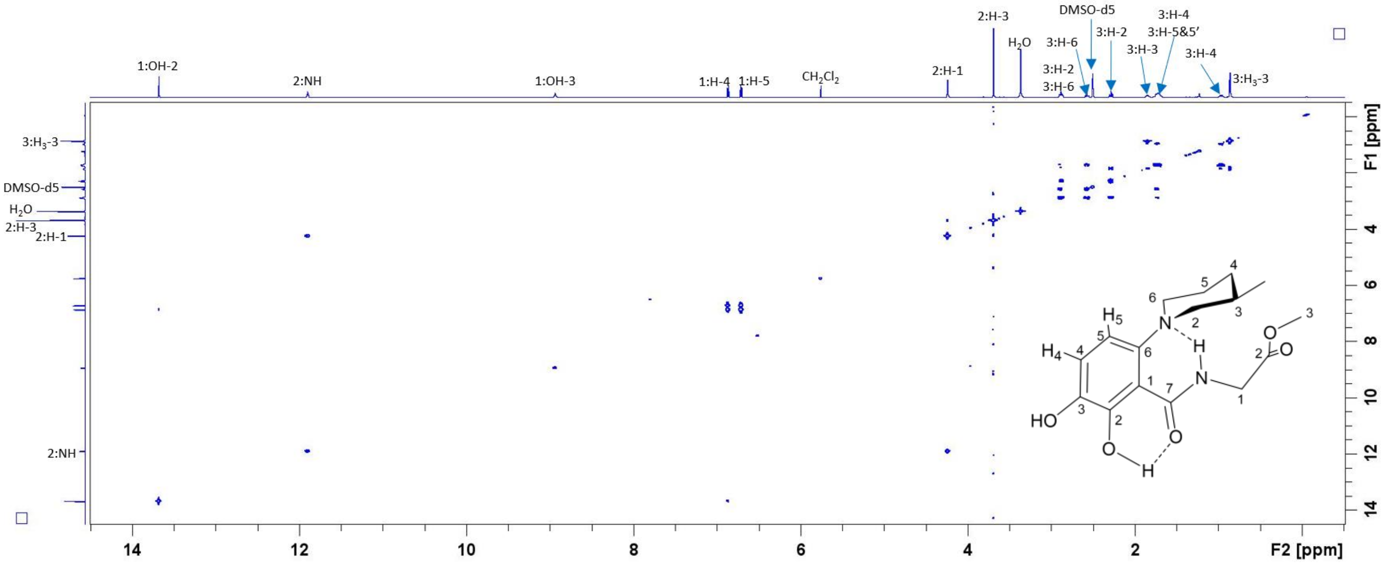

Comp 18f. 2D ^{1}H-NOESY (T_m = 300 ms) NMR of 18f in DMSO-d_6, Bruker-600

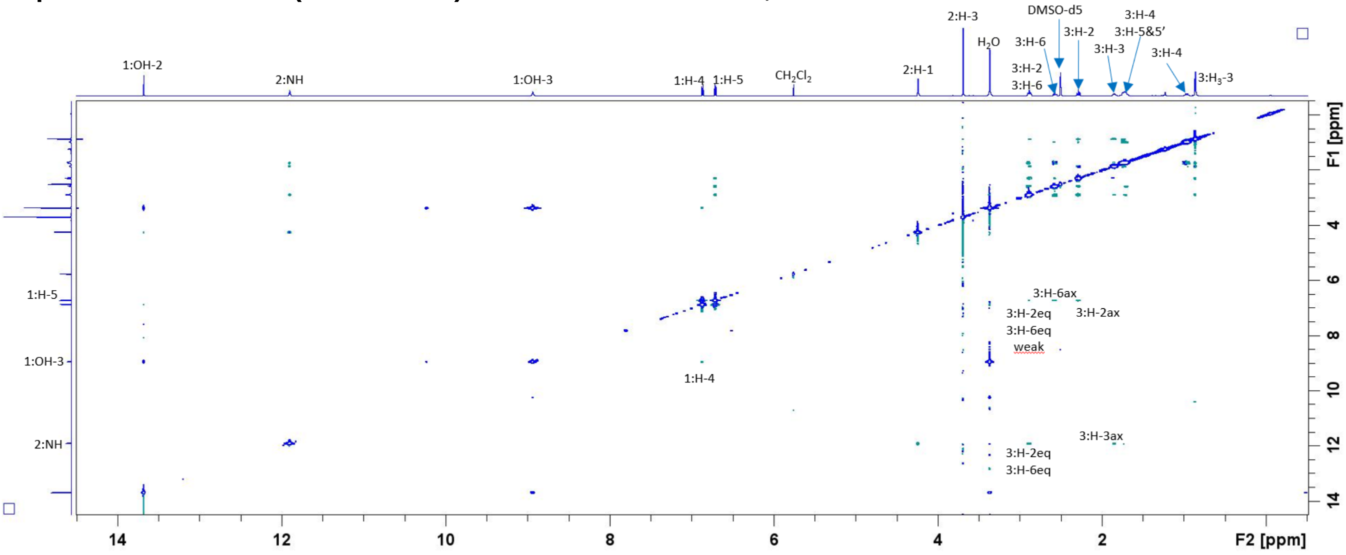

Comp 18f. ^{13}C NMR of 18f in DMSO-d_6, Bruker-600

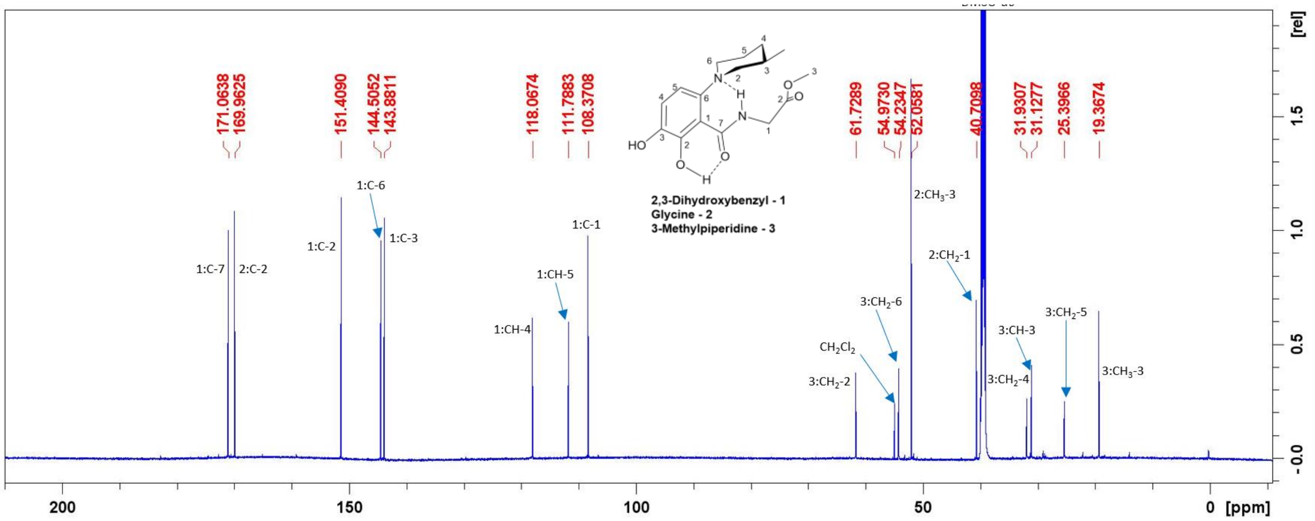

Comp 18f. ^{13}C DEPT-135 NMR of 18f in DMSO-d_6, Bruker-600

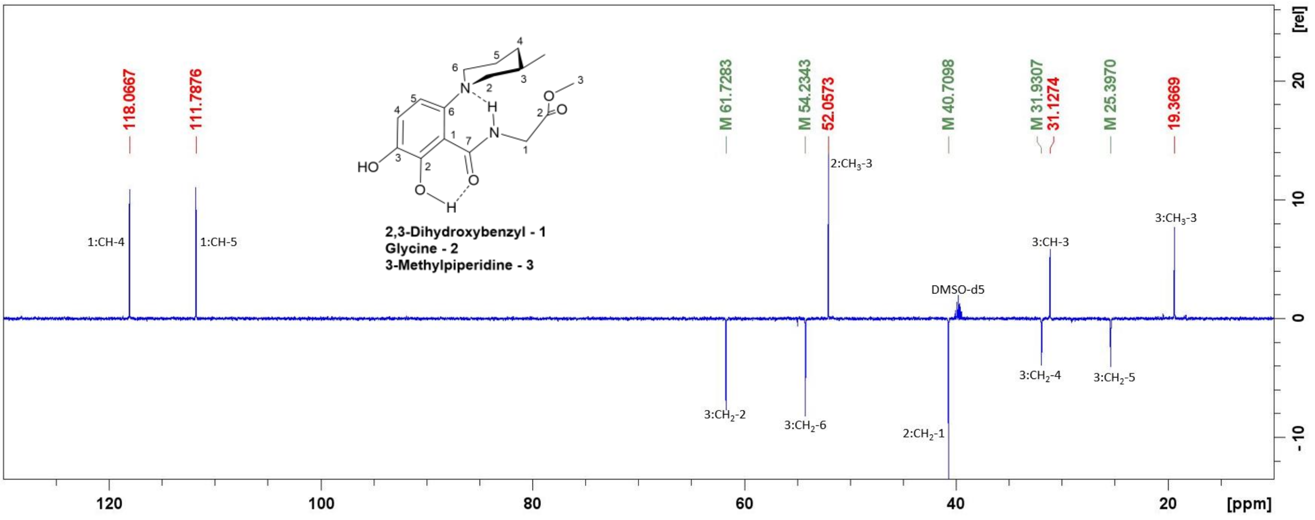

Comp 18f. 2D ^{1}H-^{13}C-HSQC-DEPT of 18f in DMSO-d_6, Bruker-600

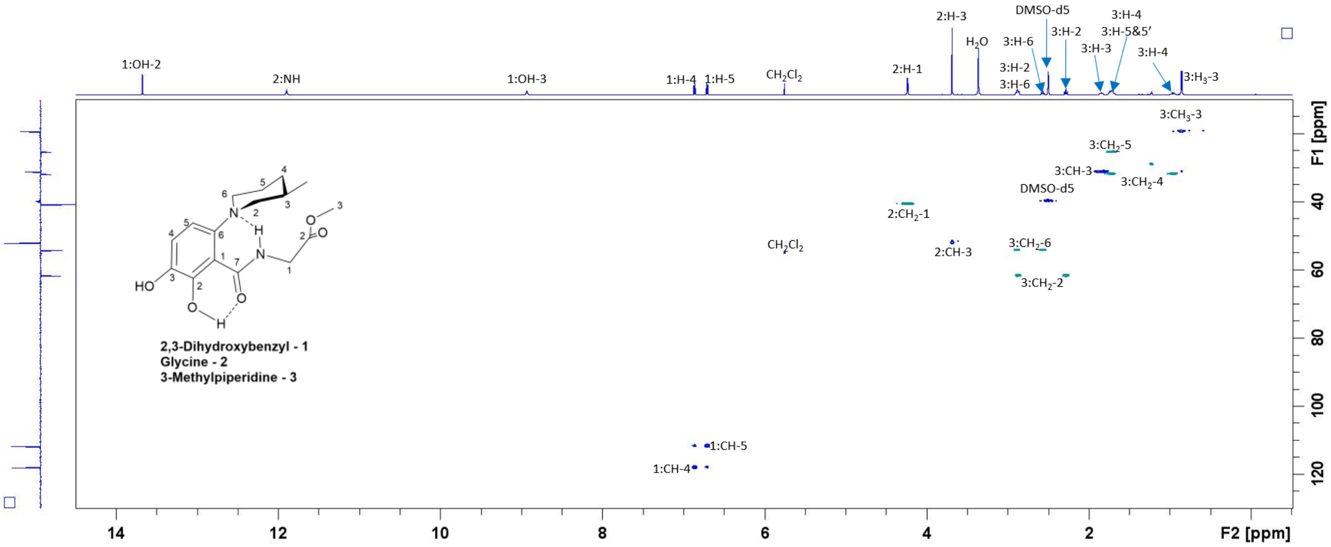

Comp 18f. 2D ^{1}H-^{13}C-HSQC-TOCSY (Tm = 27 ms) of 18f in DMSO-d_6, Bruker-600

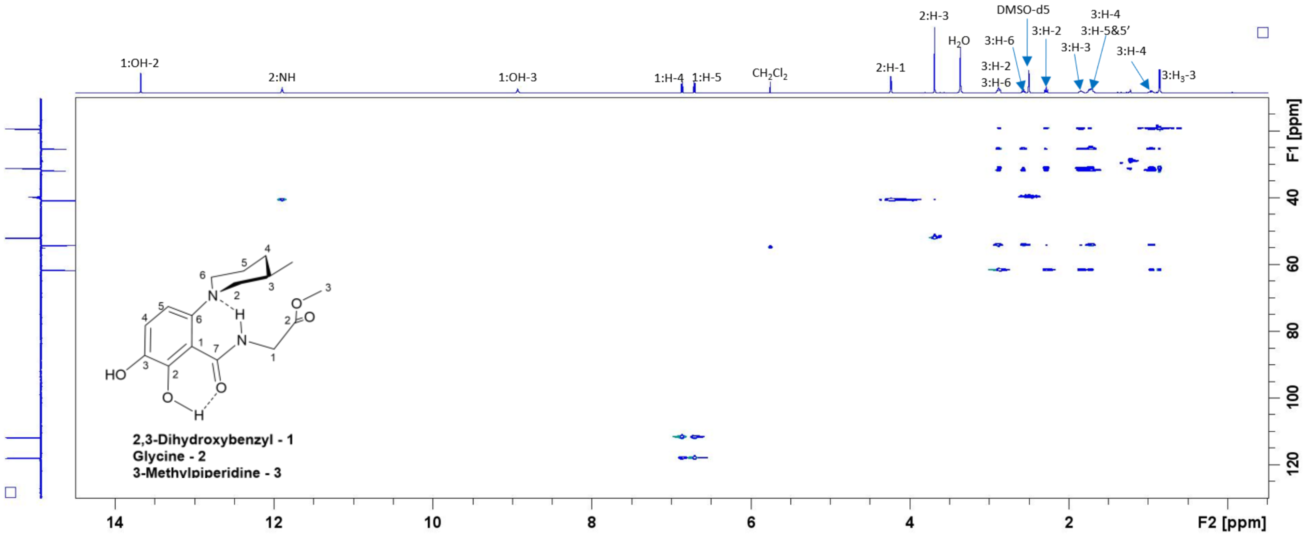

Comp 18f. 2D ^{1}H-^{15}N-HSQC NMR of 18f in DMSO-d_6, Bruker-600

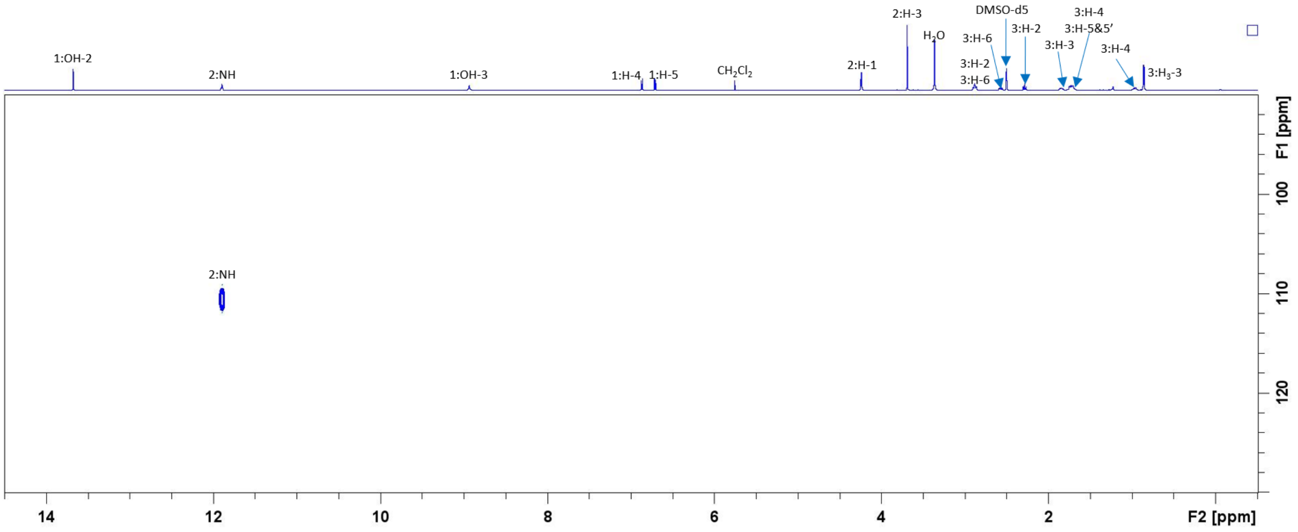

Comp 18f. 2D ^{1}H-^{13}C-HMBC (J_{app} = 10 Hz) NMR of 18f in DMSO-d_6, Bruker-600

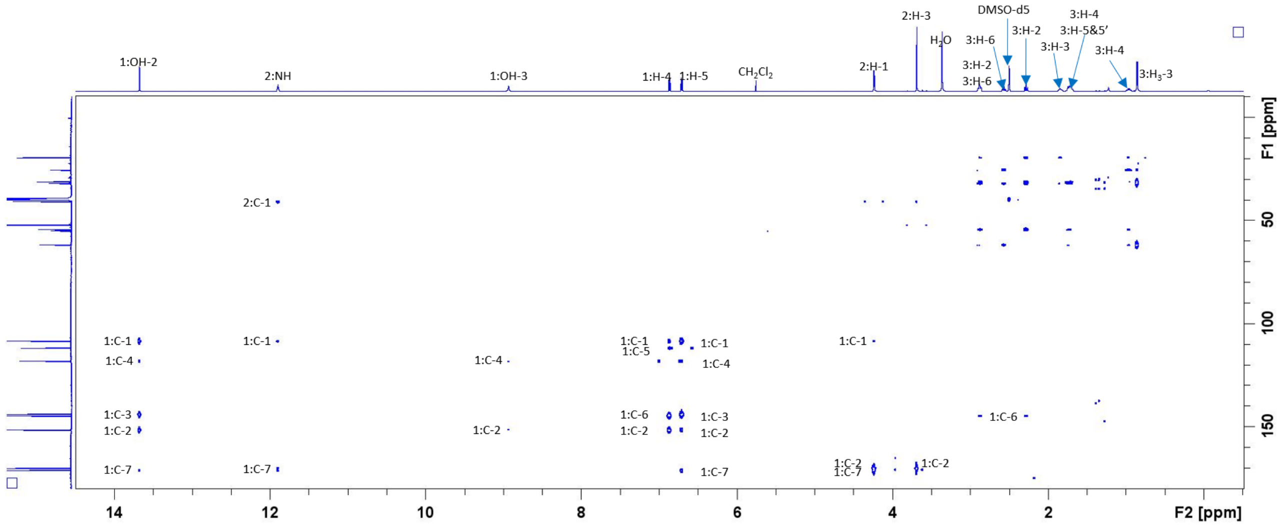

Comp 18f. 2D ^{1}H-^{15}N-HMBC (J_{app} = 8 Hz) NMR of 18f in DMSO-d_6, Bruker-600

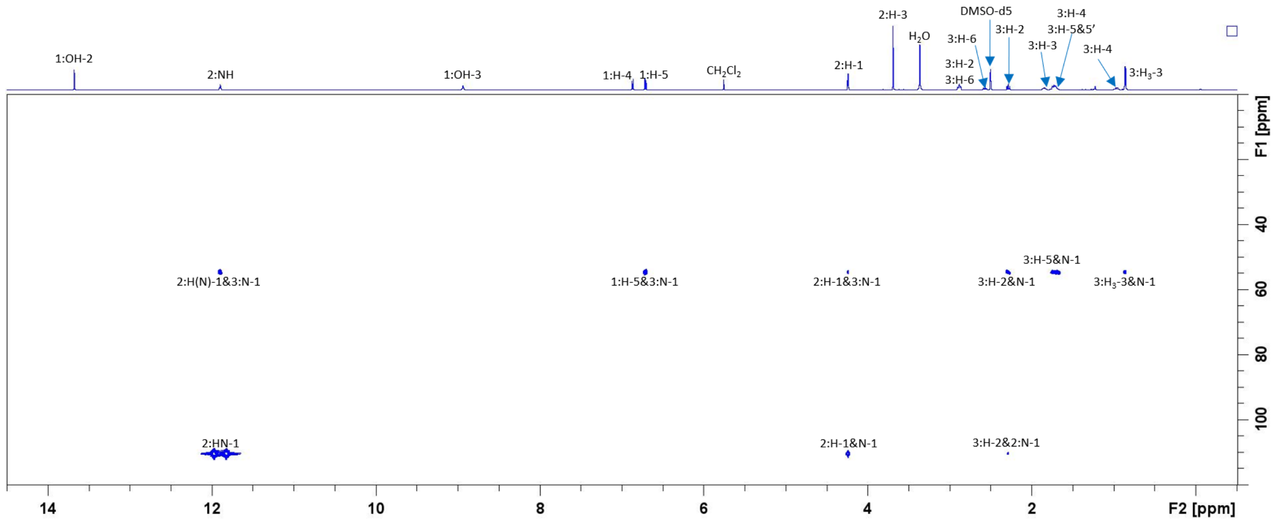

Comp 18f. HRMS (ES+) analysis of 18f

Single Mass Analysis
Tolerance = 80.0 PPM / DBE: min = -1.5, max = 50.0
Element prediction: Off
Number of isotope peaks used for i-FIT = 3

Monoisotopic Mass, Even Electron Ions
20 formula(e) evaluated with 3 results within limits (up to 5 closest results for each mass)
Elements Used:
C: 15-20 H: 10-30 N: 1-3 O: 1-6

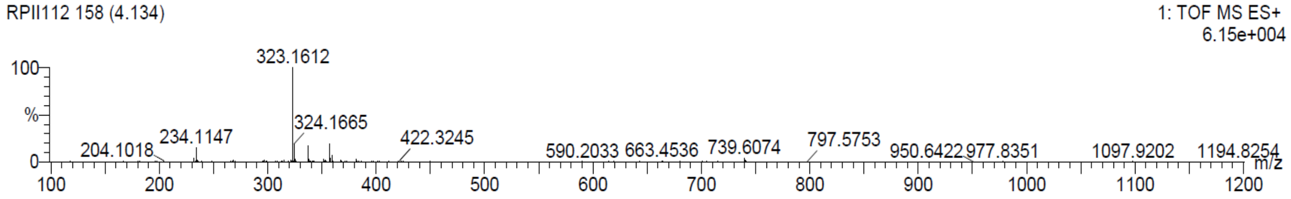

Minimum:				-1.5		
Maximum:		10.0	80.0	50.0		
Mass	Calc. Mass	mDa	PPM	DBE	i-FIT	Formula
323.1612	323.1607	0.5	1.5	6.5	3.6	C16 H23 N2 O5
	323.1760	-14.8	-45.8	10.5	689.8	C20 H23 N2 O2
	323.1396	21.6	66.8	11.5	1115.9	C19 H19 N2 O3

NMR and HRMS Compound 19, Bruker-300

Comp 19b. ^{1}H NMR of 19b in DMSO-d_6, Bruker-300

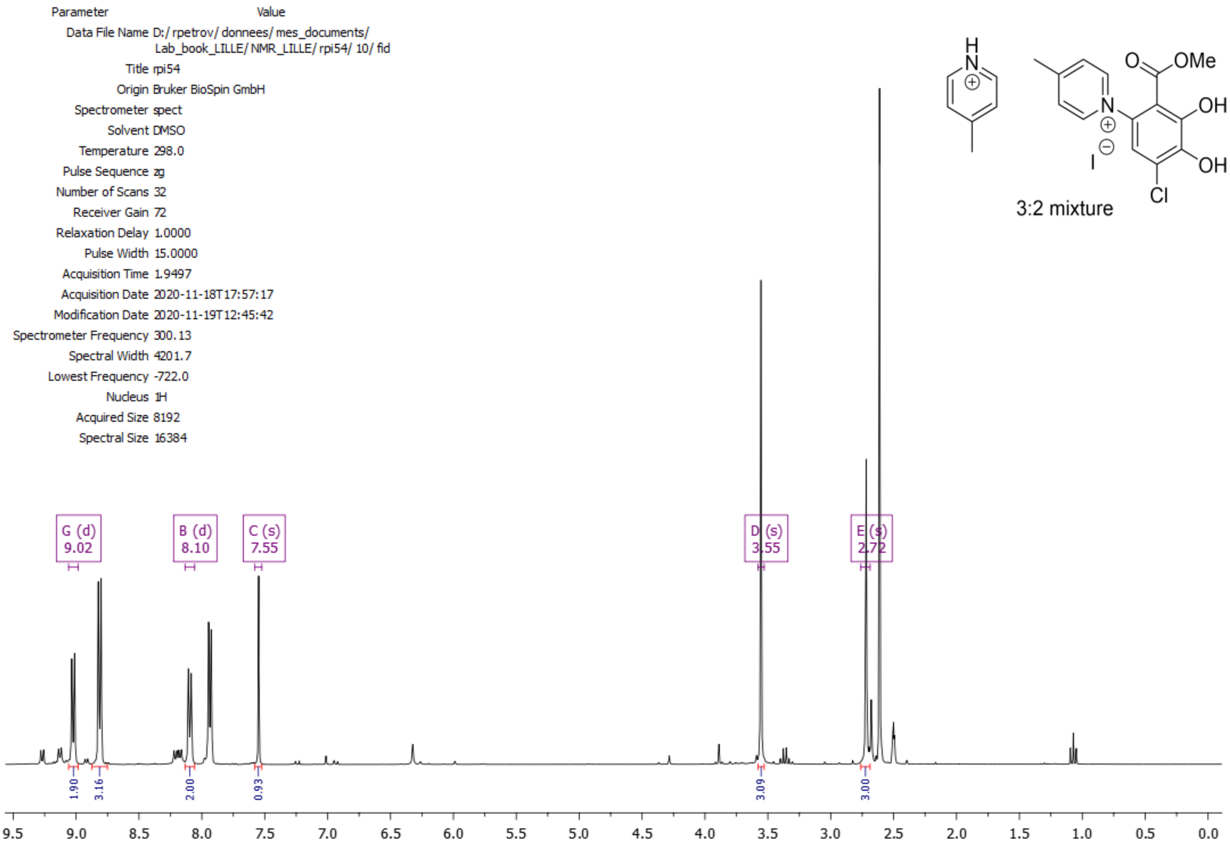

Comp 19b. HRMS (ES+) analysis of comp 19b

Single Mass Analysis
Tolerance = 10.0 mDa / DBE: min = -1.5, max = 50.0
Element prediction: Off
Number of isotope peaks used for i-FIT = 2

Monoisotopic Mass, Even Electron Ions
412 formula(e) evaluated with 22 results within limits (up to 50 closest results for each mass)
Elements Used:
C: 0-20 H: 0-20 N: 0-10 O: 0-10 Cl: 0-1
RPI58 62 (1.695)

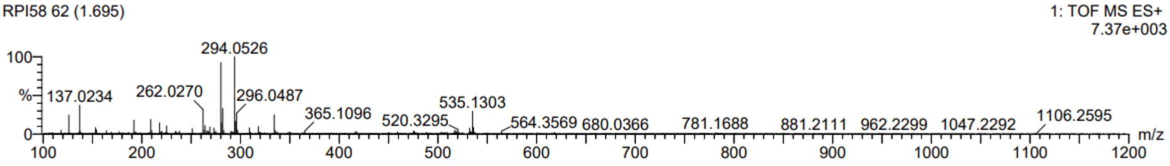

Minimum:				-1.5		
Maximum:		10.0	10.0	50.0		
Mass	Calc. Mass	mDa	PPM	DBE	i-FIT	Formula
294.0526	294.0533	-0.7	-2.4	8.5	0.2	C14 H13 N O4 Cl
	294.0488	3.8	12.9	14.5	5.0	C11 H4 N9 O2
	294.0515	1.1	3.7	13.5	5.8	C15 H8 N3 O4
	294.0546	-2.0	-6.8	13.5	11.1	C15 H9 N5 Cl
	294.0506	2.0	6.8	9.5	13.2	C10 H9 N7 O2 Cl

Comp 19c. ^{1}H NMR of 19c in DMSO-d_6, Bruker-300

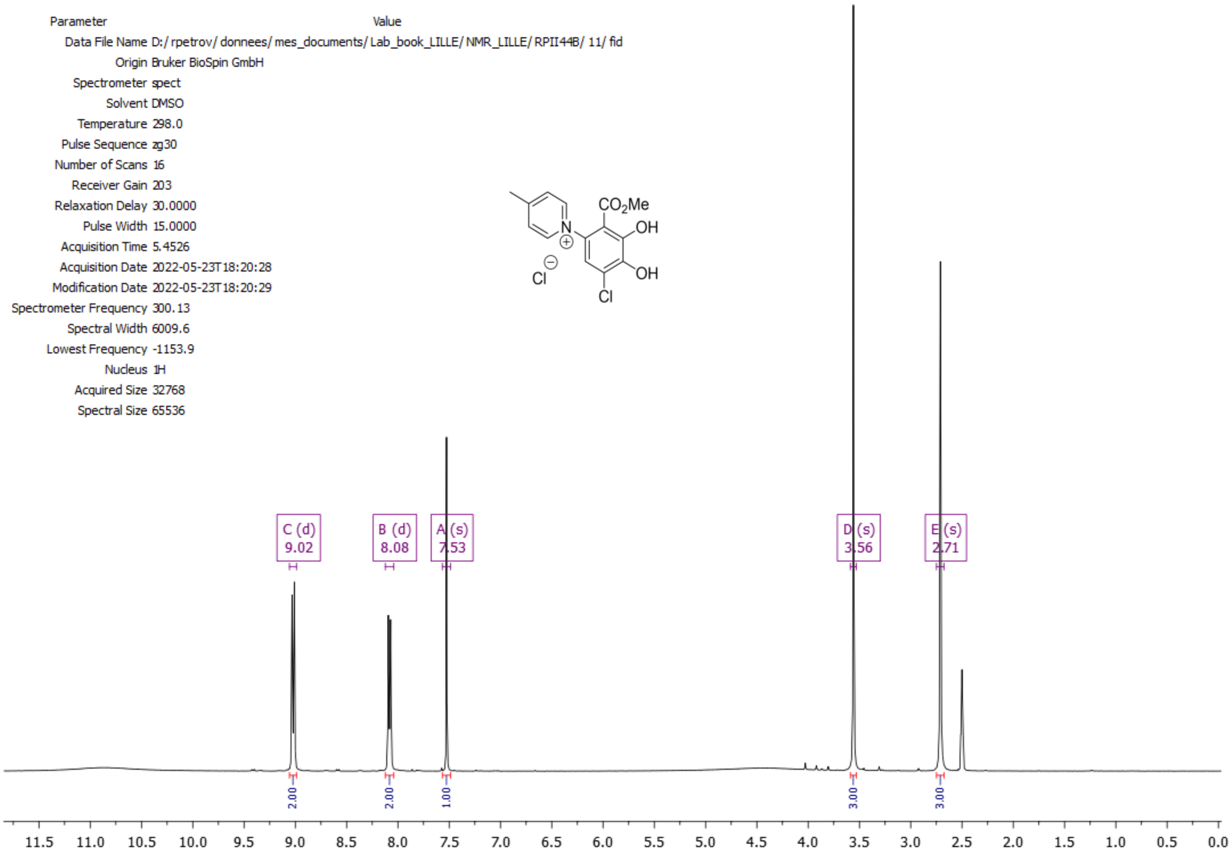

Comp 19c. ^{13}C NMR of 19c in DMSO-d_6, Bruker-300

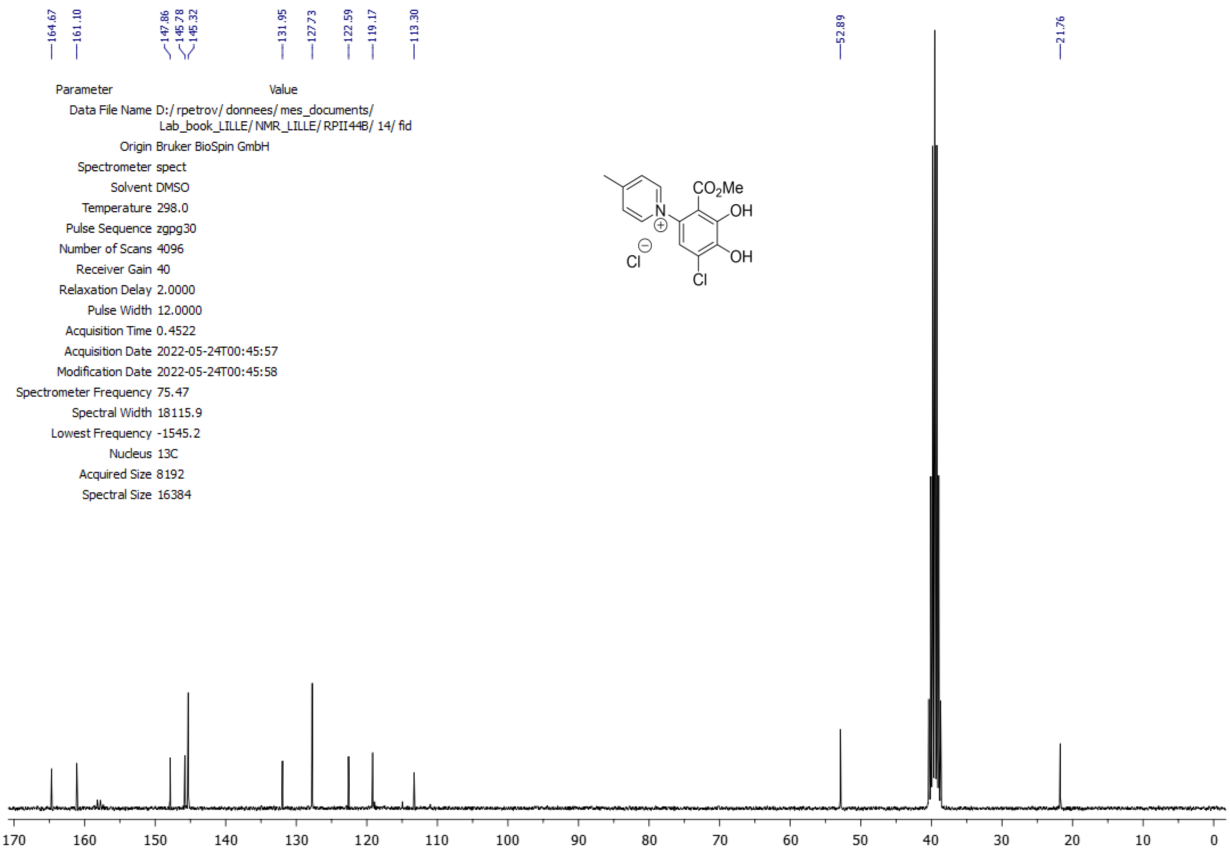

Comp 19c. HRMS (ES+) analysis

Single Mass Analysis
Tolerance = 5.0 mDa / DBE: min = -1.5, max = 50.0
Element prediction: Off
Number of isotope peaks used for i-FIT = 3

Monoisotopic Mass, Even Electron Ions
489 formula(e) evaluated with 11 results within limits (up to 50 best isotopic matches for each mass)
Elements Used:
C: 0-20 H: 0-25 N: 0-10 O: 0-10 Cl: 0-1

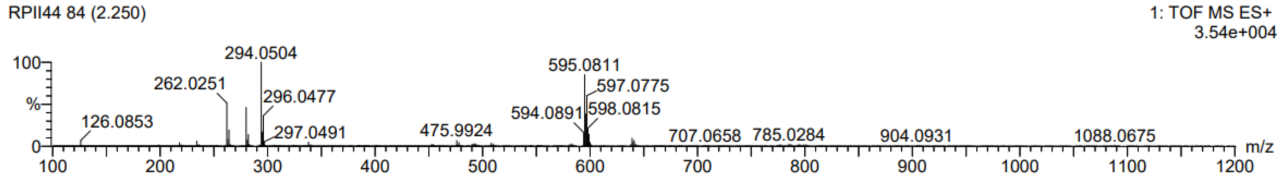

Minimum:				-1.5		
Maximum:		5.0	10.0	50.0		

Mass	Calc. Mass	mDa	PPM	DBE	i-FIT	Formula
294.0504	294.0533	-2.9	-9.9	8.5	30.7	C14 H13 N O4 Cl
	294.0546	-4.2	-14.3	13.5	88.1	C15 H9 N5 Cl
	294.0506	-0.2	-0.7	9.5	102.6	C10 H9 N7 O2 Cl
	294.0493	1.1	3.7	4.5	291.1	C9 H13 N3 O6 Cl
	294.0466	3.8	12.9	5.5	645.1	C5 H9 N9 O4 Cl

NMR and HRMS Compound 20, Bruker-300

Comp 20b. HRMS (ES+) analysis

Single Mass Analysis
Tolerance = 10.0 mDa / DBE: min = -1.5, max = 50.0
Element prediction: Off
Number of isotope peaks used for i-FIT = 2

Monoisotopic Mass, Even Electron Ions
412 formula(e) evaluated with 22 results within limits (up to 50 closest results for each mass)
Elements Used:
C: 0-20 H: 0-20 N: 0-10 O: 0-10 Cl: 0-1

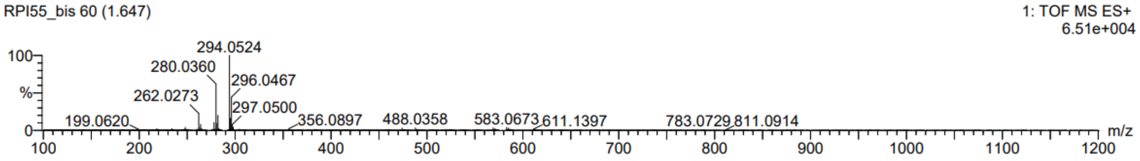

Mass	Calc. Mass	mDa	PPM	DBE	i-FIT	Formula
Minimum:				-1.5		
Maximum:		10.0	10.0	50.0		
294.0524	294.0533	-0.9	-3.1	8.5	2.1	C14 H13 N O4 Cl
	294.0488	3.6	12.2	14.5	41.2	C11 H4 N9 O2
	294.0515	0.9	3.1	13.5	64.7	C15 H8 N3 O4
	294.0506	1.8	6.1	9.5	100.8	C10 H9 N7 O2 Cl
	294.0546	-2.2	-7.5	13.5	116.1	C15 H9 N5 Cl

Comp 20c. ^{1}H NMR of 20c in DMSO-d_6, Bruker-300

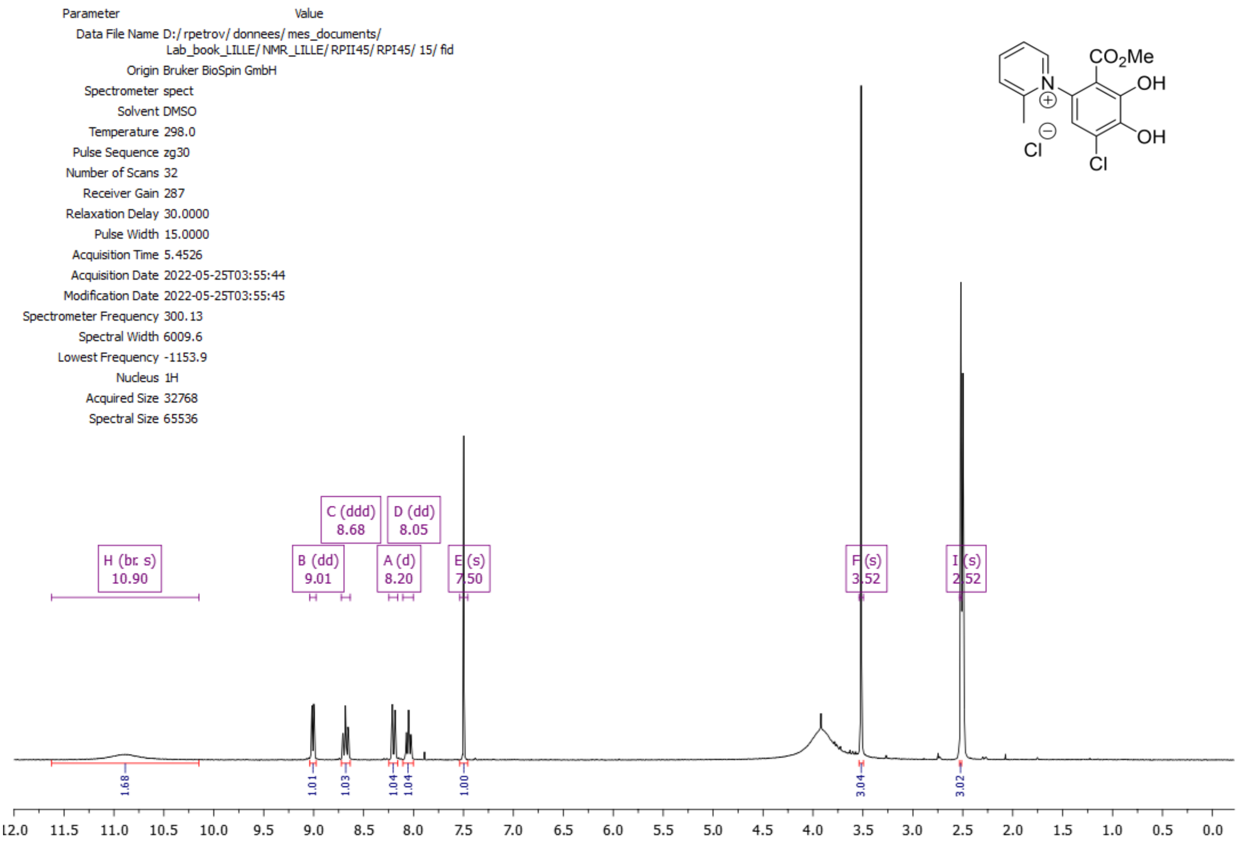

Comp 20c. ^{13}C NMR of 20c in DMSO-d_6, Bruker-300

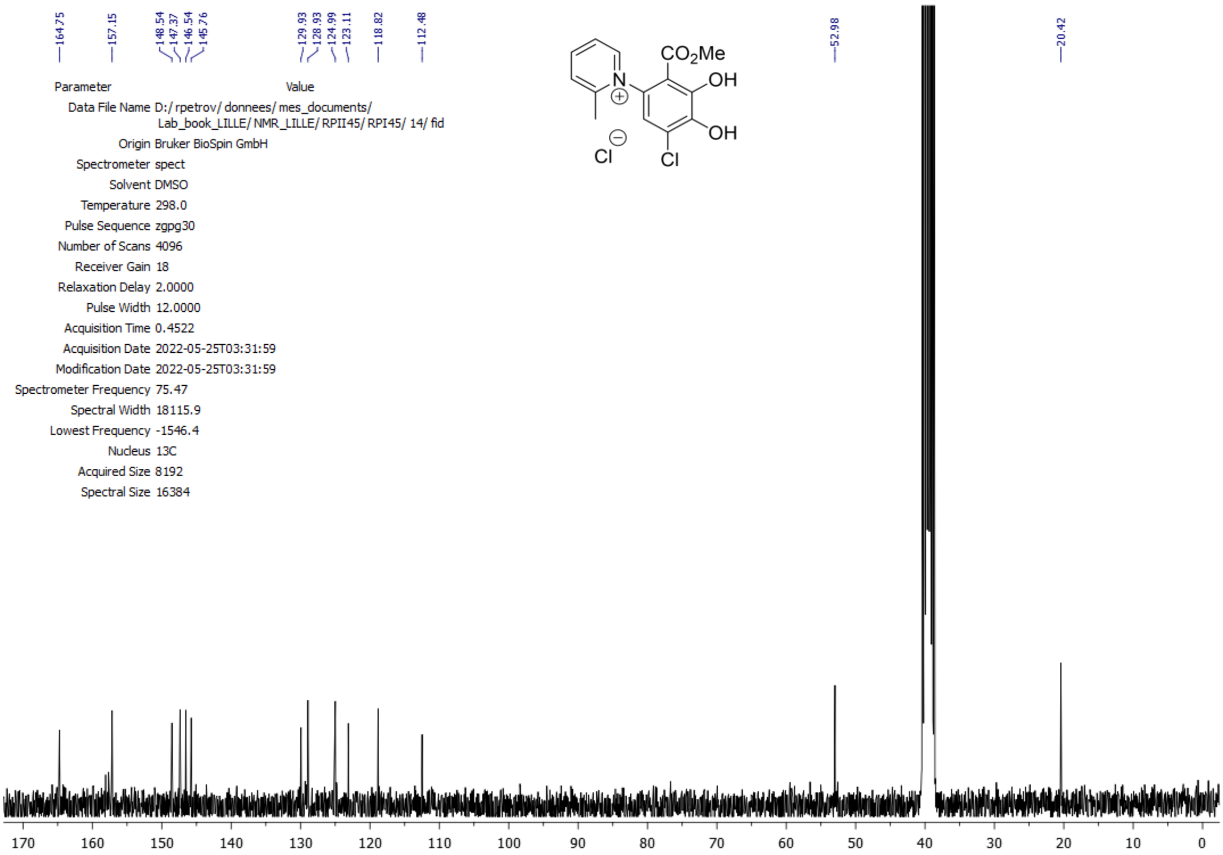

Comp 20c. HRMS (ES+) analysis

Single Mass Analysis
Tolerance = 5.0 mDa / DBE: min = -1.5, max = 50.0
Element prediction: Off
Number of isotope peaks used for i-FIT = 3

Monoisotopic Mass, Even Electron Ions
489 formula(e) evaluated with 11 results within limits (up to 50 best isotopic matches for each mass)
Elements Used:
C: 0-20 H: 0-25 N: 0-10 O: 0-10 Cl: 0-1

RPII45 74 (1.983) 1: TOF MS ES+ 6.71e+004

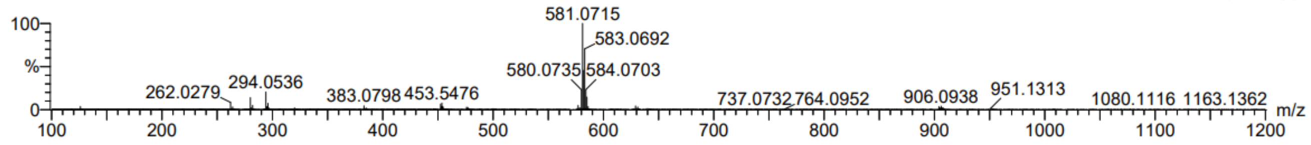

Minimum:				-1.5		
Maximum:		5.0	10.0	50.0		
Mass	Calc. Mass	mDa	PPM	DBE	i-FIT	Formula
294.0536	294.0533	0.3	1.0	8.5	4.6	C14 H13 N O4 Cl
	294.0546	-1.0	-3.4	13.5	16.5	C15 H9 N5 Cl
	294.0506	3.0	10.2	9.5	45.9	C10 H9 N7 O2 Cl
	294.0493	4.3	14.6	4.5	128.2	C9 H13 N3 O6 Cl
	294.0565	-2.9	-9.9	0.5	454.2	C3 H13 N7 O7 Cl

NMR and HRMS Compound 21, 3-pyridyl TAMRA Bruker-300

Comp 21. ^{1}H NMR of 21 in DMSO-d_6, Bruker-300

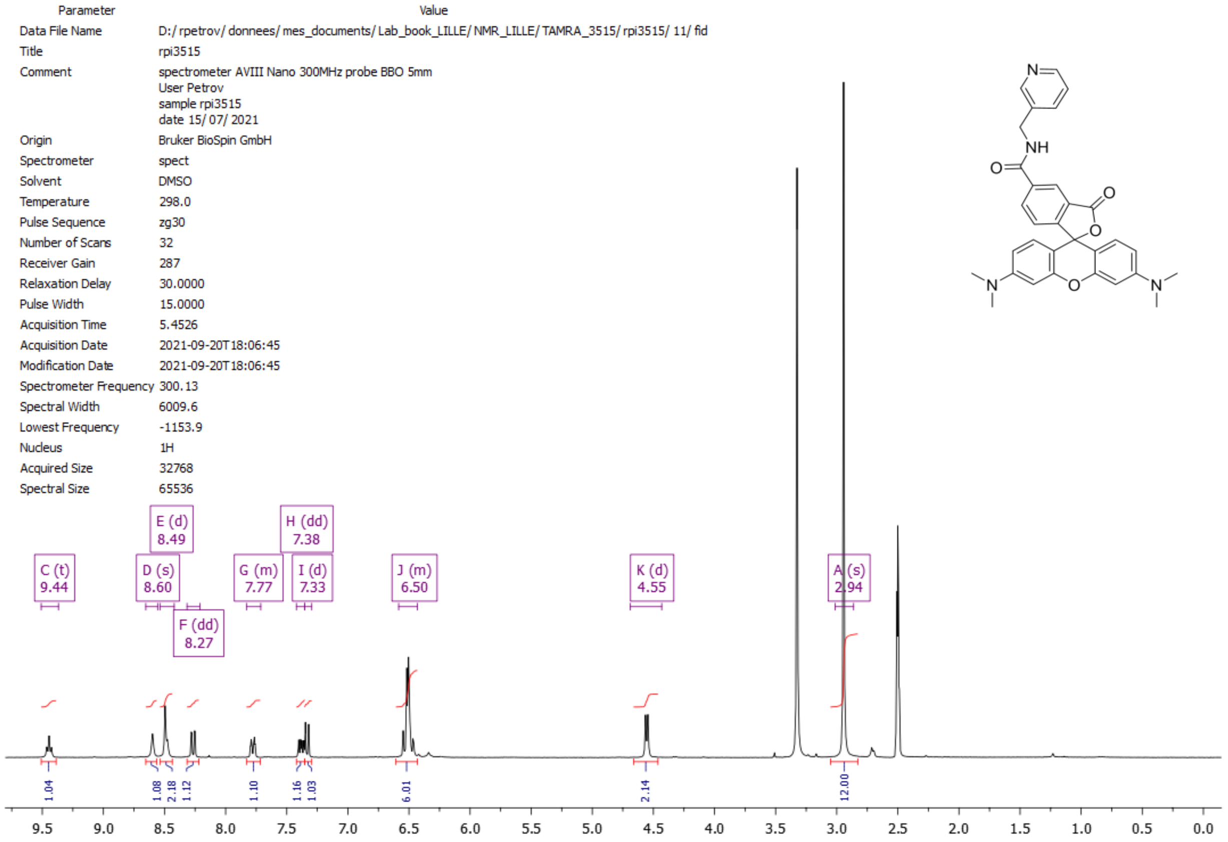

Comp 21. HRMS (ES+) analysis

Single Mass Analysis

Tolerance = 10.0 mDa / DBE: min = -1.5, max = 50.0

Element prediction: Off

Number of isotope peaks used for i-FIT = 3

Monoisotopic Mass, Even Electron Ions

515 formula(e) evaluated with 10 results within limits (up to 50 best isotopic matches for each mass)

Elements Used:

C: 0-50 H: 0-70 N: 0-5 O: 0-15

TAMRA3515 72 (1.934)

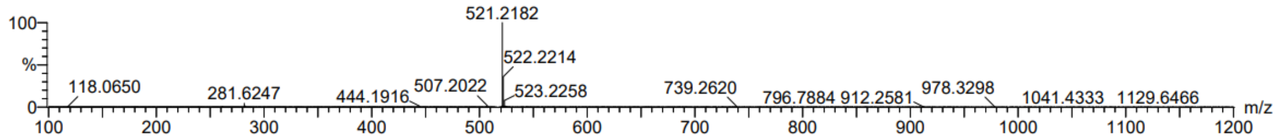

Mass	Calc. Mass	mDa	PPM	DBE	i-FIT	Formula
Minimum:				-1.5		
Maximum:		10.0	10.0	50.0		
521.2182	521.2189	-0.7	-1.3	19.5	18.7	C31 H29 N4 O4
	521.2175	0.7	1.3	14.5	35.9	C30 H33 O8
	521.2229	-4.7	-9.0	23.5	350.6	C36 H29 N2 O2
	521.2117	6.5	12.5	23.5	457.0	C37 H29 O3
	521.2135	4.7	9.0	10.5	596.6	C25 H33 N2 O10

NMR and HRMS Compound 22:gallium, Chlorodactyloferrin-TAMRA gallium complex Bruker-600

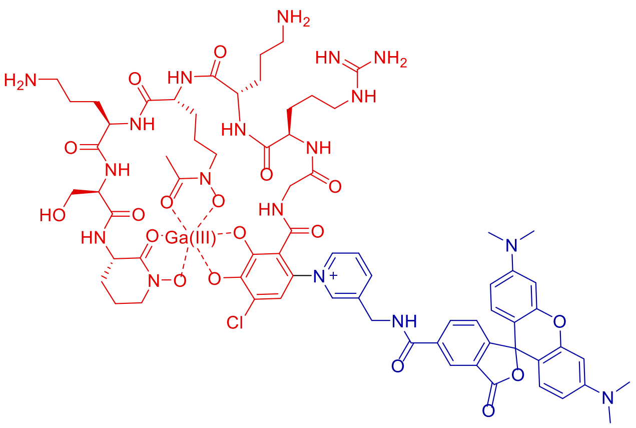

A) Comp 22:galium: 1D-¹H. NMR spectrum in CD_3OD at 14 T and 293 K.

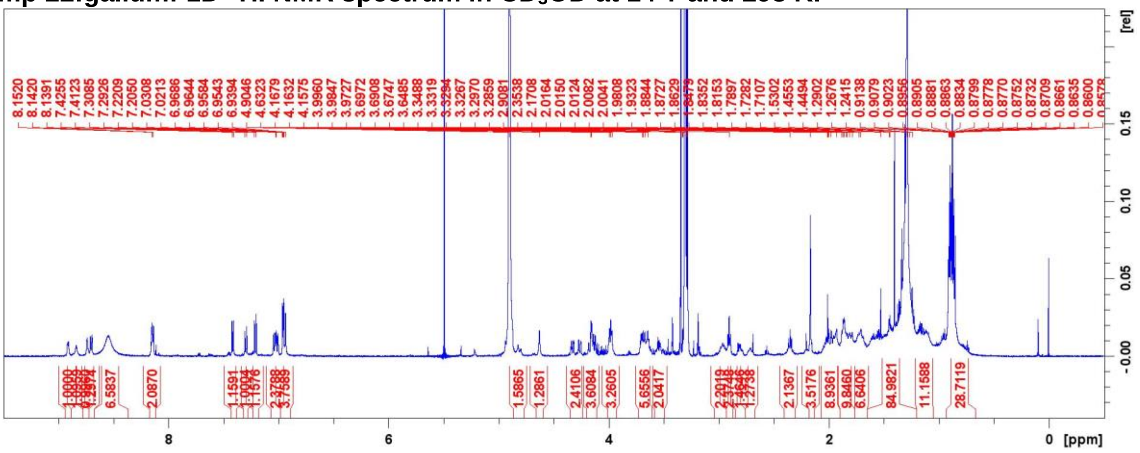

B) Comp 22:galium:2D-¹H-ROESY (*T*m = 400 ms). NMR spectrum in CD_3OD at 14 T and 293 K.

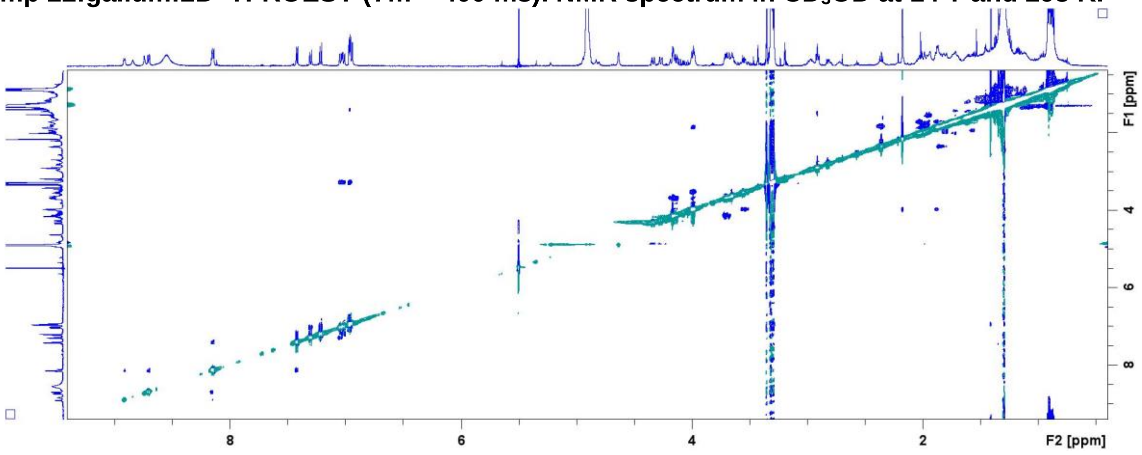

C) Comp 22:galium:2D-^{1}H-^{13}Caromatic-HSQC. NMR spectrum in CD_3OD at 14 T and 293 K.

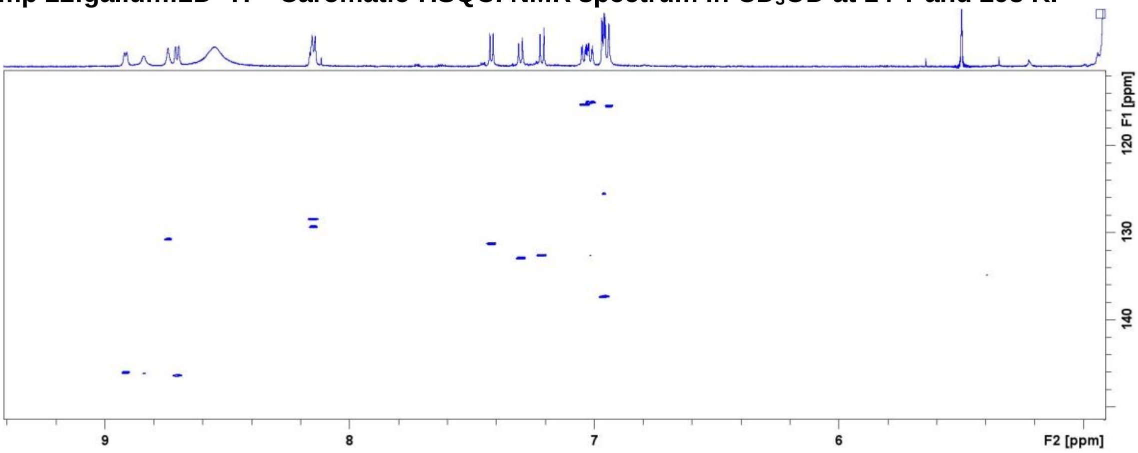

D) Comp 22:galium:2D-^{1}H-^{13}C-HSQC-TOCSY (*T*m = 59 ms). NMR spectrum in CD_3OD at 14 T and 293 K.

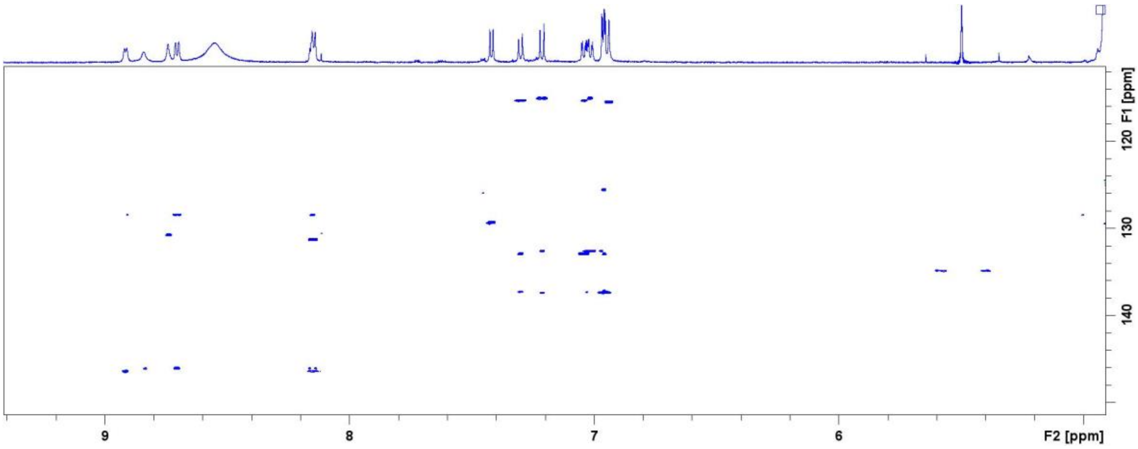

E) Comp 22:galium:2D-^{1}H-^{13}C-HMBC (*J*app = 10 Hz). NMR spectrum in CD_3OD at 14 T and 293 K.

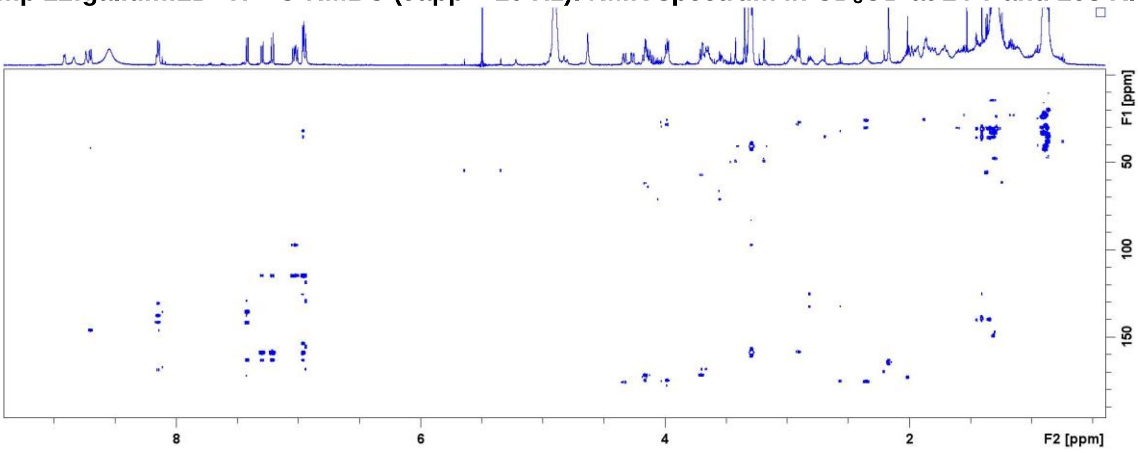

F) Comp 22:galium: 2D-^{1}H-^{15}N-HMBC (*J*app = 8 Hz). NMR spectrum in CD_3OD at 14 T and 293 K.

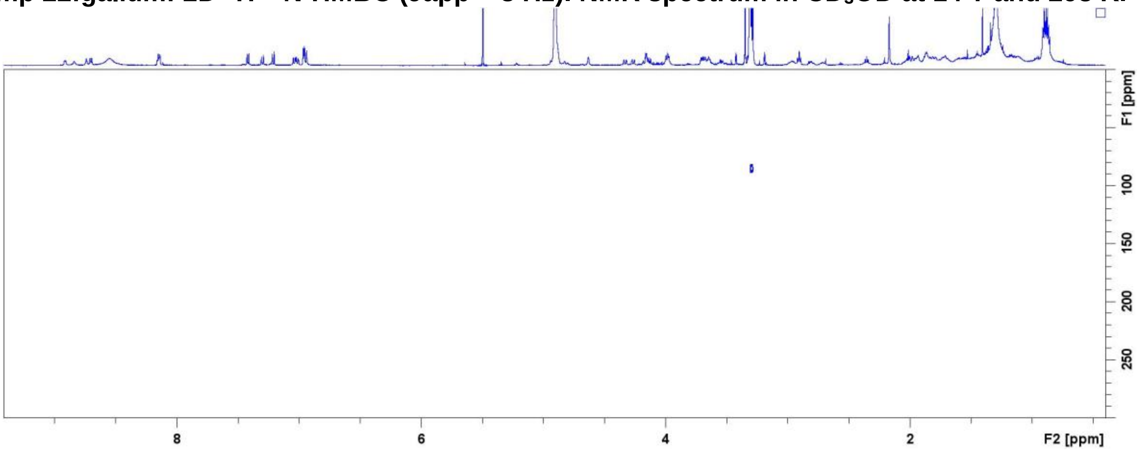

G) Comp 22:iron: HRMS (ES+) analysis

Single Mass Analysis
Tolerance = 10.0 mDa / DBE: min = -1.5, max = 50.0
Element prediction: Off
Number of isotope peaks used for i-FIT = 2

Monoisotopic Mass, Odd and Even Electron Ions
1102 formula(e) evaluated with 16 results within limits (up to 50 closest results for each mass)
Elements Used:
C: 70-80 H: 0-90 N: 0-20 O: 0-20 Cl: 1-1 Fe: 0-1
ClDafTAMRA3515_bis 68 (1.838)

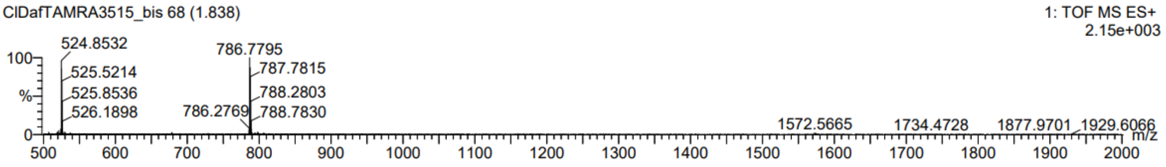

Minimum:				-1.5		
Maximum:		10.0	10.0	50.0		

Mass	Calc. Mass	mDa	PPM	DBE	i-FIT	Formula
1572.5665	1572.5640	2.5	1.6	36.0	0.4	C71 H89 N18 O18 Cl Fe
	1572.5752	-8.7	-5.5	36.0	0.4	C70 H89 N20 O17 Cl Fe
	1572.5614	5.1	3.2	44.0	0.6	C75 H81 N18 O19 Cl
	1572.5726	-6.1	-3.9	44.0	0.6	C74 H81 N20 O18 Cl
	1572.5667	-0.2	-0.1	40.5	0.6	C74 H87 N19 O15 Cl Fe

NMR and HRMS Compound 23: 3-pyridyl penicillin Bruker-300

Comp 23-1: intermediate 1 ^{1}H NMR of 23-1 in DMSO-d_6, Bruker-300

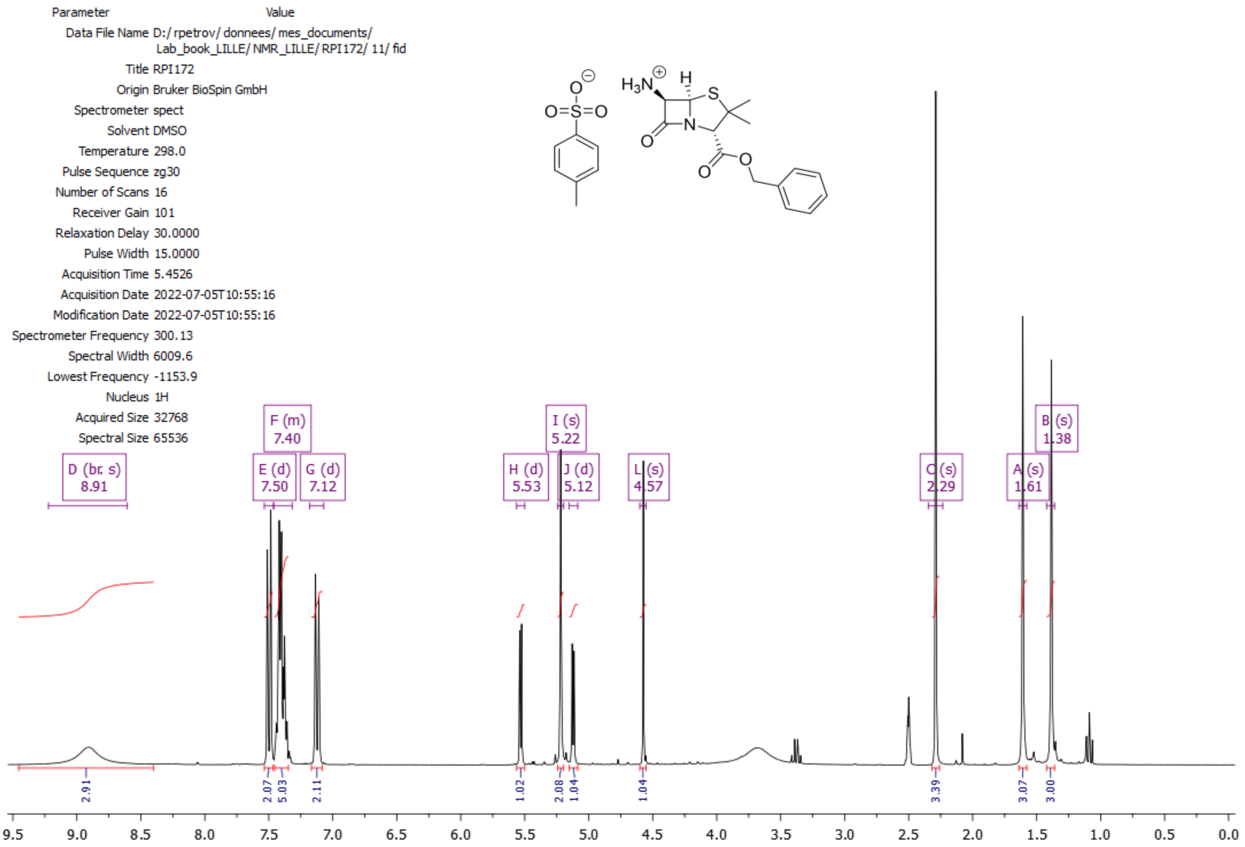

Comp 23-1: intermediate 1 ^{13}C NMR of 23-1 in DMSO-d_6, Bruker-300

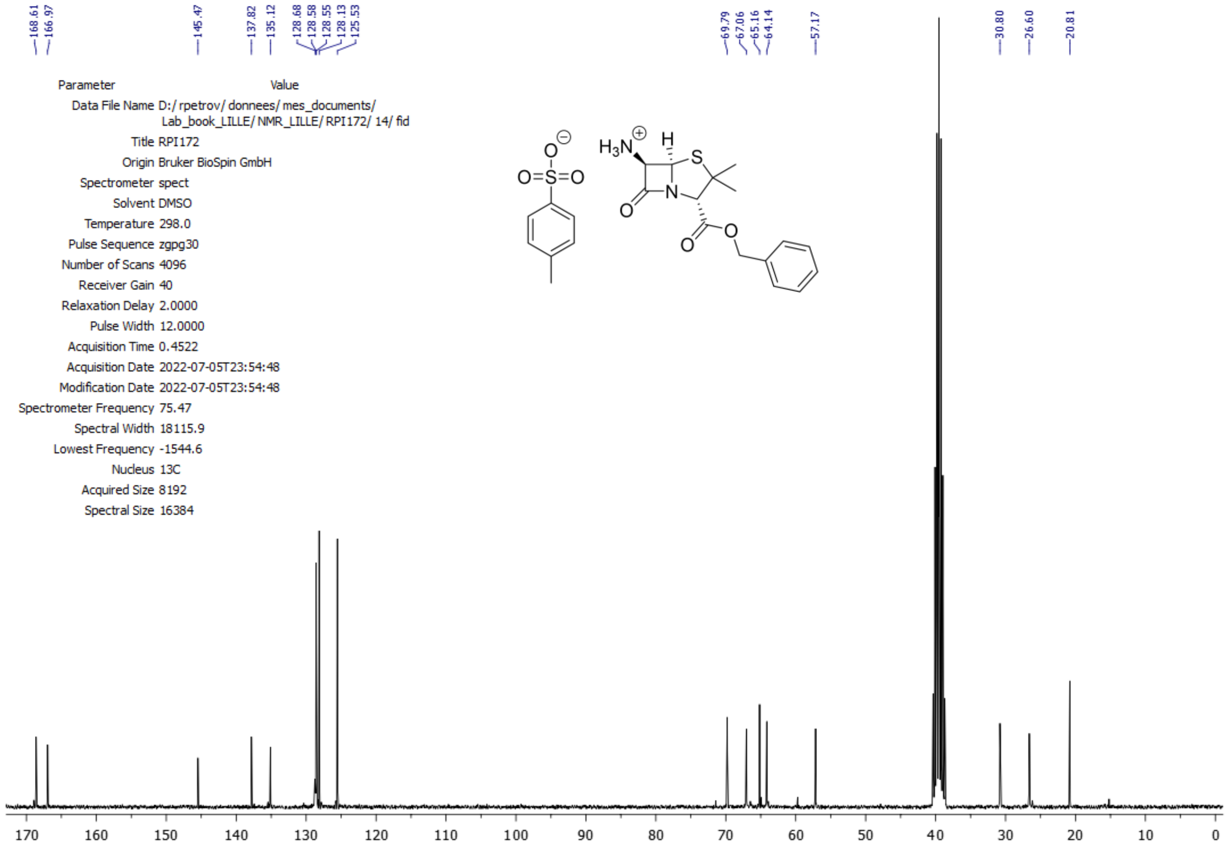

Comp 23-2 intermediate 2: ^{1}H NMR of 23-2 in $CDCl_3$, Bruker-300

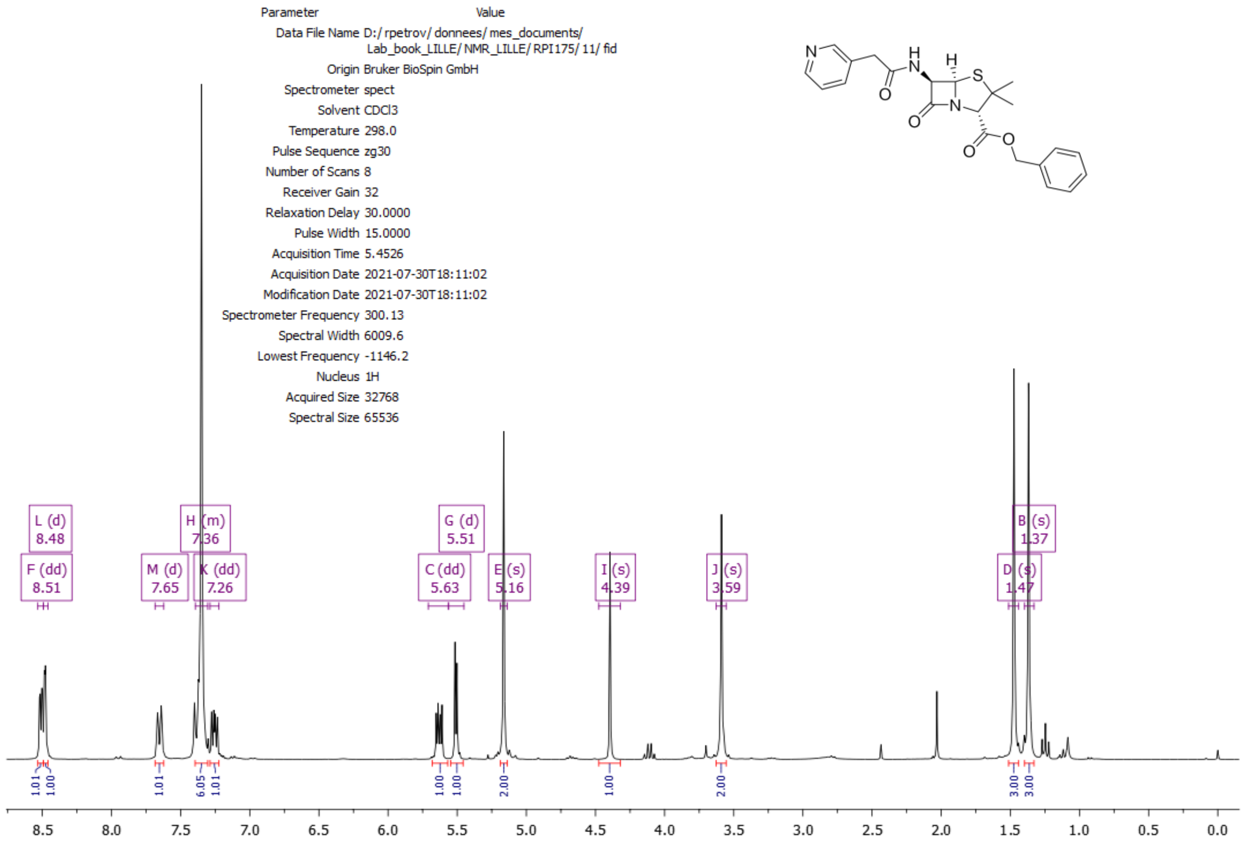

Comp 23-2 intermediate 2: ^{13}C NMR of 23-2 in $CDCl_3$, Bruker-300

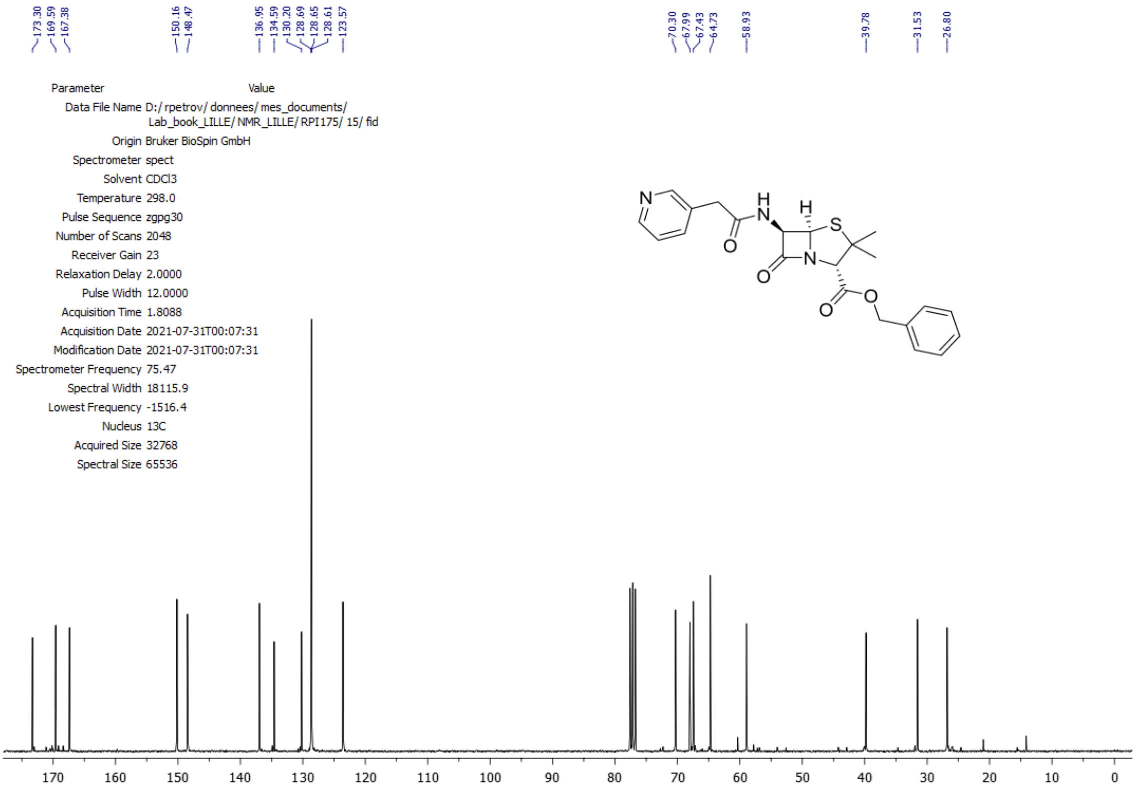

Comp 23-2 intermediate 2: HRMS (ES+) analysis

Single Mass Analysis
Tolerance = 5.0 mDa / DBE: min = -1.5, max = 50.0
Element prediction: Off
Number of isotope peaks used for i-FIT = 3

Monoisotopic Mass, Even Electron Ions
428 formula(e) evaluated with 10 results within limits (up to 50 best isotopic matches for each mass)
Elements Used:
C: 0-25 H: 0-25 N: 0-10 O: 0-10 S: 0-1

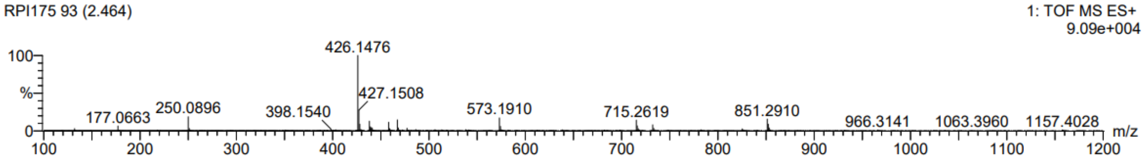

Minimum:				-1.5		
Maximum:		5.0	10.0	50.0		
Mass	Calc. Mass	mDa	PPM	DBE	i-FIT	Formula
426.1476	426.1488	-1.2	-2.8	12.5	6.0	C22 H24 N3 O4 S
	426.1501	-2.5	-5.9	17.5	66.4	C23 H20 N7 S
	426.1461	1.5	3.5	13.5	157.3	C18 H20 N9 O2 S
	426.1447	2.9	6.8	8.5	411.3	C17 H24 N5 O6 S
	426.1454	2.2	5.2	17.5	848.0	C25 H20 N3 O4

Comp 23. ^{1}H NMR of 26 in DMSO-d_6, Bruker-300

Parameter	Value
Comment	spectrometer AVIII Nano 300MHz probe BBO 5mm<br>sample RPI176P<br>date 15/ 07/ 2021<br>te 298<br>RPI176 std proton in DMSO-d6<br>SN_1D1H-quantitatif DMSO / opt/ DATA/ routine 7<br>NS=16, DS=4,<br>RG=287, D1=30.00s, O1=1853.30Hz, O2P=6.175ppm, O3P=6.175ppm<br>experiment time = 11min 49sec
Origin	Bruker BioSpin GmbH
Spectrometer	spect
Solvent	DMSO
Temperature	298.0
Pulse Sequence	zg30
Number of Scans	16
Receiver Gain	287
Relaxation Delay	30.0000
Pulse Width	15.0000
Acquisition Time	5.4526
Acquisition Date	2021-09-02T13:00:39
Modification Date	2021-09-02T13:00:39
Spectrometer Frequency	300.13
Spectral Width	6009.6
Lowest Frequency	-1153.9
Nucleus	1H
Acquired Size	32768
Spectral Size	65536

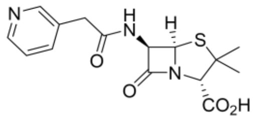

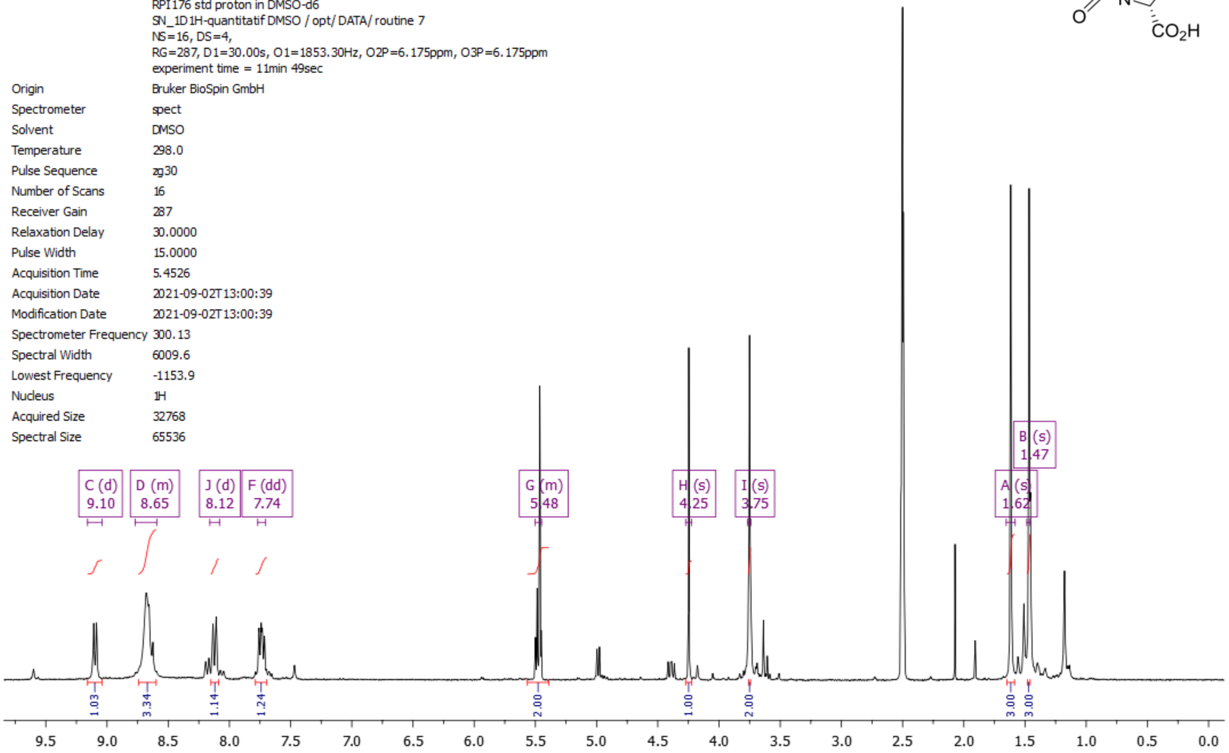

NMR and HRMS Compound 24:gallium, Chlorodactyloferrin-penicillin gallium complex Bruker-600

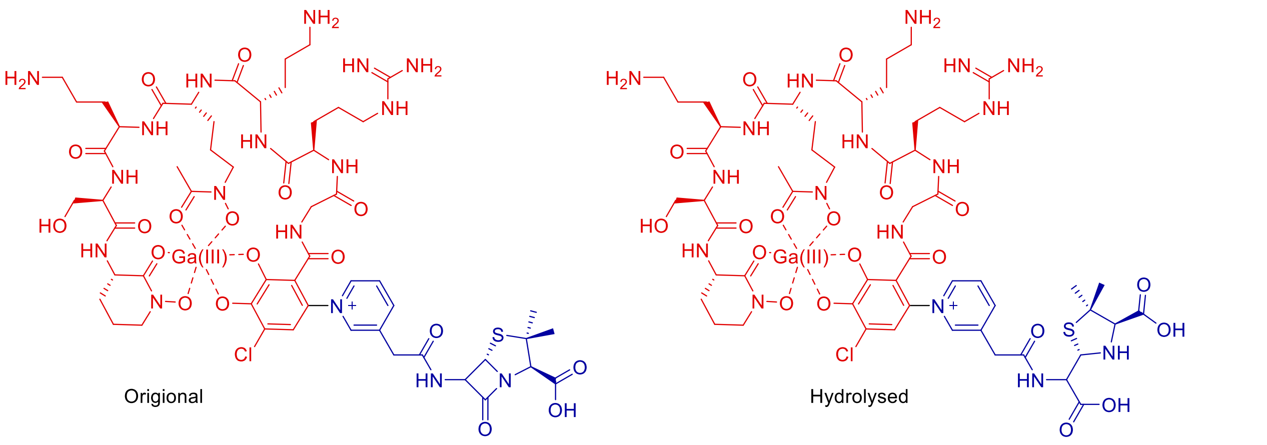

A) Comp. 24:gallium: 1D-^{1}H Final. NMR spectrum in CD_3OD at 14 T and 293 K.

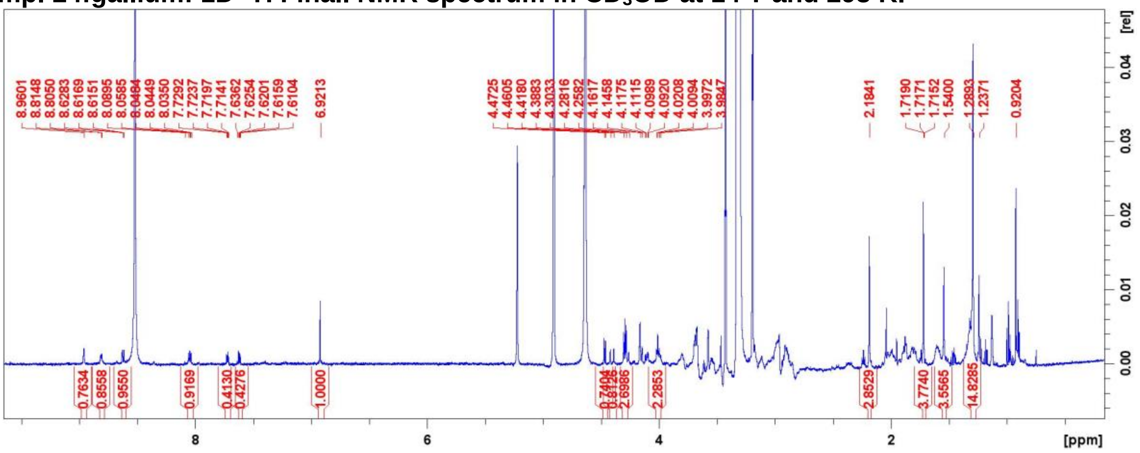

B) Comp. 24:gallium: 1D-^{1}H at t_0, t_0+3,25hours, t_0+1day21hours, t_0+6days+20hours (from bottom to top). NMR spectra in CD_3OD at 14 T and 293 K.

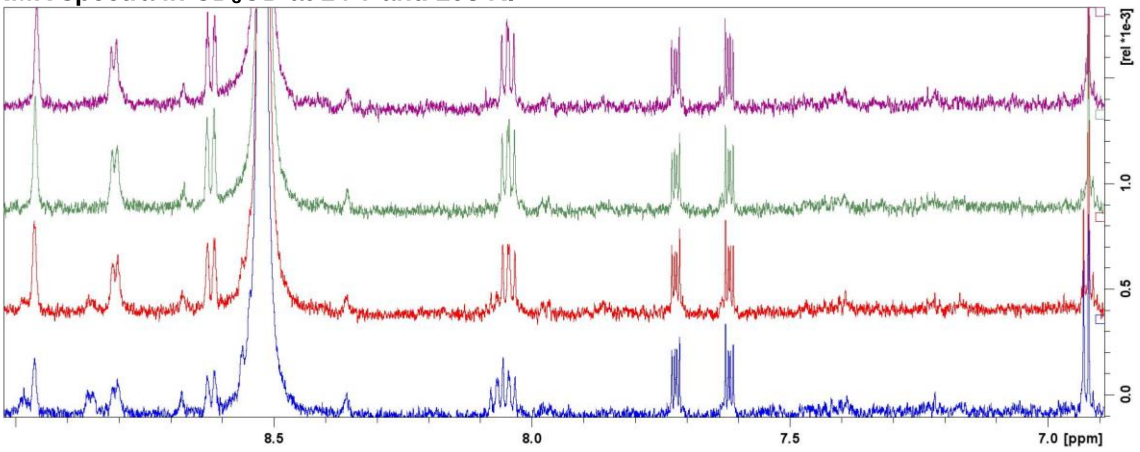

Comp. 24::iron. HRMS (ES+) analysis

Single Mass Analysis
Tolerance = 5.0 mDa / DBE: min = -1.5, max = 50.0
Element prediction: Off
Number of isotope peaks used for i-FIT = 3

Monoisotopic Mass, Odd and Even Electron Ions
182 formula(e) evaluated with 4 results within limits (up to 50 best isotopic matches for each mass)
Elements Used:
C: 0-58 H: 0-80 N: 15-18 O: 15-20 S: 0-1 Cl: 0-1 Fe: 0-1

ClDafBlac 53 (1.451)

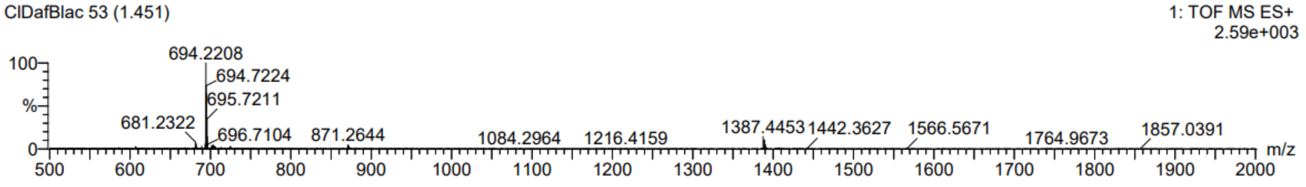

Minimum:				-1.5		
Maximum:		5.0	10.0	50.0		
Mass	Calc. Mass	mDa	PPM	DBE	i-FIT	Formula
1387.4453	1387.4469	-1.6	-1.2	25.0	1.5	C55 H78 N17 O18 S Cl Fe
	1387.4496	-4.3	-3.1	29.5	1.7	C58 H76 N18 O15 S Cl Fe
	1387.4436	1.7	1.2	30.0	3.1	C58 H74 N17 O18 Cl Fe
	1387.4465	-1.2	-0.9	29.5	32.9	C58 H75 N16 O19 S Fe

NMR and HRMS Compound 25: 3-pyridyl rifampicin Bruker-300

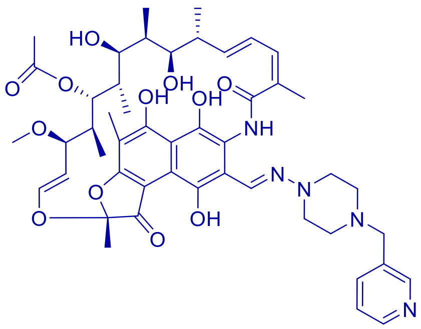

Comp 25. ^{1}H NMR spectrum in $CDCl_3$

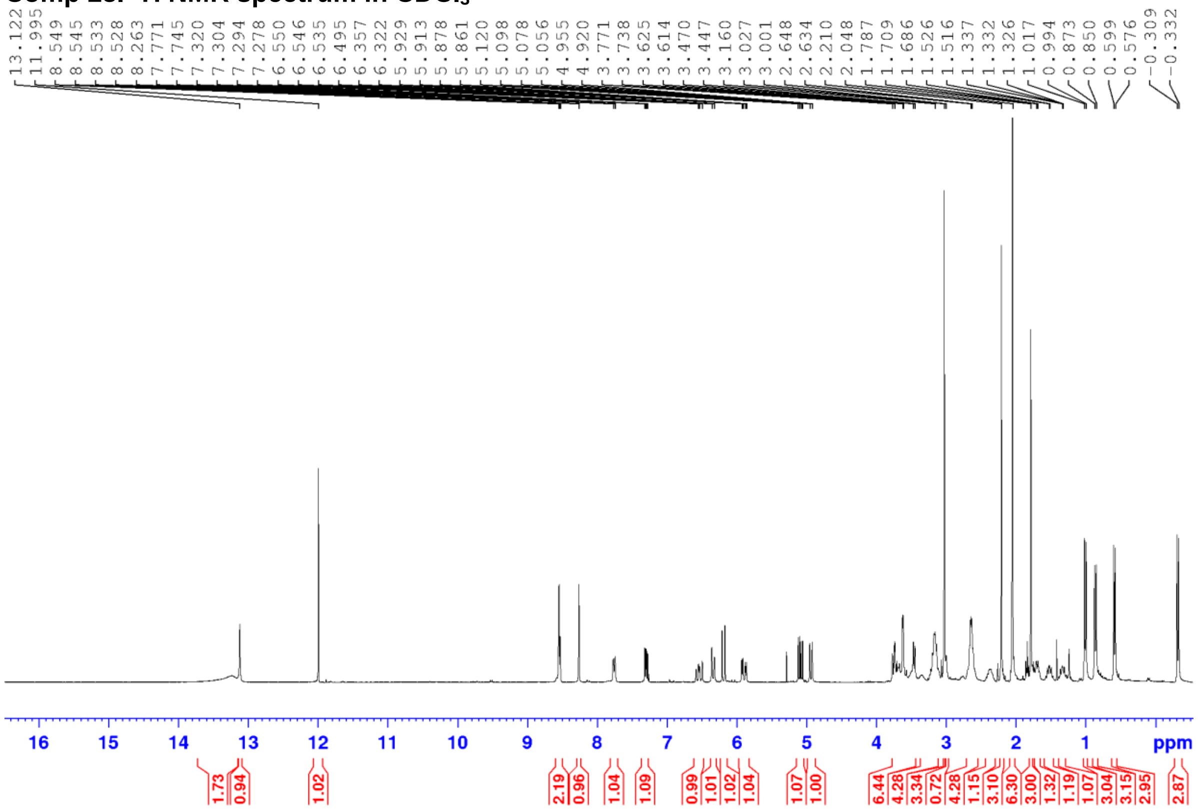

Comp 25. ^{13}C NMR spectrum

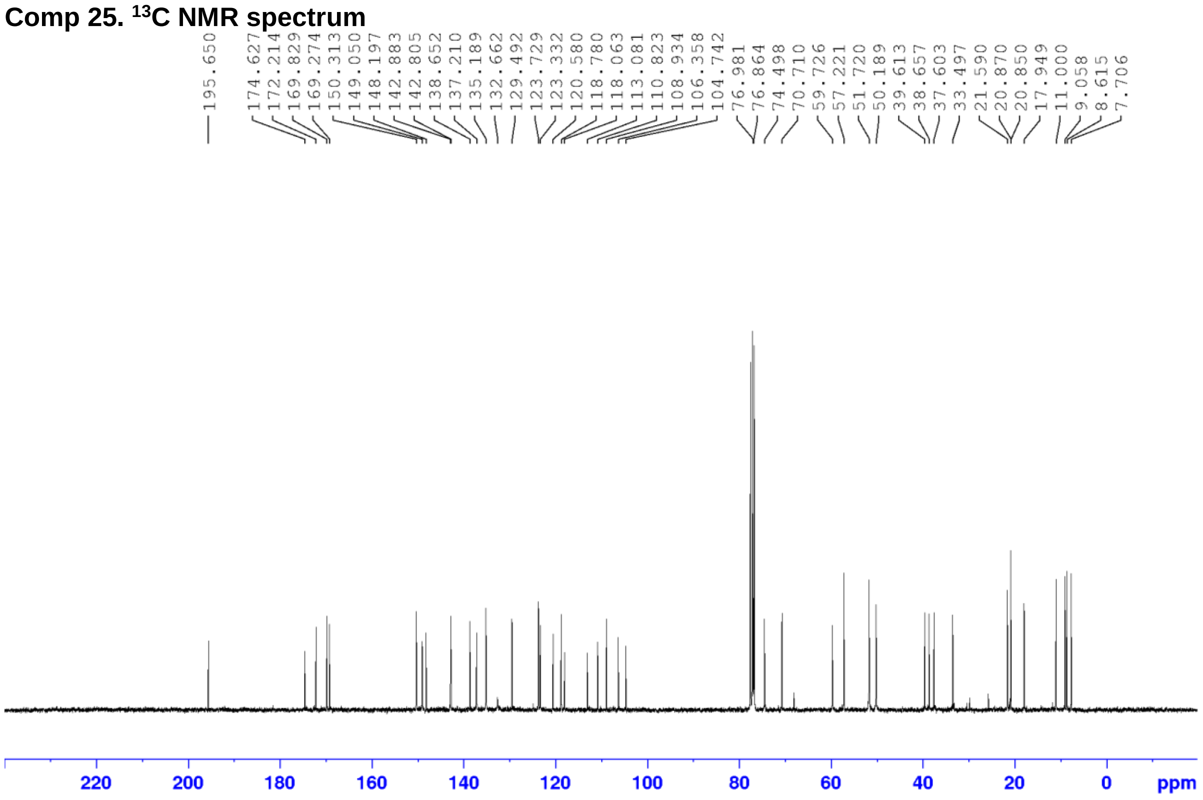

Comp 25. HRMS (ESI+) analysis

Elemental Composition Report **Page 1**

Single Mass Analysis
Tolerance = 10.0 PPM / DBE: min = -1.5, max = 50.0
Element prediction: Off
Number of isotope peaks used for i-FIT = 3

Monoisotopic Mass, Even Electron Ions
382 formula(e) evaluated with 4 results within limits (all results (up to 1000) for each mass)
Elements Used:
C: 1-50 H: 5-80 N: 0-10 O: 0-15

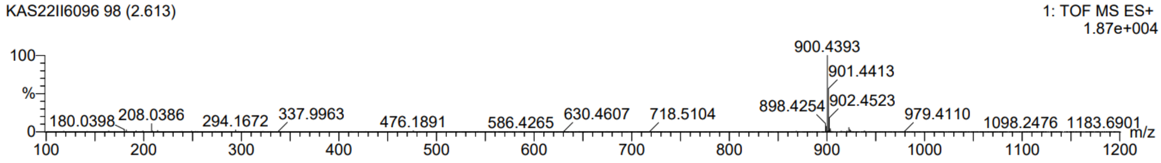

Mass	Calc. Mass	mDa	PPM	DBE	i-FIT	Formula
Minimum:				-1.5		
Maximum:		5.0	10.0	50.0		
900.4393	900.4395	-0.2	-0.2	20.5	0.7	C48 H62 N5 O12
	900.4408	-1.5	-1.7	25.5	8.7	C49 H58 N9 O8
	900.4355	3.8	4.2	16.5	37.8	C43 H62 N7 O14
	900.4467	-7.4	-8.2	16.5	52.6	C42 H62 N9 O13

NMR and HRMS Compound 26: Chlorodactyloferrin-3-pyridyl rifampicin gallium complex Bruker-600

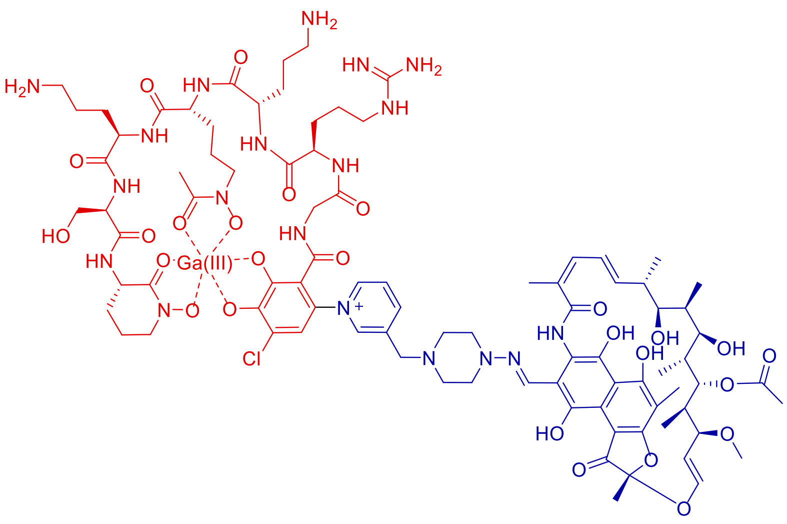

A) Comp. 26:gallium: 1D-^{1}H wet (11ms). NMR spectrum in CD_3OD at 14 T and 293 K.

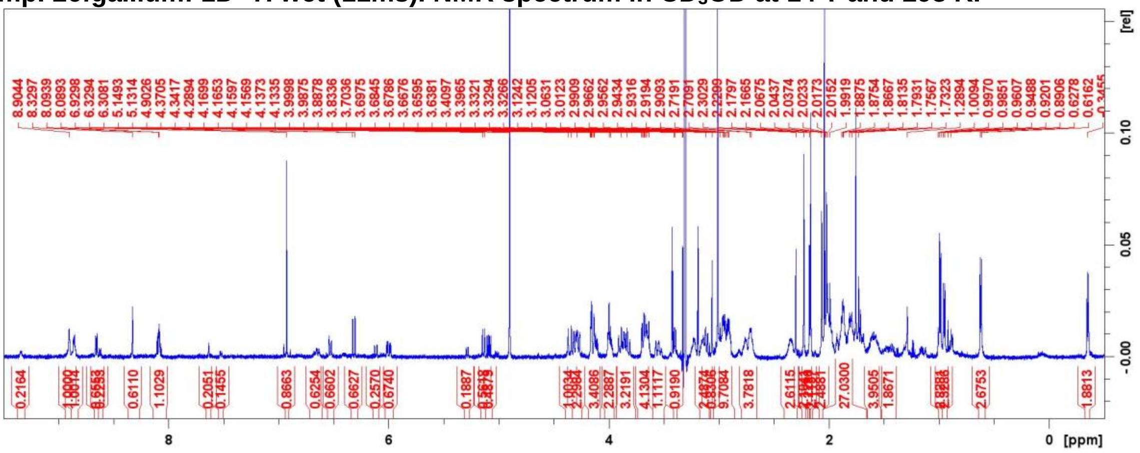

B) Comp. 26:gallium: 2D-^{1}H-ROESY (*T*m = 400 ms). NMR spectrum in CD_3OD at 14 T and 293 K.

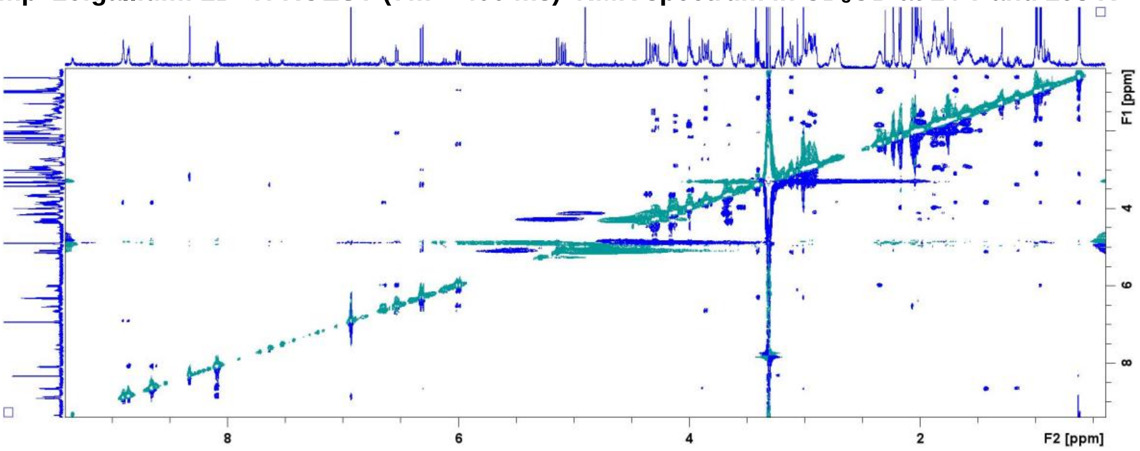

C). Comp. 26:gallium: 2D-^{1}H-^{13}Caliphatic-HSQC. NMR spectrum in CD_3OD at 14 T and 293 K.

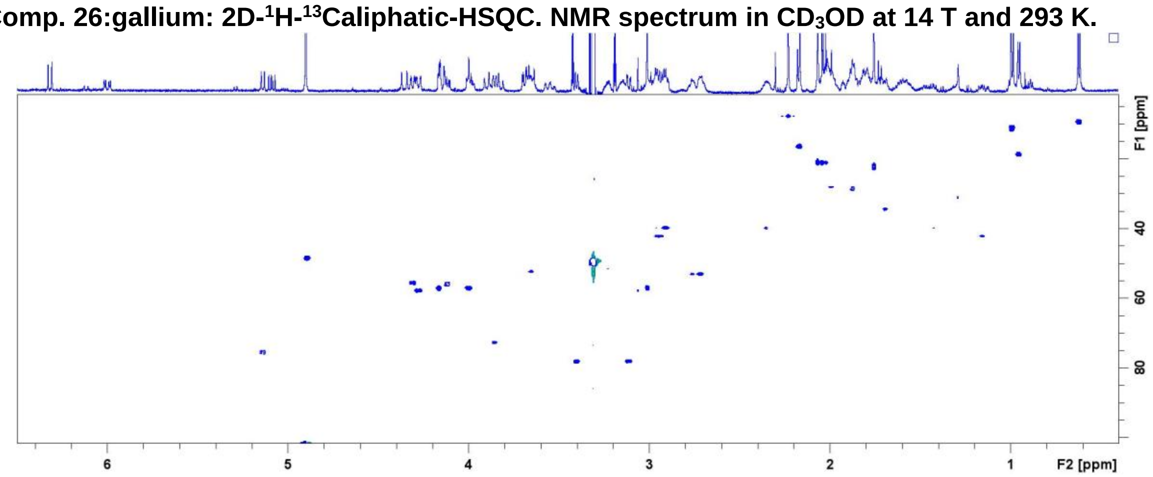

D). Comp. 26:gallium: 2D-^{1}H-^{13}Caromatic-HSQC. NMR spectrum in CD_3OD at 14 T and 293 K.

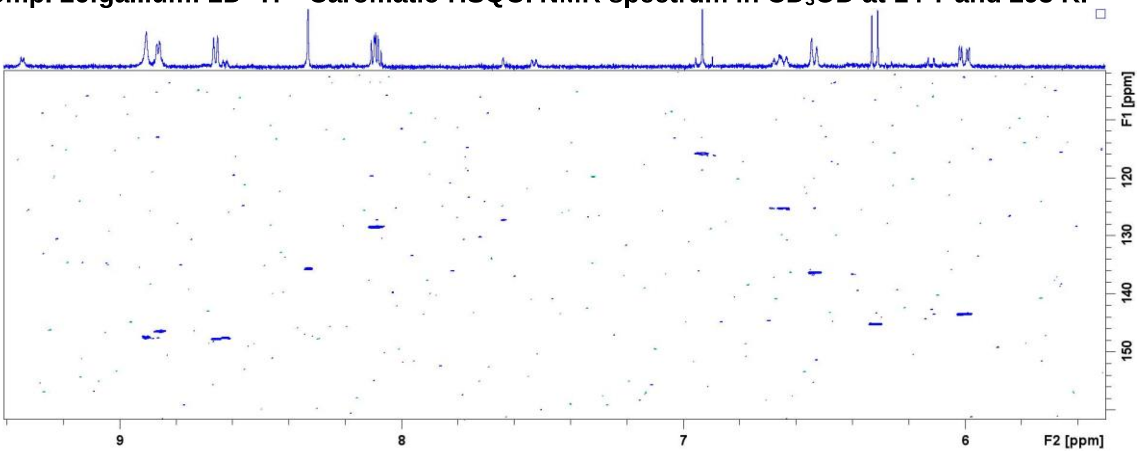

E) Comp. 26:gallium: 2D-^{1}H-^{13}C-HMBC (*J*app = 10 Hz). NMR spectrum in CD_3OD at 14 T and 293 K.

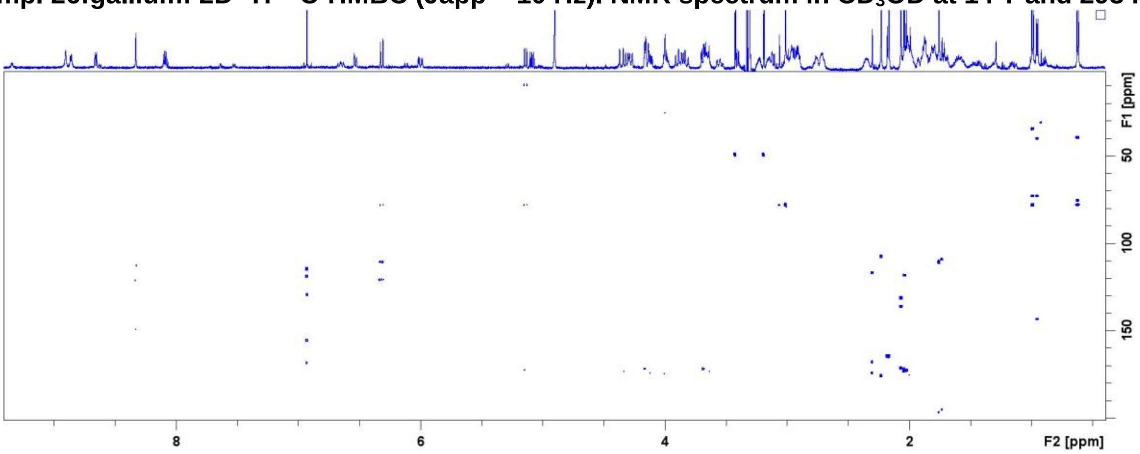

Comp 26:iron. HRMS (ESI+) analysis

Single Mass Analysis

Tolerance = 5.0 mDa / DBE: min = -1.5, max = 50.0
Element prediction: Off
Number of isotope peaks used for i-FIT = 3

Monoisotopic Mass, Odd and Even Electron Ions
758 formula(e) evaluated with 4 results within limits (up to 50 best isotopic matches for each mass)
Elements Used:
C: 0-90 H: 0-125 N: 15-20 O: 0-30 Cl: 0-1 Fe: 0-1

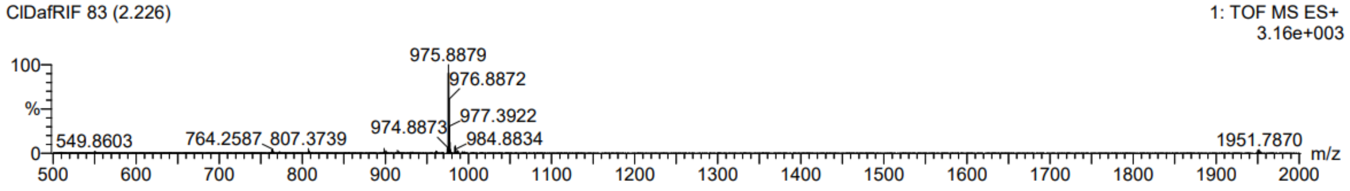

		mDa	PPM	DBE	i-FIT	Formula
Minimum:				-1.5		
Maximum:		5.0	10.0	50.0		

Mass	Calc. Mass	mDa	PPM	DBE	i-FIT	Formula
1951.7870	1951.7846	2.4	1.2	37.0	15.6	C88 H122 N19 O26 Cl Fe
	1951.7860	1.0	0.5	36.5	16.0	C90 H124 N16 O27 Cl Fe
	1951.7906	-3.6	-1.8	40.5	16.2	C88 H116 N20 O29 Cl
	1951.7919	-4.9	-2.5	40.0	16.6	C90 H118 N17 O30 Cl

NMR and HRMS Compound 28: Chlorodactyloferrin 3-pyridyl norfloxacin gallium complex Bruker-600

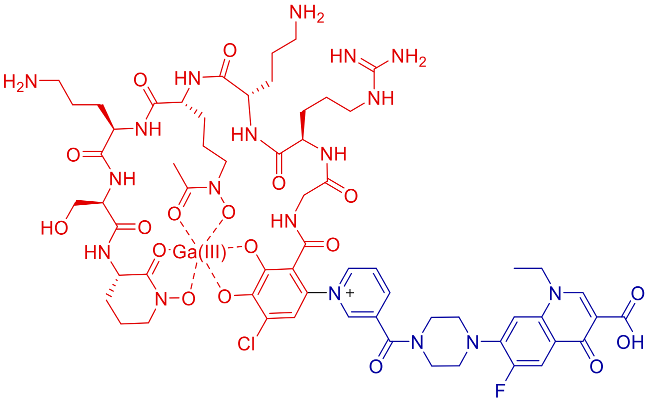

A) Comp 28:gallium: 1D-^{1}H. NMR spectrum in CD_3OD at 14 T and 293 K.

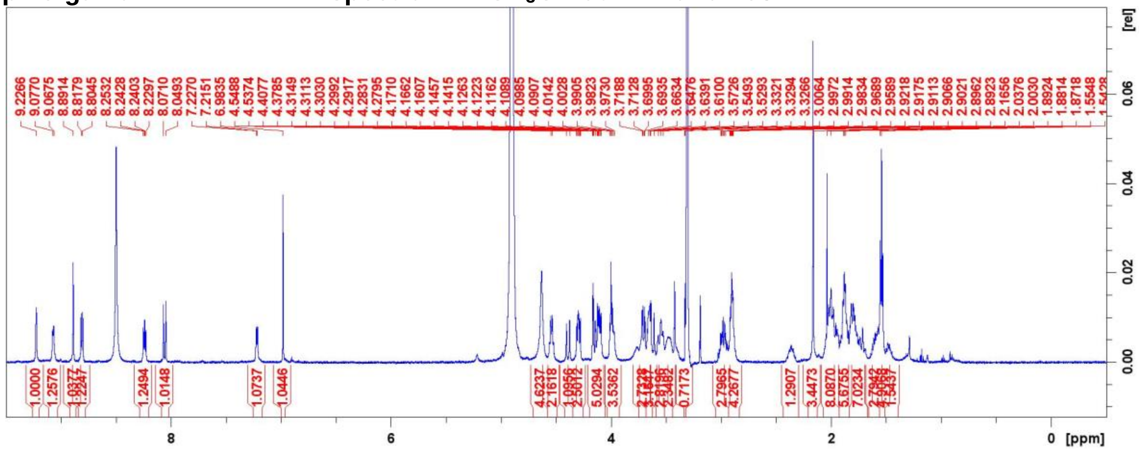

B) Comp 28:gallium: 2D-^{1}H-ROESY (*T*m = 400 ms). NMR spectrum in CD_3OD at 14 T and 293 K.

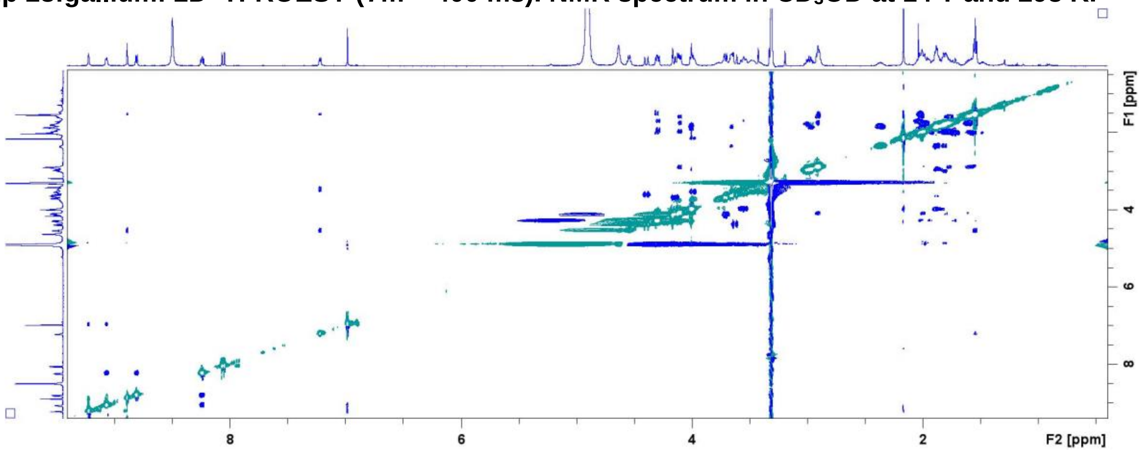

C). Comp 28:gallium: 2D-^{1}H-^{13}Caliphatic-HSQC. NMR spectrum in CD_3OD at 14 T and 293 K.

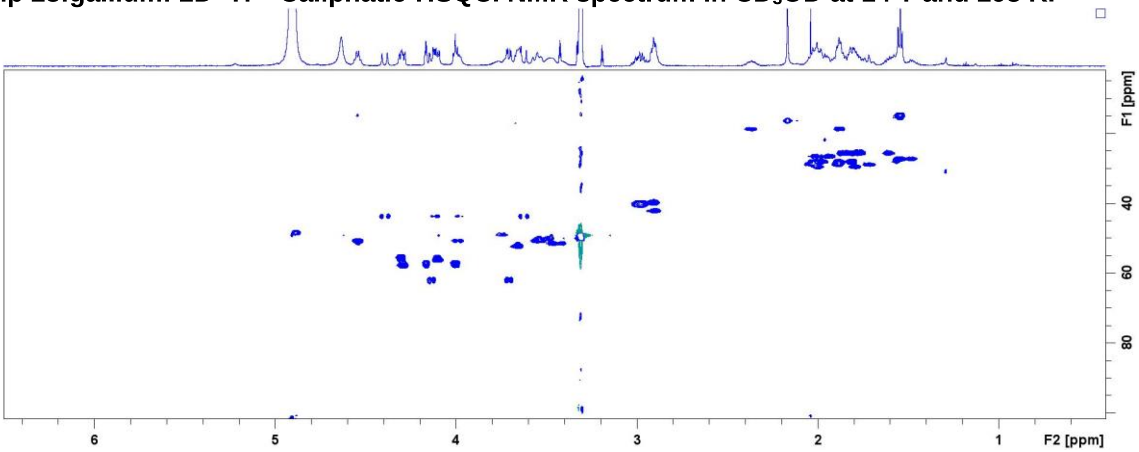

D). Comp 28:gallium: 2D-^{1}H-^{13}Caromatic-HSQC. NMR spectrum in CD_3OD at 14 T and 293 K.

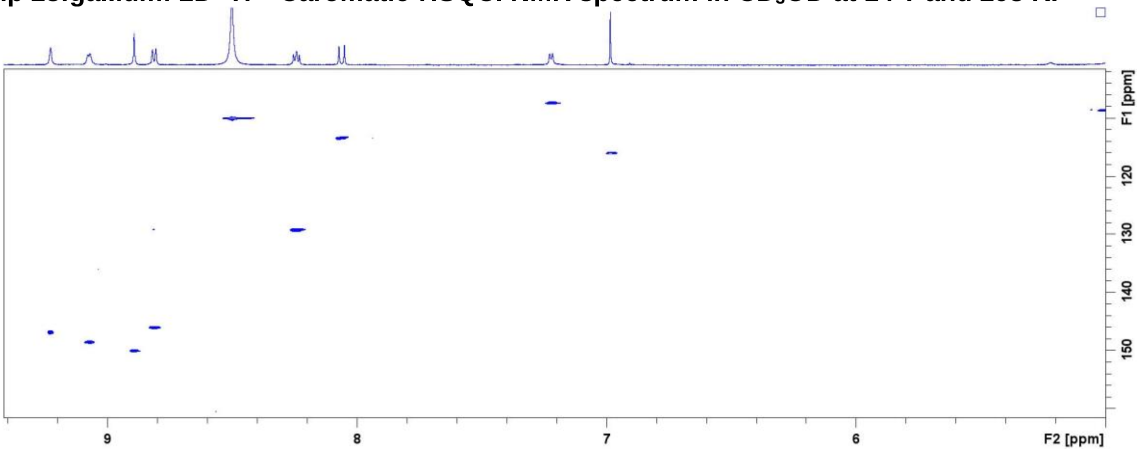

E) Comp 28:gallium: 2D-^{1}H-^{13}C-HMBC (*J*app = 10 Hz). NMR spectrum in CD_3OD at 14 T and 293 K.

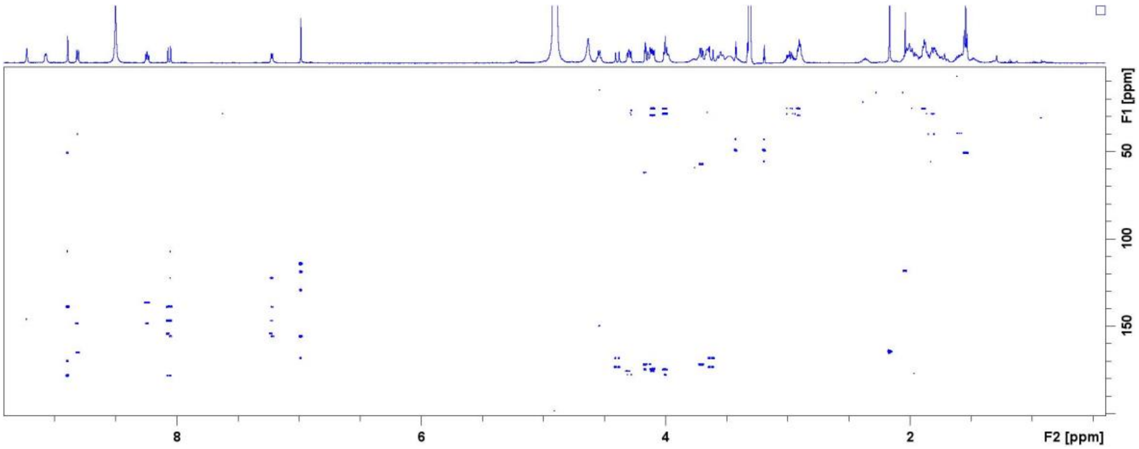

F) Comp 28:gallium: 1D-$^{19}F\{^{1}H\}$. NMR spectrum in CD_3OD at 14 T and 293 K.

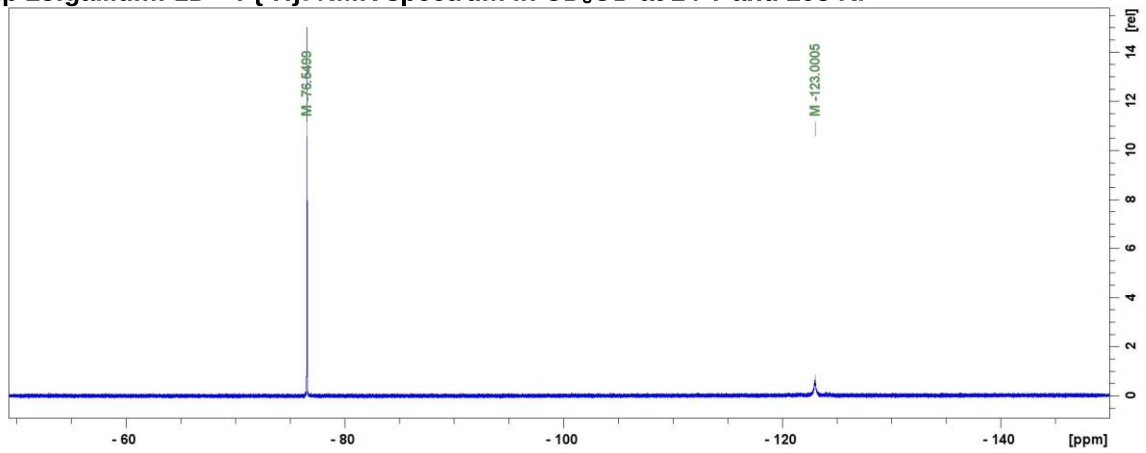

Comp 28:iron. HRMS (ESI+) analysis

Single Mass Analysis
Tolerance = 5.0 mDa / DBE: min = -1.5, max = 50.0
Element prediction: Off
Number of isotope peaks used for i-FIT = 3

Monoisotopic Mass, Odd and Even Electron Ions
942 formula(e) evaluated with 6 results within limits (up to 50 best isotopic matches for each mass)
Elements Used:
C: 60-65 H: 0-100 N: 16-20 O: 0-20 F: 0-1 Cl: 0-1 Fe: 0-1

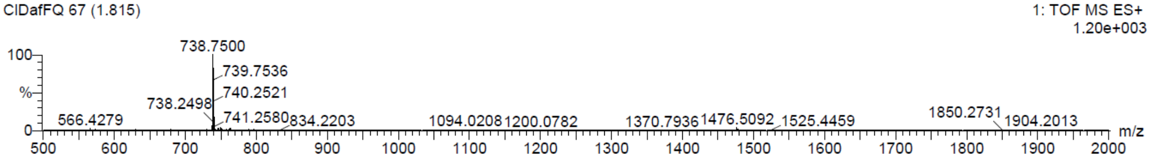

Minimum:				-1.5		
Maximum:		5.0	10.0	50.0		
Mass	Calc. Mass	mDa	PPM	DBE	i-FIT	Formula
1476.5092	1476.5076	1.6	1.1	30.0	2.1	C62 H82 N18 O18 F Cl Fe
	1476.5124	-3.2	-2.2	37.5	2.3	C65 H75 N19 O20 Cl
	1476.5065	2.7	1.8	34.0	2.4	C65 H81 N18 O17 Cl Fe
	1476.5103	-1.1	-0.7	34.5	2.5	C65 H80 N19 O15 F Cl Fe
	1476.5058	3.4	2.3	35.0	4.5	C63 H77 N20 O18 F Fe
	1476.5072	2.0	1.4	34.5	4.5	C65 H79 N17 O19 F Fe
